# Identification of N-linked Glycoproteins in Silkworm Serum Using Con A Lectin Affinity Chromatography and Mass Spectrometry

**DOI:** 10.1093/jisesa/ieab057

**Published:** 2021-08-17

**Authors:** Zhaoming Dong, Lin Ye, Yan Zhang, Zhiyong Chen, Benchi Li, Tao Zhang, Ping Zhao

**Affiliations:** 1 State Key Laboratory of Silkworm Genome Biology, Southwest University, Chongqing 400715, China; 2 Biological Science Research Center, Southwest University, Chongqing 400715, China; 3 Chongqing Key Laboratory of Sericultural Science, Southwest University, Chongqing 400715, China

**Keywords:** insect, N-glycan, glycosylation, glycoprotein, hemolymph

## Abstract

Glycosylation is one of the most common post-translational modifications to occur during protein biosynthesis, but remains poorly understood in insects. In this study, we collected serum proteins from two silkworm developmental stages, namely day 7 of the fifth instar larval stage and day 2 of the pupal stage. Results of SDS–PAGE and periodic acid-Schiff staining revealed that most serum proteins with high abundance were putative glycoproteins. LC-MS/MS identified 149 larval and 303 pupal serum proteins in the Con A lectin-enriched fractions. GO analysis revealed that many serum proteins were involved in the proteolysis and carbohydrate metabolic process. 82 N-linked glycoproteins with at least one glycosylation site were identified. N-Linked glycosylation occurred at the sequon, Asn-X-Ser/Thr, and the proportions of Ser and Thr glycosylation at the hydroxy position were found 39.6% and 60.3%, respectively. The N-glycan structures found in serum glycoproteins were mainly Man_2_FucGlcNAc_2_ (67.9%). Since storage protein 1 and transferrin had a relatively high abundance in the serum and could be significantly enriched by Con A lectin, their glycosylation was analyzed in detail. Glycoside hydrases, serine proteases and serpins were found to form three interacting glycoprotein networks using the website STRING. This study provides important clues for the understanding of the function of N-linked glycosylation in metabolism, immunity, and metamorphosis.

## Graphical Abstract

**Figure:**
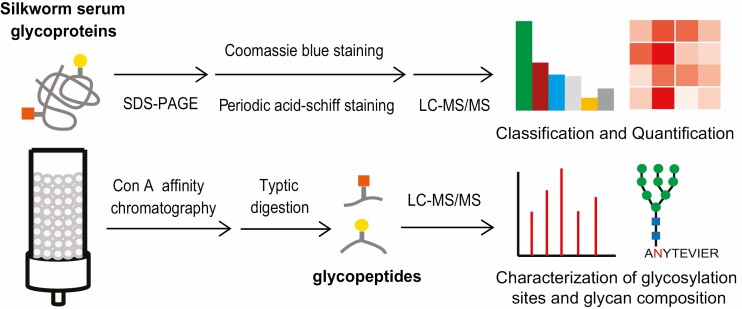

Glycosylation is one of the most common post-translational modifications (PTM) and plays an important role in different biological processes (Bertozzi and Kiessling 2001). It determines the localization, activity, and function of proteins and thus regulates various cellular processes (Kaji et al. 2003), including cell adhesion, molecular trafficking and clearance, receptor activation, signal transduction, and endocytosis. Protein glycosylation mainly involves N-glycans and O-glycans. N-glycans are linked to asparagine residues, whereas O-glycans are attached to serines and threonines (Schachter 2000).

Research on mammalian glycosylation has contributed to the diagnoses and treatment of various diseases. An increasing number of diseases related to glycosylation have been discovered, which are primarily severe syndromes (Ohtsubo and Marth 2006). Since glycoproteins comprise a major part of the serum proteome, and serum is the most commonly used fluid for diagnostics, researchers explored serum glycoproteins through lectin affinity chromatography and identified a large number of glycoproteins (Yang and Hancock 2004, Madera et al. 2007). Many of the serum glycoproteins were identified as transferrins, protease inhibitors, apolipoproteins, ceruloplasmins, and complement factors.

Insect glycoproteins have N-glycans and O-glycans with core structures similar to those produced by other eukaryotes, but they also contain some unique glycans that mediate specific functions (Walski et al. 2017, Scheys et al. 2018, Li et al. 2020). Insect glycoproteins fail to acquire antennae of the N-acetyllactosamine sugar or peripheral sugars and thus have few complex N-glycans (Chung et al. 2017). The major N-glycan structure found in insect glycoproteins is the paucimannose-type Man_3_(Fuc)GlcNAc_2_ (Yehuda and Padler-Karavani 2020).

In *Drosophila*, 138, 185, and 148 glycoproteins were identified by using *Galanthus nivalis* agglutinin (GNA), *Nicotiana tabacum* lectin (Nictaba), and *Rhizoctoni solani* agglutinin (RSA). GNA has a high specificity for paucimannose and highmannose N-glycans (Fouquaert et al. 2009), and Nictaba recognizes the Man_3_GlcNAc_2_ core of N-glycans (Lannoo et al. 2006), while RSA shows a high affinity for some mucin type O-glycans and terminal Gal/GalNAc residues on complex N-glycans (Blixt et al. 2004). Gene ontology (GO) analysis indicated that *Drosophila* glycoproteins were mainly involved in cell–cell adhesion, metabolic process, cell respiration, glycolysis, and tricarboxylic acid cycle (Vandenborre et al. 2010). In honeybees, 25 N-glycosylated royal jelly proteins with 53 N-glycosylation sites were identified, most of which were related to metabolic activities and health improvement (Zhang et al. 2014a). Some researchers identified 130, 49, 87, 118, and 218 putative glycoproteins from flour beetle (*Tribolium castaneum*), silkworm (*Bombyx mori*), honey bee (*Apis mellifera*), fruit fly (*Drosophila melanogaster*), and pea aphid (*Acyrthosiphon pisum*) by using GNA affinity chromatography and LC-MS/MS, and found that many glycoproteins were related to metabolic processes and hydrolase activity (Vandenborre et al. 2011).

In this study, we analyzed the serum proteins of silkworm larvae, pupae, and moths using periodic acid-Schiff staining protein gel and mass spectrometry. Furthermore, we used concanavalin A (Con A) lectin to enrich N-linked glycoproteins from the silkworm serum at two developmental stages, the larval stage and the pupal stage. LC-MS/MS was employed to identify N-linked glycoproteins. GO annotation was performed using the Singular Enrichment Analysis tool in the website AgriGO, and the protein interaction network model was constructed using the website STRING. The silkworm glycoproteins from different developmental stages were compared and analyzed for the first time. Our data indicated that N-linked glycoproteins may play important roles in the immunity and metamorphosis in the silkworm.

## Materials and Methods

### Serum Collection

*Bombyx mori* strain p50 (DaZao) silkworms were reared on mulberry leaves at 25°C. Hemolymph was collected at nine-time points, including days 1/3/5/7 of the fifth instar, day 2 of wandering, days 1/4/7 of the pupal stage, and day 1 of the moth stage. In each time points, four tubes of hemolymph were collected. Serum was collected by centrifuging the hemolymph at 4,000 g for 20 min at 4°C.

### Electrophoresis and Staining

Two microliters of serum protein was separated by sodium dodecyl sulfate-polyacrylamide gel electrophoresis (SDS–PAGE) on 8% SDS-polyacrylamide gel. Following SDS–PAGE, separated proteins were stained with coomassie blue. For periodic acid-Schiff staining, protein gel was soaked in 7.5% (v/v) acetic acid for 30 min and then transferred in 0.2% (w/v) periodic acid for 1 hr at 4°C. After removing the periodic acid solution, the gel was incubated with the Schiff reagent for 1 hr at 4°C in the dark. The gel was soaked in 7.5% acetic acid for 1 hr and subsequently stored in water.

### Processing of Protein Samples From Polyacrylamide Gel

Protein bands were excised from the polyacrylamide gel, placed in centrifuge tubes, and incubated with 100 µl decolorization solution (15 mM potassium ferric chloride and 50 mM sodium thiosulfate). After removing the decolorization solution, excised gel bands were washed three times with Milli-Q water and then incubated with 100% acetonitrile. The acetonitrile was removed when the gel appeared milky. The gel was then allowed to dry for 20 min, following which the gel bands were incubated with trypsin (10 μg/ml) at 37°C for 24 hr. Tryptic peptides were extracted from gels, by soaking in 25 µl of 50% acetonitrile/5% trifluoroacetate for 60 min, concentrated, and resuspended in 0.1% formic acid.

### Con A Affinity Chromatography

A Con A Sepharose 4B (GE Healthcare, USA) column (1 ml) was equilibrated with 5 ml binding buffer (20 mM Tris–HCl, 150 mM NaCl, 1 mM MgCl_2_, 1 mM CaCl_2_, 1 mM MnCl_2_, pH 7.4). Serum (0. 4 ml) from day 7 of the fourth larval instar and day 2 of the pupal stage was diluted five times with binding buffer and then loaded onto the Con A column. After washing the column with 10 ml binding buffer, Con A-bound proteins were eluted with 5 ml 300 mM α-D-mannose in the binding buffer. Con A affinity chromatography was performed in four biological replicates.

### Liquid Chromatography-Tandem Mass Spectrometry

Con A-enriched proteins were reduced with 10 mM dithiothreitol (DTT) for 150 min at 37°C and then alkylated with 50 mM iodoacetamide (IAA) for 40 min in the dark. After washing twice with 50 mM NH_4_HCO_3_, proteins were incubated with trypsin (1 μg/50 μg protein) overnight at 37°C in 150 µl of 50 mM NH_4_HCO_3_. Tryptic peptides were concentrated and resuspended in 0.1% formic acid.

Tryptic peptides were separated using the Thermo Fisher Scientific EASY-nLC 1000 system and EASY-Spray column (C18, 2 μm, 100 Å, 75 μm × 50 cm) with a 2–100% acetonitrile gradient in 0.1% formic acid over 180 min at a flow rate of 250 nl/min. The separated peptides were analyzed using a Thermo Scientific Q Exactive mass spectrometer operating in data-dependent mode. Up to 10 of the most abundant isotope patterns with charge ≥2 from an initial survey scan were automatically selected for fragmentation by higher energy collisional dissociation with normalized collision energies of 27%. The maximum ion injection times for the survey scan and the MS/MS scans were 20 and 60 ms, respectively, and the ion target value for both scan modes was set to 1E6. An 18-s dynamic exclusion of previously sequenced ions was applied.

### Protein Identification and Quantification

The resulting raw MS data were analyzed with the MaxQuant software (version 1.3.0.1) (Cox and Mann 2008). MaxQuant searches were executed against an integrated silkworm proteome database containing protein sequences from silkDB, Genkbank, and Uniprot. Peptide searches were performed with the Andromeda search algorithms (Cox et al. 2011). The search parameters for protein identification specified the initial precursor and fragment mass tolerances of 6 and 20 ppm, respectively. Carbamidomethylation of cysteine was set as a fixed modification, and N-terminal protein acetylation and methionine oxidation were set as variable modifications. The minimal peptide length was set to six amino acids, and up to two miscleavages were allowed. The false discovery rate was set to 0.01 for both peptides and proteins. All common contaminants and reverse hits were removed. A minimum of one unique peptide was required for an identified protein. For comparison of protein abundance, we used the intensity-based absolute quantification (iBAQ) algorithm in MaxQuant. To compare the protein abundance in the Con A-binding fraction with that of total serum protein, an unpaired *t*-test was used with a significance level set at *P* < 0.05. The heat maps of protein abundance were generated using HemI (Heatmap Illustrator, version 1.0.3.3) (Deng et al. 2014).

### Glycoprotein Identification

The raw MS data were searched with the Byonic software (version 2.13.17) against the Con A-enriched serum protein database (Bern et al. 2012). N-glycosylation was searched for with the following search parameters: precursor mass tolerance = 10 ppm, fragmentation type = QTOF/HCD, fragment mass tolerance = 0.02 Da; a maximum of two missed cleavages was allowed; modifications: Carbamidomethyl/+57.021464 @ C (fixed), Oxidation/+15.994915 @ M (common1), Deamidated/+0.984016 @ N, Q (common1), Acetyl/+42.010565 @ Protein NTerm (common1), insect N-glycan (Glycan modifications). Data with a Byonic score ≥200, Delta Mod ≥10, |Log Prob| ≥3, number of unique peptides >1, protein false discovery rate (FDR) <1% was filtered. Furthermore, a manual inspection was performed for the quality of each glycopeptide.

### Bioinformatics Analysis

The GO annotation was performed using the Singular Enrichment Analysis tool in the website AgriGO (Tian et al. 2017). The enrichment value of each GO term was defined as the ratio of protein number in the input list to protein number in the reference. The protein interaction network model was constructed by using the website STRING (Szklarczyk et al. 2011). The small interaction networks with less than five proteins were excluded. The domains and functions of proteins were annotated automatically by STRING.

## Results

### Visualization of Glycoproteins in the Silkworm Serum

**Fig. 1.**
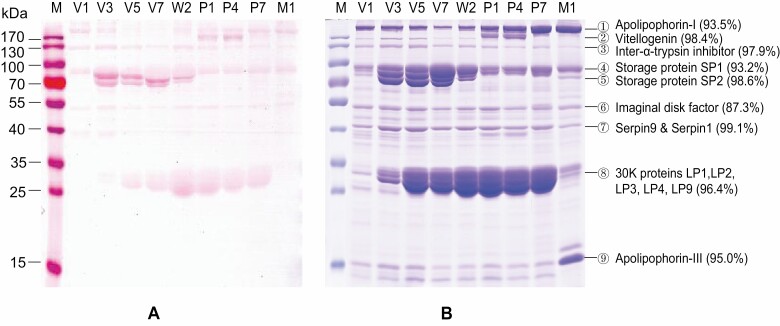
Electrophoretic analysis of serum proteins. (A) Periodic acid-Schiff staining following SDS–PAGE of serum protein. (B) Coomassie blue staining following SDS–PAGE of serum proteins. Two microliters of serum protein was run on an 8% polyacrylamide gel. M, protein marker; V1/3/5/7, days 1/3/5/7 of the fifth instar; W2, day 2 of wandering; P1/4/7, days 1/4/7 of pupae; M1, day 1 of moth. The horizontal lines connected by numbers indicate protein bands. The proteins from each band were identified using a Q Exactive mass spectrometer and MaxQuant software. The abundance proportion of the major proteins in each band was calculated according to their iBAQ intensity (Table 1 and Supp Table 1 [online only]).

Silkworm serum proteins were collected from nine developmental stages, including five larval stages, three pupal stages, and one adult stage, and were then separated with SDS–PAGE (Fig. 1). To visualize the serum glycoproteins, protein gels were stained with periodic acid-Schiff staining (Fig. 1A) and coomassie blue (Fig. 1B). Most proteins were stained with both reagents, including proteins with a molecular weight of >170 kDa (protein band 1), 170 kDa (protein band 2), 130 kDa (protein band 3), 90 kDa (protein band 4), 80 kDa (protein band 5), 50 kDa (protein band 6), 40 kDa (protein band 7), 30 kDa (protein band 8) (Fig. 1). Gel-based mass spectrometry identification revealed that each protein band is not a single protein, but contains 6–24 proteins (Supp Table 1 [online only]). However, the major proteins in each band occupy a very high abundance from 87.3% to 99.1% (Table 1). The major proteins in bands 1–8 are as follows: apolipophorin-I (93.5% in protein band 1), vitellogenin (98.4% in protein band 2), inter-alpha-trypsin inhibitor (97.9% in protein band 3), storage protein SP1 (93.2% in protein band 4), storage protein SP2 (98.6% in protein band 5), imaginal disk factor (87.3% in protein band 6), serpins (serpin9 and serpin1) (99.1% in protein band 7), and 30K proteins (Bmlp1, Bmlp2, Bmlp3, Bmlp4, and Bmlp9) (96.4% in protein band 8) (Fig. 1, Table 1 and Supp Table 1 [online only]), which are putative glycoproteins. Protein band 9 was identified as apolipophorin-III (95.0% in Band 9) (Fig. 1, Table 1 and Supp Table 1 [online only]), which could not be stained with periodic acid-Schiff staining and thus may not be a glycoprotein (Fig. 1).

**Table 1.** Identification of the major silkworm hemolymph proteins

Band No.	Annotated name^*a*^	Uniprot ID	Genebank ID	Peptide number	Sequence coverage [%]	MW [kDa]	Relative abundance^*b*^
Band 1	Apolipophorin-I	tr|G1UIS8|	gi|1200716789	226	62.5%	369	93.5%
Band 2	Vitellogenin	sp|Q27309|	–	79	54	203	98.4%
Band 3	Inter-alpha-trypsin inhibitor heavy chain	–	gi|1200719391	27	27.1	99	97.9%
Band 4	Storage protein 1	sp|P09179|	–	89	73.4	87	93.2%
Band 5	Storage protein 2	–	gi|1174445	76	72.3	83	98.6%
Band 6	Imaginal disk growth factor	–	gi|1200731398	21	42.2	49	87.3%
Band 7	Serpin9	sp|Q03383|	–	15	37	45	62.6%
Band 7	Serpin1	tr|C7ASM9|	–	26	64.3	43	36.5%
Band 8	BmLP1	sp|P09334|	–	26	77	30	39.2%
Band 8	BmLP2	tr|H9B444|	–	25	59.8	30	16.1%
Band 8	BmLP4	sp|Q00801|	–	20	62.4	30	14.7%
Band 8	BmLP3	sp|Q00802|	–	26	68.4	30	14.5%
Band 8	BmLP9	tr|H9J4G0|	–	33	57.6	44	11.8%
Band 9	Apolipophorin-III	tr|H9JU96|	–	14	48.2	28	95.0%

^*a*^“BmLP’’ are nomenclature of *B. mori* 30K proteins reported by Zhang et al. (2012).

^*b*^Relative abundance was calculated according to the iBAQ intensity of each protein in Supp Table 1 (online only).

### Shotgun Proteomics Analysis of Con A Lectin-Enriched Proteins in the Silkworm Serum

**Fig. 2.**
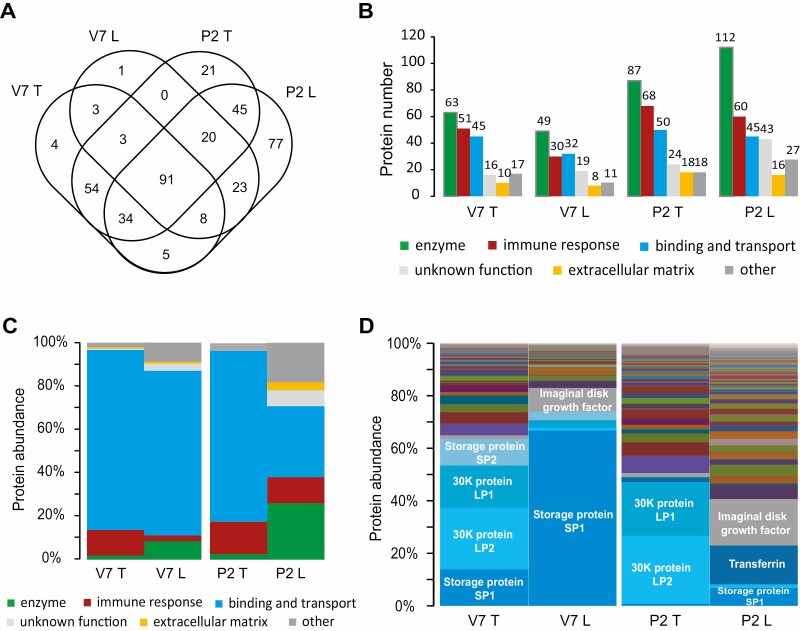
Identification, classification, and quantification of total serum proteins and Con A lectin-enriched serum proteins. (A) The Venn diagram shows 202 total serum proteins from day 7 of the fifth larval instar (V7 T), 149 lectin-enriched serum proteins from day 7 of the fifth larval instar (V7 L), 268 total serum proteins from day 2 of pupae (P2 T), 303 lectin-enriched serum proteins from day 2 of pupae (P2 L). (B) Numbers of proteins from functional categories in each sample. (C) Proportions of proteins from functional categories in each sample. (D) Proportions of proteins in each sample. Relative molar abundance of proteins from each category was calculated based on iBAQ intensity.

The Con A lectin affinity column was used to enrich N-linked glycoproteins from silkworm serum at two developmental stages, day 7 of the larval fifth instar (V7) and day 2 of the pupal stage (P2). LC-MS/MS was employed to identify Con A lectin-enriched serum proteins. Combining the results of four biological replicates, we identified 202 larval serum proteins (V7 T), 149 lectin-enriched larval serum proteins (V7 L), 268 pupal serum proteins (P2 T), and 303 lectin-enriched pupal serum proteins (V7 T) (Fig. 2ASupp Table 2 [online only]). A total of 389 serum proteins were identified by LC-MS/MS, which were classified into six categories based on annotated molecular function according to previous reports (Dong et al. 2016, Chen et al. 2019), including enzyme (135), immune response (84), binding and transport (63), extracellular matrix (22), unknown function (50), and other function (35). By comparing protein numbers between serum proteins and lectin-enriched serum proteins, we found that more enzymes and proteins of unknown function were observed in lectin-enriched pupal serum proteins (Fig. 2B).

We estimated protein abundance using LC−MS/MS and the iBAQ algorithm and found that enzymes and proteins of unknown function showed higher abundance in lectin-enriched larval and pupal serum when compared to normal serum (Fig. 2C). Some proteins were highly abundant in serum but were not enriched by Con A lectin (Fig. 2D), including storage protein 2 (Genebank ID: gi|95103012), 30K proteins BmLP1 (Genebank ID: gi|379046494), and BmLP2 (Genebank ID: gi|379046488). Some proteins with high abundance in serum were enriched by Con A lectin (Fig. 2D), including storage protein 1 (Genebank ID: gi|1335609), imaginal disk growth factor (Genebank ID: gi|152061158), and transferrin (SilkDB ID: BGIBMGA011424).

### GO Analysis of Con A Lectin-Enriched Proteins in the Silkworm Serum

**Fig. 3.**
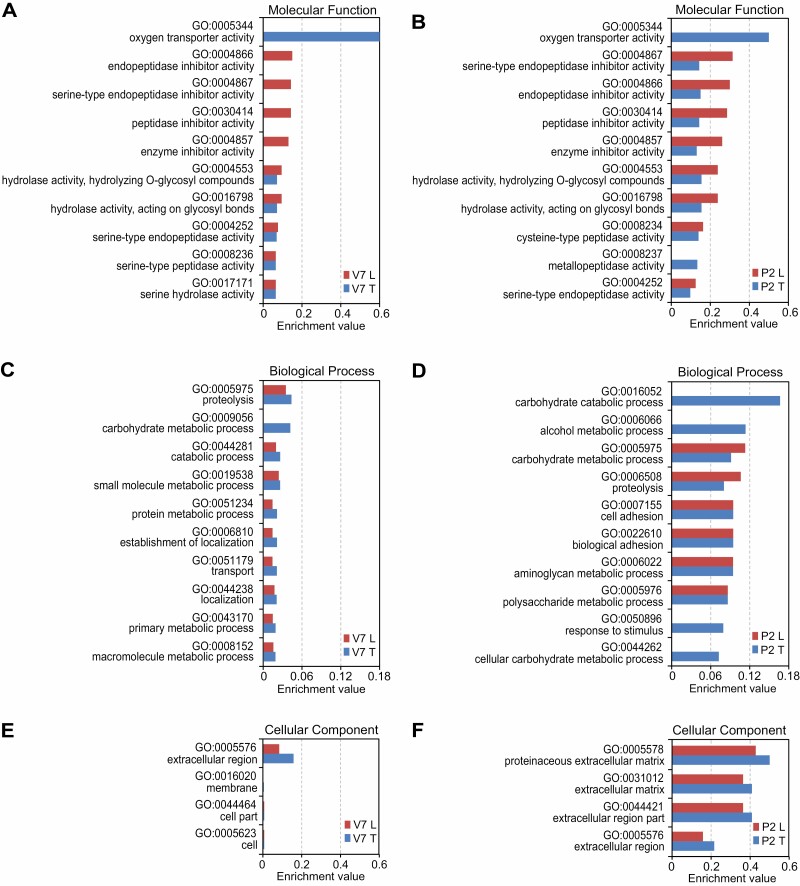
Gene Ontology analysis of total serum proteins and Con A lectin-enriched serum proteins. GO terms enrichment analysis was performed using AgriGO. GO terms were divided into three parts: molecular function (A, B), biological process (C, D), and cellular component (E, F). GO terms are labeled using the GO number, term definition, and enrichment value. GO terms of total serum proteins from day 7 of the fifth larval instar (V7 T) and that from day 2 of pupae (P2 T) are showed as blue bars, and GO terms of lectin-enriched serum proteins from day 7 of the fifth larval instar (V7 L) and that from day 2 of pupae (P2 L) are red bars.

The GO annotation of silkworm serum proteins at two developmental stages was predicted by using the Singular Enrichment Analysis tool in AgriGO. In the molecular function, oxygen transporter activity (GO:0005344) is the most important GO term in the total serum proteins (Fig. 3A and B), and not found in the lectin-enriched serum proteins, indicating that most of the proteins with oxygen transporter activity may not be glycoproteins. The lectin-enriched larval and pupal serum proteins share similar molecular function GO terms, including peptidase/protease inhibitor activity (GO:0004866, GO:0004867, GO:0030414, GO:0004857), peptidase/protease activity (GO:0004252, GO:0008236, GO:0017171), and glycoside hydrolase activity (GO:0004543, GO:0016798). In the biological process, proteolysis (GO:0005975) and carbohydrate metabolic process (GO:0009056) are two important GO terms for lectin-enriched larval and pupal serum proteins (Fig. 3C and D). The GO terms of the biological process are highly correlated with the GO terms of molecular function. For instance, the carbohydrate metabolic process is related to the glycoside hydrolase activity, while the proteolysis is associated with the peptidase/protease activity and peptidase/protease inhibitor activity. Interestingly, the adhesion GO terms (GO:0007155 and GO:0022610) are unique for lectin-enriched pupal serum proteins but not for lectin-enriched larval serum proteins (Fig. 3C and D). In the cellular component, the extracellular region GO terms (GO:0005576, GO:0044421) and extracellular matrix GO terms (GO:0005578, GO:0031012) indicated that serum proteins are secreted out of the cell to play roles (Fig. 3E and F).

### Identification of Silkworm Serum Proteins That Were Significantly Enriched by Con A Lectin

**Fig. 4.**
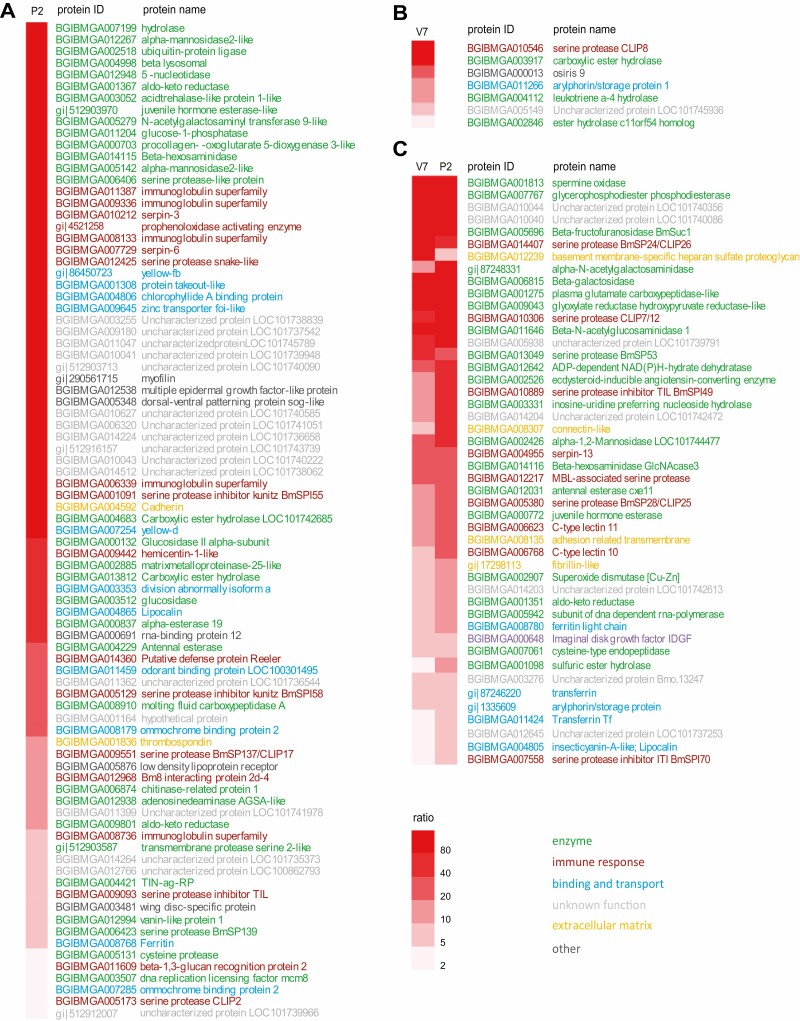
Con A lectin-binding ability of proteins from silkworm serum. A total of 139 proteins were significantly enriched by Con A lectin according to the following conditions: the ratio of protein abundance >2 and *P* < 0.05. Con A lectin-binding ability was quantified according to the iBAQ intensity of the Con A lectin-binding protein divided by the iBAQ intensity of the extracted protein. This heat map was generated using HemI. Colors indicate the protein category: enzymes, green; immune response proteins, red; binding and transport proteins, blue; proteins of unknown function, light grey; extracellular matrix protein, orange; other, dark grey.

To determine which proteins were significantly enriched by Con A lectin, we calculated the ratio of protein abundance as (enriched/unenriched) >2 and considered *P* < 0.05 to be statistically significant. We found that a total of 139 glycoproteins were significantly enriched by Con A lectin, including 55 enzymes, 28 immune-related proteins, 25 proteins of unknown function, 17 binding and transport proteins, six extracellular matrix proteins, and eight other proteins (Fig. 4 and Supp Table 2 [online only]). The enzymes mainly contain glycoside hydrolases, esterases, and peptides/proteases, and the immune-related proteins are composed of immunoglobulins, serine proteases, serine protease inhibitors, and lectins.

### Identification of N-linked Glycoproteins in the Silkworm Serum

**Fig. 5.**
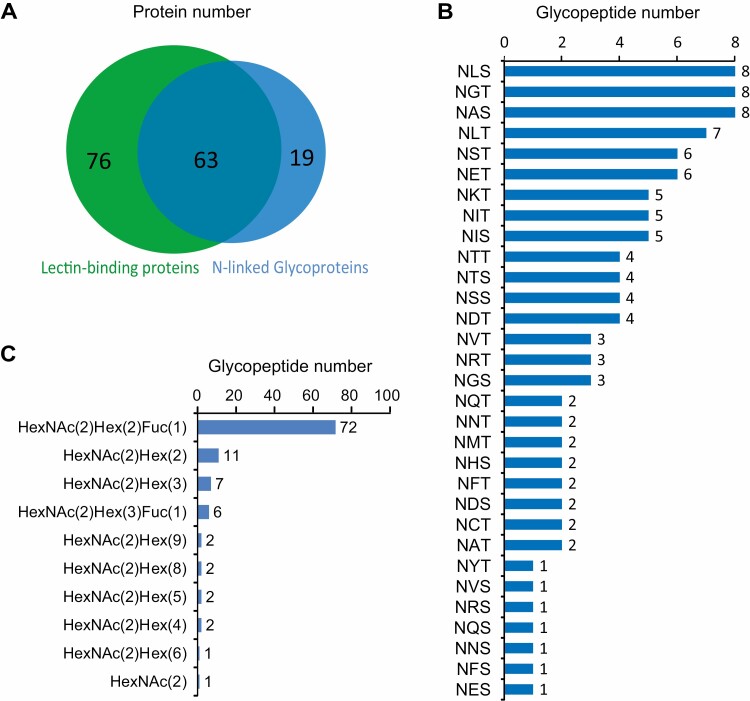
N-Linked glycoproteins and glycopeptides in silkworm serum. (A) The Venn diagram shows 139 ConA lectin-binding proteins and 82 N-linked glycoproteins. (B) The horizontal bar chart shows the sequons where N-Linked glycosylation occurs. (C) The horizontal bar chart shows the glycoforms of N-Linked glycosylation.

Through the Byonic software search, we identified 82 N-linked glycoproteins with at least one glycosylation site, 63 of which were significantly enriched by Con A lectin (Fig. 5A and Supp Table 3 [online only]).

One to four glycosylation sites were found in each N-linked glycoprotein. We found that N-Linked glycosylation occurred at the sequon, Asn-X-Ser/Thr (Fig. 5B, Supp Table 4, and Supp Fig. 1 [online only]), which was consistent with previous reports (Gavel and von Heijne 1990, Shakin-Eshleman et al. 1996). The proportions of Ser and Thr at the hydroxy position were found to be 39.6% and 60.3%, respectively. The proportions of Leu, Gly, Ile, Ala, and Ser in the X position were found to be 14.2%, 10.3%, 9.4%, 9.4%, and 9.4%, respectively, whereas Tyr, Cys, His, and Met accounted for 0.9%, 1.8%, 1.8%, and 1.8%, respectively. No Trp or Pro were identified at the X position.

The N-glycan structures found on serum glycoproteins were mainly HexNAc(2)Hex(2)Fuc(1) (67.9%), HexNAc(2)Hex(2) (10.4%), HexNAc(2)Hex(3) (6.6%), and HexNAc(2)Hex(3)Fuc(1) (5.7%) (Fig. 5C and Supp Table 4 [online only]). Since the N-glycan structures in insect glycoproteins are Man_n_(Fuc)GlcNAc_2_ linked to Asn residues (Marchal et al. 2001, Rendic et al. 2008, Chung et al. 2017), the above four N-glycan structures were actually Man_2_FucGlcNAc_2_ (67.9%), Man_2_GlcNAc_2_ (10.4%), Man_3_GlcNAc_2_ (6.6%), and Man_3_FucGlcNAc_2_ (5.7%), which is consistent with previously reported paucimannose structure in the insects (Yehuda and Padler-Karavani 2020).

### N-linked Glycosylation of Storage Protein 1 and Transferrin

Among Con A lectin-enriched glycoproteins, storage protein 1 (Genebank ID: gi|1335609), imaginal disk growth factor (Genebank ID: gi|152061158), and transferrin (SilkDB ID: BGIBMGA011424) had the highest abundance (Fig. 2D). Thus, we investigated the N-linked glycosylation of these three proteins and found 0, 1, and 2 N-Linked glycosylation sites on imaginal disk growth factor, transferrin, and storage protein 1, respectively (Supp Table 4 [online only]).

**Fig. 6.**
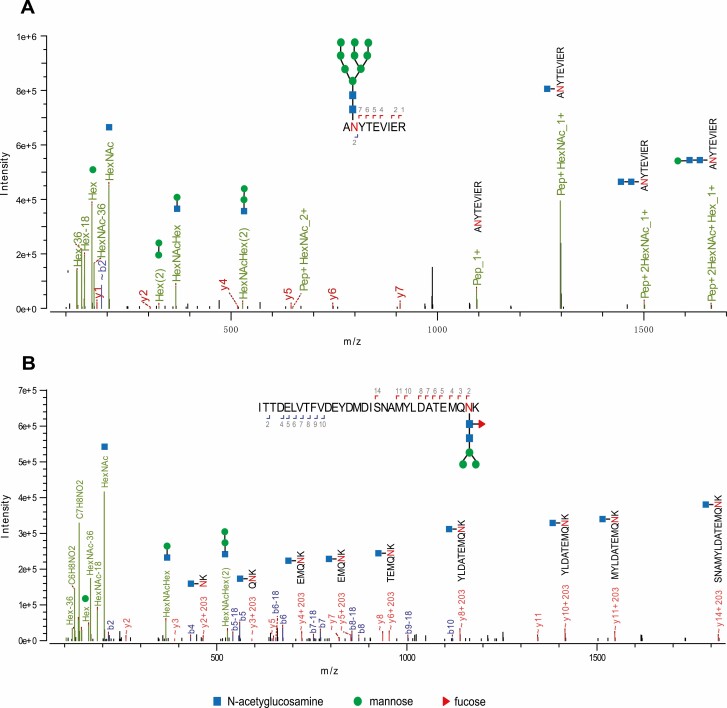
MS/MS spectrum used for identifying N-linked glycopeptides. (A) A MS/MS spectrum of m/z 1864.634^3+^ ion identifying N318 glycopeptides as the Man_9_GlcNAc_2_ glycoform in transferrin. (B) MS/MS spectrum of m/z 1038.375^4+^ ion identifying the N494 glycopeptides as the Man_3_GlcNAc_2_Fuc_1_ glycoform in storage protein 1.

Collision-induced fragmentation of the doubly- and triply-charged ions revealed the presence of five different high-mannose type N-glycans (Man_5-9_GlcNAc_2_) on the N318 residue in tryptic peptide 317-ANYTEVIER-325 of transferrin. Fig. 6A shows an example of an MS/MS spectrum for the triply-charged precursor (*m*/*z* 1864.634), which was identified to be the Man_9_GlcNAc_2_ glycoform at the N318 site. The MS/MS spectrum provided direct evidence for the high mannose-type glycosylation linked to the N318 site. A summary of all N-linked sites and the heterogeneous glycoforms for transferrin identified in this study is provided in Supp Table 4.

For storage protein 1, eight different glycoforms (Man_0-4_GlcNAc_1-2_Fuc_0-1_) were identified on the N494 residue of the tryptic peptide 465-ITTDELVTFVDEYDMDISNAMYLDATEMQNK-495. Fig. 6B shows an example of an MS/MS spectrum for the quadruply-charged precursor (*m*/*z* 1038.375), which was identified to be the Man_3_GlcNAc_2_Fuc_1_ glycoform at the N494 site. A summary of all N-linked sites and heterogeneous glycoforms identified for storage protein 1 in this study is given in Supp Table 4 [online only].

### Interaction Network of N-linked Glycoproteins

**Fig. 7.**
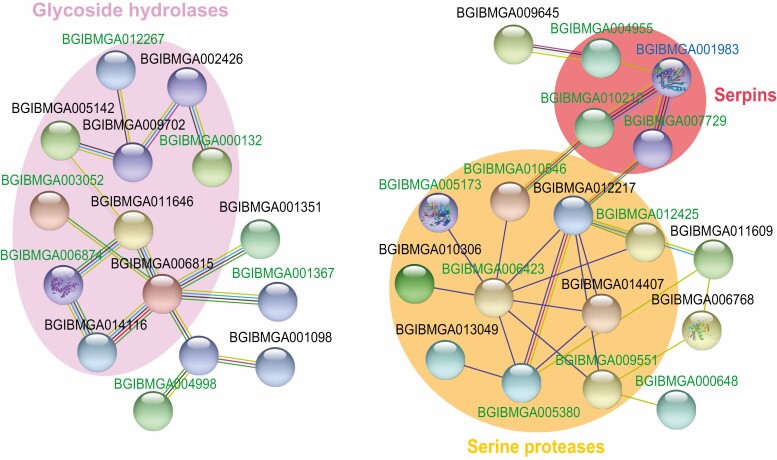
Interaction network model of N-Linked glycoproteins from silkworm serum. The 139 ConA lectin-binding proteins and 82 glycoproteins were used to construct the interaction networks by using the website STRING. According to the annotated domains, three groups of proteins were observed obvious, including glycoside hydrolases (pink), serpins (red), and serine proteases (orange). The lectin-binding proteins are represented by green, the N-linked glycoproteins are indicated with blue, while the lectin-binding N-glycoproteins are shown in black.

The 139 ConA lectin-binding proteins and 82 N-linked glycoproteins were used to construct a protein interaction network model by using the website STRING. The model indicated that 10 glycoside hydrolases formed an interaction network, while four serpins interacted with 10 serine proteases from another network (Fig. 7). Among them, six glycoside hydrolases and four serine proteases were not only significantly enriched by ConA lectin, but also proved as glycopeptide-containing proteins, whereas four glycoside hydrolases, three serpins, and six serine proteases were ConA lectin-enriched proteins but lack of glycopeptide evidence. There were six CLIP-domain serine proteases in the network, including BGIBMGA005173 (CLIP 2), BGIBMGA010546 (CLIP 8), BGIBMGA010306 (CLIP 7/12), BGIBMGA009551 (CLIP 17), BGIBMGA005380 (CLIP 25), BGIBMGA014407 (CLIP 26), which may help to amplify the initial immune recognition signal, and are negatively regulated by serpins (Christophides et al. 2002).

## Discussion

Currently, many researches on insect glycosylation focused on the foreign recombinant glycoproteins expressed in insect cells (Marchal et al. 2001, Rendic et al. 2008, Chung et al. 2017). The data about the glycosylation of the insects’ own proteins was mainly obtained from studies with fruit fly, *D. melanogaster* (Vandenborre et al. 2010, Baycin-Hizal et al. 2011). In this study, we collected silkworm serum proteins, enriched the N-linked glycoproteins by Con A lectin chromatography, and identified them with LC-MS/MS. The findings of this work provided details on the characterization of amino acid site occupancy and glycan composition in silkworm serum glycoproteins.

Using periodic acid-Schiff staining, nearly all the high-abundance proteins in silkworm serum, such as apolipophorin-I, vitellogenin, storage proteins, 30K proteins, serpins, and inter-α-trypsin inhibitor, were found to be glycoproteins. Previous studies suggested that apolipophorin-I, vitellogenin, storage proteins, and 30K proteins play roles in nutrient storage (Zhang et al. 2014b), including providing sources of amino acids and lipid transportation, whereas serpins and the inter-α-trypsin inhibitor act as protease inhibitors to maintain a stable body fluid environment (Zhao et al. 2012).

Using the Con A lectin affinity column to enrich N-linked glycoproteins, we found that storage protein 1, transferrin, and imaginal disk growth factor were significantly enriched by Con A lectin, whereas storage protein 2 and 30K proteins LP1 and LP2 could not be enriched. 30K proteins have a sugar-binding CTD domain and were reported to bind glucans (Ujita et al. 2002, Ujita et al. 2005, Yang et al. 2011). 30K proteins are sugar-binding proteins and may not be N-linked glycoproteins, and thus could not be enriched by Con A lectin. Storage proteins are also called arylphorins, used primarily as a source of aromatic amino acids for protein synthesis during metamorphosis. In the tobacco worm, the storage protein arylphorin is a hexameric glycoprotein and contains two subunits, A1 and A2. Both subunits are glycosylated and have molecular weights of 77 kDa and 72 kDa, respectively. NMR spectroscopy of arylphorin glycopeptides revealed a Man_9_GlcNAc_2_ oligosaccharide structure, similar to that observed in mammalian glycoproteins (Ryan et al. 1985). Consistent with results from tobacco worms, this study identified eight different glycoforms (Man_0-4_GlcNAc_1-2_Fuc_0-1_) in silkworm storage protein 1. By comparing human and silkworm lectin-enriched glycoproteins, we found that glycosylation was prevalent in high-abundance serum proteins, including apolipoproteins, transferrins, inter-α-trypsin inhibitors, and serpins (Yang and Hancock 2004, Madera et al. 2007). N-linked glycosylation may protect secreted proteins in serum against proteolytic degradation, aggregation, and thermal denaturation through the maintenance of optimal conformations (Zhou and Qiu 2019).

By GO analysis, we found that many N-Linked glycoproteins were related to hydrolase activity, enzyme inhibitor activity, metabolic processes, and cell adhesion, which is highly consistent with the results of comparative glycoproteomics among five insect species (Vandenborre et al. 2011). Interestingly, more glycosylated hydrolases were identified in the silkworm than in other insects (Vandenborre et al. 2011), and more glycosylated hydrolase were found in the pupae than in the larvae. Furthermore, the metabolic processes are found to be mainly related to the carbohydrates and proteins, whereas the substrates of hydrolases are also carbohydrates and proteins.

By interaction network analysis, we found that these protein hydrolases in the silkworm serum are serine proteases, most of which have CLIP domains. CLIP-domain serine proteases rapidly amplify the immune signal after pathogens are recognized, and finally lead to the formation of melanin and production of antimicrobial peptides (Zhang et al. 2014b, Chen et al. 2019). By using Edman degradation, two N-linked glycosylation sites were observed in a silkworm CLIP serine protease, the prophenoloxidase-activating enzyme (PPAE) (Satoh et al. 1999). Serpins regulate immune response via inhibiting CLIP-domain serine proteases, and were enriched by ConA lectin in this study. The glycosylation of immune-related proteins may enhance protein stabilization, mediate interaction with pathogens, and modulate immune responses (Dwek 1996, Spiro 2002, Lis and Sharon 2005). More immune-related glycoproteins were found in the pupal hemolymph than in the larval hemolymph, may reflecting that pupae need a more stable and efficient immune response system than larvae.

Among glycoside hydrolases, three of which are α-mannosidases, including BGIBMGA002426, BGIBMGA005142, and BGIBMGA012267. These mannosidases showed higher abundance in the pupal stage than in the larval stage, and may be involved in trimming of high-mannose N-glycans to form paucimannose N-glycans (Walski et al. 2016). In *T. castaneum*, RNAi of α-mannosidases caused pupa malformation and prevented adult eclosion, and thus demonstrated that α-mannosidases involved in the N-glycan processing are crucial for insect metamorphosis (Walski et al. 2016).

### Conclusions

In this study, 139 silkworm serum proteins were identified from Con A lectin-enriched fractions, 63 of which were proved as N-linked glycoproteins with at least one glycosylation site. The N-linked glycosylation sites and N-glycan structures were analyzed in detail. The GO analysis and interaction network revealed that N-linked glycosylation is important for glycoside hydrolases, serine proteases, and serpins. This study provides important clues for understanding the roles of N-linked glycosylation in insect immunity and metamorphosis.

## Supplementary Material

ieab057_suppl_Supplementary_Table_S1

ieab057_suppl_Supplementary_Table_S2

ieab057_suppl_Supplementary_Table_S3

ieab057_suppl_Supplementary_Table_S4

ieab057_suppl_Supplementary_Figure_S1
