# Supplementary material for: Identification of N-linked Glycoproteins in Silkworm Serum Using Con A Lectin Affinity Chromatography and Mass Spectrometry
Source: J Insect Sci. 2021 Aug 17;21(4):14. doi: 10.1093/jisesa/ieab057 (PMC8367846; doi:10.1093/jisesa/ieab057)
Supplement: ieab057_suppl_Supplementary_Table_S1 [file ieab057_suppl_supplementary_table_s1.pdf]

Supplementary Table S1. Identified results of proteins digged from polyacrylamide gel. The identification information was listed, including Uniprot ID, Genebank ID, annotated name, peptide number, sequence coverage, molecular weight, posterior error probability (PEP), and iBAQ intensity.

Uniprot IDs	Genebank IDs	Annotated names	Peptides	Unique peptides	Sequence coverage [%]	Mol. weight [kDa]	PEP	iBAQ B1	iBAQ B2	iBAQ B3	iBAQ B4	iBAQ B5	iBAQ B6	iBAQ B7	iBAQ B8	iBAQ B9
tr|G1UIS8|	gi|1200716789|	Apolipophorin protein	226	11	62.5	369.19	0	3E+09	1E+07	1E+06	4E+07	128700	0	0	0	0
sp|Q27309|	-	Vitellogenin	79	79	54	203.05	0	1E+07	1E+09	91135	0	0	0	0	0	0
-	gi|1200719391|	Inter-alpha-trypsin inhibitor heavy chain H4	27	27	27.1	99.103	0	0	0	4E+08	0	1E+06	0	0	0	0
sp|P09179|	-	Sex-specific storage-protein 1	89	89	73.4	87.241	0	386500	0	4E+06	5E+09	3E+06	7E+06	0	3E+06	0
-	gi|1174445|	Sex-specific storage-protein 2	76	76	72.3	83.466	0	0	0	504140	9E+06	1E+10	6E+07	4E+06	0	0
-	gi|1200731398|	Imaginal disk growth factor, chitinase-like protein	21	21	42.2	49.065	0	0	0	0	0	0	3E+09	2E+06	0	393920
sp|Q03383|	-	Serpin9; Antichymotrypsin-1	15	15	37	44.572	0	0	0	0	0	597830	0	3E+09	4E+06	529360
tr|C7ASM9|	-	Serpin1; Antitrypsin isoform 1	26	26	64.3	43.428	0	0	0	0	0	2E+07	0	2E+09	708070	0
sp|P09334|	-	BmLP1; 30 kDa lipoprotein PBMHP-6	26	25	77	29.734	0	3E+06	0	0	0	0	0	0	1E+10	7E+07
tr|H9B444|	-	BmLP2; 30K protein 11	25	13	59.8	30.53	0	703920	662570	645630	0	0	7E+06	0	6E+09	9E+06
sp|Q00801|	-	BmLP4; 30 kDa lipoprotein 21G1	20	2	62.4	30.183	0	762760	0	0	0	0	0	0	5E+09	577730
sp|Q00802|	-	BmLP3; 30 kDa lipoprotein 19G1	26	17	68.4	29.499	0	0	0	0	0	0	694080	0	5E+09	1E+07
tr|H9J4G0|	-	BmLP9	33	10	57.6	43.772	0	0	0	183760	0	0	0	0	4E+09	1E+06
tr|H9JU96|	-	Apolipophorin-III	14	14	48.2	27.586	0	0	0	0	0	0	0	0	0	2E+09
tr|H9B445|	-	BmLP25; 30K protein 12	15	11	31.4	49.77	0	0	0	0	0	0	2E+08	0	0	0
tr|H9B457|	-	BmLP17; 30K protein 24	19	19	68	28.495	0	0	0	0	0	0	0	0	6E+08	0
-	gi|512890995|	BmLP5; 30 kDa lipoprotein 21G1-like	7	3	26.5	30.149	0	0	0	0	0	0	0	0	2E+08	0
tr|H9JUX9|	-	Uncharacterized protein	210	15	71.8	290.28	0	2E+08	1E+06	0	0	0	0	0	0	0
tr|H9JCP0|	-	ommochrome-binding protein-like	8	8	57.3	29.403	0	0	0	0	0	0	0	0	1E+08	0
tr|Q17233|	-	p50 protein	9	9	19.9	52.217	1.25E-108	0	0	0	0	0	8E+07	0	0	0
sp|Q8T113|	-	27 kDa glycoprotein	8	8	41.9	24.902	3.84E-200	0	0	0	0	0	0	0	8E+07	0
tr|H9ITU7|	gi|512921342|	EF-hand calcium-binding domain-containing	2	2	10.8	25.71	0.0019421	0	0	0	8E+07	0	0	0	0	0
tr|G9I6Y1|	-	Arylphorin	65	64	73	82.85	0	0	0	173040	3E+07	4E+07	4E+06	0	0	0
tr|Q8WPH3|	-	Fibrillin-like protein	3	3	7.6	63.114	6.90E-22	0	0	0	8E+07	2E+06	0	0	0	0
tr|O97158|	-	Transferrin	56	56	68.1	75.722	0	0	0	329760	7E+07	650870	774410	0	0	0
tr|H9JIA9|	gi|512891154|	fibrohexamerin	3	3	18.4	26.872	1.06E-22	0	0	0	0	0	0	0	4E+07	0
-	gi|512898603|	glyoxylate reductase/hydroxypyruvate reductase	8	8	30.1	39.996	0	0	0	0	0	0	0	3E+07	0	0
tr|Q9U556|	-	Hemolymph juvenile hormone binding protein	4	4	28.4	26.643	2.91E-29	0	0	0	0	0	0	0	3E+07	0
tr|Q05432|	-	Hemolymph protein	4	4	15.6	29.737	1.66E-24	0	0	0	0	0	0	0	3E+07	0
tr|H9J236|	gi|1200732189|	atlastin	16	16	27.2	85.709	3.11E-147	0	0	0	3E+07	0	0	0	0	0
tr|H9J8N4|	-	Uncharacterized protein	5	5	4.1	143.06	8.19E-11	0	206280	0	0	0	0	0	3E+07	0
tr|H9J4G1|	-	Uncharacterized protein	21	5	57.1	35.308	0	0	0	0	0	0	0	0	3E+07	0
tr|Q8N0P2|	gi|1200724839|	Heat shock protein 70	26	22	37.4	71.175	0	0	0	0	0	3E+07	0	0	0	0
sp|Q27451|	-	Phenoloxidase subunit 1	12	12	22.6	78.784	5.24E-134	0	0	0	0	2E+07	0	0	0	0
tr|H9JU11|	-	Uncharacterized protein	11	11	28	53.429	0	0	0	0	0	0	2E+07	0	0	0
tr|H9J4G4|	-	BmLP14; 30K protein 5	3	2	12.4	29.079	1.80E-44	0	0	0	0	0	0	0	1E+07	0
tr|Q75RW3|	-	BmLSP-T	3	3	15.7	30.888	2.81E-14	0	0	0	0	0	0	0	1E+07	0
-	gi|827537784|	xaa-Pro aminopeptidase ApepP	11	11	25.1	74.888	1.77E-34	0	0	0	0	1E+07	0	0	0	0
tr|H9J5S6|	-	Uncharacterized protein	3	3	19.2	25.009	3.49E-09	0	0	0	0	0	0	0	1E+07	0
tr|H9J4M3|	-	Uncharacterized protein	4	4	20.7	29.799	4.60E-24	0	0	0	0	0	0	0	1E+07	0
tr|H9JJ98|	-	Uncharacterized protein	9	8	8.8	127.74	3.69E-107	0	1E+07	0	0	0	0	0	0	0

tr|H9IW30|	gi|1200731218|	alpha-tocopherol transfer protein	4	4	7.3	61.138	9.67E-08	0	0	0	8E+06	0	0	0	0	0
-	gi|827538326|	BmLP16; 30 kDa lipoprotein 21G1-like	14	3	47.4	32.611	0	0	0	0	0	0	0	0	7E+06	0
tr|H9B448|	-	BmLP13; 30K protein 15	2	2	8.2	29.858	8.99E-19	0	0	0	0	0	0	0	7E+06	0
tr|H9JTA2|	gi|1200729338|	phenoloxidase subunit 1-like	13	13	20.4	79.237	5.93E-38	0	0	0	0	6E+06	0	0	0	0
tr|Q8I924|	-	Prophenoloxidase activating factor 3	3	3	13.2	42.646	1.05E-13	0	0	0	0	6E+06	0	0	0	0
tr|Q1HPN3|	-	Putative hydroxypyruvate isomerase	4	4	23.5	29.208	0	0	0	0	0	0	0	0	5E+06	0
tr|Q2F5Z9|	-	Alpha-N-acetylgalactosaminidase	3	3	7.9	49.008	2.61E-06	0	0	0	0	0	4E+06	0	0	0
tr|H9J261|	-	Uncharacterized protein	5	5	8	86.483	8.72E-27	0	0	0	3E+06	0	0	0	0	0
tr|H9JJA9|	-	Uncharacterized protein	7	7	5.3	181.32	1.16E-21	0	0	0	0	0	443740	2E+06	0	0
sp|Q27452|	-	Phenoloxidase subunit 2	11	11	20.2	80.118	4.85E-70	0	0	0	1E+06	1E+06	0	0	0	0
tr|Q5CCL2|	-	Peptidylprolyl isomerase	2	2	4.2	51.019	2.37E-16	0	0	0	0	0	2E+06	0	0	0
-	gi|512928928|	centromere-associated protein E	2	2	3.8	62.896	5.00E-19	0	0	0	0	2E+06	0	0	0	0
tr|B7XFU6|	-	Clathrin heavy chain	18	18	15	191.99	1.17E-57	0	0	0	0	0	2E+06	0	0	0
tr|A4PHN7|	-	Beta-hexosaminidase	5	5	11.8	61.694	4.91E-148	0	0	0	0	951420	0	0	0	0
tr|H9JFB7|	-	Uncharacterized protein	2	2	6.1	48.376	1.46E-05	0	0	0	0	0	940980	0	0	0
tr|H9JMY9|	gi|827542143|	zonadhesin	7	7	13.8	68.216	2.01E-29	0	0	0	0	0	0	896850	0	0
tr|H9J6I9|	gi|1200712789|	uncharacterized protein LOC101741510	3	3	2.2	228.05	5.95E-71	0	0	0	0	844070	0	0	0	0
tr|H9JPS0|	gi|1200738389|	Uncharacterized protein	6	6	13.9	113.72	0	0	0	0	0	0	0	781310	0	0
sp|P29520|	-	Elongation factor 1-alpha	21	21	50.5	50.371	0	0	0	0	0	0	672280	0	0	0
-	gi|827558163|	serine protease inhibitor 3	2	2	2.5	97.024	0.0003077	0	0	0	0	0	661090	0	0	0
-	gi|1200740129|	cartilage oligomeric matrix protein	4	4	3.7	125.65	3.37E-07	0	0	629850	0	0	0	0	0	0
tr|Q6Q2D6|	-	Serpin-4A	3	3	7.3	46.3	2.71E-17	0	0	0	0	0	544460	0	0	0
tr|H9JHU2|	-	Uncharacterized protein	2	2	4.6	49.447	0.0003892	0	0	0	0	458080	0	0	0	0
tr|B5BSX5|	gi|1200735149|	Paralytic peptide binding protein 2	7	5	21.4	48.471	6.52E-44	0	0	0	0	0	331520	0	0	0
tr|S0BCV4|	-	Apolipoprotein of lipid transfer particle-I/II	3	3	0.9	470.18	6.13E-16	0	0	0	315370	0	0	0	0	0
tr|H9IS30|	gi|1200722898|	cytochrome P450 4V2	2	2	5.4	55.207	0.0033619	0	0	315110	0	0	0	0	0	0
tr|H9IUI0|	-	Uncharacterized protein	2	2	1.2	199.48	0.0002392	217780	0	0	0	0	0	0	0	0
tr|H9JEU1|	-	Peptidoglycan-recognition protein GN=PGRP-S1	2	2	9.2	24.205	0.0006665	0	0	0	0	0	0	0	0	8E+06
tr|Q1HPI5|	-	Nucleoside diphosphate kinase	3	3	27.3	17.313	0	0	0	0	0	0	0	0	0	1E+07
