# Supplementary material for: Identification of N-linked Glycoproteins in Silkworm Serum Using Con A Lectin Affinity Chromatography and Mass Spectrometry
Source: J Insect Sci. 2021 Aug 17;21(4):14. doi: 10.1093/jisesa/ieab057 (PMC8367846; doi:10.1093/jisesa/ieab057)
Supplement: ieab057_suppl_Supplementary_Table_S2 [file ieab057_suppl_supplementary_table_s2.pdf]

Supplementary Table S2. List of total serum proteins and conA lectin-enriched proteins. The proteins were listed by silkDB ID, Genebank ID, peptide number, molecular weight, score, and iBAQ intensity. The proteins enriched significantly by con A lectin were screened by ratio value > 2 and p value < 0.05. V7 T, total serum proteins from day 7 of the larval fifth instar; P2 T, total serum proteins from day 2 of the pupae; V7 L, lectin-enriched serum proteins from day 7 of the larval fifth instar; P2 L, lectin-enriched serum proteins from day 2 of the pupae.

No.	Classification	Annotated name	Genebank IDs	silkDB IDs	Peptides	Unique peptides	Mol. weight [kDa]	Score	iBAQ of V7 T	iBAQ of V7 L	iBAQ of P2 T	iBAQ of P2 L	Ratio of iBAQ (V7 L/V7 T)	P value (V7 L/V7 T)	Ratio iBAQ of (P2 L/P2 T)	P value (P2 L/P2 T)	Con A Lectin-binding
54	binding and transport	30K protein BmLP2	gi|379046488|gb|AFC8780	BGIBMGA004399-PA	36	5	29.251	323.31	2.3E+10	1.2E+09	2.6E+10	1.3E+09	0.0506354	4.7E-06	0.052088	1.6E-07	
61	binding and transport	30K protein BmLP1	gi|379046494|gb|AFC8780	BGIBMGA004394-PA	22	20	29.734	323.31	1.6E+10	1.6E+07	2E+10	2.7E+08	0.00095	5.8E-07	0.013071	2E-06	
41	binding and transport	arylphorin/storage protein 1	gi|1335609|emb|CAA3141	—	102	2	85.757	323.31	1.4E+10	6.7E+10	8.3E+08	6.8E+09	4.7888784	8.8E-06	8.155471	0.0039	Yes
39	binding and transport	arylphorin/storage protein 2	gi|95103012|gb|ABF51447	BGIBMGA009028-PA	80	80	83.451	323.31	9E+09	3.6E+09	2.2E+07	8.5E+07	0.3963772	0.0013	3.855053	0.11099	
51	binding and transport	30K protein BmLP4	gi|379046486|gb|AFC8780	BGIBMGA004395-PA	33	19	30.242	323.31	4.6E+09	2.3E+08	6.6E+09	4.2E+08	0.0500249	1.2E-06	0.063709	8.4E-06	
46	binding and transport	30K protein BmLP9	gi|512890676|ref|XP_0049	BGIBMGA004400-PA	26	8	29.51	323.31	4.4E+09	9658048	5.2E+09	5.7E+07	0.0022121	1.1E-06	0.011099	2.9E-09	
290	immune-signaling	serpin-9	gi|14028769|gb|AAK5249	BGIBMGA001983-PA	38	38	41.918	323.31	3.3E+09	1.3E+08	1.4E+09	5.7E+08	0.0398478	2.8E-06	0.398834	0.01378	
265	immune-non-serpin type	serine protease inhibitor amfpi	gi|51594279|gb|AAU0817	BGIBMGA008205-PA	4	4	9.6615	108.18	3E+09	0	3.2E+09	6.8E+07	0	0.00013	0.020975	0.00038	
37	binding and transport	Chemosensory protein 7	gi|77415578|emb|CAJ0145	BGIBMGA004041-PA	11	11	13.519	303.04	2.5E+09	0	2.4E+09	4206617	0	1E-07	0.001773	3.7E-05	
56	binding and transport	30K protein BmLP17	gi|525343846|ref|NP_0012	BGIBMGA004398-PA	24	24	28.495	323.31	1.4E+09	9.4E+07	2E+09	1.6E+08	0.0686633	6.3E-05	0.082141	6.2E-06	
62	binding and transport	27 kDa glycoprotein	gi|74843747|sp|Q8T113.1|	BGIBMGA000462-PA	15	15	24.902	323.31	1.4E+09	2.6E+08	1.7E+09	2.8E+09	0.1930425	0.00012	1.618501	0.01407	
228	other	Imaginal disk growth factor	gi|152061158|dbj|BAF736	BGIBMGA000648-PA	35	35	48.163	323.31	1.3E+09	8.4E+09	1.7E+09	1.7E+10	6.5959913	0.00055	9.724561	0.00404	Yes
50	binding and transport	30K protein BmLP5	gi|512890995|ref|XP_0049	BGIBMGA004456-PA	13	6	29.247	126.64	1.1E+09	1.7E+07	1.8E+09	4.6E+07	0.0160334	2.1E-05	0.026275	1.4E-05	
254	immune-non-serpin type	serine protease inhibitor TIL	—	BGIBMGA006239-PA	10	9	9.9994	207.07	1E+09	6180207	3.5E+09	2.4E+07	0.0059017	0.00309	0.006773	4.1E-06	
266	immune-non-serpin type	Chymotrypsin inhibitorSCI-II	gi|124924|sp|P10832.1|ISC	—	7	7	7.067	81.214	1E+09	0	1.2E+09	0	0	2.9E-05	0	0.00111	
38	binding and transport	arylphorin/storage protein 5	gi|512929380|ref|XP_0049	BGIBMGA008859-PA	64	63	87.399	323.31	8.3E+08	4.1E+08	9.8E+07	3.7E+08	0.4911954	0.00441	3.817889	0.10907	
324	immune-recognition	immunoglobulin-like	gi|33285891|gb|AAQ0156	BGIBMGA008221-PA	9	9	17.123	247.54	7.3E+08	0	7.9E+08	0	0	1.6E-06	0	5.7E-06	
7	binding and transport	Transferrin GN=Tf	—	BGIBMGA011424-PA	76	6	71.651	323.31	6.7E+08	2.5E+09	1.7E+09	1.4E+10	3.6900004	0.00068	8.510778	0.00326	Yes
218	extracellular matrix	cuticular protein glycine-rich 16	gi|267844873|ref|NP_0011	BGIBMGA002175-PA	4	4	21.628	178.13	6.1E+08	5.5E+07	4.2E+07	0	0.0902619	0.00146	0	0.00564	

43	binding and transport	apolipophorin-III	gi|1381798|gb|AAB02853.	BGIBMGA013108-PA	34	12	74.719	323.31	5.7E+08	3.9E+07	6.6E+08	4E+08	0.0681277	3.2E-05	0.604713	0.05663	
49	binding and transport	30K protein BmLP6	gi|5896|emb|CAA30437.1|	BGIBMGA004457-PA	30	5	30.343	73.507	5.5E+08	0	4.9E+08	1E+07	0	4.6E-05	0.02046	4E-07	
105	enzyme	placental protein 11	gi|512922547|ref|XP_0049	BGIBMGA003688-PA	20	20	39.626	323.31	4.6E+08	3934743	3.8E+08	2.3E+07	0.0085711	0.00059	0.059804	0.00094	
26	binding and transport	apolipophorin	gi|512908283|ref|XP_0049	BGIBMGA013342-PA	269	2	368.77	323.31	4.5E+08	2.5E+08	1.8E+09	2.1E+09	0.5530027	0.01312	1.167142	0.77492	
366	unknown protein	Uncharacterized protein	gi|512937718|ref|XP_0049	BGIBMGA014203-PA	16	16	36.643	323.31	4.4E+08	2.3E+09	1.5E+08	2.4E+09	5.2955322	0.00063	15.66278	0.00016	Yes
263	immune-non-serpin type	serine protease inhibitor ITI	gi|512899354|ref|XP_0049	BGIBMGA007558-PA	48	48	99.103	323.31	4.1E+08	8.3E+08	2.5E+08	1.3E+09	2.0256036	0.00304	5.056626	0.00045	Yes
237	immune-effector	prophenoloxidase PPO2	gi|994751|dbj|BAA08369.	BGIBMGA013115-PA	44	43	80.118	323.31	3.6E+08	316711	5.6E+08	801216	0.0008827	3.9E-08	0.001441	3.5E-06	
18	binding and transport	ommochrome binding protein 2	gi|512905108|ref|XP_0049	BGIBMGA007286-PA	14	14	29.403	323.31	3.2E+08	0	1.3E+09	1.2E+07	0	2.3E-05	0.00933	1.1E-07	
48	binding and transport	30K protein BmLP7	gi|1587670|prf||2207200A	BGIBMGA004397-PA	76	3	2691	323.31	3.1E+08	1.2E+07	4.4E+08	7.8E+07	0.0374262	2.5E-07	0.177519	2.2E-06	
252	immune-non-serpin type	serine protease inhibitor TIL	gi|512898433|ref|XP_0049	BGIBMGA009094-PA	3	3	8.765	27.705	2.9E+08	0	8.7E+07	0	0	0.00316	0	0.35592	
93	enzyme	seminal fluid protein	gi|512908815|ref|XP_0049	BGIBMGA001351-PA	21	21	38.555	323.31	2.8E+08	1.7E+09	3E+08	4.3E+09	6.1373209	0.00033	14.44873	0.0078	Yes
14	binding and transport	pheromone binding protein /	gi|512924921|ref|XP_0049	BGIBMGA011276-PA	9	9	20.55	266.31	2.4E+08	0	3.5E+08	0	0	6.7E-05	0	4.5E-07	
262	immune-non-serpin type	serine protease inhibitor kazal	gi|87248517|gb|ABD3631	BGIBMGA011573-PA	5	5	10.582	79.771	2.4E+08	0	2.1E+08	0	0	0.0036	0	0.00058	
332	other	fibroin p25 like	gi|512891154|ref|XP_0049	BGIBMGA009259-PA	6	6	26.872	272.04	2.2E+08	3.3E+08	8.5E+07	7.6E+08	1.5094931	0.38036	8.972668	0.08528	
333	other	fatbody protein 3rev-g1, MBF2	gi|512894503|ref|XP_0049	BGIBMGA013781-PA	6	6	12.834	119.63	1.9E+08	0	1.5E+08	0	0	2.2E-06	0	0.00033	
44	binding and transport	Antennal binding protein	gi|87248601|gb|ABD3635	BGIBMGA002626-PA	8	8	15.063	180.8	1.7E+08	0	5.9E+08	2E+07	0	2.8E-07	0.033247	2.9E-05	
238	immune-effector	prophenoloxidase PPO1	gi|994749|dbj|BAA08368.	BGIBMGA012763-PA	40	8	78.72	323.31	1.5E+08	0	2.5E+08	2188687	0	1.1E-05	0.008784	2.2E-05	
13	binding and transport	pheromone binding protein /	gi|512933090|ref|XP_0049	BGIBMGA011432-PA	8	8	14.865	168.75	1.5E+08	0	4.7E+08	5.4E+07	0	0.00081	0.113401	0.00543	
314	immune-signaling	ENF peptides binding protein	gi|525342911|ref|NP_0012	BGIBMGA010876-PA	28	6	49.77	323.31	1.4E+08	0	2.6E+08	3671537	0	3.7E-07	0.014082	6.9E-06	
95	enzyme	receptor tyrosine phosphatase type	gi|512933991|ref|XP_0049	BGIBMGA012106-PA	9	9	22.753	88.623	1.4E+08	2.3E+07	8.3E+07	1.8E+08	0.1696499	8E-05	2.147398	0.08005	
284	immune-recognition	C-type lectin 20	gi|512888320|ref|XP_0049	BGIBMGA002288-PA	13	6	35.143	318.52	1.2E+08	1.8E+08	5.7E+07	1.8E+08	1.5109682	0.15591	3.227074	0.15216	
287	immune-recognition	C-type lectin 10	gi|148298818|ref|NP_0010	BGIBMGA006768-PA	16	16	36.562	323.31	1.1E+08	6.9E+08	1.2E+08	2.5E+09	5.999891	2.4E-06	20.75449	0.02323	Yes
53	binding and transport	30K protein BmLP20	gi|379046490|gb|AFC8780	BGIBMGA004464-PA	13	13	29.726	274.83	1.1E+08	0	1E+08	0	0	7E-09	0	3.3E-06	
57	binding and transport	30K protein BmLP15	gi|512890626|ref|XP_0049	BGIBMGA004403-PA	22	12	30.086	151.09	1.1E+08	5.3E+07	9758566	966645	0.4930148	0.00204	0.099056	0.00113	
269	immune-non-serpin type	BCP inhibitor GN=bcpi	gi|4753670|emb|CAB4193	BGIBMGA007039-PA	7	7	10.79	159.04	1E+08	0	7.9E+08	4759308	0	7.7E-06	0.006032	5.5E-05	

175	enzyme	Beta-hexosaminidase	—	BGIBMGA014116-PA	41	4	61.688	323.31	9.9E+07	2.9E+09	1.9E+08	5.3E+09	28.930349	0.00011	27.83763	0.00635	Yes
129	enzyme	Hydroxypyruvate isomerase	gi|164459610|gb|ABY579	BGIBMGA002349-PA	18	18	29.169	136.97	9.8E+07	0	1.3E+08	0	0	2.7E-05	0	7E-07	
68	other	gelsolin	—	BGIBMGA001755-PA	18	18	39.376	189.98	9.3E+07	0	6.9E+07	155309	0	7.1E-05	0.002257	8.8E-05	
132	enzyme	GTPase	gi|512922101|ref|XP_0049	BGIBMGA003573-PA	37	37	85.709	323.31	9.1E+07	0	2.2E+07	0	0	1.3E-06	0	0.00045	
362	unknown protein	uncharacterized protein	gi|512903120|ref|XP_0049	—	7	7	12.313	108.4	9E+07	0	1.1E+08	0	0	0.00033	0	0.00289	
249	immune-effector	antibacterial peptide	gi|82949288|dbj|BAE5337	—	4	4	7.2564	25.885	8.7E+07	0	1.9E+08	0	0	0.00896	0	3.5E-05	
317	immune-signaling	ENF peptides binding protein	gi|379046502|gb|AFC8781	BGIBMGA008164-PA	25	12	49.352	198.21	8.6E+07	0	1.7E+08	0	0	0.00047	0	2.4E-06	
90	enzyme	serine protease BmSP139	gi|95102830|gb|ABF5135€	BGIBMGA006423-PA	12	12	30.826	323.31	8.2E+07	1.4E+08	7.1E+07	3.8E+08	1.6692128	0.03177	5.301811	0.0006	Yes
318	immune-signaling	ENF peptide	gi|307746851|dbj|BAJ212	BGIBMGA014551-PA	3	3	14.275	22.353	7E+07	0	6.4E+07	0	0	0.00076	0	0.00031	
267	immune-non-serpin type	Chymotrypsin inhibitor SCI-III	gi|124145|sp|P07481.1|ISC	BGIBMGA013945-PA	10	4	103.45	209.86	6.8E+07	0	1.1E+08	0	0	2.3E-05	0	7.5E-05	
24	binding and transport	Lipocalin	gi|512894212|ref|XP_0049	BGIBMGA004866-PA	8	8	25.009	159.68	6.4E+07	5.8E+07	7.8E+07	3.1E+08	0.9127202	0.85831	3.981374	0.11468	
66	other	myosin heavy chain	gi|512932812|ref|XP_0049	BGIBMGA011525-PA	31	31	113.72	323.31	6.2E+07	8852646	1.7E+08	1.3E+07	0.1437075	0.00059	0.08078	2.6E-06	
248	immune-effector	Attacin 1	gi|957281|gb|AAB33990.1	BGIBMGA002747-PA	17	3	96.923	180.7	6.1E+07	8055580	3.7E+07	3954740	0.1328912	0.00035	0.10765	0.00274	
315	immune-signaling	ENF peptides binding protein	gi|83416494|gb|ABC1826	BGIBMGA010168-PA	23	9	50.013	166.32	5.8E+07	0	8.8E+07	3461389	0	2.7E-05	0.039458	0.00027	
289	immune-recognition	beta-1,3-glucan recognition	gi|7007410|dbj|BAA90831	BGIBMGA011608-PA	18	3	58.465	152.13	5.8E+07	0	5.5E+07	5117255	0	5.9E-06	0.093724	0.00076	
240	immune-effector	prophenoloxidase	—	BGIBMGA012764-PA	34	25	73.582	323.31	5.7E+07	0	8.9E+07	0	0	6.1E-06	0	9.3E-08	
81	enzyme	Superoxide dismutase [Cu-	gi|68144076|gb|AAY8607	BGIBMGA002907-PA	10	4	18.149	173.78	5.7E+07	2.6E+08	1.4E+08	2.4E+09	4.5659527	0.00171	17.01354	0.02527	Yes
375	other	proline-rich extensin-like	gi|512922539|ref|XP_0049	BGIBMGA003508-PA	2	2	15.241	20.815	5.6E+07	0	1.9E+07	0	0	0.00685	0	0.02123	
356	unknown protein	uncharacterized protein	gi|512903687|ref|XP_0049	—	5	5	15.477	96.775	5.3E+07	0	5.4E+07	0	0	0.00701	0	0.01725	
268	immune-non-serpin type	carboxypeptidase inhibitor	gi|95102736|gb|ABF5130	—	2	2	12.561	90.796	5.1E+07	4.1E+07	1E+08	1.1E+08	0.8019713	0.7009	1.102602	0.81267	
184	enzyme	antennal esterase cxe11	gi|87248285|gb|ABD3619	BGIBMGA012031-PA	34	34	61.415	323.31	5E+07	7E+08	7E+07	2.6E+09	14.133794	0.00041	36.94645	0.01122	Yes
272	immune-recognition	Peptidoglycan-recognition	gi|4666254|dbj|BAA7721	BGIBMGA008038-PA	10	10	21.626	164.45	4.9E+07	0	1.5E+08	0	0	0.00149	0	2.5E-05	
224	extracellular matrix	adhesion related transmembrane	gi|512895721|ref|XP_0049	BGIBMGA008135-PA	22	22	31.639	323.31	4.6E+07	4.9E+08	8.2E+07	2.2E+09	10.598118	0.01134	26.72551	0.00179	Yes
20	binding and transport	odorant binding protein	gi|226000891|dbj|BAH367	BGIBMGA008354-PA	12	12	15.962	118.87	4.4E+07	0	2.2E+08	1715011	0	0.00055	0.00793	0.00075	
158	enzyme	cysteine-type endopeptidase	gi|13548667|dbj|BAB4080	BGIBMGA007061-PA	16	16	37.56	272.37	3.9E+07	2.2E+08	1.2E+07	1E+08	5.6788681	0.05886	9.019082	0.00116	Yes

215	extracellular matrix	fibrillin-like	gi|17298113|dbj|BAB7852	—	6	6	63.114	323.31	3.9E+07	2.6E+08	6.1E+07	9.4E+08	6.6891873	0.00215	15.49394	0.02387	Yes
16	binding and transport	ommochrome binding protein 2	gi|512905104|ref|XP_0049	BGIBMGA007285-PA	8	8	29.387	158.11	3.7E+07	1.6E+07	6.7E+07	2.1E+08	0.424603	0.0803	3.153042	0.00858	Yes
234	other	Heat shock protein 25.4	gi|169646838|ref|NP_0011	BGIBMGA005781-PA	7	7	25.392	111.38	3.3E+07	3922531	1.4E+08	3222429	0.1179936	0.00034	0.02322	8.7E-07	
59	binding and transport	30K protein BmLP13	gi|530234120|ref|NP_0012	BGIBMGA004454-PA	13	13	27.823	113.73	3.3E+07	0	4.8E+07	0	0	3.7E-06	0	2.2E-05	
380	unknown protein	Uncharacterized protein	—	BGIBMGA003276-PA	4	4	12.733	56.356	3.3E+07	2.3E+08	9686436	6.1E+07	6.889438	0.08666	6.321623	0.0108	Yes
111	enzyme	Nucleoside diphosphate	gi|95103130|gb|ABF51506	BGIBMGA007701-PA	8	8	17.182	47.346	3.3E+07	0	4.5E+07	0	0	3.2E-06	0	4.4E-05	
280	immune-recognition	hemocytin, humoral lectin	—	BGIBMGA006693-PA	28	25	125.78	278.6	3.1E+07	0	6289652	394753	0	0.00059	0.062762	0.00025	
8	binding and transport	transferrin	gi|87246220|gb|ABD3528	—	73	3	75.722	27.024	3.1E+07	1.5E+08	1E+08	8.5E+08	4.8038161	0.02779	8.221136	0.00075	Yes
278	immune-recognition	Hemocytin, humoral lectin	gi|664884|dbj|BAA06160.	BGIBMGA006691-PA	71	36	343.35	323.31	3.1E+07	0	8912517	494926	0	4E-06	0.055532	0.0001	
199	enzyme	aldo-keto reductase	gi|512908854|ref|XP_0049	BGIBMGA001348-PA	14	14	36.93	232.8	3.1E+07	0	5E+07	0	0	0.00065	0	0.0003	
304	immune-signaling	serine protease CLIP2	gi|84778397|dbj|BAE7325	BGIBMGA005173-PA	11	11	40.744	93.446	2.8E+07	2.3E+07	2.3E+07	4.8E+07	0.8137753	0.3687	2.09339	0.04839	Yes
305	immune-signaling	serine protease CLIP11	gi|198041267|dbj|BAG704	BGIBMGA014404-PA	31	31	106.18	323.31	2.8E+07	1.3E+07	6.4E+07	1.1E+08	0.4788141	0.06805	1.786517	0.01376	
144	enzyme	gamma-interferon-	gi|87248349|gb|ABD3622	BGIBMGA009614-PA	13	13	27.395	192.69	2.8E+07	0	1.7E+08	4.6E+07	0	0.00156	0.277902	0.00164	
60	binding and transport	30K protein BmLP10	gi|379046508|gb|AFC8781	BGIBMGA004455-PA	11	6	29.191	15.94	2.5E+07	5790381	4622338	0	0.2355059	0.06076	0	0.17094	
58	binding and transport	30K protein BmLP14	gi|525343768|ref|NP_0012	BGIBMGA004404-PA	11	10	29.079	148.64	2.4E+07	0	1.7E+08	0	0	1.2E-05	0	3.3E-06	
159	enzyme	cysteine-type endopeptidase	gi|512917642|ref|XP_0049	BGIBMGA013023-PA	16	16	53.429	119.39	2.2E+07	0	3.3E+07	356244	0	0.00015	0.010774	4.3E-05	
29	binding and transport	insecticyanin-A-like; Lipocalin	gi|512894208|ref|XP_0049	BGIBMGA004805-PA	10	10	24.176	256.29	2.2E+07	5.2E+07	7.1E+07	4.1E+08	2.3767856	0.07592	5.687803	0.00048	Yes
148	enzyme	Fibroinase GN=Bcp	gi|164420679|ref|NP_0010	BGIBMGA011342-PA	9	3	38.138	107.31	2.1E+07	4832681	976172	4843882	0.226024	0.02053	4.962118	0.25925	
150	enzyme	ester hydrolase c11orf54 homolog	gi|512885211|ref|XP_0049	BGIBMGA002846-PA	14	14	36.295	132.49	2.1E+07	7.1E+07	2.6E+07	2E+08	3.3559587	0.00032	7.792204	0.05071	Yes
126	enzyme	juvenile hormone esterase	gi|18001001|gb|AAL5524	BGIBMGA000772-PA	26	7	62.628	323.31	2E+07	2.4E+08	9.1E+07	2.8E+09	11.570052	0.00012	31.25119	0.01461	Yes
244	immune-effector	gloverin4	gi|52421211|dbj|BAD5147	BGIBMGA013865-PA	12	6	18.8	106.51	2E+07	0	2.3E+08	0	0	0.0006	0	4.3E-05	
286	immune-recognition	C-type lectin 11	gi|31559111|gb|AAP5084	BGIBMGA006623-PA	13	13	34.511	323.31	2E+07	3.1E+08	3.3E+07	7.3E+08	15.780511	2.6E-05	22.26259	0.03337	Yes
47	binding and transport	30K protein BmLP8	gi|525343731|ref|NP_0012	BGIBMGA004401-PA	23	9	29.383	318.61	2E+07	0	3.1E+08	0	0	0.01297	0	7.8E-05	
152	enzyme	ecdysteroid-inducible	gi|8918492|dbj|BAA97657	BGIBMGA002526-PA	41	41	74.912	323.31	1.9E+07	3.7E+08	1.6E+07	9.8E+08	18.978626	1E-05	62.02592	0.00254	Yes
75	enzyme	vanin-like protein 1	gi|512917546|ref|XP_0049	BGIBMGA012994-PA	23	5	109.29	323.31	1.9E+07	7740836	2E+07	1.6E+08	0.4159336	0.03971	7.885625	0.04293	Yes

17	binding and transport	ommochrome binding protein 2	gi|512895687|ref|XP_0049	BGIBMGA008179-PA	5	5	31.095	240.8	1.8E+07	3.3E+07	1E+07	2.5E+08	1.8312717	0.31738	23.80549	0.01948	Yes
123	enzyme	liver carboxylesterase 1	gi|95102790|gb|ABF5133	BGIBMGA006456-PA	24	24	75.502	276.52	1.8E+07	5.2E+07	7555066	1.7E+07	2.8811606	0.12902	2.21318	0.34704	
378	unknown protein	Uncharacterized protein	gi|512909709|ref|XP_0049	BGIBMGA012645-PA	8	8	16.054	143.45	1.8E+07	4.9E+07	6.2E+07	4.6E+08	2.816131	0.23042	7.424168	0.00078	Yes
146	enzyme	fumarate hydratase	gi|512909065|ref|XP_0049	BGIBMGA008216-PA	14	2	101.14	205.43	1.7E+07	2104007	3.9E+07	6846001	0.1219795	0.0002	0.173893	0.00065	
138	enzyme	glutamate carboxypeptidase	gi|512915932|ref|XP_0049	BGIBMGA007728-PA	22	22	58.864	113.02	1.7E+07	0	3.5E+07	0	0	6.3E-05	0	4.9E-05	
52	binding and transport	30K protein BmLP21	gi|379046492|gb|AFC8780	BGIBMGA004465-PA	14	14	30.674	62.413	1.6E+07	0	3.4E+07	0	0	0.00089	0	0.00033	
31	binding and transport	Ferritin	gi|95102694|gb|ABF51285	BGIBMGA008768-PA	14	14	19.526	132.42	1.6E+07	1E+07	5.5E+07	2.9E+08	0.6333136	0.05972	5.289577	0.00399	Yes
288	immune-recognition	beta-1,3-glucan recognition	—	BGIBMGA011609-PA	18	2	47.632	58.333	1.6E+07	1.6E+07	3.3E+07	1.3E+08	1.0091105	0.98266	3.95847	0.03599	Yes
32	binding and transport	Ecdysteroid-regulated 16 kDa	gi|87248319|gb|ABD3621	BGIBMGA008405-PA	2	2	15.824	24.315	1.6E+07	0	0	2173856	0	0.02526	∞	0.16527	
316	immune-signaling	ENF peptides binding protein	gi|512907075|ref|XP_0049	BGIBMGA009573-PA	18	9	49.087	66.039	1.5E+07	0	1.5E+07	0	0	0.00017	0	0.01445	
236	other	alpha-crystallin, HSP20-	gi|512937756|ref|XP_0049	BGIBMGA014586-PA	10	10	28.694	99.204	1.4E+07	1.1E+07	7178561	5.3E+07	0.8081512	0.71505	7.375217	0.09633	
110	enzyme	Peptidyl-prolyl cis-trans	gi|95103068|gb|ABF51475	BGIBMGA004059-PA	16	16	21.893	124.9	1.3E+07	0	1.3E+08	0	0	5.7E-05	0	2.6E-07	
243	immune-effector	lebocin 5	gi|6016491|sp|P55796.2|LI	BGIBMGA006775-PA	3	3	21.012	11.577	1.3E+07	0	2.7E+07	0	0	0.06828	0	0.01978	
368	unknown protein	Uncharacterized protein	gi|512937706|ref|XP_0049	BGIBMGA014204-PA	13	13	32.404	251.17	1.3E+07	1.4E+08	1.1E+07	6.7E+08	11.271787	0.00066	58.70622	0.00016	Yes
55	binding and transport	30K protein BmLP19	gi|512890719|ref|XP_0049	BGIBMGA004463-PA	9	9	29.799	24.967	1.3E+07	0	9442715	0	0	2.9E-05	0	0.07689	
193	enzyme	alpha-1,2-Mannosidase	gi|512926654|ref|XP_0049	BGIBMGA002426-PA	26	26	61.159	323.31	1.2E+07	2.5E+08	1.2E+07	5.6E+08	20.964007	0.00222	46.88443	0.02338	Yes
310	immune-signaling	serine protease BmSP137/CLIP1	gi|95102990|gb|ABF5143	BGIBMGA009551-PA	18	18	43.345	225.88	1.1E+07	1.8E+07	2.4E+07	2.6E+08	1.6350995	0.56541	11.24993	0.00064	Yes
365	unknown protein	Uncharacterized protein	gi|512886969|ref|XP_0049	BGIBMGA005149-PA	5	5	21.132	69.624	9889225	8.2E+07	9713162	2.3E+08	8.3126832	8.7E-05	23.45852	0.07137	Yes
119	enzyme	Malic enzyme	gi|95103050|gb|ABF5146	BGIBMGA006419-PA	18	18	67.257	119.46	9760003	0	1.4E+07	0	0	3.5E-05	0	6.7E-05	
312	immune-signaling	ENF peptides binding protein	—	BGIBMGA010878-PA	3	2	16.515	25.688	9494813	0	2.1E+07	0	0	0.13541	0	0.00166	
279	immune-recognition	hemocytin, humoral lectin	—	BGIBMGA006694-PA	18	7	74.343	36.235	9365037	0	2512568	0	0	0.00513	0	0.00019	
303	immune-signaling	serine protease CLIP3	gi|95102804|gb|ABF51343	BGIBMGA010257-PA	16	16	43.107	137.29	9273476	6E+07	7585711	1E+08	6.5149009	0.088	13.17835	0.09563	
241	immune-effector	lysozyme-like protein 3	gi|512936646|ref|XP_0049	BGIBMGA014001-PA	4	4	16.841	18.816	9211224	0	1.6E+07	1.6E+07	0	1.1E-05	0.975776	0.8616	
281	immune-recognition	hemocytin, humoral lectin	—	BGIBMGA006692-PA	27	6	85.739	116.33	8648941	0	1849892	0	0	0.00065	0	0.14186	
122	enzyme	ly-6 neurotoxin superfamily	gi|512929412|ref|XP_0049	BGIBMGA008847-PA	4	4	17.871	63.464	8320524	2324698	2.4E+07	2.3E+07	0.2793932	0.0522	0.969469	0.95606	

283	immune-recognition	C-type lectin19	gi|284813581|ref|NP_0011	—	7	2	35.351	87.98	7477136	9203113	1.2E+07	3.9E+07	1.2308339	0.33033	3.313406	0.23516	
84	enzyme	subunit of dna dependent rna-	gi|512919003|ref|XP_0049	BGIBMGA005942-PA	3	3	42.94	14.378	6981858	4.2E+07	1700412	2.5E+07	5.9733967	0.08902	14.56955	0.01302	Yes
124	enzyme	leukotriene a-4 hydrolase	—	BGIBMGA004112-PA	27	2	88.144	242.72	6433864	8.1E+07	8514964	8.1E+07	12.613563	0.0008	9.541869	0.09593	Yes
83	enzyme	Sulfhydryl oxidase	gi|512921696|ref|XP_0049	BGIBMGA000481-PA	22	22	64.649	246.02	6146182	8182755	7323962	6E+07	1.3313559	0.48536	8.15606	0.17463	
311	immune-signaling	hemolymph protein14; MBL-	gi|389620198|gb|AFK9353	BGIBMGA012217-PA	24	24	73.053	323.31	6122166	1.2E+08	1.7E+07	5.6E+08	19.623124	2.2E-06	33.12742	0.02065	Yes
145	enzyme	gamma-glutamylcyclotran	gi|512923155|ref|XP_0049	—	4	4	14.228	20.874	5909205	2.2E+07	9351459	3.3E+08	3.6658378	0.13748	35.79206	0.25332	
128	enzyme	inosine-uridine preferring	gi|512893076|ref|XP_0049	BGIBMGA003331-PA	15	15	33.264	279.25	5875148	1.1E+08	7772395	4E+08	18.330372	0.01329	51.81528	0.01461	Yes
200	enzyme	ADP-dependent NAD(P)H-hydrate	gi|512909717|ref|XP_0049	BGIBMGA012642-PA	13	13	32.733	172.13	5705805	2E+08	9345874	4.5E+08	34.401763	0.00308	47.6938	0.0147	Yes
351	unknown protein	uncharacterized protein	gi|512919035|ref|XP_0049	BGIBMGA005938-PA	13	13	24.487	287.51	5549288	2.6E+08	5788156	7.1E+08	46.580933	0.0019	123.2494	0.00739	Yes
221	extracellular matrix	chaoptin-like	gi|512895038|ref|XP_0049	BGIBMGA006308-PA	2	2	137.63	4.8464	5475808	3584209	1.9E+07	2.7E+07	0.6545535	0.08385	1.388369	0.67544	
160	enzyme	cysteine protease	—	BGIBMGA005131-PA	21	21	206.84	208.37	5472141	7768464	6749920	2.9E+07	1.4196388	0.52811	4.369758	0.00771	Yes
86	enzyme	spermidine synthase	gi|512899765|ref|XP_0049	BGIBMGA005897-PA	4	4	32.427	22.707	5282354	0	0	0	0	5.7E-05	—	#DIV/0!	
141	enzyme	Glucose-6-phosphate	gi|87248657|gb|ABD3638	BGIBMGA004221-PA	10	10	61.982	51.588	5257439	0	7310718	0	0	0.00585	0	0.00171	
40	binding and transport	arylphorin/storage protein 1	gi|169234936|ref|NP_0011	BGIBMGA011266-PA	102	2	87.268	105.78	4882853	8.7E+07	0	0	17.773586	0.04449	—	#DIV/0!	Yes
133	enzyme	GTPase	gi|512922005|ref|XP_0049	BGIBMGA003598-PA	14	14	86.483	67.146	4704086	0	1233060	0	0	0.00034	0	0.00168	
36	binding and transport	Chemosensory protein 9	gi|77415572|emb|CAJ0145	BGIBMGA004035-PA	7	7	14.734	85.652	4626376	0	1.2E+08	0	0	0.01325	0	0.00027	
253	immune-non-serpin type	serine protease inhibitor TIL	gi|512898441|ref|XP_0049	BGIBMGA009095-PA	2	2	8.8603	31.609	4477957	0	3.5E+07	0	0	0.35592	0	0.09469	
143	enzyme	glucose-1-phosphatase	gi|512925150|ref|XP_0049	BGIBMGA011203-PA	5	5	47.511	8.0996	3924451	1286193	0	319254	0.3277383	0.17289	∞	0.35592	
106	enzyme	phosphoric diester hydrolase	gi|512932832|ref|XP_0049	BGIBMGA011531-PA	3	3	49.839	26.811	3906575	0	0	0	0	0.00051	—	#DIV/0!	
65	other	paramyosin	gi|512928928|ref|XP_0049	BGIBMGA008949-PA	21	21	62.896	136.22	3812891	0	1.9E+07	3.4E+07	0	0.00695	1.780695	0.5705	
245	immune-effector	Gloverin3 GN=glv3	gi|52421209|dbj|BAD5147	BGIBMGA013803-PA	8	3	19.029	12.805	3799242	0	1810198	0	0	0.14338	0	0.26346	
308	immune-signaling	serine protease BmSP28/CLIP25	gi|512931268|ref|XP_0049	BGIBMGA005380-PA	7	7	12.192	145.99	3652137	4.1E+07	2924695	1E+08	11.280973	0.11329	34.88798	0.00161	Yes
71	enzyme	xaa-pro aminopeptidase	gi|512889668|ref|XP_0049	BGIBMGA001638-PA	29	29	132.11	323.31	3485522	508546	8397652	5E+07	0.1459025	0.0032	5.936945	0.24473	
307	immune-signaling	serine protease BmSP63/CLIP20	gi|379699022|ref|NP_0012	BGIBMGA009610-PA	12	12	181.32	78.678	3264439	0	3197715	0	0	0.00193	0	0.00057	
340	unknown protein	uncharacterized protein	gi|512907002|ref|XP_0049	BGIBMGA009599-PA	13	13	150.08	142.08	3164510	0	5946615	461197	0	0.00036	0.077556	0.00069	

25	binding and transport	Lipocalin	gi|512909266|ref|XP_0049	BGIBMGA013131-PA	3	3	29.851	23.84	2853623	2.2E+07	0	5312910	7.7120524	0.06233	∞	0.2164	
30	binding and transport	ferritin light chain	gi|95102796|gb|ABF51339	BGIBMGA008780-PA	13	13	26.033	155.42	2813535	1.6E+07	1.6E+07	1.7E+08	5.7696328	0.27459	11.12622	0.00996	Yes
179	enzyme	Beta-galactosidase	gi|255652865|ref|NP_0011	BGIBMGA006815-PA	24	24	66.671	323.31	2688574	2.2E+08	2501833	6.7E+08	81.073762	0.00179	267.2432	0.00534	Yes
389	unknown protein	Uncharacterized protein	—	BGIBMGA001941-PA	2	2	10.749	-2	2668989	0	700243	0	0	0.35592	0	0.35592	
206	extracellular matrix	Putative cuticle protein CPR149	gi|262034563|gb|ACY069	BGIBMGA000348-PA	2	2	31.065	7.2644	2666870	0	3192534	0	0	0.04023	0	0.03122	
188	enzyme	alpha-N-acetylgalactosami	gi|87248331|gb|ABD3621	—	10	2	49.008	68.763	2505634	3.1E+07	0	1.8E+07	12.240274	0.00727	∞	0.02958	Yes
242	immune-effector	lysozyme	gi|112984208|ref|NP_0010	BGIBMGA006216-PA	29	2	187.66	144.45	2238331	4440473	1.5E+07	6.6E+07	1.9838325	0.19846	4.370727	0.05377	
345	unknown protein	uncharacterized protein	gi|512910875|ref|XP_0049	—	7	7	27.92	23.782	2229960	0	8160057	1E+07	0	0.20267	1.273327	0.64799	
174	enzyme	Beta-N-acetylglucosamini	gi|145651816|ref|NP_0010	BGIBMGA011646-PA	31	31	69.654	323.31	2088598	1.7E+08	3008385	2.9E+08	82.055504	0.00956	95.07624	0.00976	Yes
134	enzyme	glyoxylate reductase	gi|512898607|ref|XP_0049	BGIBMGA009043-PA	16	16	38.432	312.02	2000011	2E+08	4643188	5E+08	102.07032	0.01094	108.4285	0.0308	Yes
187	enzyme	Aminoacylase-1 GN=LOC101742	gi|512902991|ref|XP_0049	BGIBMGA011002-PA	17	4	45.296	33.794	1991783	0	1.1E+07	0	0	0.04692	0	5.5E-06	
235	other	Heat shock cognate protein	gi|320526705|gb|ADW417	BGIBMGA002381-PA	18	13	71.175	164.85	1922040	395146	1.4E+07	5263023	0.2055868	0.04521	0.37866	0.02251	
72	enzyme	venom serine carboxypeptidase-	gi|512925630|ref|XP_0049	BGIBMGA003110-PA	3	3	43.079	9.3988	1870970	0	0	596106	0	0.14039	∞	0.19333	
251	immune-non-serpin type	serine protease inhibitor TIL	gi|512898143|ref|XP_0049	BGIBMGA010889-PA	16	16	135.62	323.31	1580003	2.3E+07	1361030	7.9E+07	14.255625	0.02011	58.03588	0.01398	Yes
302	immune-signaling	serine protease CLIP7/12	gi|512909603|ref|XP_0049	BGIBMGA010306-PA	16	10	43.249	197.79	1563273	7.7E+07	907371	1.5E+08	49.489452	0.00015	159.9555	0.01152	Yes
28	binding and transport	Juvenile hormone binding protein	gi|73992794|dbj|BAE4341	BGIBMGA001325-PA	2	2	24.342	12.174	1551515	0	1.2E+07	0	0	0.03172	0	0.0216	
377	unknown protein	Uncharacterized protein	gi|512937361|ref|XP_0049	BGIBMGA014512-PA	6	5	31.619	44.883	1539934	3.2E+07	0	3.8E+07	20.958797	0.05693	∞	0.00426	Yes
230	other	small heat shock protein	gi|512896393|ref|XP_0049	BGIBMGA005755-PA	4	4	26.587	18.613	1412316	0	4.9E+07	4.4E+07	0	0.35592	0.901459	0.66357	
171	enzyme	carboxylic ester hydrolase	gi|512908407|ref|XP_0049	BGIBMGA003917-PA	9	9	37.729	139.15	1367873	1.3E+08	0	2.3E+08	96.747251	0.01034	∞	0.06822	Yes
74	enzyme	venom acid phosphatase acph-	gi|512891983|ref|XP_0049	BGIBMGA009488-PA	3	3	44.212	5.2057	1350185	0	0	1332425	0	0.02705	∞	0.13717	
27	binding and transport	lipid binding protein	gi|518743876|dbj|BAN587	BGIBMGA003344-PA	4	4	51.075	6.7997	1122127	882707	985782	0	0.7866374	0.83419	0	0.06366	
157	enzyme	d-3-phosphoglycerate	gi|512934875|ref|XP_0049	BGIBMGA012309-PA	5	5	34.73	24.322	995877	0	1E+07	0	0	0.31779	0	7.3E-05	
326	other	osiris 9	gi|512906099|ref|XP_0049	BGIBMGA000013-PA	5	5	25.909	33.205	975977	3.4E+07	1.4E+07	3E+07	34.760319	0.00029	2.077714	0.07533	Yes
335	immune-recognition	BmP109	gi|315306480|gb|ADU039	BGIBMGA014221-PA	3	3	108.71	8.2378	964002	0	136966	0	0	0.04535	0	0.35592	
42	binding and transport	apolipoprotein	gi|518743874|dbj|BAN587	BGIBMGA013893-PA	22	10	470.18	84.404	942502	0	682554	0	0	1.2E-05	0	0.01069	

<table>
<tr><td>194</td><td>enzyme</td><td>Alpha amylase GN=LOC100500</td><td>gi|306518660|ref|NP_0011</td><td>BGIBMGA003057-PA</td><td>15</td><td>15</td><td>67.443</td><td>51.799</td><td>919386</td><td>0</td><td>2711581</td><td>1.6E+07</td><td>0</td><td>0.13727</td><td>5.717031</td><td>0.31496</td><td></td></tr>
<tr><td>313</td><td>immune-signaling</td><td>ENF peptides binding protein</td><td>gi|512897933|ref|XP_0049</td><td>BGIBMGA010877-PA</td><td>15</td><td>2</td><td>35.414</td><td>7.8239</td><td>912332</td><td>0</td><td>1783158</td><td>0</td><td>0</td><td>0.15292</td><td>0</td><td>0.03064</td><td></td></tr>
<tr><td>296</td><td>immune-signaling</td><td>serpin-13</td><td>gi|226342886|ref|NP_0011</td><td>BGIBMGA004955-PA</td><td>12</td><td>10</td><td>49.134</td><td>148.41</td><td>909303</td><td>3.3E+07</td><td>2935378</td><td>7.3E+07</td><td>36.249001</td><td>0.00567</td><td>24.72977</td><td>0.00596</td><td>Yes</td></tr>
<tr><td>107</td><td>enzyme</td><td>peroxiredoxin</td><td>gi|38260562|gb|AAR1542</td><td>BGIBMGA002186-PA</td><td>3</td><td>3</td><td>21.916</td><td>17.418</td><td>907624</td><td>0</td><td>541400</td><td>0</td><td>0</td><td>0.35592</td><td>0</td><td>0.35592</td><td></td></tr>
<tr><td>328</td><td>other</td><td>low density lipoprotein</td><td>gi|226531274|ref|NP_0011</td><td>BGIBMGA005876-PA</td><td>38</td><td>38</td><td>143.06</td><td>323.31</td><td>902409</td><td>0</td><td>1.8E+07</td><td>2E+08</td><td>0</td><td>0.29954</td><td>10.8976</td><td>9E-05</td><td>Yes</td></tr>
<tr><td>82</td><td>enzyme</td><td>sulfuric ester hydrolase</td><td>gi|512913875|ref|XP_0049</td><td>BGIBMGA001098-PA</td><td>21</td><td>20</td><td>50.776</td><td>258.76</td><td>848505</td><td>3624631</td><td>1.2E+07</td><td>1.2E+08</td><td>4.2717863</td><td>0.01728</td><td>10.05551</td><td>6.7E-05</td><td>Yes</td></tr>
<tr><td>320</td><td>other</td><td>semaphorin receptor</td><td>gi|512909073|ref|XP_0049</td><td>BGIBMGA008238-PA</td><td>18</td><td>18</td><td>211.78</td><td>109.32</td><td>644270</td><td>1749346</td><td>0</td><td>4395808</td><td>2.7152384</td><td>0.23026</td><td>∞</td><td>0.09485</td><td></td></tr>
<tr><td>112</td><td>enzyme</td><td>N-acetylglucosamine</td><td>gi|512917348|ref|XP_0049</td><td>BGIBMGA012952-PA</td><td>4</td><td>4</td><td>56.564</td><td>20.429</td><td>597780</td><td>0</td><td>315113</td><td>5567005</td><td>0</td><td>0.1539</td><td>17.66667</td><td>0.25313</td><td></td></tr>
<tr><td>70</td><td>enzyme</td><td>zinc-binding alcohol</td><td>gi|512887563|ref|XP_0049</td><td>BGIBMGA005615-PA</td><td>2</td><td>2</td><td>25.991</td><td>3.2192</td><td>579390</td><td>0</td><td>0</td><td>1443040</td><td>0</td><td>0.13696</td><td>∞</td><td>0.13586</td><td></td></tr>
<tr><td>136</td><td>enzyme</td><td>Glyceraldehyde-3-phosphate</td><td>gi|161088520|gb|ABX573</td><td>BGIBMGA007490-PA</td><td>3</td><td>3</td><td>24.089</td><td>15.154</td><td>519865</td><td>0</td><td>9526998</td><td>0</td><td>0</td><td>0.35592</td><td>0</td><td>0.01697</td><td></td></tr>
<tr><td>104</td><td>enzyme</td><td>plasma glutamate carboxypeptidase-</td><td>gi|512913737|ref|XP_0049</td><td>BGIBMGA001275-PA</td><td>22</td><td>22</td><td>52.373</td><td>194.79</td><td>424580</td><td>6.1E+07</td><td>583791</td><td>9.3E+07</td><td>144.74351</td><td>0.00325</td><td>159.6577</td><td>0.00406</td><td>Yes</td></tr>
<tr><td>195</td><td>enzyme</td><td>aldosereductase-like</td><td>gi|512935629|ref|XP_0049</td><td>BGIBMGA009800-PA</td><td>2</td><td>2</td><td>38.624</td><td>5.3971</td><td>418315</td><td>0</td><td>1.4E+07</td><td>0</td><td>0</td><td>0.35592</td><td>0</td><td>0.00012</td><td></td></tr>
<tr><td>170</td><td>enzyme</td><td>carboxylic ester hydrolase</td><td>gi|82792184|gb|ABB9096</td><td>BGIBMGA002670-PA</td><td>2</td><td>2</td><td>31.736</td><td>5.326</td><td>374107</td><td>0</td><td>0</td><td>0</td><td>0</td><td>0.35592</td><td>—</td><td>#DIV/0!</td><td></td></tr>
<tr><td>205</td><td>extracellular matrix</td><td>thrombospondin</td><td>gi|512936253|ref|XP_0049</td><td>BGIBMGA001836-PA</td><td>18</td><td>18</td><td>89.963</td><td>249.35</td><td>329097</td><td>0</td><td>3793303</td><td>5.5E+07</td><td>0</td><td>0.31945</td><td>14.4372</td><td>0.00831</td><td>Yes</td></tr>
<tr><td>154</td><td>enzyme</td><td>dna replication licensing factor</td><td>gi|512922551|ref|XP_0049</td><td>BGIBMGA003507-PA</td><td>8</td><td>8</td><td>59.411</td><td>50.202</td><td>307727</td><td>0</td><td>1899086</td><td>8250432</td><td>0</td><td>0.15088</td><td>4.344422</td><td>0.02883</td><td>Yes</td></tr>
<tr><td>156</td><td>enzyme</td><td>deoxyribonucleas e i</td><td>gi|512910144|ref|XP_0049</td><td>BGIBMGA004667-PA</td><td>10</td><td>10</td><td>42.841</td><td>149.78</td><td>271966</td><td>0</td><td>6987896</td><td>7876113</td><td>0</td><td>0.35592</td><td>1.127108</td><td>0.89724</td><td></td></tr>
<tr><td>373</td><td>unknown protein</td><td>Uncharacterized protein</td><td>gi|512893821|ref|XP_0049</td><td>BGIBMGA010043-PA</td><td>7</td><td>3</td><td>31.804</td><td>72.992</td><td>257895</td><td>2.8E+07</td><td>0</td><td>1E+08</td><td>108.51947</td><td>0.05074</td><td>∞</td><td>0.00108</td><td>Yes</td></tr>
<tr><td>219</td><td>extracellular matrix</td><td>connectin-like</td><td>gi|512915242|ref|XP_0049</td><td>BGIBMGA008307-PA</td><td>23</td><td>23</td><td>63.476</td><td>323.31</td><td>247912</td><td>2336368</td><td>6246889</td><td>3.7E+08</td><td>9.4241776</td><td>0.0035</td><td>59.01544</td><td>0.00117</td><td>Yes</td></tr>
<tr><td>89</td><td>enzyme</td><td>serine protease BmSP53</td><td>gi|512917698|ref|XP_0049</td><td>BGIBMGA013049-PA</td><td>15</td><td>15</td><td>67.397</td><td>124.05</td><td>239510</td><td>1.2E+07</td><td>2723126</td><td>8.3E+07</td><td>51.949213</td><td>0.003</td><td>30.40243</td><td>0.01126</td><td>Yes</td></tr>
<tr><td>384</td><td>unknown protein</td><td>Putative fatbody protein 3Rev-G1</td><td>gi|512894507|ref|XP_0049</td><td>BGIBMGA013782-PA</td><td>2</td><td>2</td><td>13.318</td><td>9.8942</td><td>229362</td><td>0</td><td>2.2E+07</td><td>6854214</td><td>0</td><td>0.35592</td><td>0.317604</td><td>0.13109</td><td></td></tr>
<tr><td>97</td><td>enzyme</td><td>Putative lipase</td><td>gi|512905828|ref|XP_0049</td><td>BGIBMGA009162-PA</td><td>10</td><td>10</td><td>40.321</td><td>129.23</td><td>214005</td><td>0</td><td>623079</td><td>4E+07</td><td>0</td><td>0.35592</td><td>63.74138</td><td>0.07513</td><td></td></tr>
<tr><td>233</td><td>other</td><td>Heat shock protein 83</td><td>gi|328774765|gb|AEB3978</td><td>BGIBMGA004612-PA</td><td>3</td><td>3</td><td>78.594</td><td>4.0857</td><td>184828</td><td>7248953</td><td>0</td><td>0</td><td>39.220062</td><td>0.36759</td><td>—</td><td>#DIV/0!</td><td></td></tr>
<tr><td>147</td><td>enzyme</td><td>Fructose-bisphosphate</td><td>gi|148298833|ref|NP_0010</td><td>BGIBMGA013021-PA</td><td>5</td><td>5</td><td>81.745</td><td>12.245</td><td>170901</td><td>0</td><td>714076</td><td>0</td><td>0</td><td>0.14428</td><td>0</td><td>0.1997</td><td></td></tr>
<tr><td>367</td><td>binding and transport</td><td>apolipophorins-like</td><td>gi|512936795|ref|XP_0049</td><td>BGIBMGA013894-PA</td><td>14</td><td>2</td><td>292.8</td><td>6.4911</td><td>147348</td><td>0</td><td>0</td><td>0</td><td>0</td><td>0.18995</td><td>—</td><td>#DIV/0!</td><td></td></tr>
<tr><td>349</td><td>unknown protein</td><td>uncharacterized protein</td><td>gi|512912007|ref|XP_0049</td><td>—</td><td>11</td><td>11</td><td>88.889</td><td>77.966</td><td>124172</td><td>0</td><td>2562412</td><td>7027507</td><td>0</td><td>0.35592</td><td>2.742536</td><td>0.0499</td><td>Yes</td></tr>
</table>

1	binding and transport	zinc transporter foi-like	gi|512939606|ref|XP_0049	BGIBMGA009645-PA	3	3	244.53	6.1419	116795	756732	0	2337088	6.4791573	0.21761	∞	0.00065	Yes
92	enzyme	ser thr protein phosphatase	gi|512918105|ref|XP_0049	BGIBMGA012947-PA	3	3	57.09	23.273	96690.4	4411443	0	0	45.624408	0.14991	—	#DIV/0!	
382	unknown protein	Uncharacterized protein	gi|512921357|ref|XP_0049	BGIBMGA000544-PA	11	11	75.491	55.995	93353.5	0	5790103	307932	0	0.35592	0.053182	0.01156	
210	extracellular matrix	laminin subunit beta-1	gi|512914161|ref|XP_0049	BGIBMGA000910-PA	9	9	197.5	29.254	69474.9	0	1070016	0	0	0.35592	0	0.00263	
211	extracellular matrix	laminin subunit alpha-like	gi|512933761|ref|XP_0049	BGIBMGA002018-PA	26	26	405.14	94.025	51677.9	0	1549101	405501	0	0.35592	0.261765	0.07828	
319	other	wing disc-specific protein	gi|17298115|dbj|BAB7852	BGIBMGA003481-PA	2	2	115.96	7.5287	46738.9	0	251222	2285014	0	0.35592	9.095605	0.02446	Yes
212	extracellular matrix	Laminin	gi|512933533|ref|XP_0049	BGIBMGA002114-PA	6	6	174.74	19.462	37834.8	0	841425	0	0	0.35592	0	0.00629	
327	other	LOWQUALITYPROTEIN	gi|512890270|ref|XP_0049	BGIBMGA004090-PA	2	2	581.72	5.0886	15014.5	0	0	1557349	0	0.35592	∞	0.09571	
270	immune-other	Putative defense protein Reeler	gi|325660637|gb|ADZ4041	BGIBMGA014360-PA	8	8	18.482	283.84	0	2924219	7E+07	2E+09	∞	0.35592	28.04025	0.00506	Yes
273	immune-recognition	immunoglobulin superfamily	gi|258642521|gb|ACQ828	BGIBMGA008736-PA	23	23	44.849	323.31	0	0	1.9E+08	1.9E+09	—	#DIV/0!	9.700121	0.00134	Yes
369	unknown protein	Uncharacterized protein	gi|512925198|ref|XP_0049	BGIBMGA011399-PA	9	9	16.046	317.71	0	0	1.2E+08	1.6E+09	—	#DIV/0!	13.75002	0.00907	Yes
169	enzyme	Carboxylic ester hydrolase	gi|168823411|ref|NP_0011	BGIBMGA013812-PA	27	26	63.296	318.72	0	7870938	7936183	5.2E+08	∞	0.20236	65.54594	6.1E-05	Yes
386	unknown protein	hypothetical protein	gi|73992802|dbj|BAE4341	BGIBMGA001164-PA	8	8	27.768	104.57	0	0	1.1E+07	2.3E+08	—	#DIV/0!	20.39559	0.00163	Yes
354	unknown protein	uncharacterized protein	gi|512911134|ref|XP_0049	BGIBMGA009184-PA	29	29	90.68	323.31	0	0	2.1E+07	1.7E+08	—	#DIV/0!	8.291293	0.06789	
255	immune-non-serpin type	serine protease inhibitor TIL	gi|512898429|ref|XP_0049	BGIBMGA009093-PA	5	5	22.654	39.202	0	170896	2.7E+07	1.2E+08	∞	0.35592	4.643067	0.03982	Yes
197	enzyme	aldo-keto reductase	gi|512935651|ref|XP_0049	BGIBMGA009801-PA	14	14	37.728	167.76	0	0	1.3E+07	1.5E+08	—	#DIV/0!	11.13657	0.0016	Yes
6	binding and transport	vitellogenin	gi|871834|dbj|BAA06397.	BGIBMGA004585-PA	121	121	203.09	323.31	0	0	2.8E+09	9.6E+07	—	#DIV/0!	0.034132	4.4E-06	
271	immune-other	Bm8 interacting protein 2d-4	gi|306518642|ref|NP_0011	BGIBMGA012968-PA	8	8	48.29	199.97	0	0	5593918	1E+08	—	#DIV/0!	18.68119	0.0035	Yes
135	enzyme	glycerophosphodiester	gi|32997080|dbj|BAC7938	BGIBMGA007767-PA	17	17	41.45	323.31	0	1.8E+08	373293	3.8E+08	∞	0.00332	1020.848	0.03074	Yes
164	enzyme	chitinase	gi|290560651|ref|NP_0011	BGIBMGA010240-PA	21	21	60.969	303.24	0	0	4.9E+07	7.1E+07	—	#DIV/0!	1.43607	0.49947	
115	enzyme	molting fluid carboxypeptidase	gi|54114893|dbj|BAD6091	BGIBMGA008910-PA	12	12	53.979	48.229	0	0	4551499	1E+08	—	#DIV/0!	22.03251	0.02096	Yes
185	enzyme	Antennal esterase	gi|298566244|ref|NP_0011	BGIBMGA004229-PA	14	14	59.19	260.13	0	0	4165737	1.6E+08	—	#DIV/0!	37.56029	6.3E-06	Yes
33	binding and transport	division abnormally	gi|512892728|ref|XP_0049	BGIBMGA003353-PA	29	29	60.9	323.31	0	1961807	4697782	2.5E+08	∞	0.2408	53.44547	0.00891	Yes
192	enzyme	alpha-esterase 19	gi|174840656|ref|NP_0011	BGIBMGA000837-PA	21	6	61.295	297.78	0	0	2675524	1.3E+08	—	#DIV/0!	47.1332	0.00027	Yes
282	immune-recognition	hemicentin-1-like	gi|512892086|ref|XP_0049	BGIBMGA009442-PA	16	16	72.035	280.23	0	0	864942	6.5E+07	—	#DIV/0!	75.12316	0.03763	Yes

4	binding and transport	yellow-d	gi|86450717|gb|ABC9669	BGIBMGA007254-PA	18	18	50.358	323.31	0	2.8E+07	2578414	2.2E+08	∞	0.05488	84.64808	0.00216	Yes
274	immune-recognition	immunoglobulin superfamily	gi|512895113|ref|XP_0049	BGIBMGA006339-PA	7	7	30.761	35.537	0	0	555819	1.7E+08	—	#DIV/0!	297.3292	0.00171	Yes
259	immune-non-serpin type	serine protease inhibitor kunitz	gi|512912752|ref|XP_0049	BGIBMGA001091-PA	27	27	85.443	262.5	0	1.8E+07	620687	9.4E+07	∞	0.05394	151.8109	0.0018	Yes
198	enzyme	aldo-keto reductase	gi|512908819|ref|XP_0049	BGIBMGA001367-PA	8	8	37.229	124.99	0	0	0	6.7E+07	—	#DIV/0!	∞	0.03387	Yes
359	unknown protein	uncharacterized protein	gi|512925035|ref|XP_0049	BGIBMGA011362-PA	9	9	176.3	319.76	0	0	2811826	6.5E+07	—	#DIV/0!	23.28621	0.02873	Yes
19	binding and transport	odorant binding protein	gi|237648972|ref|NP_0011	BGIBMGA011459-PA	7	7	28.895	28.969	0	0	3145964	7.8E+07	—	#DIV/0!	24.94369	0.00463	Yes
376	unknown protein	Uncharacterized protein	gi|512900823|ref|XP_0049	BGIBMGA003255-PA	5	5	41.649	112.81	0	3.2E+07	0	1.6E+08	∞	0.14873	∞	0.00615	Yes
275	immune-recognition	immunoglobulin superfamily	gi|512925142|ref|XP_0049	BGIBMGA011387-PA	6	6	41.236	64.718	0	1485208	0	5.5E+07	∞	0.35592	∞	0.02912	Yes
276	immune-recognition	immunoglobulin superfamily	gi|512895990|ref|XP_0049	BGIBMGA009336-PA	11	11	98.549	161.73	0	2209997	0	5.1E+07	∞	0.21883	∞	0.03809	Yes
258	immune-non-serpin type	serine protease inhibitor kunitz	gi|512886752|ref|XP_0049	BGIBMGA005129-PA	55	55	339.38	323.31	0	1035572	1990656	4.5E+07	∞	0.2318	22.46505	0.01574	Yes
140	enzyme	glucosidase	gi|30142168|gb|AAP1385	BGIBMGA003512-PA	13	12	57.117	114.51	0	0	1020431	5.4E+07	—	#DIV/0!	52.64936	0.00948	Yes
222	extracellular matrix	Cadherin	gi|512894737|ref|XP_0049	BGIBMGA004592-PA	22	22	104.62	161.91	0	0	286989	3.8E+07	—	#DIV/0!	131.8053	0.01894	Yes
387	unknown protein	Uncharacterized protein	—	BGIBMGA004793-PA	2	2	9.5309	36.13	0	6147488	0	8.3E+07	∞	0.35592	∞	0.07745	
189	enzyme	alpha-n-acetyl glucosaminidase	—	BGIBMGA009235-PA	20	20	90.297	159.65	0	0	1367844	3.2E+07	—	#DIV/0!	23.28601	0.0772	
163	enzyme	Chitinase GN=BmChi-h	gi|29467722|dbj|BAC6724	BGIBMGA008709-PA	21	21	60.79	135.59	0	0	4E+07	3.5E+07	—	#DIV/0!	0.875912	0.69532	
381	extracellular matrix	Uncharacterized protein	gi|512935047|ref|XP_0049	BGIBMGA012240-PA	62	45	425.72	323.31	0	775424	5956152	2.8E+07	∞	0.2378	4.761392	0.08385	
309	immune-signaling	serine protease BmSP24/CLIP26	—	BGIBMGA014407-PA	8	8	39.059	84.29	0	2.4E+07	863119	4.9E+07	∞	0.03631	56.65452	0.00043	Yes
239	immune-effector	prophenoloxidase activating enzyme	gi|4521258|dbj|BAA76308	—	11	3	47.971	154.59	0	0	0	3.6E+07	—	#DIV/0!	∞	0.00754	Yes
196	enzyme	aldo-keto reductase	gi|379698180|dbj|BAL703	BGIBMGA012831-PA	8	8	34.352	32.329	0	0	1314250	2.7E+07	—	#DIV/0!	20.23764	0.06763	
23	binding and transport	Lipocalin	gi|13928531|dbj|BAB4715	BGIBMGA004865-PA	2	2	17.996	25.211	0	2413997	728122	3.6E+07	∞	0.35592	49.25057	0.0028	Yes
177	enzyme	Beta-glucuronidase	gi|512931427|ref|XP_0049	BGIBMGA005500-PA	15	15	61.022	130.62	0	0	4034613	2.9E+07	—	#DIV/0!	7.155517	0.09962	
80	enzyme	TIN-ag-RP	gi|81303350|gb|ABB7110	BGIBMGA004421-PA	7	7	45.669	210.72	0	0	6133584	3.1E+07	—	#DIV/0!	5.018253	0.0058	Yes
285	immune-recognition	C-type lectin 19	—	BGIBMGA002289-PA	6	2	27.312	4.5942	0	0	1.6E+07	2.2E+07	—	#DIV/0!	1.378766	0.68044	
183	enzyme	antennal esterase cxe5	gi|189181680|ref|NP_0011	BGIBMGA009544-PA	14	14	64.158	141.99	0	0	1.2E+08	2.2E+07	—	#DIV/0!	0.186931	0.41746	
15	binding and transport	ommochrome-bindingprotein-	gi|512899467|ref|XP_0049	BGIBMGA007527-PA	4	4	32.192	47.799	0	506534	0	1.4E+07	∞	0.35592	∞	0.11384	

301	immune-signaling	serine protease CLIP8	gi|512919765|ref|XP_0049	BGIBMGA010546-PA	14	14	41.356	106.46	0	6081463	3785191	7.8E+07	∞	0.00027	20.50678	0.09204	Yes
372	unknown protein	Uncharacterized protein	gi|512893824|ref|XP_0049	BGIBMGA010044-PA	4	4	31.781	42.185	0	5.2E+07	0	3.9E+07	∞	0.00295	∞	0.01553	Yes
113	enzyme	myrosinase1-like	gi|512934737|ref|XP_0049	—	7	6	52.867	76.909	0	0	0	1.8E+07	—	#DIV/0!	∞	0.08655	
217	extracellular matrix	fibrillin 2	gi|512937156|ref|XP_0049	BGIBMGA000209-PA	41	41	280.85	323.31	0	0	3381418	1.4E+07	—	#DIV/0!	4.137378	0.09303	
339	unknown protein	uncharacterized protein	gi|512922322|ref|XP_0049	BGIBMGA003660-PA	4	4	22.885	33.302	0	0	1.3E+07	7145945	—	#DIV/0!	0.529959	0.41802	
98	enzyme	protease m1 zinc metalloprotease	gi|389568608|gb|AFK8502	BGIBMGA010764-PA	25	25	107.43	212.36	0	3361374	563520	3.1E+07	∞	0.26367	54.3871	0.09658	
162	enzyme	chitinase-related protein 1	gi|13537190|dbj|BAB4077	BGIBMGA006874-PA	32	32	303.4	256.21	0	0	1141516	1.9E+07	—	#DIV/0!	16.53204	0.03827	Yes
294	immune-signaling	serpin-16	gi|226342892|ref|NP_0011	BGIBMGA003292-PA	8	5	44.367	27.144	0	0	2149948	1.2E+07	—	#DIV/0!	5.420769	0.18619	
357	unknown protein	uncharacterized protein	gi|512911073|ref|XP_0049	BGIBMGA009180-PA	6	6	30.379	80.773	0	7949821	0	3.5E+07	∞	0.14651	∞	0.04693	Yes
346	unknown protein	uncharacterized protein	gi|512916072|ref|XP_0049	BGIBMGA007828-PA	3	3	23.768	32.684	0	0	0	1.4E+07	—	#DIV/0!	∞	0.06967	
231	other	small heat shock protein	gi|512937752|ref|XP_0049	BGIBMGA014587-PA	6	6	28.313	23.897	0	3576939	419885	1.9E+07	∞	0.16949	45.70353	0.17063	
347	unknown protein	uncharacterized protein	gi|512903132|ref|XP_0049	BGIBMGA010627-PA	3	3	29.249	33.449	0	0	0	1.6E+07	—	#DIV/0!	∞	0.01438	Yes
3	binding and transport	yellow-fa	gi|379046452|gb|AFC8778	—	6	6	39.001	38.363	0	0	0	1.2E+07	—	#DIV/0!	∞	0.13295	
102	enzyme	probable chitinase3-like	gi|512916372|ref|XP_0049	BGIBMGA007678-PA	6	6	32.559	20.977	0	0	0	1.4E+07	—	#DIV/0!	∞	0.05471	
260	immune-non-serpin type	serine protease inhibitor Kunitz	gi|27151479|sp|P81902.1|(	—	2	2	6.0267	3.0899	0	0	1.2E+08	2E+07	—	#DIV/0!	0.167973	8.8E-05	
99	enzyme	prolylendopeptidase-like	gi|512932046|ref|XP_0049	BGIBMGA002593-PA	11	11	75.333	80.759	0	0	280066	1.5E+07	—	#DIV/0!	51.97286	0.07281	
360	unknown protein	uncharacterized protein	gi|512902459|ref|XP_0049	BGIBMGA014264-PA	2	2	57.683	3.3949	0	0	1700469	1.3E+07	—	#DIV/0!	7.576581	0.02058	Yes
334	other	dorsal-ventral patterning protein	gi|512931348|ref|XP_0049	BGIBMGA005348-PA	16	16	88.053	84.843	0	0	0	2E+07	—	#DIV/0!	∞	1.2E-07	Yes
374	unknown protein	Uncharacterized protein	gi|512893817|ref|XP_0049	BGIBMGA010040-PA	7	4	31.984	24.793	0	2E+07	0	1.9E+07	∞	0.02472	∞	0.00023	Yes
79	enzyme	transmembrane protease serine 2-	gi|512903587|ref|XP_0049	—	8	4	49.765	91.378	0	5142285	3949867	3.2E+07	∞	0.23437	8.019212	0.00494	Yes
261	immune-non-serpin type	serine protease inhibitor kazal	gi|512898569|ref|XP_0049	BGIBMGA009047-PA	2	2	20.925	10.277	0	0	1193099	5914639	—	#DIV/0!	4.957375	0.27912	
139	enzyme	Glucosidase II alpha-subunit	gi|530233753|ref|NP_0012	BGIBMGA000132-PA	18	18	96.288	99.586	0	876322	242378	1.8E+07	∞	0.35592	75.7243	0.00095	Yes
201	enzyme	adenosinedeaminase AGSA-like	gi|512917269|ref|XP_0049	BGIBMGA012938-PA	8	8	59.101	47.165	0	0	767836	1.1E+07	—	#DIV/0!	14.56637	0.01031	Yes
85	enzyme	spermine oxidase	gi|512889701|ref|XP_0049	BGIBMGA001813-PA	9	9	57.487	84.824	0	2.3E+07	0	2.7E+07	∞	0.03973	∞	0.00173	Yes
338	unknown protein	uncharacterized protein	gi|512886114|ref|XP_0049	BGIBMGA011047-PA	7	7	48.289	37.783	0	1E+07	0	1.1E+07	∞	0.07582	∞	0.0024	Yes

256	immune-non-serpin type	serine protease inhibitor TIL	gi|512903709|ref|XP_0049	BGIBMGA006235-PA	5	5	43.157	8.387	0	0	0	1.5E+07	—	#DIV/0!	∞	0.18836	
223	extracellular matrix	basement membrane-	gi|512935237|ref|XP_0049	BGIBMGA012239-PA	26	9	196.16	177.4	0	699058	1769836	1.7E+07	∞	0.03275	9.578413	0.00055	Yes
130	enzyme	hydrolase	gi|512901127|ref|XP_0049	BGIBMGA007199-PA	20	19	72.822	158.81	0	3902634	0	4.8E+07	∞	0.30411	∞	0.01485	Yes
168	enzyme	Carboxylic ester hydrolase	gi|512910396|ref|XP_0049	BGIBMGA004683-PA	12	11	63.224	39.464	0	0	126589	1.4E+07	—	#DIV/0!	113.7271	0.03612	Yes
121	enzyme	lysosomal alpha-mannosidase	gi|512937736|ref|XP_0049	BGIBMGA014566-PA	5	5	37.46	48.858	0	0	1.3E+07	9028452	—	#DIV/0!	0.71817	0.58734	
155	enzyme	dipeptidase1-like	gi|512933693|ref|XP_0049	BGIBMGA002050-PA	4	4	40.29	39.555	0	0	0	2.6E+07	—	#DIV/0!	∞	0.07173	
202	enzyme	acidtrehalase-like protein 1-like	gi|512925622|ref|XP_0049	BGIBMGA003052-PA	11	11	77.517	87.704	0	0	0	2E+07	—	#DIV/0!	∞	0.00026	Yes
353	unknown protein	uncharacterized protein	gi|512937392|ref|XP_0049	BGIBMGA014522-PA	4	4	33.318	26.528	0	0	0	2.8E+07	—	#DIV/0!	∞	0.12932	
361	unknown protein	uncharacterized protein	gi|379698952|ref|NP_0012	BGIBMGA012766-PA	4	4	34.886	11.787	0	0	1260796	6981836	—	#DIV/0!	5.537641	0.04966	Yes
78	enzyme	trehalase	gi|685089|gb|AAC60507.1	BGIBMGA005664-PA	5	5	66.541	38.858	0	0	0	7492448	—	#DIV/0!	∞	0.15092	
208	extracellular matrix	peritrophin-1-like	gi|512916368|ref|XP_0049	BGIBMGA007901-PA	2	2	23.674	25.092	0	0	0	9805093	—	#DIV/0!	∞	0.18911	
358	unknown protein	uncharacterized protein	gi|537544628|ref|NP_0012	BGIBMGA014224-PA	10	10	49.77	58.809	0	0	0	1.3E+07	—	#DIV/0!	∞	0.00216	Yes
204	extracellular matrix	type iv collagen	—	BGIBMGA014039-PA	3	3	194.21	33.818	0	483512	1337480	5812453	∞	0.1361	4.345824	0.05077	
12	binding and transport	protein takeout-like	gi|512910333|ref|XP_0049	BGIBMGA001308-PA	2	2	24.062	4.9408	0	0	0	8922346	—	#DIV/0!	∞	0.0272	Yes
325	other	probable salivary secreted peptide-	gi|512902241|ref|XP_0049	BGIBMGA004294-PA	2	2	13.07	4.021	0	0	0	8121281	—	#DIV/0!	∞	0.17824	
343	unknown protein	uncharacterized protein	gi|512916157|ref|XP_0049	—	5	5	168.02	24.393	0	0	0	8974084	—	#DIV/0!	∞	0.00309	Yes
300	immune-signaling	serine protease snake-like	gi|512903467|ref|XP_0049	BGIBMGA012425-PA	9	9	52.407	55.658	0	0	0	1.4E+07	—	#DIV/0!	∞	0.00212	Yes
291	immune-signaling	serpin-6	gi|160333383|ref|NP_0011	BGIBMGA007729-PA	10	10	46.466	107.76	0	0	0	3.7E+07	—	#DIV/0!	∞	0.01908	Yes
101	enzyme	probable maltaseH-like	gi|512925606|ref|XP_0049	BGIBMGA003056-PA	7	7	66.102	21.023	0	0	0	8086703	—	#DIV/0!	∞	0.08786	
180	enzyme	Beta-fructofuranosidas	gi|187281652|ref|NP_0011	BGIBMGA005696-PA	9	9	55.941	66.846	0	1E+07	214161	3.1E+07	∞	0.02059	146.1806	0.01507	Yes
96	enzyme	putative polypeptide N-	gi|512897354|ref|XP_0049	BGIBMGA005279-PA	7	7	69.749	32.777	0	0	0	7911661	—	#DIV/0!	∞	0.00075	Yes
225	other	notch-like protein	gi|87248377|gb|ABD3624	—	7	7	121.75	55	0	0	0	3809324	—	#DIV/0!	∞	0.13398	
34	binding and transport	chlorophyllide A binding protein	gi|82940307|emb|CAJ3465	BGIBMGA004806-PA	15	15	301.99	96.537	0	0	0	6972326	—	#DIV/0!	∞	0.04147	Yes
321	other	rna-binding protein 12	—	BGIBMGA000691-PA	4	4	125.82	90.751	0	5070440	648938	2.7E+07	∞	0.27509	41.99291	0.01273	Yes
370	unknown protein	Uncharacterized protein	gi|512894936|ref|XP_0049	BGIBMGA006320-PA	5	5	76.383	46.122	0	0	0	1.3E+07	—	#DIV/0!	∞	0.03131	Yes

125	enzyme	juvenile hormone esterase-like	gi|512903970|ref|XP_0049	—	6	2	52.886	28.756	0	0	0	1.1E+07	—	#DIV/0!	∞	0.00283	Yes
114	enzyme	multiple inositolpolyphosp	gi|512929861|ref|XP_0049	BGIBMGA000402-PA	3	3	50.37	19.592	0	0	0	5903660	—	#DIV/0!	∞	0.0906	
379	unknown protein	Uncharacterized protein	gi|512936608|ref|XP_0049	BGIBMGA013991-PA	2	2	30.321	28.982	0	0	0	4951807	—	#DIV/0!	∞	0.12153	
350	unknown protein	uncharacterized protein	gi|512893813|ref|XP_0049	BGIBMGA010041-PA	6	4	32.133	30.387	0	578873	0	1.6E+07	∞	0.35592	∞	0.01589	Yes
209	extracellular matrix	mucin-3A	—	BGIBMGA006846-PA	5	5	335.03	53.983	0	0	374245	2431336	—	#DIV/0!	6.496643	0.18685	
292	immune-signaling	serpin-3	gi|87248403|gb|ABD3625	BGIBMGA010212-PA	9	9	51.671	49.484	0	4048353	0	1.2E+07	∞	0.07337	∞	0.00105	Yes
323	other	protein unzipped-like	gi|512925807|ref|XP_0049	BGIBMGA003021-PA	6	6	47.777	45.428	0	0	0	8691119	—	#DIV/0!	∞	0.10326	
277	immune-recognition	immunoglobulin superfamily	gi|512895729|ref|XP_0049	BGIBMGA008133-PA	6	6	157.18	70.728	0	0	0	6570600	—	#DIV/0!	∞	0.00034	Yes
11	binding and transport	protein takeout-like	gi|512932963|ref|XP_0049	BGIBMGA011457-PA	2	2	27.371	4.7073	0	0	0	4061905	—	#DIV/0!	∞	0.16108	
5	binding and transport	Yellow-8	gi|379046458|gb|AFC8779	BGIBMGA014026-PA	2	2	52.216	5.9562	0	0	0	2168467	—	#DIV/0!	∞	0.11583	
203	enzyme	5 -nucleotidase	gi|512917317|ref|XP_0049	BGIBMGA012948-PA	4	4	60.111	16.783	0	1014906	0	2677316	∞	0.35592	∞	0.04674	Yes
116	enzyme	metallocarboxypeptidase	gi|512930275|ref|XP_0049	BGIBMGA000307-PA	2	2	54.054	6.5121	0	0	189267	1764142	—	#DIV/0!	9.320942	0.21826	
299	immune-signaling	serpin-10	gi|226342882|ref|NP_0011	BGIBMGA010214-PA	3	3	53.874	16.742	0	0	0	4731298	—	#DIV/0!	∞	0.05922	
142	enzyme	glucose-1-phosphatase	gi|512925482|ref|XP_0049	BGIBMGA011204-PA	4	4	37.122	11.953	0	0	0	7481859	—	#DIV/0!	∞	0.0004	Yes
226	other	notch-like protein	—	BGIBMGA006856-PA	33	33	860.26	154.88	0	0	0	2213469	—	#DIV/0!	∞	0.1235	
331	other	frizzled-5-like	gi|512906986|ref|XP_0049	BGIBMGA009598-PA	2	2	226.72	3.0649	0	0	0	1954885	—	#DIV/0!	∞	0.09412	
161	enzyme	chitooligosaccharidolytic beta-N-	gi|998377|gb|AAC60521.1	—	20	2	68.213	8.4287	0	0	1148151	2901422	—	#DIV/0!	2.527039	0.37677	
264	immune-non-serpin type	serine protease inhibitor	gi|512911303|ref|XP_0049	BGIBMGA013655-PA	2	2	55.093	13.574	0	0	0	1932801	—	#DIV/0!	∞	0.13627	
117	enzyme	matrixmetalloproteinase-25-like	gi|512885379|ref|XP_0049	BGIBMGA002885-PA	7	7	59.086	21.487	0	0	134007	9165364	—	#DIV/0!	68.3945	0.04031	Yes
120	enzyme	lysosomal alpha-mannosidase-like	gi|512889506|ref|XP_0049	—	8	3	83.279	66.1	0	0	4976907	1880090	—	#DIV/0!	0.377763	0.16382	
137	enzyme	glutamyl aminopeptidase	gi|512903235|ref|XP_0049	BGIBMGA010679-PA	15	15	107.99	44.685	0	0	0	5259497	—	#DIV/0!	∞	0.21184	
100	enzyme	procollagen- -oxoglutarate 5-	gi|512921489|ref|XP_0049	BGIBMGA000703-PA	4	4	51.878	24.12	0	0	0	3900912	—	#DIV/0!	∞	0.03047	Yes
9	binding and transport	Secreted protein acidic and rich in	gi|95102834|gb|ABF51358	BGIBMGA013448-PA	12	12	37.384	51.14	0	0	1329344	1.5E+07	—	#DIV/0!	11.06719	0.06827	
232	other	reticulocalbin-2-like	gi|512939713|ref|XP_0049	—	3	3	128.33	7.9172	0	0	0	1881069	—	#DIV/0!	∞	0.10689	
329	other	leucine-rich repeat-containing	gi|512931641|ref|XP_0049	BGIBMGA002675-PA	3	3	77.492	19.518	0	0	0	1552703	—	#DIV/0!	∞	0.13508	

77	enzyme	ubiquitin-protein ligase	gi|512927111|ref|XP_0049	BGIBMGA002518-PA	3	3	73.913	41.8	0	971231	0	9837526	∞	0.14966	∞	0.01935	Yes
298	immune-signaling	serpin-11	gi|226342884|ref|NP_0011	BGIBMGA010213-PA	5	5	46.16	22.945	0	0	0	4153496	—	#DIV/0!	∞	0.12027	
214	extracellular matrix	Fibronectin type-III	gi|512900304|ref|XP_0049	BGIBMGA006817-PA	10	10	194.84	34.803	0	0	0	1629365	—	#DIV/0!	∞	0.14375	
149	enzyme	extracellular serine/threonine	gi|512932324|ref|XP_0049	BGIBMGA007012-PA	3	3	51.434	5.709	0	0	0	1520017	—	#DIV/0!	∞	0.05504	
103	enzyme	plasma kallikrein-like	gi|512903478|ref|XP_0049	BGIBMGA012423-PA	4	4	65.008	8.4038	0	0	0	3571541	—	#DIV/0!	∞	0.13302	
87	enzyme	serine-type endopeptidase	gi|512908708|ref|XP_0049	BGIBMGA002365-PA	8	8	132.81	28.782	0	0	0	5687479	—	#DIV/0!	∞	0.1853	
172	enzyme	carboxyl/cholinest erase 6	gi|306518658|ref|NP_0011	BGIBMGA000774-PA	5	5	63.809	23.47	0	0	0	2611394	—	#DIV/0!	∞	0.14303	
181	enzyme	beta lysosomal	gi|512899137|ref|XP_0049	BGIBMGA004998-PA	6	6	101.55	16.492	0	1658680	0	2558063	∞	0.35592	∞	0.02132	Yes
165	enzyme	cathepsin l	gi|512905760|ref|XP_0049	BGIBMGA009139-PA	8	8	62.689	44.291	0	1.2E+07	0	1980581	∞	0.06134	∞	0.18857	
88	enzyme	serine protease-like protein	—	BGIBMGA006406-PA	3	2	101.99	11.852	0	0	0	1323822	—	#DIV/0!	∞	0.0079	Yes
190	enzyme	alpha-mannosidase2-	gi|512934954|ref|XP_0049	BGIBMGA012267-PA	17	17	117.91	143.21	0	539825	0	1.3E+07	∞	0.14088	∞	0.01592	Yes
213	extracellular matrix	glypican-1-like	gi|512893132|ref|XP_0049	BGIBMGA003304-PA	2	2	71.332	11.639	0	0	0	1645729	—	#DIV/0!	∞	0.17714	
109	enzyme	peroxidase-like	gi|512924512|ref|XP_0049	BGIBMGA006518-PA	4	4	84.452	11.422	0	351783	0	1207963	∞	0.35592	∞	0.14342	
76	enzyme	uncharacterized family 31	gi|512901183|ref|XP_0049	BGIBMGA007153-PA	3	2	70.63	9.9901	0	0	0	1639530	—	#DIV/0!	∞	0.18398	
257	immune-non-serpin type	serine protease inhibitor TIL	gi|512903757|ref|XP_0049	—	2	2	66.272	4.4517	0	0	0	2063923	—	#DIV/0!	∞	0.08196	
216	extracellular matrix	fibrillin-2-like	gi|512894789|ref|XP_0049	BGIBMGA004571-PA	2	2	127.9	4.694	0	0	0	316601	—	#DIV/0!	∞	0.25751	
385	unknown protein	hypothetical protein	gi|512925174|ref|XP_0049	BGIBMGA011192-PA	3	3	57.535	14.118	0	0	0	183878	—	#DIV/0!	∞	0.35592	
344	unknown protein	uncharacterized protein	gi|512903769|ref|XP_0049	BGIBMGA006234-PA	2	2	246.79	5.7842	0	0	0	289412	—	#DIV/0!	∞	0.16006	
229	other	IGF-like	gi|223890162|ref|NP_0011	BGIBMGA012305-PA	3	3	9.5599	88.641	0	0	2E+08	0	—	#DIV/0!	0	6.8E-06	
330	other	gustatory receptor candidate 59	gi|512931731|ref|XP_0049	BGIBMGA002603-PA	2	2	8.8338	85.038	0	0	4.5E+07	0	—	#DIV/0!	0	6.8E-05	
247	immune-effector	Cecropin-D GN=CECD	gi|46395627|sp|O76146.1|	BGIBMGA000017-PA	2	2	6.4355	24.2	0	0	4.3E+07	0	—	#DIV/0!	0	0.10283	
35	binding and transport	chemosensory protein4	gi|115551740|dbj|BAF343	BGIBMGA004065-PA	4	4	14.257	39.767	0	0	4.2E+07	0	—	#DIV/0!	0	0.00045	
246	immune-effector	gloverin1	gi|52421205|dbj|BAD5147	BGIBMGA013863-PA	7	6	19.122	20.96	0	0	1.5E+07	0	—	#DIV/0!	0	0.00028	
250	immune-non-serpin type	serine protease inhibitor WAP	gi|512892340|ref|XP_0049	BGIBMGA008016-PA	3	3	14.652	21.225	0	0	0	1.7E+07	—	#DIV/0!	∞	0.16198	
45	binding and transport	Acyl-CoA binding protein	gi|8575541|gb|AAF78043.	BGIBMGA002905-PA	3	3	9.6877	4.0656	0	0	1.2E+07	0	—	#DIV/0!	0	0.0502	

371	unknown protein	Uncharacterized protein	gi|512910345|ref|XP_0049	BGIBMGA001328-PA	3	3	51.014	29.762	0	1.7E+07	0	0	∞	0.10348	—	#DIV/0!	
352	unknown protein	uncharacterized protein	gi|512898481|ref|XP_0049	BGIBMGA009063-PA	2	2	13.13	4.3794	0	0	0	1.1E+07	—	#DIV/0!	∞	0.0862	
348	unknown protein	uncharacterized protein	gi|512903713|ref|XP_0049	—	4	4	30.441	10.709	0	4426383	0	7613408	∞	0.22573	∞	0.02637	Yes
388	enzyme	short chain dehydrogenase	—	BGIBMGA005829-PA	4	2	15.575	27.477	0	0	0	1E+07	—	#DIV/0!	∞	0.25031	
341	unknown protein	uncharacterized protein	gi|512924445|ref|XP_0049	BGIBMGA006371-PA	7	7	81.8	53.196	0	0	6445401	0	—	#DIV/0!	0	0.00113	
2	binding and transport	yellow-fb	gi|86450723|gb|ABC9669	—	7	7	47.125	33.967	0	3249098	0	6449498	∞	0.16075	∞	0.03883	Yes
336	unknown protein	uncharacterized protein	gi|148298789|ref|NP_0010	BGIBMGA005301-PA	3	3	26.803	19.756	0	0	6105139	0	—	#DIV/0!	0	0.02561	
69	other	beta-tubulin	gi|3399724|dbj|BAA32102	BGIBMGA009131-PA	5	5	45.541	14.185	0	0	4250743	0	—	#DIV/0!	0	0.00185	
131	enzyme	hydrolase	gi|512932225|ref|XP_0049	BGIBMGA007025-PA	5	5	32.292	8.6583	0	0	0	5155191	—	#DIV/0!	∞	0.35592	
127	enzyme	Isocitrate dehydrogenase	gi|87248167|gb|ABD3613	BGIBMGA006907-PA	6	5	46.176	21.194	0	0	4428270	0	—	#DIV/0!	0	0.00364	
67	other	myofilin	gi|290561715|ref|NP_0010	—	2	2	13.538	5.2429	0	0	0	5565451	—	#DIV/0!	∞	0.02497	Yes
355	unknown protein	uncharacterized protein	gi|512913586|ref|XP_0049	—	2	2	26.482	5.4542	0	0	0	4162082	—	#DIV/0!	∞	0.17337	
63	other	tubulin alpha-1chain-like	gi|512933487|ref|XP_0049	BGIBMGA002542-PA	3	3	49.948	6.1598	0	0	3582332	0	—	#DIV/0!	0	0.22605	
207	extracellular matrix	proteoglycan 4-like	gi|512892210|ref|XP_0049	BGIBMGA009443-PA	5	5	77.621	7.068	0	0	2857379	0	—	#DIV/0!	0	0.00077	
306	immune-signaling	serine protease BmSP72/CLIP18	gi|512894586|ref|XP_0049	BGIBMGA013797-PA	3	3	45.587	6.6139	0	0	0	3307334	—	#DIV/0!	∞	0.05769	
176	enzyme	Beta-hexosaminidase	gi|51243503|gb|AAT9945	BGIBMGA014115-PA	27	4	61.55	13.59	0	0	0	3276625	—	#DIV/0!	∞	0.03527	Yes
64	other	Profilin	gi|56404766|sp|Q68HB4.1	BGIBMGA002981-PA	2	2	13.713	4.4166	0	0	1629877	0	—	#DIV/0!	0	0.18585	
21	binding and transport	ML-domain containing	gi|95102958|gb|ABF5142	—	3	3	16.972	5.3201	0	0	2473673	0	—	#DIV/0!	0	0.13083	
91	enzyme	serine protease 7	gi|95102988|gb|ABF5143	BGIBMGA012427-PA	2	2	44.088	30.147	0	0	0	2610205	—	#DIV/0!	∞	0.35592	
337	unknown protein	uncharacterized protein	gi|512936588|ref|XP_0049	BGIBMGA013986-PA	7	7	59.453	44.175	0	886982	0	1726451	∞	0.35592	∞	0.15498	
167	enzyme	carboxypeptidase D-like	gi|512917758|ref|XP_0049	BGIBMGA012806-PA	6	6	156.09	26.607	0	0	0	2220771	—	#DIV/0!	∞	0.06404	
10	binding and transport	scavenger receptor	gi|512917690|ref|XP_0049	BGIBMGA013041-PA	4	4	30.68	10.844	0	0	1045410	0	—	#DIV/0!	0	0.22489	
342	unknown protein	uncharacterized protein	gi|512915164|ref|XP_0049	—	7	2	87.459	21.083	0	1305833	0	1101993	∞	0.35592	∞	0.13409	
363	unknown protein	uncharacterized protein	—	BGIBMGA002596-PA	3	3	141.48	46.746	0	0	0	1918380	—	#DIV/0!	∞	0.07092	
151	enzyme	Enolase	gi|158451613|gb|ABW391	BGIBMGA005493-PA	3	3	40.779	13.139	0	0	1331384	0	—	#DIV/0!	0	1.5E-05	

73	enzyme	venom dipeptidyl peptidase 4-like	gi|512914285|ref|XP_0049	BGIBMGA001272-PA	5	5	80.888	8.4389	0	0	288079	1227320	—	#DIV/0!	4.260358	0.47728	
22	binding and transport	microvitellogenin-like	gi|512937862|ref|XP_0049	BGIBMGA014097-PA	2	2	32.072	14.275	0	0	0	1280867	—	#DIV/0!	∞	0.35592	
191	enzyme	alpha-mannosidase2-	gi|512887311|ref|XP_0049	BGIBMGA005142-PA	4	4	133.62	14.021	0	0	0	1306711	—	#DIV/0!	∞	0.04742	Yes
322	other	putative epidermal cell	gi|512887229|ref|XP_0049	BGIBMGA005105-PA	2	2	119.78	25.4	0	0	0	1311816	—	#DIV/0!	∞	0.14249	
153	enzyme	Ecdysone oxidase GN=LOC100302	gi|300360466|ref|NP_0011	BGIBMGA000158-PA	3	3	61.805	5.7745	0	0	0	1283397	—	#DIV/0!	∞	0.35592	
108	enzyme	peroxidase-like	gi|512905378|ref|XP_0049	BGIBMGA012740-PA	2	2	78.238	8.8944	0	0	482947	297023	—	#DIV/0!	0.615022	0.66456	
364	unknown protein	Uncharacterized protein	gi|512893969|ref|XP_0049	BGIBMGA010036-PA	4	2	31.886	13.695	0	0	0	1017913	—	#DIV/0!	∞	0.35592	
227	other	multiple epidermal growth	gi|512901872|ref|XP_0049	BGIBMGA012538-PA	3	3	68.131	7.6123	0	0	0	1020402	—	#DIV/0!	∞	0.02619	Yes
220	extracellular matrix	chitin binding protein	gi|512916360|ref|XP_0049	BGIBMGA007899-PA	4	4	26.419	8.2567	0	0	211117	0	—	#DIV/0!	0	0.35592	
293	immune-signaling	serpin-2	gi|7341330|gb|AAF61252.	BGIBMGA007720-PA	2	2	38.699	3.9194	0	0	950170	0	—	#DIV/0!	0	0.35592	
295	immune-signaling	serpin-14	gi|226342888|ref|NP_0011	BGIBMGA013958-PA	2	2	45.778	5.2642	0	0	0	860993	—	#DIV/0!	∞	0.35592	
94	enzyme	ribonuclease T2	gi|512919647|ref|XP_0049	BGIBMGA010520-PA	2	2	22.062	3.0647	0	0	0	774914	—	#DIV/0!	∞	0.35592	
118	enzyme	matrixmetalloproteinase	gi|172356221|ref|NP_0011	BGIBMGA007804-PA	2	2	57.993	8.5311	0	0	533005	253083	—	#DIV/0!	0.474823	0.65198	
178	enzyme	beta-glucosidase precursor	gi|512906269|ref|XP_0049	BGIBMGA014178-PA	3	3	57.807	15.51	0	0	0	725272	—	#DIV/0!	∞	0.35592	
186	enzyme	Aminopeptidase N-8	gi|525343809|ref|NP_0012	BGIBMGA008066-PA	5	5	120.31	13.781	0	0	0	668077	—	#DIV/0!	∞	0.35592	
182	enzyme	arylsulfataseB-like	gi|512897002|ref|XP_0049	BGIBMGA012511-PA	3	2	60.177	3.2205	0	0	0	523633	—	#DIV/0!	∞	0.35592	
297	immune-signaling	serpin-12	gi|13359088|dbj|BAB3329	BGIBMGA010216-PA	3	3	42.863	12.226	0	0	0	554441	—	#DIV/0!	∞	0.35592	
383	unknown protein	Uncharacterized protein	gi|148298740|ref|NP_0010	BGIBMGA007655-PA	3	3	37.069	15.407	0	0	462459	0	—	#DIV/0!	0	0.35592	
166	enzyme	Catalase GN=Cat	gi|51571867|dbj|BAD3885	BGIBMGA000701-PA	2	2	56.898	3.4241	0	0	179696	0	—	#DIV/0!	0	0.35592	
173	enzyme	carbamoyl-phosphate	gi|512900327|ref|XP_0049	BGIBMGA006816-PA	2	2	245.81	3.2822	0	0	28154.5	0	—	#DIV/0!	0	0.35592	
