# Supplementary material for: Identification of N-linked Glycoproteins in Silkworm Serum Using Con A Lectin Affinity Chromatography and Mass Spectrometry
Source: J Insect Sci. 2021 Aug 17;21(4):14. doi: 10.1093/jisesa/ieab057 (PMC8367846; doi:10.1093/jisesa/ieab057)
Supplement: ieab057_suppl_Supplementary_Table_S3 [file ieab057_suppl_supplementary_table_s3.pdf]

Supplementary Table S3. List of N-linked glycoproteins by classification, annotated name, silkDB ID, Genebank ID, glycopeptides evidence. V7 L, lectin-enriched serum proteins from day 7 of the larval fifth instar; P2 L, lectin-enriched serum proteins from day 2 of the pupae.

Classification	Annotated name	Genebank IDs	silkDB IDs	glycopeptides evidence of V7 L-1	glycopeptides evidence of V7 L-2	glycopeptides evidence of V7 L-3	glycopeptides evidence of V7 L-4	glycopeptides evidence of P2 L-1	glycopeptides evidence of P2 L-2	glycopeptides evidence of P2 L-3	glycopeptides evidence of P2 L-4	Con A Lectin-enriched protein
unknown protein	Uncharacterized protein GN=LOC101742613	gi|512937718|ref|XP_004933833.1|P	BGIBMGA014203-PApe	+	+	+	+	+	+	+	+	Yes
unknown protein	Uncharacterized protein GN=LOC101742472	gi|512937706|ref|XP_004933830.1|P	BGIBMGA014204-PApe	+	+	+	+	+	+	+	+	Yes
unknown protein	Uncharacterized protein GN=LOC101738062	gi|512937361|ref|XP_004933749.1|P	BGIBMGA014512-PApe	-	-	-	-	-	-	-	+	Yes
unknown protein	uncharacterized protein LOC101739791	gi|512919035|ref|XP_004929277.1|P	BGIBMGA005938-PApe	-	-	-	-	-	+	-	+	Yes
unknown protein	uncharacterized protein LOC101743739	gi|512916157|ref|XP_004928564.1|P	—	-	-	-	-	+	-	+	+	Yes
unknown protein	uncharacterized protein LOC101737542	gi|512911073|ref|XP_004927326.1|P	BGIBMGA009180-PApe	-	+	-	-	-	+	+	+	Yes
unknown protein	Uncharacterized protein GN=LOC101737253	gi|512909709|ref|XP_004926988.1|P	BGIBMGA012645-PApe	-	-	-	-	+	-	+	+	Yes
unknown protein	uncharacterized protein LOC101735373	gi|512902459|ref|XP_004925381.1|P	BGIBMGA014264-PApe	+	+	+	+	+	+	+	+	Yes
unknown protein	Uncharacterized protein GN=LOC101740356	gi|512893824|ref|XP_004923275.1|P	BGIBMGA010044-PApe	+	+	+	+	-	-	-	+	Yes
unknown protein	Uncharacterized protein GN=LOC101740222	gi|512893821|ref|XP_004923274.1|P	BGIBMGA010043-PApe	+	-	-	-	+	+	-	+	Yes
unknown protein	Uncharacterized protein GN=LOC101740086	gi|512893817|ref|XP_004923273.1|P	BGIBMGA010040-PApe	+	-	+	+	-	+	-	+	Yes
unknown protein	uncharacterized protein LOC101739948	gi|512893813|ref|XP_004923272.1|P	BGIBMGA010041-PApe	-	-	-	-	-	+	-	-	Yes
unknown protein	Uncharacterized protein	gi|148298740|ref|NP_001091783.1|u	BGIBMGA007655-PApe	+	-	-	-	-	-	-	-	
unknown protein	uncharacterized protein LOC105842972	—	BGIBMGA014513-PA p	-	-	-	-	-	-	-	+	
unknown protein	uncharacterized protein LOC105842972	-	BGIBMGA010047-PA p	-	-	-	-	+	+	+	-	
other	fibroin p25 like	gi|512891154|ref|XP_004922635.1|P	BGIBMGA009259-PApe	-	-	-	-	+	-	+	-	
other	putative epidermal cell surface receptor-like	gi|512887229|ref|XP_004921888.1|P	BGIBMGA005105-PApe	-	-	-	-	-	-	-	+	
other	low density lipoprotein receptor	gi|226531274|ref|NP_001140194.1|l	BGIBMGA005876-PApe	-	-	-	-	+	-	+	+	Yes
other	very low-density lipoprotein receptor	—	BGIBMGA006214-PA p	-	-	-	-	-	-	+	-	

<table>
<tr><td>immune-signaling</td><td>serine protease CLIP7/12</td><td>gi|512909603|ref|XP_004926962.1|P</td><td>BGIBMGA010306-PAp</td><td>-</td><td>+</td><td>-</td><td>+</td><td>+</td><td>+</td><td>+</td><td>+</td><td>Yes</td></tr>
<tr><td>immune-signaling</td><td>hemolymph protein14; MBL-associated serine proteases</td><td>gi|389620198|gb|AFK93534.1|hemol</td><td>BGIBMGA012217-PAp</td><td>+</td><td>-</td><td>+</td><td>-</td><td>+</td><td>+</td><td>+</td><td>+</td><td>Yes</td></tr>
<tr><td>immune-signaling</td><td>serpin-9</td><td>gi|14028769|gb|AAK52495.1|AF361</td><td>BGIBMGA001983-PAp</td><td>-</td><td>-</td><td>-</td><td>-</td><td>-</td><td>-</td><td>+</td><td>+</td><td></td></tr>
<tr><td>immune-signaling</td><td>serpin-13</td><td>—</td><td>BGIBMGA002368-PA</td><td>-</td><td>-</td><td>-</td><td>-</td><td>+</td><td>+</td><td>+</td><td>+</td><td></td></tr>
<tr><td>immune-recognition</td><td>immunoglobulin superfamily</td><td>gi|512925142|ref|XP_004930766.1|P</td><td>BGIBMGA011387-PAp</td><td>-</td><td>-</td><td>-</td><td>-</td><td>+</td><td>+</td><td>+</td><td>-</td><td>Yes</td></tr>
<tr><td>immune-recognition</td><td>immunoglobulin superfamily</td><td>gi|512895990|ref|XP_004923806.1|P</td><td>BGIBMGA009336-PAp</td><td>-</td><td>-</td><td>-</td><td>-</td><td>+</td><td>-</td><td>+</td><td>+</td><td>Yes</td></tr>
<tr><td>immune-recognition</td><td>immunoglobulin superfamily</td><td>gi|512895113|ref|XP_004923595.1|P</td><td>BGIBMGA006339-PAp</td><td>-</td><td>-</td><td>-</td><td>-</td><td>-</td><td>+</td><td>-</td><td>-</td><td>Yes</td></tr>
<tr><td>immune-recognition</td><td>C-type lectin 11</td><td>gi|31559111|gb|AAP50846.1|immule</td><td>BGIBMGA006623-PAp</td><td>-</td><td>-</td><td>-</td><td>-</td><td>-</td><td>+</td><td>-</td><td>+</td><td>Yes</td></tr>
<tr><td>immune-recognition</td><td>immunoglobulin superfamily</td><td>gi|258642521|gb|ACQ82817.2|hemo</td><td>BGIBMGA008736-PAp</td><td>-</td><td>-</td><td>-</td><td>-</td><td>+</td><td>+</td><td>+</td><td>+</td><td>Yes</td></tr>
<tr><td>immune-recognition</td><td>C-type lectin 10</td><td>gi|148298818|ref|NP_001091784.1|C</td><td>BGIBMGA006768-PAp</td><td>+</td><td>+</td><td>+</td><td>+</td><td>+</td><td>+</td><td>-</td><td>+</td><td>Yes</td></tr>
<tr><td>immune-recognition</td><td>beta-1,3-glucan recognition protein 2</td><td>—</td><td>BGIBMGA011609-PAp</td><td>-</td><td>-</td><td>-</td><td>-</td><td>-</td><td>+</td><td>-</td><td>+</td><td>Yes</td></tr>
<tr><td>immune-other</td><td>Putative defense protein Reeler</td><td>gi|325660637|gb|ADZ40416.1|Immu</td><td>BGIBMGA014360-PAp</td><td>-</td><td>-</td><td>-</td><td>-</td><td>+</td><td>-</td><td>+</td><td>+</td><td>Yes</td></tr>
<tr><td>immune-other</td><td>Bm8 interacting protein 2d-4</td><td>gi|306518642|ref|NP_001182383.1|B</td><td>BGIBMGA012968-PAp</td><td>-</td><td>-</td><td>-</td><td>-</td><td>+</td><td>+</td><td>+</td><td>+</td><td>Yes</td></tr>
<tr><td>immune-non-serpin type</td><td>serine protease inhibitor kunitz BmSPI55</td><td>gi|512912752|ref|XP_004927733.1|P</td><td>BGIBMGA001091-PAp</td><td>-</td><td>-</td><td>-</td><td>-</td><td>-</td><td>-</td><td>-</td><td>+</td><td>Yes</td></tr>
<tr><td>immune-non-serpin type</td><td>serine protease inhibitor ITI BmSPI70</td><td>gi|512899354|ref|XP_004924623.1|P</td><td>BGIBMGA007558-PAp</td><td>+</td><td>+</td><td>+</td><td>+</td><td>+</td><td>+</td><td>+</td><td>+</td><td>Yes</td></tr>
<tr><td>immune-non-serpin type</td><td>serine protease inhibitor TIL BmSPI49</td><td>gi|512898143|ref|XP_004924327.1|P</td><td>BGIBMGA010889-PAp</td><td>-</td><td>-</td><td>-</td><td>-</td><td>+</td><td>+</td><td>+</td><td>+</td><td>Yes</td></tr>
<tr><td>immune-non-serpin type</td><td>serine protease inhibitor kunitz BmSPI58</td><td>gi|512886752|ref|XP_004921811.1|P</td><td>BGIBMGA005129-PAp</td><td>-</td><td>-</td><td>-</td><td>-</td><td>-</td><td>-</td><td>+</td><td>-</td><td>Yes</td></tr>
<tr><td>immune-effector</td><td>prophenoloxidase activating enzyme</td><td>gi|4521258|dbj|BAA76308.1|propher</td><td>—</td><td>-</td><td>-</td><td>-</td><td>-</td><td>-</td><td>-</td><td>+</td><td>-</td><td>Yes</td></tr>
<tr><td>extracellular matrix</td><td>thrombospondin</td><td>gi|512936253|ref|XP_004933473.1|P</td><td>BGIBMGA001836-PAp</td><td>-</td><td>-</td><td>-</td><td>-</td><td>-</td><td>-</td><td>+</td><td>-</td><td>Yes</td></tr>
<tr><td>extracellular matrix</td><td>basement membrane-specific heparan sulfate proteoglycan</td><td>gi|512935237|ref|XP_004933225.1|P</td><td>BGIBMGA012239-PAp</td><td>-</td><td>-</td><td>-</td><td>-</td><td>-</td><td>+</td><td>+</td><td>-</td><td>Yes</td></tr>
<tr><td>extracellular matrix</td><td>connectin-like</td><td>gi|512915242|ref|XP_004928341.1|P</td><td>BGIBMGA008307-PAp</td><td>-</td><td>-</td><td>-</td><td>-</td><td>-</td><td>+</td><td>+</td><td>+</td><td>Yes</td></tr>
<tr><td>extracellular matrix</td><td>adhesion related transmembrane</td><td>gi|512895721|ref|XP_004923739.1|P</td><td>BGIBMGA008135-PAp</td><td>+</td><td>+</td><td>+</td><td>+</td><td>+</td><td>+</td><td>+</td><td>+</td><td>Yes</td></tr>
<tr><td>extracellular matrix</td><td>fibrillin-like</td><td>gi|17298113|dbj|BAB78525.1|fibrilli</td><td>—</td><td>+</td><td>+</td><td>+</td><td>+</td><td>+</td><td>+</td><td>+</td><td>+</td><td>Yes</td></tr>
</table>

<table>
<tr><td>enzyme</td><td>ecdysteroid-inducible angiotensin-converting</td><td>gi|8918492|dbj|BAA97657.1|BmAce</td><td>BGIBMGA002526-PAp</td><td>+</td><td>+</td><td>+</td><td>+</td><td>+</td><td>+</td><td>+</td><td>+</td><td>Yes</td></tr>
<tr><td>enzyme</td><td>antennal esterase cxe11</td><td>gi|87248285|gb|ABD36195.1|carbox</td><td>BGIBMGA012031-PAp</td><td>+</td><td>+</td><td>+</td><td>+</td><td>+</td><td>+</td><td>+</td><td>+</td><td>Yes</td></tr>
<tr><td>enzyme</td><td>Superoxide dismutase [Cu-Zn] GN=ea4</td><td>gi|68144076|gb|AAY86076.1|diapau</td><td>BGIBMGA002907-PAp</td><td>+</td><td>-</td><td>-</td><td>-</td><td>+</td><td>+</td><td>-</td><td>+</td><td>Yes</td></tr>
<tr><td>enzyme</td><td>aldo-keto reductase</td><td>gi|512935651|ref|XP_004933326.1|P</td><td>BGIBMGA009801-PAp</td><td>-</td><td>-</td><td>-</td><td>-</td><td>+</td><td>-</td><td>+</td><td>+</td><td>Yes</td></tr>
<tr><td>enzyme</td><td>ubiquitin-protein ligase</td><td>gi|512927111|ref|XP_004931253.1|P</td><td>BGIBMGA002518-PAp</td><td>-</td><td>-</td><td>-</td><td>-</td><td>+</td><td>+</td><td>+</td><td>+</td><td>Yes</td></tr>
<tr><td>enzyme</td><td>alpha-1,2-Mannosidase GN=LOC101744477</td><td>gi|512926654|ref|XP_004931140.1|P</td><td>BGIBMGA002426-PAp</td><td>-</td><td>-</td><td>-</td><td>-</td><td>-</td><td>+</td><td>-</td><td>+</td><td>Yes</td></tr>
<tr><td>enzyme</td><td>gamma-glutamylcyclotransferase-like</td><td>gi|512923155|ref|XP_004930284.1|P</td><td>—</td><td>-</td><td>-</td><td>+</td><td>-</td><td>+</td><td>+</td><td>+</td><td>+</td><td></td></tr>
<tr><td>enzyme</td><td>subunit of dna dependent rna-polymerase</td><td>gi|512919003|ref|XP_004929269.1|P</td><td>BGIBMGA005942-PAp</td><td>-</td><td>-</td><td>-</td><td>-</td><td>-</td><td>-</td><td>+</td><td>+</td><td>Yes</td></tr>
<tr><td>enzyme</td><td>ser thr protein phosphatase nucleotidase</td><td>gi|512918105|ref|XP_004929050.1|P</td><td>BGIBMGA012947-PAp</td><td>-</td><td>+</td><td>-</td><td>-</td><td>-</td><td>-</td><td>-</td><td>-</td><td></td></tr>
<tr><td>enzyme</td><td>serine protease BmSP53</td><td>gi|512917698|ref|XP_004928951.1|P</td><td>BGIBMGA013049-PAp</td><td>-</td><td>-</td><td>+</td><td>-</td><td>+</td><td>+</td><td>+</td><td>+</td><td>Yes</td></tr>
<tr><td>enzyme</td><td>adenosinedeaminase AGSA-like</td><td>gi|512917269|ref|XP_004928846.1|P</td><td>BGIBMGA012938-PAp</td><td>-</td><td>-</td><td>-</td><td>-</td><td>-</td><td>-</td><td>-</td><td>+</td><td>Yes</td></tr>
<tr><td>enzyme</td><td>sulfuric ester hydrolase</td><td>gi|512913875|ref|XP_004928000.1|P</td><td>BGIBMGA001098-PAp</td><td>-</td><td>-</td><td>-</td><td>-</td><td>+</td><td>+</td><td>+</td><td>+</td><td>Yes</td></tr>
<tr><td>enzyme</td><td>plasma glutamate carboxypeptidase-like</td><td>gi|512913737|ref|XP_004927967.1|P</td><td>BGIBMGA001275-PAp</td><td>-</td><td>-</td><td>-</td><td>-</td><td>-</td><td>+</td><td>-</td><td>-</td><td>Yes</td></tr>
<tr><td>enzyme</td><td>ADP-dependent NAD(P)H-hydrate dehydratase</td><td>gi|512909717|ref|XP_004926990.1|P</td><td>BGIBMGA012642-PAp</td><td>+</td><td>-</td><td>+</td><td>+</td><td>+</td><td>+</td><td>+</td><td>+</td><td>Yes</td></tr>
<tr><td>enzyme</td><td>seminal fluid protein hacp058/aldo-keto reductase</td><td>gi|512908815|ref|XP_004926771.1|P</td><td>BGIBMGA001351-PAp</td><td>+</td><td>+</td><td>+</td><td>+</td><td>+</td><td>+</td><td>+</td><td>+</td><td>Yes</td></tr>
<tr><td>enzyme</td><td>serine-type endopeptidase</td><td>gi|512908708|ref|XP_004926747.1|P</td><td>BGIBMGA002365-PAp</td><td>-</td><td>-</td><td>-</td><td>-</td><td>-</td><td>-</td><td>-</td><td>+</td><td></td></tr>
<tr><td>enzyme</td><td>carboxylic ester hydrolase</td><td>gi|512908407|ref|XP_004926674.1|P</td><td>BGIBMGA003917-PAp</td><td>+</td><td>+</td><td>+</td><td>+</td><td>-</td><td>+</td><td>+</td><td>+</td><td>Yes</td></tr>
<tr><td>enzyme</td><td>transmembrane protease serine 2-like</td><td>gi|512903587|ref|XP_004925656.1|P</td><td>—</td><td>-</td><td>-</td><td>-</td><td>-</td><td>-</td><td>-</td><td>-</td><td>+</td><td>Yes</td></tr>
<tr><td>enzyme</td><td>inosine-uridine preferring nucleoside hydrolase</td><td>gi|512893076|ref|XP_004923091.1|P</td><td>BGIBMGA003331-PAp</td><td>-</td><td>-</td><td>-</td><td>-</td><td>+</td><td>+</td><td>-</td><td>+</td><td>Yes</td></tr>
<tr><td>enzyme</td><td>alpha-mannosidase2-like</td><td>gi|512887311|ref|XP_004921902.1|P</td><td>BGIBMGA005142-PAp</td><td>-</td><td>-</td><td>-</td><td>-</td><td>-</td><td>+</td><td>-</td><td>-</td><td>Yes</td></tr>
<tr><td>enzyme</td><td>glycerophosphodiester phosphodiesterase</td><td>gi|32997080|dbj|BAC79386.1|glycer</td><td>BGIBMGA007767-PAp</td><td>-</td><td>-</td><td>-</td><td>-</td><td>+</td><td>-</td><td>-</td><td>+</td><td>Yes</td></tr>
<tr><td>enzyme</td><td>cathepsin D</td><td>gi|31559113|gb|AAP50847.1| cathep</td><td>—</td><td>+</td><td>-</td><td>-</td><td>-</td><td>-</td><td>-</td><td>-</td><td>-</td><td></td></tr>
<tr><td>enzyme</td><td>Antennal esterase</td><td>gi|298566244|ref|NP_001177297.1|a</td><td>BGIBMGA004229-PAp</td><td>-</td><td>-</td><td>-</td><td>-</td><td>+</td><td>+</td><td>+</td><td>+</td><td>Yes</td></tr>
</table>

enzyme	Beta-galactosidase GN=gal	gi|255652865|ref|NP_001157373.1|b	BGIBMGA006815-PAp	+	-	-	+	+	+	+	+	Yes
enzyme	juvenile hormone esterase	gi|18001001|gb|AAL55240.1|AF287	BGIBMGA000772-PAp	+	+	+	+	+	+	+	+	Yes
enzyme	Carboxylic ester hydrolase	gi|168823411|ref|NP_001108339.1|i	BGIBMGA013812-PAp	-	-	-	-	+	-	+	+	Yes
enzyme	Beta-N-acetylglucosaminidase 1	gi|145651816|ref|NP_001078833.1|b	BGIBMGA011646-PAp	+	+	+	+	+	+	+	+	Yes
enzyme	Beta-hexosaminidase GN=GlcNAcase3	—	BGIBMGA014116-PAp	-	-	-	-	+	+	+	+	Yes
binding and transport	Ferritin	gi|95102694|gb|ABF51285.1|ferritin	BGIBMGA008768-PAp	-	-	-	-	+	-	+	+	Yes
binding and transport	yellow-d	gi|86450717|gb|ABC96694.1|yellow-	BGIBMGA007254-PAp	-	-	-	-	-	-	-	+	Yes
binding and transport	27 kDa glycoprotein PE=1 SV=1	gi|74843747|sp|Q8T113.1|P27K_BO	BGIBMGA000462-PAp	-	-	-	-	-	-	-	+	
binding and transport	30K protein BmLP17	gi|525343846|ref|NP_001266375.1|l	BGIBMGA004398-PAp	-	-	-	-	-	+	-	-	
binding and transport	zinc transporter foi-like	gi|512939606|ref|XP_004934282.1|P	BGIBMGA009645-PAp	-	-	-	-	-	-	-	+	Yes
binding and transport	pheromone binding protein / odorant binding protein	gi|512933090|ref|XP_004932705.1|P	BGIBMGA011432-PAp	-	-	-	-	-	+	-	-	
binding and transport	insecticyanin-A-like; Lipocalin	gi|512894208|ref|XP_004923377.1|P	BGIBMGA004805-PAp	-	-	-	-	+	+	+	+	Yes
binding and transport	30K protein 26	gi|379046526|gb|AFC87824.1| 30K p	—	+	+	+	+	+	+	+	-	
binding and transport	30K protein BmLP7	gi|1587670|prf||2207200AVAPpeptic	BGIBMGA004397-PAp	-	-	+	+	-	-	+	-	
binding and transport	apolipophorin-III	gi|1381798|gb|AAB02853.1|apolipop	BGIBMGA013108-PAp	-	-	-	-	-	+	-	+	
binding and transport	Transferrin GN=Tf	—	BGIBMGA011424-PAp	-	+	-	-	+	+	+	+	Yes
binding and transport	SP1	gi|1335609|emb|CAA31417.1| SP1	—	+	+	+	+	+	+	+	+	Yes
