# Supplementary material for: Identification of N-linked Glycoproteins in Silkworm Serum Using Con A Lectin Affinity Chromatography and Mass Spectrometry
Source: J Insect Sci. 2021 Aug 17;21(4):14. doi: 10.1093/jisesa/ieab057 (PMC8367846; doi:10.1093/jisesa/ieab057)
Supplement: ieab057_suppl_Supplementary_Table_S4 [file ieab057_suppl_supplementary_table_s4.pdf]

Supplementary Table S4. List of N-linked glycopeptides by sequence, modification type, protein IDs and names.

Glycopeptides	Modification Types	Protein IDs and names	MS/MS spectrum No.
K.LAN[+876.322]TSIER.S	HexNAc(2)Hex(2)Fuc(1)	gi|512939606|ref|XP_004934282.1| retinol dehydrogenase 11-like	1
K.C[+57.021]VC[+57.021]NTGWTGN[+730.264]GTVC[+57.021]GLDRDLDGHPDEQLPC[+57.021]NEPR.C	HexNAc(2)Hex(2)	gi|512936253|ref|XP_004933473.1| LOW QUALITY PROTEIN: cartilage	2
K.SIGISNFN[+1378.476]TTQIDR.I	HexNAc(2)Hex(6)	gi|512935651|ref|XP_004933326.1| LOW QUALITY PROTEIN: aldo-keto	3
K.SIGISNFN[+1216.423]TTQIDR.I	HexNAc(2)Hex(5)	gi|512935651|ref|XP_004933326.1| LOW QUALITY PROTEIN: aldo-keto	4
R.DSIQFQN[+876.322]LSK.S	HexNAc(2)Hex(2)Fuc(1)	gi|512935237|ref|XP_004933225.1| uncharacterized protein LOC101745012	5
R.SLQSNQIEAGN[+876.322]SSSYSGALDSQSSVEK.Q	HexNAc(2)Hex(2)Fuc(1)	gi|512926654|ref|XP_004931140.1| major facilitator superfamily domain-	6
R.RPPIISDN[+876.322]STR.S	HexNAc(2)Hex(2)Fuc(1)	gi|512925142|ref|XP_004930766.1| lachesin-like	7
R.RPPIISDN[+1038.375]STR.S	HexNAc(2)Hex(3)Fuc(1)	gi|512925142|ref|XP_004930766.1| lachesin-like	8
R.N[+876.322]VTVTTPGGK.Q	HexNAc(2)Hex(2)Fuc(1)	gi|512923155|ref|XP_004930284.1| gamma-glutamylcyclotransferase-like	9
R.N[+730.264]VTVTTPGGK.Q	HexNAc(2)Hex(2)	gi|512923155|ref|XP_004930284.1| gamma-glutamylcyclotransferase-like	10
R.N[+714.269]VTVTTPGGK.Q	HexNAc(2)Hex(1)Fuc(1)	gi|512923155|ref|XP_004930284.1| gamma-glutamylcyclotransferase-like	11
R.N[+552.217]VTVTTPGGK.Q	HexNAc(2)Fuc(1)	gi|512923155|ref|XP_004930284.1| gamma-glutamylcyclotransferase-like	12
R.N[+406.159]VTVTTPGGK.Q	HexNAc(2)	gi|512923155|ref|XP_004930284.1| gamma-glutamylcyclotransferase-like	13
K.IVHDAENVPIGSSN[+876.322]K.T	HexNAc(2)Hex(2)Fuc(1)	gi|512918105|ref|XP_004929050.1| apyrase-like	14
R.FVSQIIIHPEYN[+876.322]ASTFSR.D	HexNAc(2)Hex(2)Fuc(1)	gi|512917698|ref|XP_004928951.1| serine protease gd-like	15
R.YIEC[+57.021]IN[+1038.375]GTAEEK.L	HexNAc(2)Hex(3)Fuc(1)	gi|512916157|ref|XP_004928564.1| uncharacterized protein LOC101743739	16
K.LVDEIQSYKN[+876.322]VTK.Q	HexNAc(2)Hex(2)Fuc(1)	gi|512913737|ref|XP_004927967.1| carboxypeptidase Q-like	17
K.AMVYEN[+876.322]ASR.W	HexNAc(2)Hex(2)Fuc(1)	gi|512912752|ref|XP_004927733.1| spondin-1-like	18
K.N[+876.322]ISIIQDYPK.A	HexNAc(2)Hex(2)Fuc(1)	gi|512909717|ref|XP_004926990.1| ATP-dependent (S)-NAD(P)H-hydrate	19
K.N[+1054.370]ISIIQDYPK.A	HexNAc(2)Hex(4)	gi|512909717|ref|XP_004926990.1| ATP-dependent (S)-NAD(P)H-hydrate	20
K.ASQN[+876.322]TTFVVVR.N	HexNAc(2)Hex(2)Fuc(1)	gi|512903587|ref|XP_004925656.1| transmembrane protease serine 2-like	21
R.SELFAN[+892.317]STSSNKDEILSLEPIIIFLTDGDPTVGEMNPK.T	HexNAc(2)Hex(3)	gi|512899354|ref|XP_004924623.1| inter-alpha-trypsin inhibitor heavy chain	22
R.SELFAN[+876.322]STSSNKDEILSLEPIIIFLTDGDPTVGEMNPK.T	HexNAc(2)Hex(2)Fuc(1)	gi|512899354|ref|XP_004924623.1| inter-alpha-trypsin inhibitor heavy chain	23
R.SELFAN[+730.264]STSSNKDEILSLEPIIIFLTDGDPTVGEMNPK.T	HexNAc(2)Hex(2)	gi|512899354|ref|XP_004924623.1| inter-alpha-trypsin inhibitor heavy chain	24
R.NRSELFAN[+876.322]STSSNKDEILSLEPIIIFLTDGDPTVGEMNPK.T	HexNAc(2)Hex(2)Fuc(1)	gi|512899354|ref|XP_004924623.1| inter-alpha-trypsin inhibitor heavy chain	25
R.N[+876.322]ITIDTYK.S	HexNAc(2)Hex(2)Fuc(1)	gi|512899354|ref|XP_004924623.1| inter-alpha-trypsin inhibitor heavy chain	26
R.N[+730.264]ITIDTYK.S	HexNAc(2)Hex(2)	gi|512899354|ref|XP_004924623.1| inter-alpha-trypsin inhibitor heavy chain	27
R.N[+1038.375]ITIDTYK.S	HexNAc(2)Hex(3)Fuc(1)	gi|512899354|ref|XP_004924623.1| inter-alpha-trypsin inhibitor heavy chain	28
K.YEFVTPLTSLVVVKPN[+876.322]ETDAVNAEPVGDR.E	HexNAc(2)Hex(2)Fuc(1)	gi|512899354|ref|XP_004924623.1| inter-alpha-trypsin inhibitor heavy chain	29
K.YEFVTPLTSLVVVKPN[+730.264]ETDAVNAEPVGDR.E	HexNAc(2)Hex(2)	gi|512899354|ref|XP_004924623.1| inter-alpha-trypsin inhibitor heavy chain	30
R.SKN[+876.322]GTC[+57.021]VSIDEC[+57.021]HR.E	HexNAc(2)Hex(2)Fuc(1)	gi|512898143|ref|XP_004924327.1| tenascin-like	31
R.SKN[+730.264]GTC[+57.021]VSIDEC[+57.021]HR.E	HexNAc(2)Hex(2)	gi|512898143|ref|XP_004924327.1| tenascin-like	32

R.AAN[+892.317]GTC[+57.021]IPTR.E	HexNAc(2)Hex(3)	gi|512898143|ref|XP_004924327.1| tenascin-like	33
R.AAN[+876.322]GTC[+57.021]IPTR.E	HexNAc(2)Hex(2)Fuc(1)	gi|512898143|ref|XP_004924327.1| tenascin-like	34
K.IGYLRDEN[+876.322]GTC[+57.021]IPQDK.C	HexNAc(2)Hex(2)Fuc(1)	gi|512898143|ref|XP_004924327.1| tenascin-like	35
K.VYIASDDGLNELN[+876.322]STDK.A	HexNAc(2)Hex(2)Fuc(1)	gi|512895721|ref|XP_004923739.1| uncharacterized protein LOC101739160	36
K.VYIASDDGLNELN[+730.264]STDK.A	HexNAc(2)Hex(2)	gi|512895721|ref|XP_004923739.1| uncharacterized protein LOC101739160	37
R.ETYVNVDGGN[+876.322]RTQK.S	HexNAc(2)Hex(2)Fuc(1)	gi|512894208|ref|XP_004923377.1| lopap-like	38
R.ETYVNVDGGN[+730.264]R.T	HexNAc(2)Hex(2)	gi|512894208|ref|XP_004923377.1| lopap-like	39
K.YC[+57.021]N[+730.264]FSR.E	HexNAc(2)Hex(2)	gi|512893076|ref|XP_004923091.1| uncharacterized protein LOC101743385	40
R.YLQFDN[+1038.375]GTVR.S	HexNAc(2)Hex(3)Fuc(1)	gi|4521258|dbj|BAA76308.1| prophenoloxidase activating enzyme	41
K.VAGWGLTSENGN[+876.322]ASQILK.V	HexNAc(2)Hex(2)Fuc(1)	gi|389620198|gb|AFK93534.1| hemolymph protein 14	42
K.VAGWGLTSEN[+0.984]GN[+730.264]ASQILK.V	HexNAc(2)Hex(2)	gi|389620198|gb|AFK93534.1| hemolymph protein 14	43
R.MVLN[+876.322]DTFIK.F	HexNAc(2)Hex(2)Fuc(1)	gi|379046526|gb|AFC87824.1| 30K protein 26	44
R.MVLN[+730.264]DTFIK.F	HexNAc(2)Hex(2)	gi|379046526|gb|AFC87824.1| 30K protein 26	45
R.M[+15.995]VLN[+876.322]DTFIK.F	HexNAc(2)Hex(2)Fuc(1)	gi|379046526|gb|AFC87824.1| 30K protein 26	46
R.DTYWEFHMDSVNVN[+876.322]ASR.F	HexNAc(2)Hex(2)Fuc(1)	gi|31559113|gb|AAP50847.1| cathepsin	47
R.AQEPTKN[+876.322]ETPVFK.T	HexNAc(2)Hex(2)Fuc(1)	gi|31559111|gb|AAP50846.1| immulectin	48
K.N[+876.322]LTNAAAPVDEAQTTYFFYAGFR.A	HexNAc(2)Hex(2)Fuc(1)	gi|31559111|gb|AAP50846.1| immulectin	49
R.FAN[+876.322]QSEEFYER.L	HexNAc(2)Hex(2)Fuc(1)	gi|297592381|gb|ADI47117.1| antennal esterase	50
R.FAN[+730.264]QSEEFYER.L	HexNAc(2)Hex(2)	gi|297592381|gb|ADI47117.1| antennal esterase	51
K.TMN[+876.322]GSENFITQDPFHLDFVK.R	HexNAc(2)Hex(2)Fuc(1)	gi|297592381|gb|ADI47117.1| antennal esterase	52
K.LSGQLGC[+57.021]STN[+876.322]NTK.D	HexNAc(2)Hex(2)Fuc(1)	gi|297592381|gb|ADI47117.1| antennal esterase	53
R.RATYSPEGTLYFSN[+892.317]ASLDDANDKTK.L	HexNAc(2)Hex(3)	gi|258642521|gb|ACQ82817.2| hemolin	54
R.ATYSPEGTLYFSN[+892.317]ASLDDANDKTK.L	HexNAc(2)Hex(3)	gi|258642521|gb|ACQ82817.2| hemolin	55
K.GN[+876.322]GSRPDLSVIR.S	HexNAc(2)Hex(2)Fuc(1)	gi|17298113|dbj|BAB78525.1| fibrillin-like protein	56
K.GN[+730.264]GSRPDLSVIR.S	HexNAc(2)Hex(2)	gi|17298113|dbj|BAB78525.1| fibrillin-like protein	57
K.GN[+1216.423]GSRPDLSVIR.S	HexNAc(2)Hex(5)	gi|17298113|dbj|BAB78525.1| fibrillin-like protein	58
K.GN[+1200.428]GSRPDLSVIR.S	HexNAc(2)Hex(4)Fuc(1)	gi|17298113|dbj|BAB78525.1| fibrillin-like protein	59
K.GN[+1038.375]GSRPDLSVIR.S	HexNAc(2)Hex(3)Fuc(1)	gi|17298113|dbj|BAB78525.1| fibrillin-like protein	60
R.SEHPRPTLSDN[+876.322]STSAFFDSLDVC[+57.021]R.I	HexNAc(2)Hex(2)Fuc(1)	gi|139004970|dbj|BAF52531.1| beta-N-acetylglucosaminidase 1	61
K.AHPDVEKN[+876.322]ATALR.E	HexNAc(2)Hex(2)Fuc(1)	gi|1381798|gb|AAB02853.1| apolipophorin-III precursor	62
K.AHPDVEKN[+714.269]ATALR.E	HexNAc(2)Hex(1)Fuc(1)	gi|1381798|gb|AAB02853.1| apolipophorin-III precursor	63
K.ITTDELVTFVDEYDMDISNAMYLDATEMQN[+892.317]K.T	HexNAc(2)Hex(3)	gi|1335609|emb|CAA31417.1| SP1	64
K.ITTDELVTFVDEYDMDISNAMYLDATEMQN[+876.322]K.T	HexNAc(2)Hex(2)Fuc(1)	gi|1335609|emb|CAA31417.1| SP1	65
K.ITTDELVTFVDEYDMDISNAMYLDATEMQN[+730.264]K.T	HexNAc(2)Hex(2)	gi|1335609|emb|CAA31417.1| SP1	66
K.ITTDELVTFVDEYDMDISNAMYLDATEMQN[+714.269]K.T	HexNAc(2)Hex(1)Fuc(1)	gi|1335609|emb|CAA31417.1| SP1	67
K.ITTDELVTFVDEYDMDISNAMYLDATEMQN[+406.159]K.T	HexNAc(2)	gi|1335609|emb|CAA31417.1| SP1	68
K.ITTDELVTFVDEYDMDISNAMYLDATEMQN[+349.137]K.T	HexNAc(1)Fuc(1)	gi|1335609|emb|CAA31417.1| SP1	69
K.ITTDELVTFVDEYDMDISNAMYLDATEMQN[+1038.375]K.T	HexNAc(2)Hex(3)Fuc(1)	gi|1335609|emb|CAA31417.1| SP1	70

K.ITTDELVTFVDEYDMDISN[+0.984]AMYLDATEMQN[+892.317] K.T	HexNAc(2)Hex(3)	gi|1335609|emb|CAA31417.1| SP1	71
K.ITTDELVTFVDEYDMDISN[+0.984]AMYLDATEMQN[+876.322] K.T	HexNAc(2)Hex(2)Fuc(1)	gi|1335609|emb|CAA31417.1| SP1	72
K.ITTDELVTFVDEYDMDISN[+0.984]AMYLDATEMQN[+730.264] K.T	HexNAc(2)Hex(2)	gi|1335609|emb|CAA31417.1| SP1	73
K.ITTDELVTFVDEYDMDISN[+0.984]AMYLDATEMQN[+714.269] K.T	HexNAc(2)Hex(1)Fuc(1)	gi|1335609|emb|CAA31417.1| SP1	74
K.ITTDELVTFVDEYDMDISN[+0.984]AMYLDATEMQN[+349.137] K.T	HexNAc(1)Fuc(1)	gi|1335609|emb|CAA31417.1| SP1	75
K.ITTDELVTFVDEYDMDISN[+0.984]AMYLDATEMQN[+1054.370] K.T	HexNAc(2)Hex(4)	gi|1335609|emb|CAA31417.1| SP1	76
K.DLGMSN[+876.322]TSK.T	HexNAc(2)Hex(2)Fuc(1)	gi|1335609|emb|CAA31417.1| SP1	77
K.[+42.011]ITTDELVTFVDEYDMDISNAMYLDATEMQN[+892.317] K.T	HexNAc(2)Hex(3)	gi|1335609|emb|CAA31417.1| SP1	78
R.VC[+57.021]SADQSYLAIINTKEEADHLVN[+876.322]MTR.L	HexNAc(2)Hex(2)Fuc(1)	gi|112982635|dbj|BAF03496.1| multi-binding protein	79
R.VC[+57.021]SADQSYLAIINTKEEADHLVN[+730.264]MTR.L	HexNAc(2)Hex(2)	gi|112982635|dbj|BAF03496.1| multi-binding protein	80
K.EEADHLVN[+876.322]MTR.L	HexNAc(2)Hex(2)Fuc(1)	gi|112982635|dbj|BAF03496.1| multi-binding protein	81
K.EEADHLVN[+730.264]MTR.L	HexNAc(2)Hex(2)	gi|112982635|dbj|BAF03496.1| multi-binding protein	82
K.YGN[+876.322]LSDSIIGIVK.E	HexNAc(2)Hex(2)Fuc(1)	BGIBMGA014513-PA	83
K.YGNLSDNVIGITIEN[+876.322]HSDDLYILTEDHVVYK.V	HexNAc(2)Hex(2)Fuc(1)	BGIBMGA014512-PA	84
R.DMMPQHN[+1054.370]ATAQTSPPPYTITTDAQSVAPGDSVEVVI	HexNAc(2)Hex(4)	BGIBMGA014360-PA	85
R.DMMPQHN[+1038.375]ATAQTSPPPYTITTDAQSVAPGDSVEVVI	HexNAc(2)Hex(3)Fuc(1)	BGIBMGA014360-PA	86
R.[+42.011]DMMPQHN[+1054.370]ATAQTSPPPYTITTDAQSVAPG DSVEVVIAGKLPEDTLR.G	HexNAc(2)Hex(4)	BGIBMGA014360-PA	87
R.EKGVYEVDLNLN[+876.322]TTVK.I	HexNAc(2)Hex(2)Fuc(1)	BGIBMGA014204-PA	88
K.MN[+892.317]GSDLFYAVTNDNK.A	HexNAc(2)Hex(3)	BGIBMGA014204-PA	89
K.MN[+730.264]GSDLFYAVTNDNK.A	HexNAc(2)Hex(2)	BGIBMGA014204-PA	90
K.YGTVDDSVIDIVKLN[+876.322]GTDALYVLTEDHTVYK.V	HexNAc(2)Hex(2)Fuc(1)	BGIBMGA014203-PA	91
K.LN[+892.317]GTDALYVLTEDHTVYK.V	HexNAc(2)Hex(3)	BGIBMGA014203-PA	92
K.LN[+876.322]GTDALYVLTEDHTVYK.V	HexNAc(2)Hex(2)Fuc(1)	BGIBMGA014203-PA	93
K.LN[+730.264]GTDALYVLTEDHTVYK.V	HexNAc(2)Hex(2)	BGIBMGA014203-PA	94
K.LN[+1702.581]GTDALYVLTEDHTVYK.V	HexNAc(2)Hex(8)	BGIBMGA014203-PA	95
K.LN[+1038.375]GTDALYVLTEDHTVYK.V	HexNAc(2)Hex(3)Fuc(1)	BGIBMGA014203-PA	96
R.YIQEHN[+876.322]LTSVADFHALFMR.N	HexNAc(2)Hex(2)Fuc(1)	BGIBMGA014116-PA	97
R.YIQEHN[+349.137]LTSVADFHALFMR.N	HexNAc(1)Fuc(1)	BGIBMGA014116-PA	98
R.YIQ[+0.984]EHN[+876.322]LTSVADFHALFMR.N	HexNAc(2)Hex(2)Fuc(1)	BGIBMGA014116-PA	99
R.YIQ[+0.984]EHN[+349.137]LTSVADFHALFMR.N	HexNAc(1)Fuc(1)	BGIBMGA014116-PA	100
R.QAEILDC[+57.021]PTN[+876.322]SSK.A	HexNAc(2)Hex(2)Fuc(1)	BGIBMGA013812-PA	101
R.QAEILDC[+57.021]PTN[+730.264]SSK.A	HexNAc(2)Hex(2)	BGIBMGA013812-PA	102
R.ELMDC[+57.021]TKN[+876.322]DTGEPC[+57.021]PELK.E	HexNAc(2)Hex(2)Fuc(1)	BGIBMGA012994-PA	103
K.QNADIIVFPELTLTN[+730.264]R.S	HexNAc(2)Hex(2)	BGIBMGA012994-PA	104
R.N[+1038.375]MTGDPFTFC[+57.021]RPFEAR.D	HexNAc(2)Hex(3)Fuc(1)	BGIBMGA012968-PA	105
R.N[+1038.375]M[+15.995]TGDPFTFC[+57.021]RPFEAR.D	HexNAc(2)Hex(3)Fuc(1)	BGIBMGA012968-PA	106
R.N[+876.322]ASDDVIAFDNELRK.H	HexNAc(2)Hex(2)Fuc(1)	BGIBMGA012938-PA	107
R.N[+0.984]GC[+57.021]SN[+876.322]LSLPSEEPVIR.A	HexNAc(2)Hex(2)Fuc(1)	BGIBMGA012645-PA	108
K.KLDTYEIETARN[+0.984]GC[+57.021]SN[+876.322]LSLPSEEPVI	HexNAc(2)Hex(2)Fuc(1)	BGIBMGA012645-PA	109
K.N[+730.264]ISIIQDYPK.A	HexNAc(2)Hex(2)	BGIBMGA012642-PA	110
K.NFLDFWAYPLN[+876.322]ETDAKNLNEK.L	HexNAc(2)Hex(2)Fuc(1)	BGIBMGA012031-PA	111
K.NFLDFWAYPLN[+876.322]ETDAK.N	HexNAc(2)Hex(2)Fuc(1)	BGIBMGA012031-PA	112
K.NFLDFWAYPLN[+730.264]ETDAK.N	HexNAc(2)Hex(2)	BGIBMGA012031-PA	113
K.NFLDFWAYPLN[+714.269]ETDAK.N	HexNAc(2)Hex(1)Fuc(1)	BGIBMGA012031-PA	114
K.NFLDFWAYPLN[+349.137]ETDAK.N	HexNAc(1)Fuc(1)	BGIBMGA012031-PA	115
K.N[+0.984]FLDFWAYPLN[+876.322]ETDAK.N	HexNAc(2)Hex(2)Fuc(1)	BGIBMGA012031-PA	116
K.MANDLGYN[+876.322]ETKGDHR.A	HexNAc(2)Hex(2)Fuc(1)	BGIBMGA012031-PA	117
K.MANDLGYN[+876.322]ETK.G	HexNAc(2)Hex(2)Fuc(1)	BGIBMGA012031-PA	118
K.MANDLGYN[+730.264]ETKGDHR.A	HexNAc(2)Hex(2)	BGIBMGA012031-PA	119
K.MANDLGYN[+714.269]ETKGDHR.A	HexNAc(2)Hex(1)Fuc(1)	BGIBMGA012031-PA	120
K.MAN[+0.984]DLGYN[+876.322]ETK.G	HexNAc(2)Hex(2)Fuc(1)	BGIBMGA012031-PA	121
K.KNFLDFWAYPLN[+876.322]ETDAK.N	HexNAc(2)Hex(2)Fuc(1)	BGIBMGA012031-PA	122
R.NN[+876.322]LTVSTADGNLHINAK.L	HexNAc(2)Hex(2)Fuc(1)	BGIBMGA011609-PA	123
K.SGFLTEN[+876.322]K.T	HexNAc(2)Hex(2)Fuc(1)	BGIBMGA011432-PA	124
K.AN[+1864.634]YTEVIER.G	HexNAc(2)Hex(9)	BGIBMGA011424-PA	125
K.AN[+1702.581]YTEVIER.G	HexNAc(2)Hex(8)	BGIBMGA011424-PA	126
K.AN[+1540.529]YTEVIER.G	HexNAc(2)Hex(7)	BGIBMGA011424-PA	127
K.AN[+1378.476]YTEVIER.G	HexNAc(2)Hex(6)	BGIBMGA011424-PA	128
K.AN[+1216.423]YTEVIER.G	HexNAc(2)Hex(5)	BGIBMGA011424-PA	129

R.HMLDGFDN[+876.322]STQLC[+57.021]YGDR.K	HexNAc(2)Hex(2)Fuc(1)	BGIBMGA010306-PA	130
R.HMLDGFDN[+730.264]STQLC[+57.021]YGDR.K	HexNAc(2)Hex(2)	BGIBMGA010306-PA	131
R.DGYN[+730.264]GTC[+57.021]VLSK.R	HexNAc(2)Hex(2)	BGIBMGA010306-PA	132
K.YGN[+876.322]LSDSIIGIAK.E	HexNAc(2)Hex(2)Fuc(1)	BGIBMGA010047-PA	133
K.YGN[+876.322]LSDNVIGIAK.E	HexNAc(2)Hex(2)Fuc(1)	BGIBMGA010043-PA	134
K.YGN[+876.322]LSDSVIGIVK.E	HexNAc(2)Hex(2)Fuc(1)	BGIBMGA010041-PA	135
K.YGN[+876.322]LSDSIIGILK.E	HexNAc(2)Hex(2)Fuc(1)	BGIBMGA010040-PA	136
K.YGN[+730.264]LSDSIIGILK.E	HexNAc(2)Hex(2)	BGIBMGA010040-PA	137
K.AN[+876.322]ISWSALK.R	HexNAc(2)Hex(2)Fuc(1)	BGIBMGA009336-PA	138
R.VDIPNSN[+1864.634]LTVEYHDVK.T	HexNAc(2)Hex(9)	BGIBMGA009259-PA	139
K.NAVNQFVN[+406.159]TSPR.S	HexNAc(2)	BGIBMGA009180-PA	140
K.IPTDMFN[+1702.581]SSDTMPSR.L	HexNAc(2)Hex(8)	BGIBMGA009027-PA	141
K.IPTDMFN[+1216.423]SSDTMPSR.L	HexNAc(2)Hex(5)	BGIBMGA009027-PA	142
R.APAN[+876.322]TSWESGASALEHALK.L	HexNAc(2)Hex(2)Fuc(1)	BGIBMGA008768-PA	143
R.APAN[+730.264]TSWESGASALEHALK.L	HexNAc(2)Hex(2)	BGIBMGA008768-PA	144
R.DLLN[+876.322]C[+57.021]TK.G	HexNAc(2)Hex(2)Fuc(1)	BGIBMGA008307-PA	145
R.DLLN[+730.264]C[+57.021]TK.G	HexNAc(2)Hex(2)	BGIBMGA008307-PA	146
R.ATRPNIC[+57.021]DLVYN[+876.322]NTLDVR.C	HexNAc(2)Hex(2)Fuc(1)	BGIBMGA008307-PA	147
K.NN[+876.322]ITMK.Y	HexNAc(2)Hex(2)Fuc(1)	BGIBMGA008307-PA	148
K.N[+0.984]N[+876.322]ITMK.Y	HexNAc(2)Hex(2)Fuc(1)	BGIBMGA008307-PA	149
K.SLQPFDQLELGTN[+876.322]LTYGEMASQEGLK.E	HexNAc(2)Hex(2)Fuc(1)	BGIBMGA007767-PA	150
K.ELFVGHELKPLEVFN[+876.322]K.T	HexNAc(2)Hex(2)Fuc(1)	BGIBMGA007655-PA	151
K.TEVIRN[+876.322]NSYLVDPTVDPQAIAEFPEER.T	HexNAc(2)Hex(2)Fuc(1)	BGIBMGA007254-PA	152
R.SGLVLYETTLN[+876.322]ETEGVLVLNKPR.D	HexNAc(2)Hex(2)Fuc(1)	BGIBMGA006815-PA	153
R.SGLVLYETTLN[+730.264]ETEGVLVLNKPR.D	HexNAc(2)Hex(2)	BGIBMGA006815-PA	154
K.N[+730.264]SSNLDFFTPETDPRPER.I	HexNAc(2)Hex(2)	BGIBMGA006339-PA	155
R.YAN[+892.317]ITDSSIIGVR.A	HexNAc(2)Hex(3)	BGIBMGA006214-PA	156
R.N[+876.322]TTWHDPTKY.-	HexNAc(2)Hex(2)Fuc(1)	BGIBMGA005942-PA	157
R.YLFDFTKN[+876.322]DSVETWEEISDTVR.D	HexNAc(2)Hex(2)Fuc(1)	BGIBMGA005938-PA	158
R.ETN[+876.322]ESHILSK.A	HexNAc(2)Hex(2)Fuc(1)	BGIBMGA005876-PA	159
K.VLFSVQN[+876.322]ISNPR.G	HexNAc(2)Hex(2)Fuc(1)	BGIBMGA005876-PA	160
R.SVFVN[+730.264]QTIR.N	HexNAc(2)Hex(2)	BGIBMGA005142-PA	161
R.LLNLNVDELQNFANALHN[+876.322]QTIEK.S	HexNAc(2)Hex(2)Fuc(1)	BGIBMGA005105-PA	162
K.AIIN[+730.264]ISDNTNVR.S	HexNAc(2)Hex(2)	BGIBMGA004398-PA	163
K.ALLYSDWIEC[+57.021]NHN[+876.322]K.T	HexNAc(2)Hex(2)Fuc(1)	BGIBMGA003917-PA	164
R.GN[+892.317]ITFTQVQDGK.V	HexNAc(2)Hex(3)	BGIBMGA002907-PA	165
R.GN[+876.322]ITFTQVQDGK.V	HexNAc(2)Hex(2)Fuc(1)	BGIBMGA002907-PA	166
R.GN[+730.264]ITFTQVQDGK.V	HexNAc(2)Hex(2)	BGIBMGA002907-PA	167
R.GN[+203.079]ITFTQVQDGK.V	HexNAc(1)	BGIBMGA002907-PA	168
R.SLN[+876.322]LTAMPEK.F	HexNAc(2)Hex(2)Fuc(1)	BGIBMGA002526-PA	169
R.SLN[+730.264]LTAMPEK.F	HexNAc(2)Hex(2)	BGIBMGA002526-PA	170
R.EQLQGVEPPVN[+730.264]R.T	HexNAc(2)Hex(2)	BGIBMGA002526-PA	171
R.EQLQ[+0.984]GVEPPVN[+876.322]RTEDDFDAAAK.Y	HexNAc(2)Hex(2)Fuc(1)	BGIBMGA002526-PA	172
R.EQLQ[+0.984]GVEPPVN[+730.264]RTEDDFDAAAK.Y	HexNAc(2)Hex(2)	BGIBMGA002526-PA	173
R.ASLAEWEYTSN[+730.264]ITKENEEK.S	HexNAc(2)Hex(2)	BGIBMGA002526-PA	174
K.LREQLQGVEPPVN[+730.264]R.T	HexNAc(2)Hex(2)	BGIBMGA002526-PA	175
K.KN[+892.317]FTDYVNLYNEAAK.L	HexNAc(2)Hex(3)	BGIBMGA002526-PA	176
K.KN[+730.264]FTDYVNLYNEAAK.L	HexNAc(2)Hex(2)	BGIBMGA002526-PA	177
K.NIDLLKPTESLQN[+876.322]LTLENPGR.R	HexNAc(2)Hex(2)Fuc(1)	BGIBMGA002518-PA	178
R.NHHEQFGELLQQLNDN[+876.322]ETNSK.T	HexNAc(2)Hex(2)Fuc(1)	BGIBMGA002368-PA	179
R.GVN[+876.322]FTQK.N	HexNAc(2)Hex(2)Fuc(1)	BGIBMGA002368-PA	180
R.GVN[+730.264]FTQK.N	HexNAc(2)Hex(2)	BGIBMGA002368-PA	181
R.LN[+876.322]C[+57.021]TSC[+57.021]AGSLR.L	HexNAc(2)Hex(2)Fuc(1)	BGIBMGA002365-PA	182
K.TPVSEDKIDPATAVAMFNVIFFQGHWHVPFN[+876.322]ASETEE KDFHVDEK.T	HexNAc(2)Hex(2)Fuc(1)	BGIBMGA001983-PA	183
R.LVSIAQKYN[+876.322]K.T	HexNAc(2)Hex(2)Fuc(1)	BGIBMGA001351-PA	184
K.VVQVTN[+876.322]DTEQAVIYALEAGYTHIDTAYK.Y	HexNAc(2)Hex(2)Fuc(1)	BGIBMGA001351-PA	185
K.VVQVTN[+730.264]DTEQAVIYALEAGYTHIDTAYK.Y	HexNAc(2)Hex(2)	BGIBMGA001351-PA	186
K.SLENLNLEYVDLYLIHWPIAMFEN[+876.322]DTLLDNVDFLDT	HexNAc(2)Hex(2)Fuc(1)	BGIBMGA001351-PA	187
K.SLENLNLEYVDLYLIHWPIAMFEN[+730.264]DTLLDNVDFLDT	HexNAc(2)Hex(2)	BGIBMGA001351-PA	188
K.GKVVQVTN[+876.322]DTEQAVIYALEAGYTHIDTAYK.Y	HexNAc(2)Hex(2)Fuc(1)	BGIBMGA001351-PA	189
K.GKVVQVTN[+730.264]DTEQAVIYALEAGYTHIDTAYK.Y	HexNAc(2)Hex(2)	BGIBMGA001351-PA	190
K.[+42.011]GKVVQVTN	HexNAc(2)Hex(2)Fuc(1)	BGIBMGA001351-PA	191
K.[+42.011]GKVVQVTN	HexNAc(2)Hex(2)	BGIBMGA001351-PA	192
K.TVLTVDC[+57.021]PWLNFESN[+1216.423]R.T	HexNAc(2)Hex(5)	BGIBMGA001347-PA	193
R.EGAYN[+876.322]ASLLYDSHAGR.I	HexNAc(2)Hex(2)Fuc(1)	BGIBMGA001098-PA	194
K.LN[+730.264]VSAVAPNAQPIDAR.G	HexNAc(2)Hex(2)	BGIBMGA001098-PA	195
K.C[+57.021]N[+876.322]DSIEVIQC[+57.021]KPR.D	HexNAc(2)Hex(2)Fuc(1)	BGIBMGA001098-PA	196
R.AFGGNPDN[+730.264]VTLAGQSAGAAAAHLLTLSK.A	HexNAc(2)Hex(2)	BGIBMGA000772-PA	197
K.LLFSILGVN[+876.322]STNPDEIHEK.L	HexNAc(2)Hex(2)Fuc(1)	BGIBMGA000772-PA	198
K.LLFSILGVN[+730.264]STNPDEIHEK.L	HexNAc(2)Hex(2)	BGIBMGA000772-PA	199
K.QSFPTVESANN[+876.322]LSLVEK.C	HexNAc(2)Hex(2)Fuc(1)	BGIBMGA000462-PA	200
