# Supplementary material for: Identification of N-linked Glycoproteins in Silkworm Serum Using Con A Lectin Affinity Chromatography and Mass Spectrometry
Source: J Insect Sci. 2021 Aug 17;21(4):14. doi: 10.1093/jisesa/ieab057 (PMC8367846; doi:10.1093/jisesa/ieab057)
Supplement: ieab057_suppl_Supplementary_Figure_S1 [file ieab057_suppl_supplementary_figure_s1.pdf]

Supplementary Figure S1: MS/MS spectra of N-linked glycopeptides. The N-linked glycopetide sequences and N-linked glycan chain types are in the Supplementary Table S4.

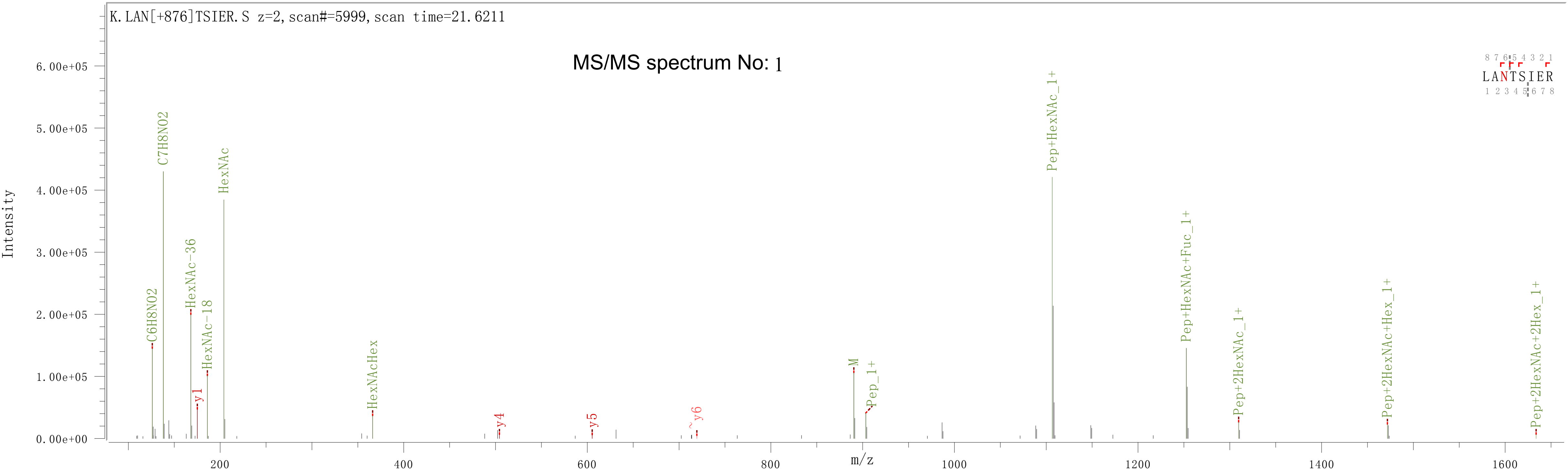

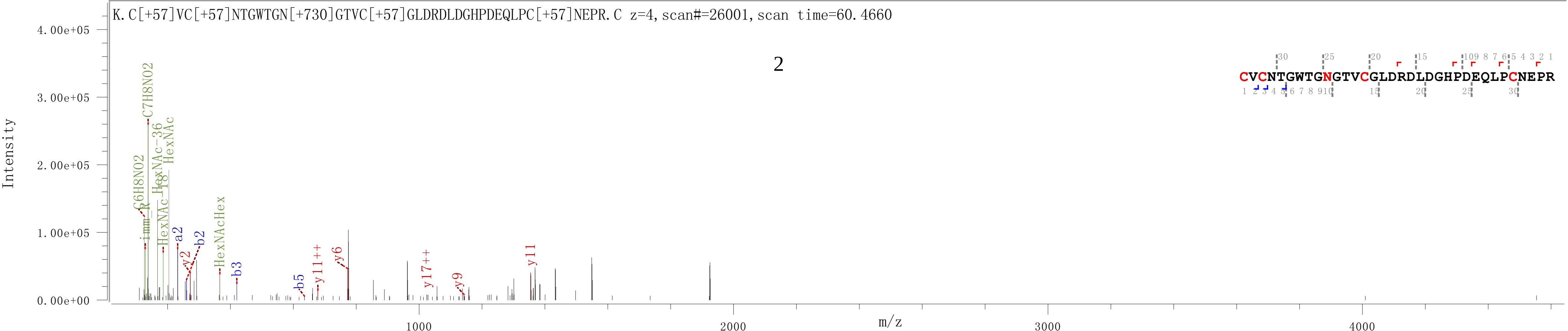
K.C[+57]VC[+57]NTGWTGN[+730]GTVC[+57]GLDRDLDGHPDEQLPC[+57]NEPR.C z=4,scan#=26001,scan time=60.4660
2
Intensity
m/z
4.00e+05
3.00e+05
2.00e+05
1.00e+05
0.00e+00
1000
2000
3000
4000
C7H8NO2
C6H8NO2
HexNAc-36
HexNAc
imm_R
HexNAc-18
HexNAcHex
a2
b2
y2
b3
b5
y11++
y6
y17++
y9
y11
CVCNTGWTGNGTVCGLDRDLDGHPDEQLPCNEPR

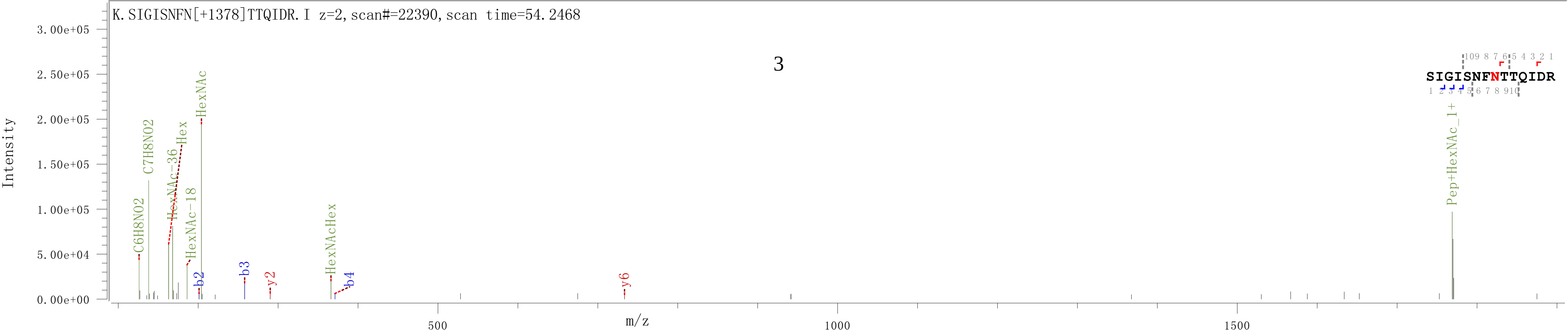

K.SIGISNFN[+1378]TTQIDR.I z=2,scan#=22390,scan time=54.2468
3
SIGISNFNTTQIDR
Intensity
m/z
C6H8NO2
C7H8NO2
HexNAc-36
Hex
HexNAc-18
HexNAc
b2
b3
y2
HexNAcHex
b4
y6
Pep+HexNAc_1+

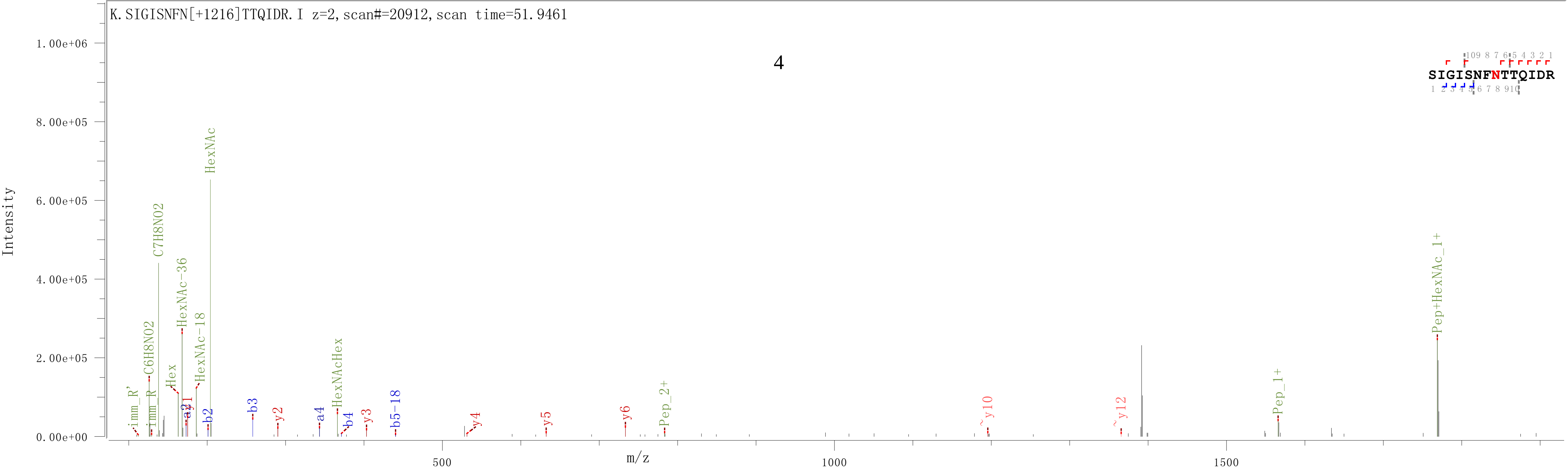
K.SIGISNFN[+1216]TTQIDR.I z=2,scan#=20912,scan time=51.9461
4
SIGISNFNTTQIDR
Intensity
m/z
HexNAc
C7H8NO2
HexNAc-36
HexNAc-18
C6H8NO2
Hex
imm_R'
imm_R
HexNAcHex
Pep_2+
Pep_1+
Pep+HexNAc_1+
b2
b3
b4
b5-18
a4
y2
y3
y4
y5
y6
~y10
~y12
1.00e+06
8.00e+05
6.00e+05
4.00e+05
2.00e+05
0.00e+00
500
1000
1500

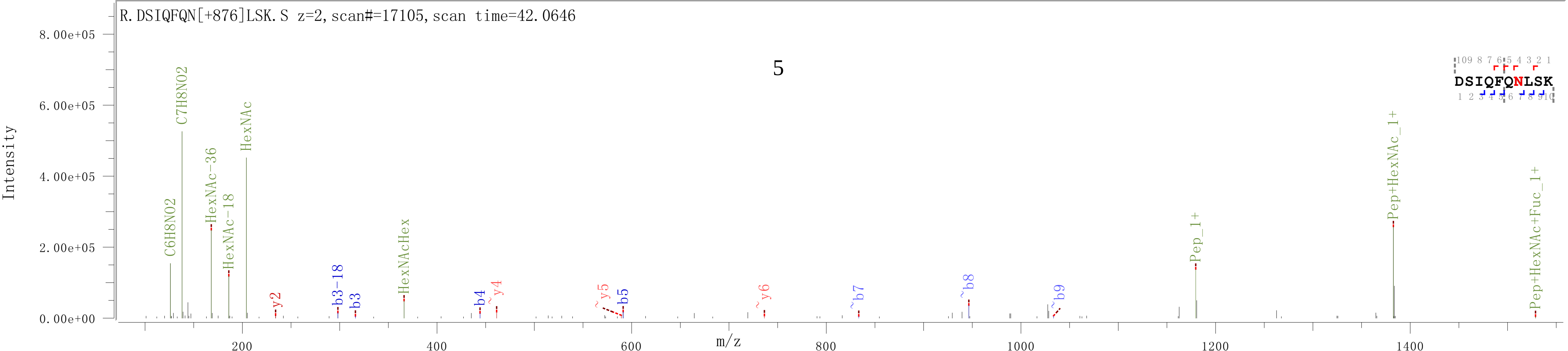

R.DSIQFQN[+876]LSK.S z=2,scan#=17105,scan time=42.0646
5
Intensity
m/z
C6H8NO2
C7H8NO2
HexNAc-36
HexNAc-18
HexNAc
y2
b3-18
b3
HexNAcHex
b4
~y4
~y5
b5
~y6
~b7
~b8
~b9
Pep_1+
Pep+HexNAc_1+
Pep+HexNAc+Fuc_1+
DSIQFQNLSK

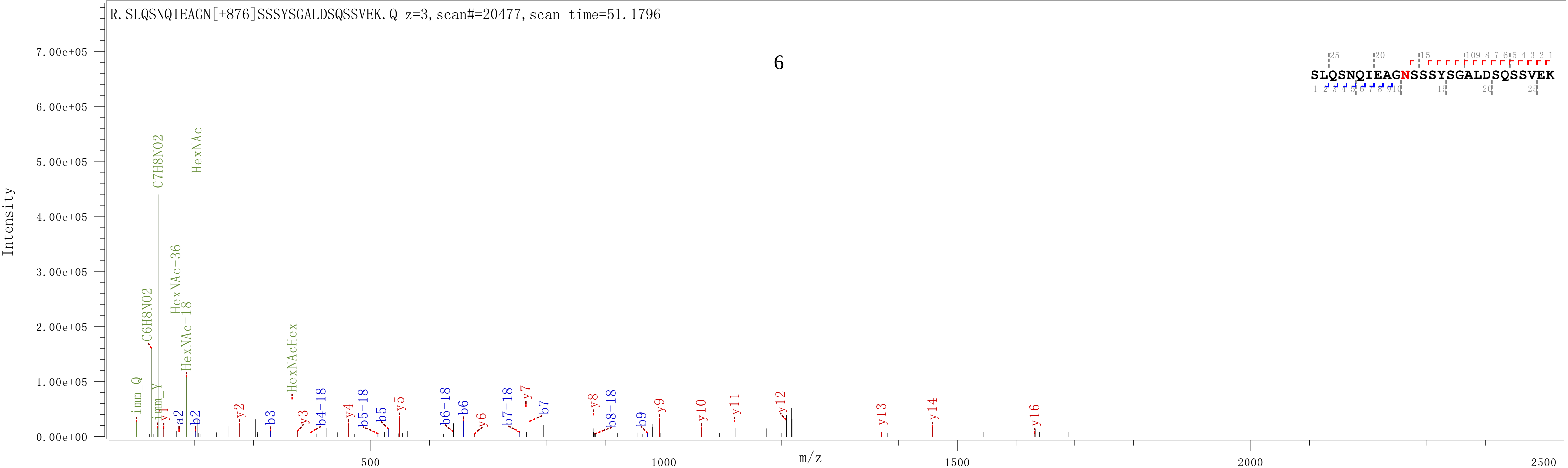

R.SLQSNQIEAGN[+876]SSSYSGALDSQSSVEK.Q z=3,scan#=20477,scan time=51.1796
6
SLQSNQIEAGNSSSYSGALDSQSSVEK
Intensity
m/z
imm_Q
C6H8NO2
imm_Y
y1
C7H8NO2
HexNAc-36
a2
HexNAc-18
b2
HexNAc
y2
b3
HexNAcHex
y3
b4-18
y4
b5-18
b5
y5
b6-18
b6
y6
b7-18
y7
b7
y8
b8-18
b9
y9
y10
y11
y12
y13
y14
y16

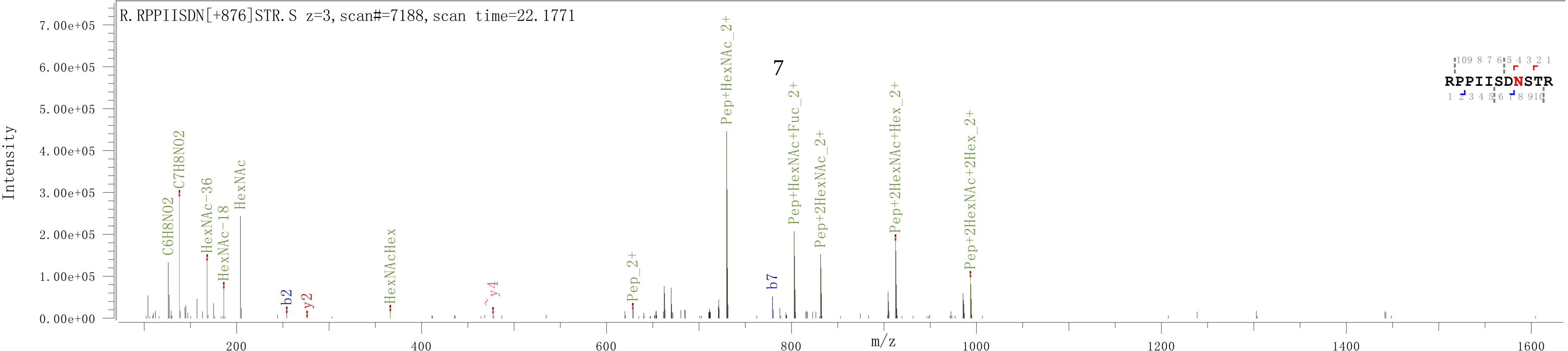

R.RPPIISDN[+876]STR.S z=3,scan#=7188,scan time=22.1771
7
RPPIISDNSTR
Intensity
m/z
C6H8NO2
C7H8NO2
HexNAc-36
HexNAc-18
HexNAc
b2
y2
HexNAcHex
~y4
Pep_2+
Pep+HexNAc_2+
b7
Pep+HexNAc+Fuc_2+
Pep+2HexNAc_2+
Pep+2HexNAc+Hex_2+
Pep+2HexNAc+2Hex_2+

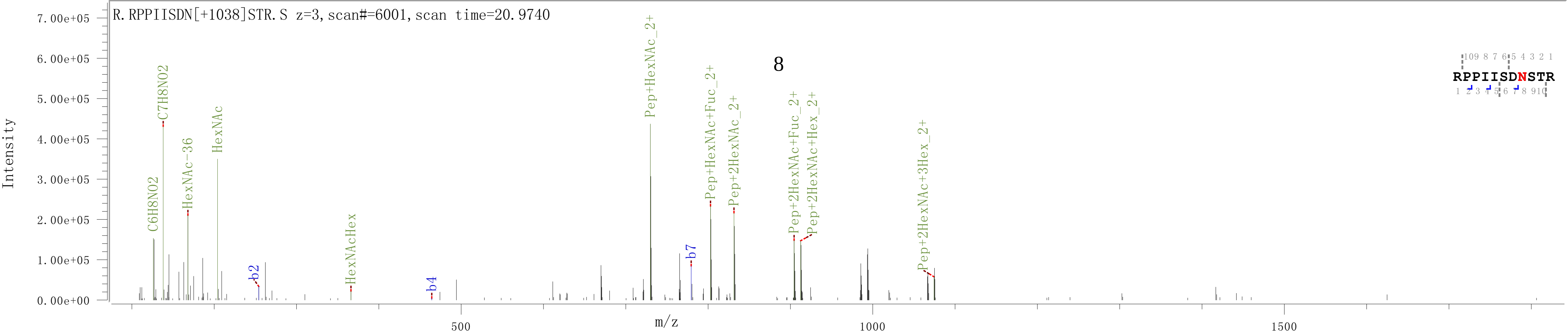
R.RPPIISDN[+1038]STR.S z=3,scan#=6001,scan time=20.9740
Intensity
7.00e+05
6.00e+05
5.00e+05
4.00e+05
3.00e+05
2.00e+05
1.00e+05
0.00e+00
m/z
500
1000
1500
C6H8NO2
C7H8NO2
HexNAc-36
HexNAc
b2
HexNAcHex
b4
Pep+HexNAc_2+
b7
Pep+HexNAc+Fuc_2+
Pep+2HexNAc_2+
Pep+2HexNAc+Fuc_2+
Pep+2HexNAc+Hex_2+
Pep+2HexNAc+3Hex_2+
RPPIISDNSTR

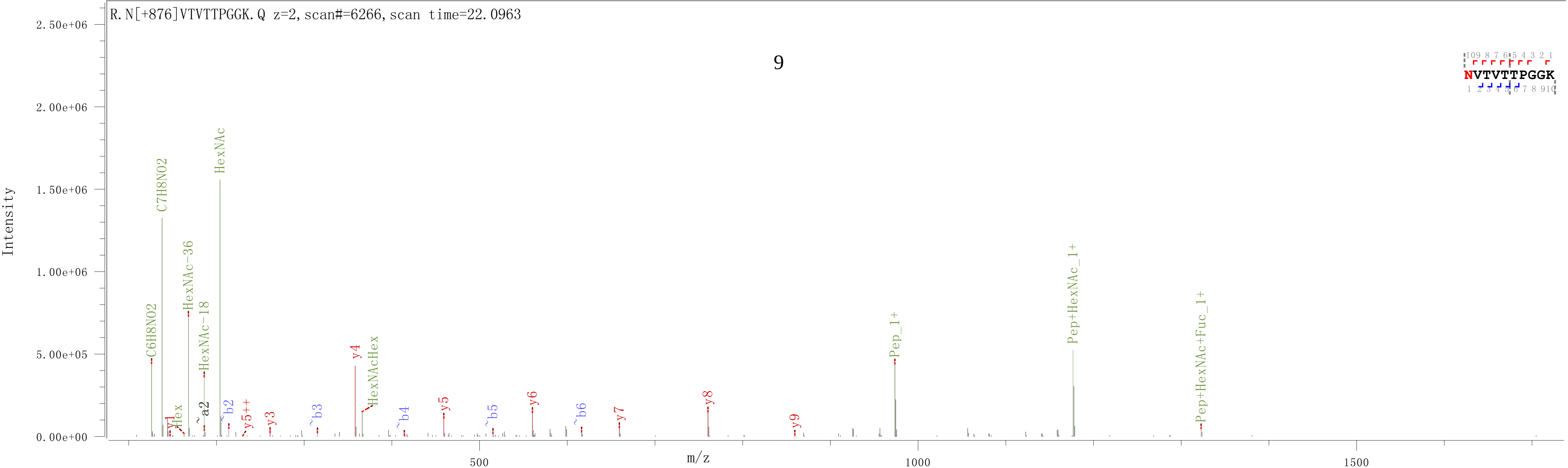

R.N[+876]VTVTTPGGK.Q z=2,scan#=6266,scan time=22.0963
9
NVTVTTPGGK
Intensity
m/z
2.50e+06
2.00e+06
1.50e+06
1.00e+06
5.00e+05
0.00e+00
500
1000
1500
C6H8NO2
C7H8NO2
HexNAc-36
HexNAc-18
HexNAc
Hex
y1
~a2
~b2
y5++
y3
~b3
y4
HexNAcHex
~b4
y5
~b5
y6
~b6
y7
y8
y9
Pep_1+
Pep+HexNAc_1+
Pep+HexNAc+Fuc_1+

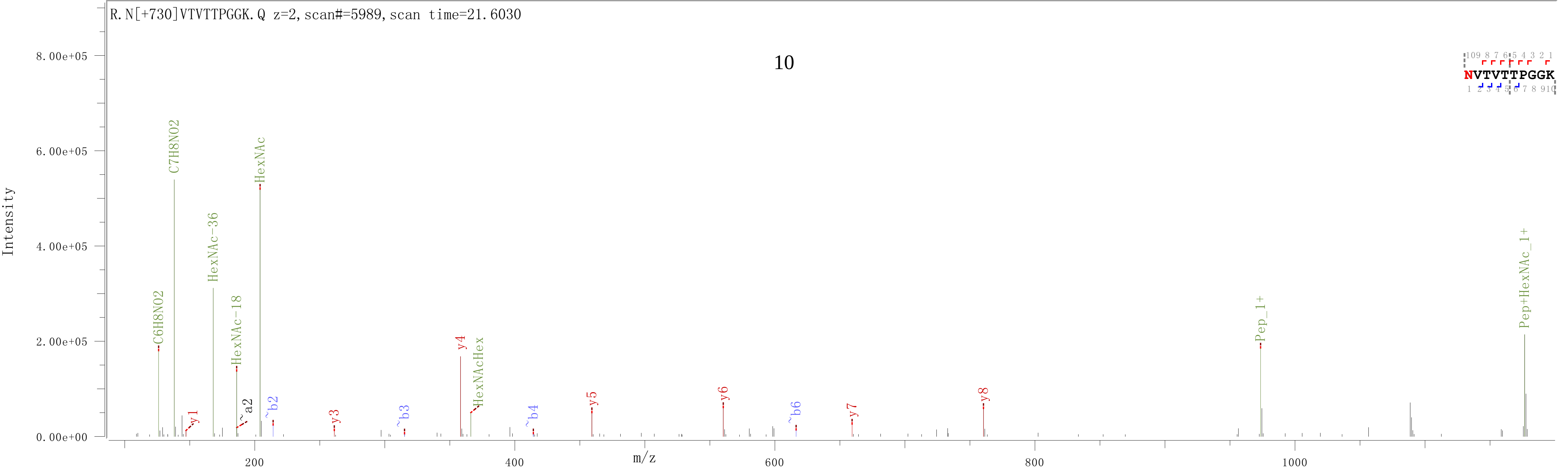

R.N[+730]VTVTTPGGK.Q z=2,scan#=5989,scan time=21.6030
10
NVTVTTPGGK
Intensity
m/z
C6H8NO2
C7H8NO2
HexNAc-36
HexNAc-18
HexNAc
y1
~a2
~b2
y3
~b3
y4
HexNAcHex
~b4
y5
y6
~b6
y7
y8
Pep_1+
Pep+HexNAc_1+

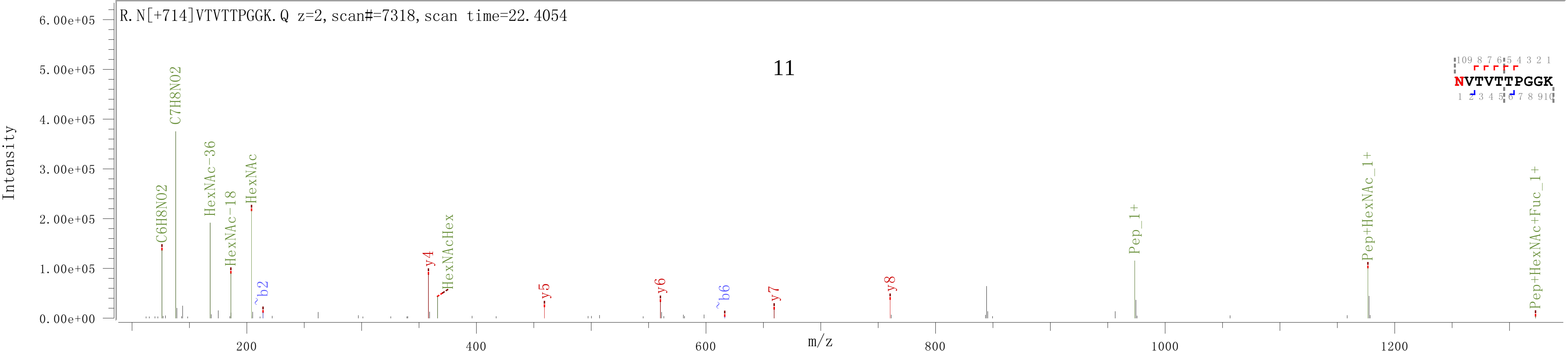

R.N[+714]VTVTTPGGK.Q z=2,scan#=7318,scan time=22.4054
11
NVTVTTPGGK
Intensity
m/z
6.00e+05
5.00e+05
4.00e+05
3.00e+05
2.00e+05
1.00e+05
0.00e+00
200
400
600
800
1000
1200
C6H8NO2
C7H8NO2
HexNAc-36
HexNAc-18
HexNAc
~b2
y4
HexNAcHex
y5
y6
~b6
y7
y8
Pep_1+
Pep+HexNAc_1+
Pep+HexNAc+Fuc_1+

12

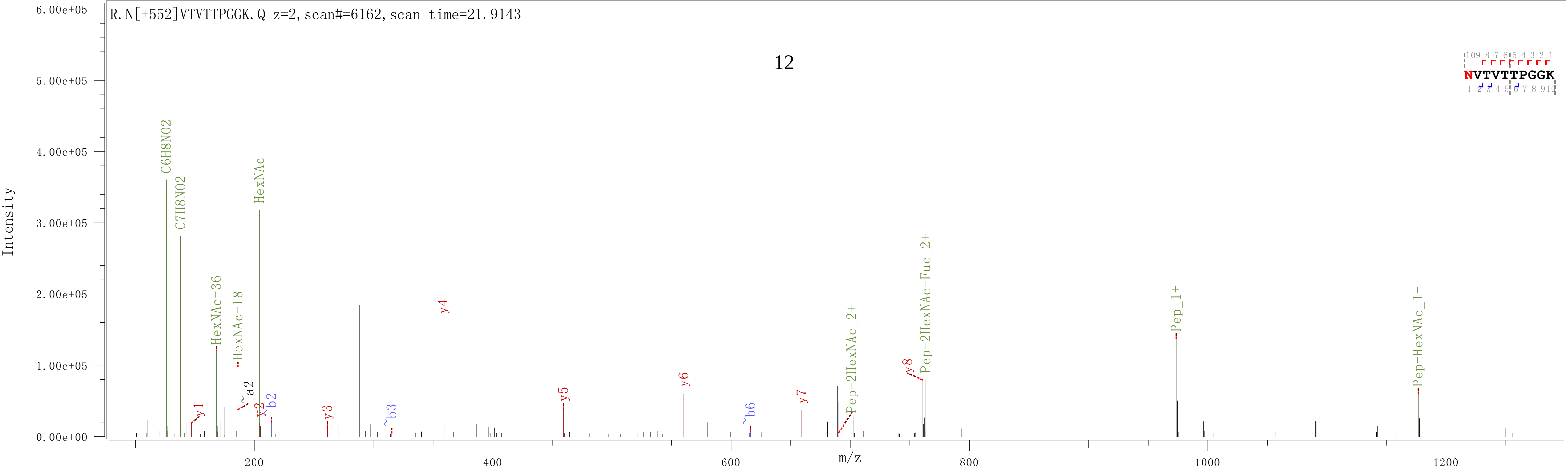

R.N[+552]VTVTTPGGK.Q z=2,scan#=6162,scan time=21.9143
NVTVTTPGGK
Intensity
m/z
C6H8NO2
C7H8NO2
HexNAc
HexNAc-36
HexNAc-18
y1
~a2
y2
b2
y3
~b3
y4
y5
y6
~b6
y7
Pep+2HexNAc_2+
y8
Pep+2HexNAc+Fuc_2+
Pep_1+
Pep+HexNAc_1+

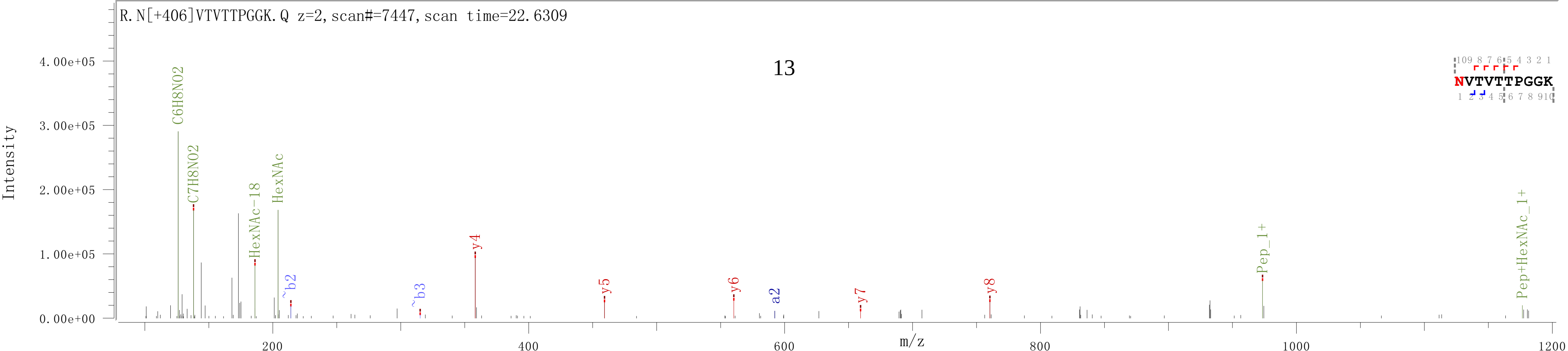
R.N[+406]VTVTTPGGK.Q z=2,scan#=7447,scan time=22.6309
Intensity
m/z
C6H8NO2
C7H8NO2
HexNAc-18
HexNAc
~b2
~b3
y4
y5
y6
a2
y7
y8
Pep_1+
Pep+HexNAc_1+
NVTVTTPGGK
109 8 7 6 5 4 3 2 1
1 2 3 4 5 6 7 8 910
0.00e+00
1.00e+05
2.00e+05
3.00e+05
4.00e+05
200
400
600
800
1000
1200

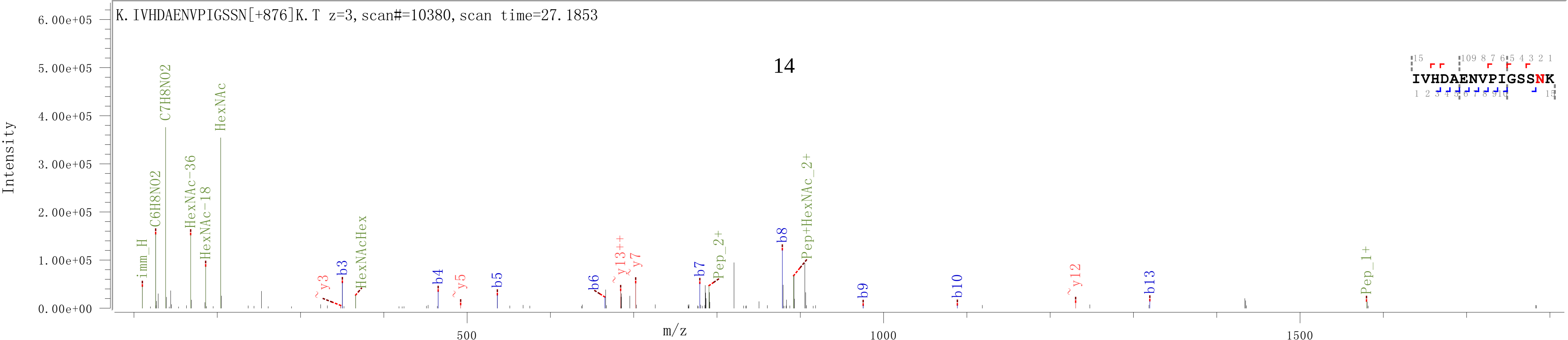
K.IVHDAENVPIGSSN[+876]K.T z=3,scan#=10380,scan time=27.1853
Intensity
m/z
imm_H
C6H8NO2
C7H8NO2
HexNAc-36
HexNAc-18
HexNAc
~y3
b3
HexNAcHex
b4
~y5
b5
b6
~y13++
y7
b7
Pep_2+
b8
Pep+HexNAc_2+
b9
b10
~y12
b13
Pep_1+
IVHDAENVPIGSSNK

14

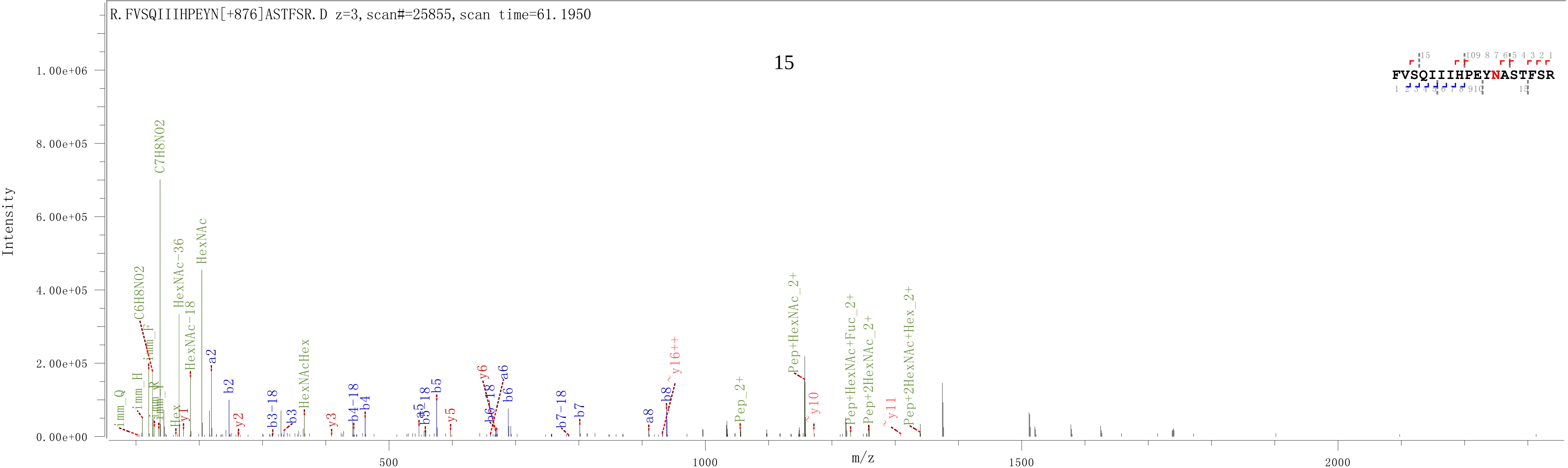
R.FVSQIIIHPEYN[+876]ASTFSR.D z=3,scan#=25855,scan time=61.1950
FVSQIIIHPEYNASTFSR
Intensity
m/z
1.00e+06
8.00e+05
6.00e+05
4.00e+05
2.00e+05
0.00e+00
500
1000
1500
2000
C7H8NO2
C6H8NO2
HexNAc
HexNAc-36
HexNAc-18
HexNAcHex
imm_Q
imm_H
imm_F
Hex
y1
a2
b2
y2
b3-18
b3
y3
b4-18
b4
a5
b5-18
b5
y5
y6
b6-18
a6
b6
b7-18
b7
a8
b8
y16++
Pep_2+
Pep+HexNAc_2+
y10
Pep+HexNAc+Fuc_2+
Pep+2HexNAc_2+
y11
Pep+2HexNAc+Hex_2+

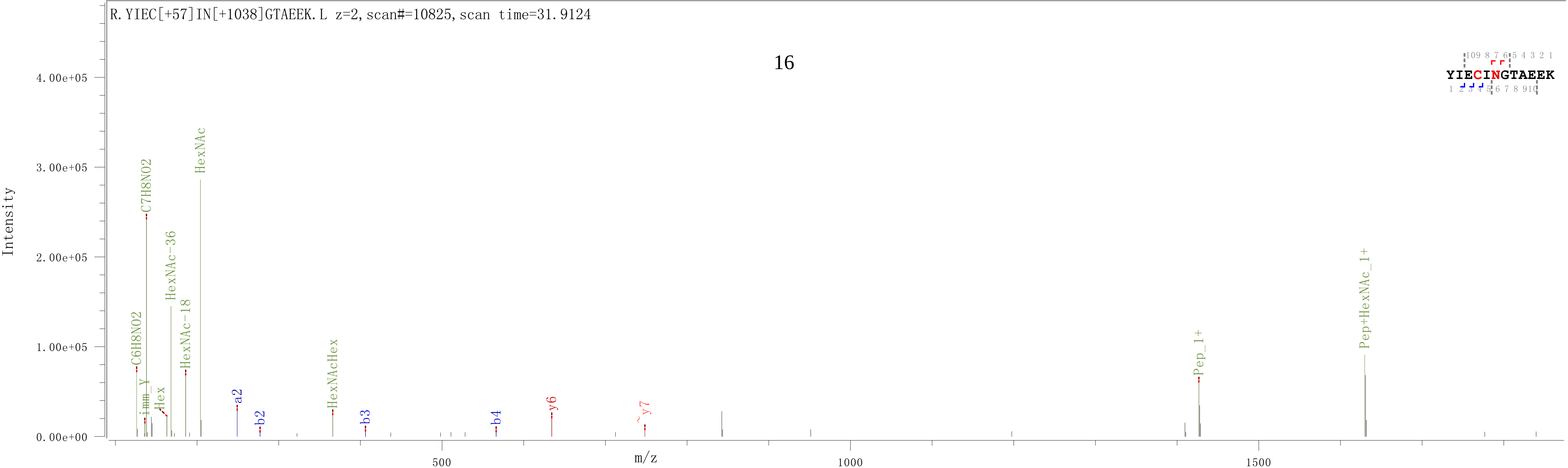
R.YIEC[+57]IN[+1038]GTAEEK.L z=2,scan#=10825,scan time=31.9124
16
YIECINGTAEEK
Intensity
m/z
C6H8NO2
C7H8NO2
imm_Y
Hex
HexNAc-36
HexNAc-18
HexNAc
a2
b2
HexNAcHex
b3
b4
y6
~y7
Pep_1+
Pep+HexNAc_1+

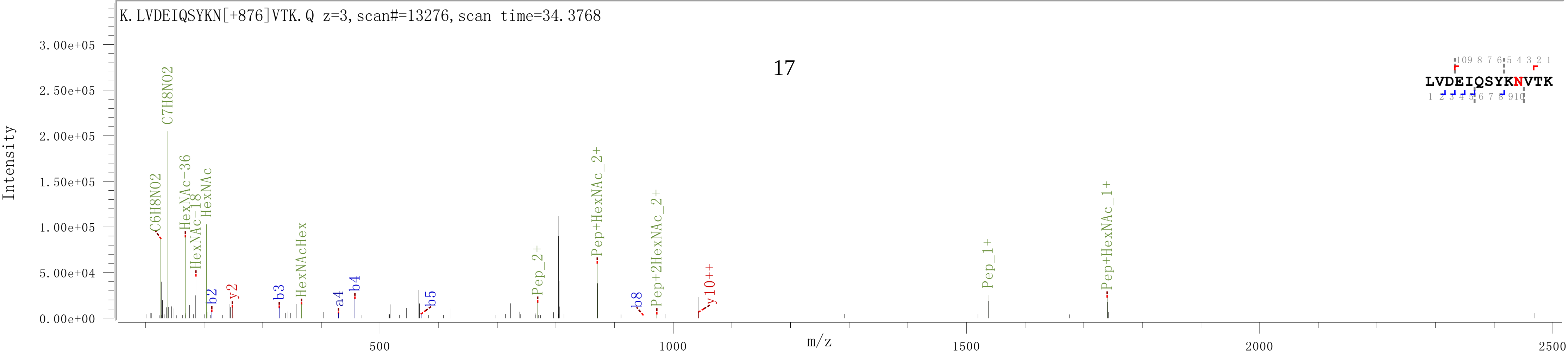

K.LVDEIQSYKN[+876]VTK.Q z=3,scan#=13276,scan time=34.3768
Intensity
m/z
C7H8NO2
C6H8NO2
HexNAc-36
HexNAc-18
HexNAc
HexNAcHex
b2
y2
b3
a4
b4
b5
Pep_2+
Pep+HexNAc_2+
b8
Pep+2HexNAc_2+
y10++
Pep_1+
Pep+HexNAc_1+
LVDEIQSYKNVTK

18

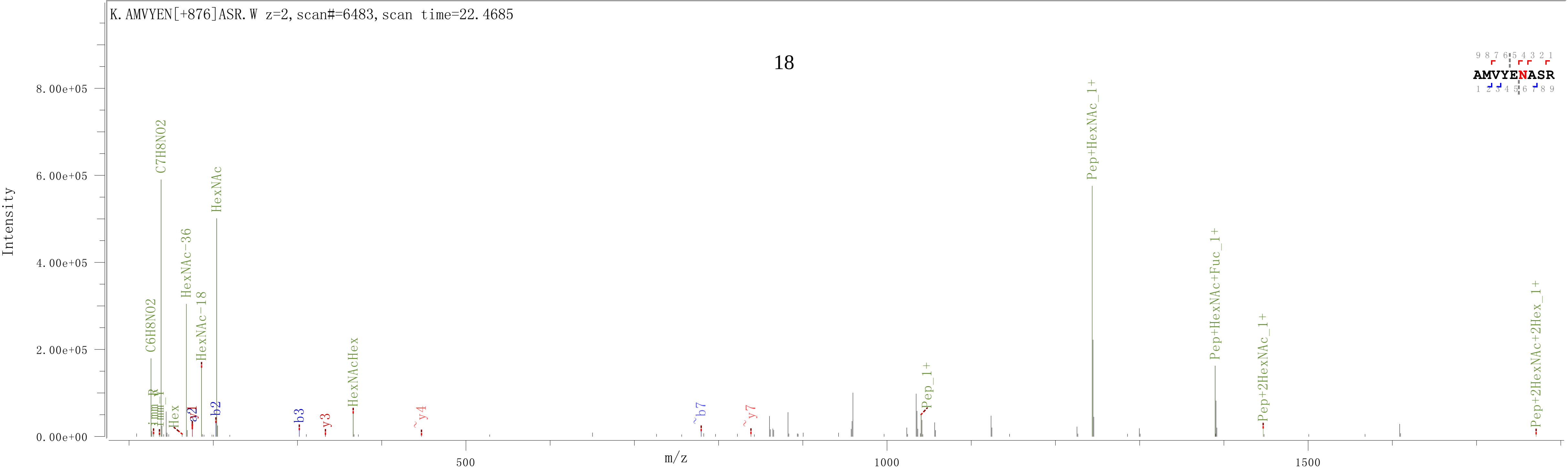
K.AMVYEN[+876]ASR.W z=2,scan#=6483,scan time=22.4685
AMVYENASR
Intensity
m/z
8.00e+05
6.00e+05
4.00e+05
2.00e+05
0.00e+00
500
1000
1500
C7H8NO2
HexNAc
HexNAc-36
HexNAc-18
C6H8NO2
Hex
a2
b2
b3
y3
HexNAcHex
~y4
~b7
~y7
Pep_1+
Pep+HexNAc_1+
Pep+HexNAc+Fuc_1+
Pep+2HexNAc_1+
Pep+2HexNAc+2Hex_1+

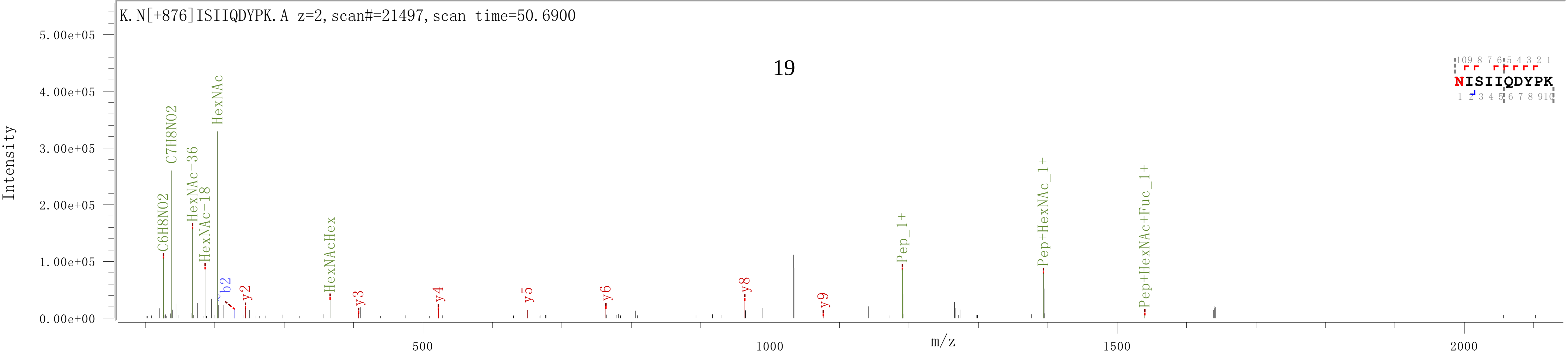
K.N[+876]ISIIQDYPK.A z=2,scan#=21497,scan time=50.6900
19
Intensity
m/z
NISIIQDYPK
C6H8NO2
C7H8NO2
HexNAc-36
HexNAc-18
HexNAc
b2
y2
HexNAcHex
y3
y4
y5
y6
y8
y9
Pep_1+
Pep+HexNAc_1+
Pep+HexNAc+Fuc_1+

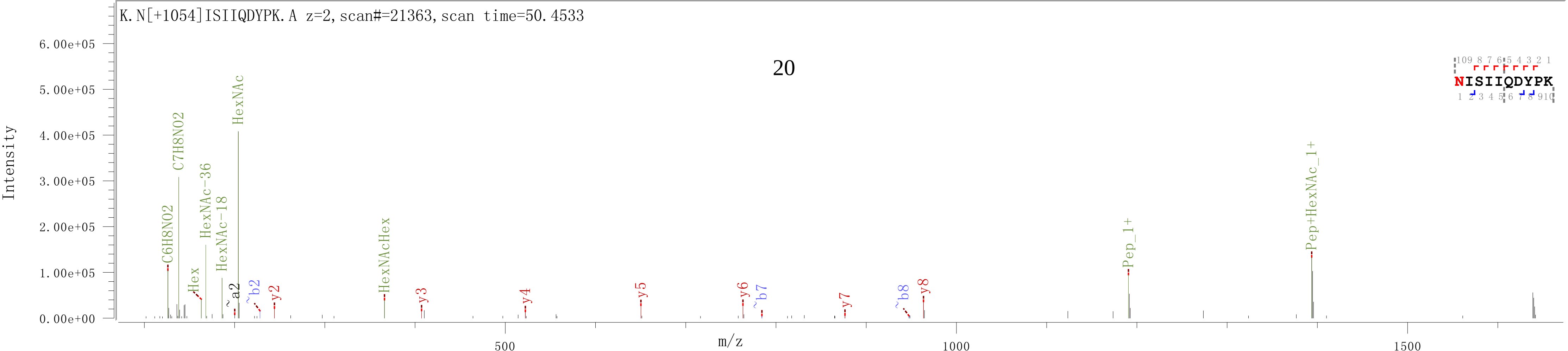
K.N[+1054]ISIIQDYPK.A z=2,scan#=21363,scan time=50.4533
Intensity
m/z
6.00e+05
5.00e+05
4.00e+05
3.00e+05
2.00e+05
1.00e+05
0.00e+00
500
1000
1500
C6H8NO2
C7H8NO2
Hex
HexNAc-36
HexNAc-18
HexNAc
~a2
~b2
y2
HexNAcHex
y3
y4
y5
y6
~b7
y7
~b8
y8
Pep_1+
Pep+HexNAc_1+
NISIIQDYPK

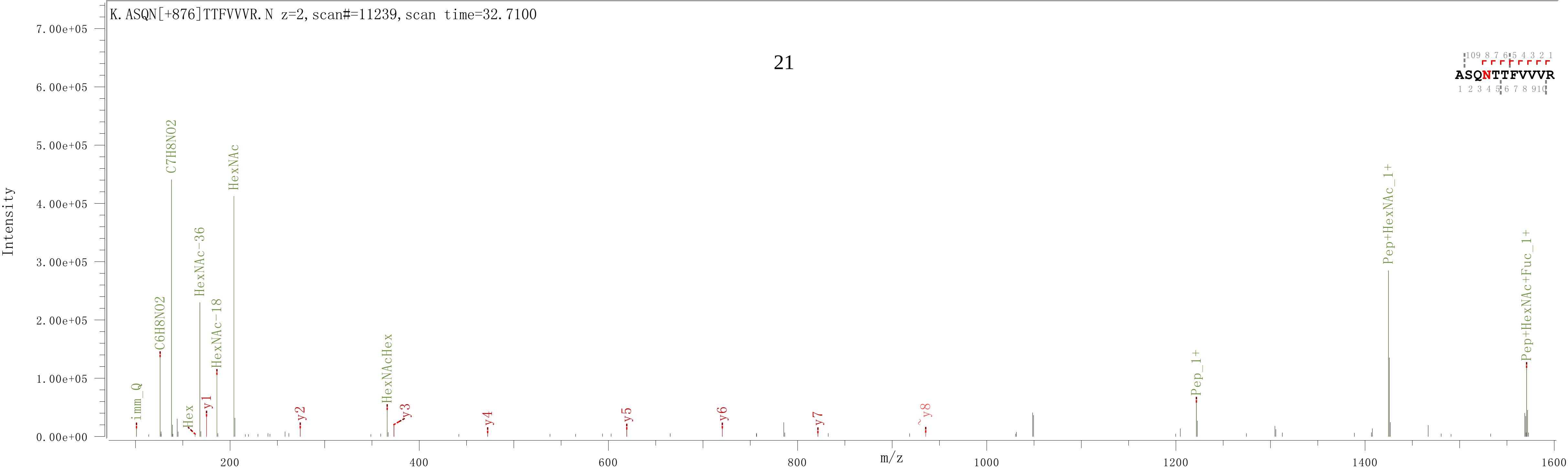

K.ASQN[+876]TTFVVVR.N z=2,scan#=11239,scan time=32.7100
21
ASQNTTFVVVR
Intensity
m/z
imm_Q
C6H8NO2
C7H8NO2
Hex
HexNAc-36
y1
HexNAc-18
HexNAc
y2
HexNAcHex
y3
y4
y5
y6
y7
~y8
Pep_1+
Pep+HexNAc_1+
Pep+HexNAc+Fuc_1+

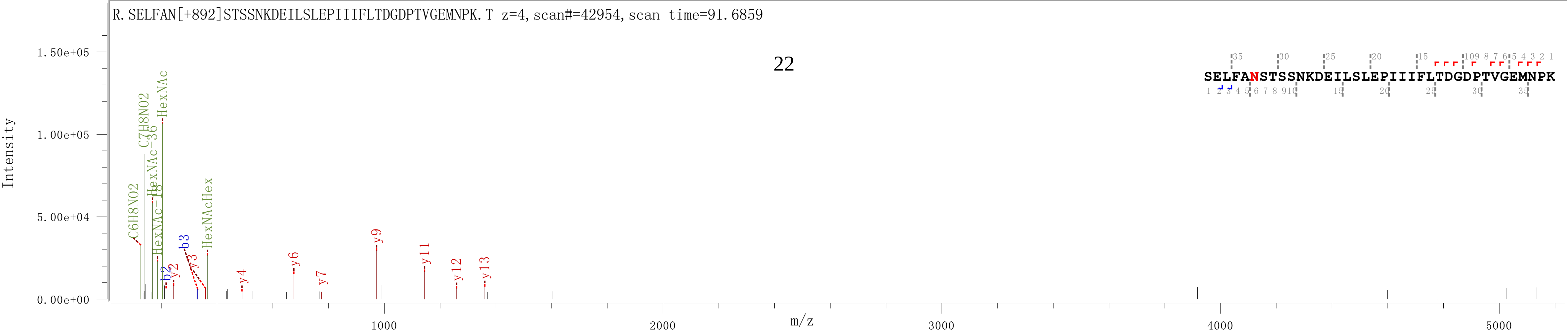
R.SELFAN[+892]STSSNKDEILSLEPIIIFLTDGDPTVGEMNPK.T z=4,scan#=42954,scan time=91.6859
22
SELFANSTSSNKDEILSLEPIIIFLTDGDPTVGEMNPK
Intensity
m/z
C6H8NO2
C7H8NO2
HexNAc-36
HexNAc-18
HexNAc
HexNAcHex
b2
b3
y2
y3
y4
y6
y7
y9
y11
y12
y13

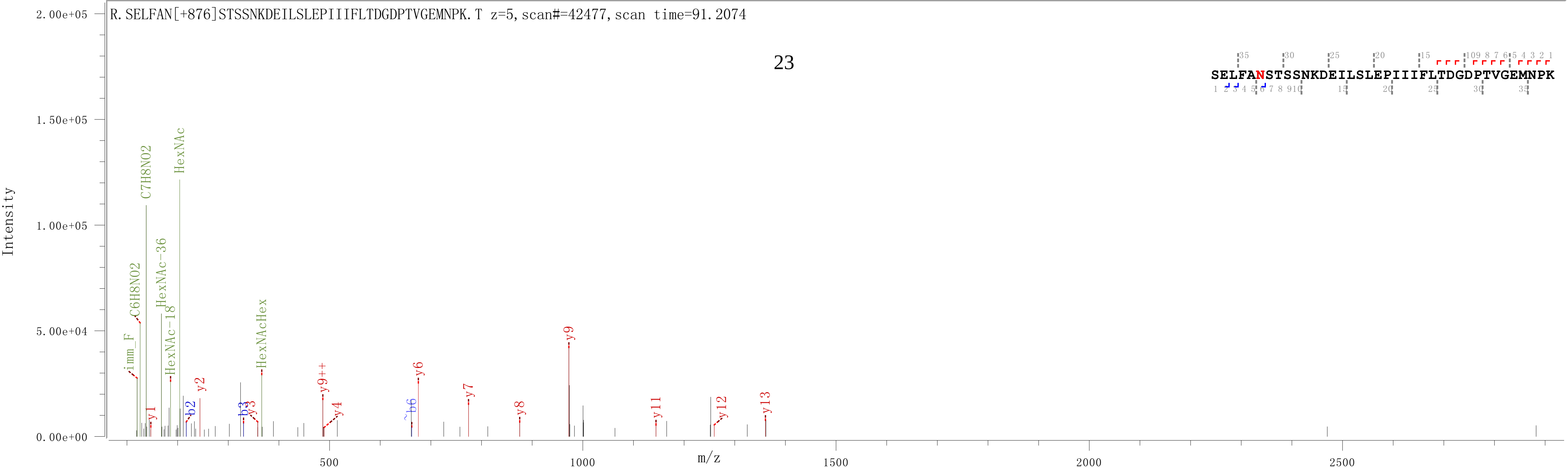
R.SELFAN[+876]STSSNKDEILSLEPIIIFLTDGDPTVGEMNPK.T z=5,scan#=42477,scan time=91.2074
SELFANSTSSNKDEILSLEPIIIFLTDGDPTVGEMNPK
Intensity
m/z
2.00e+05
1.50e+05
1.00e+05
5.00e+04
0.00e+00
500
1000
1500
2000
2500
imm_F
C6H8NO2
C7H8NO2
HexNAc-36
HexNAc-18
HexNAc
HexNAcHex
y1
b2
y2
b3
y3
y4
y9++
~b6
y6
y7
y8
y9
y11
y12
y13

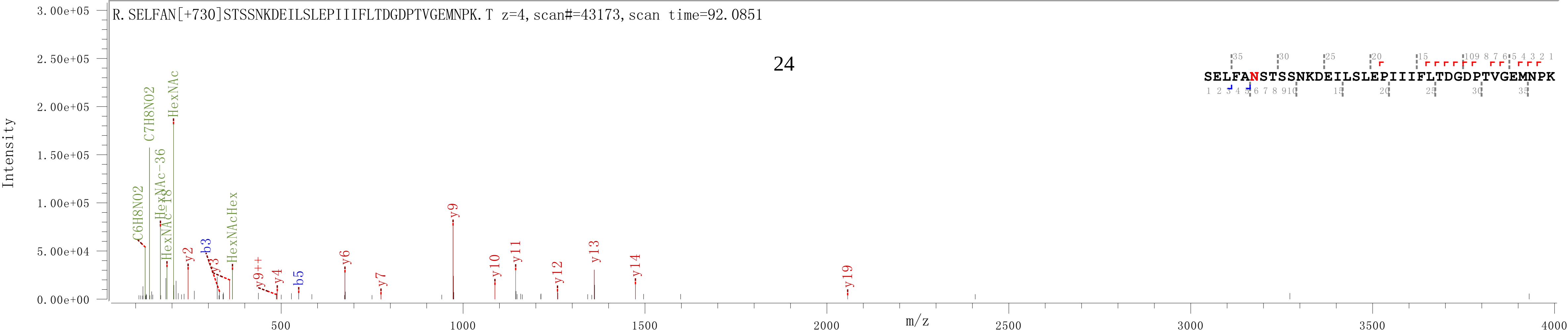
R.SELFAN[+730]STSSNKDEILSLEPIIIFLTDGDPTVGEMNPK.T z=4,scan#=43173,scan time=92.0851
24
SELFANSTSSNKDEILSLEPIIIFLTDGDPTVGEMNPK
Intensity
m/z
C6H8NO2
C7H8NO2
HexNAc-36
HexNAc-18
HexNAc
HexNAcHex
y2
b3
y3
y9++
y4
b5
y6
y7
y9
y10
y11
y12
y13
y14
y19

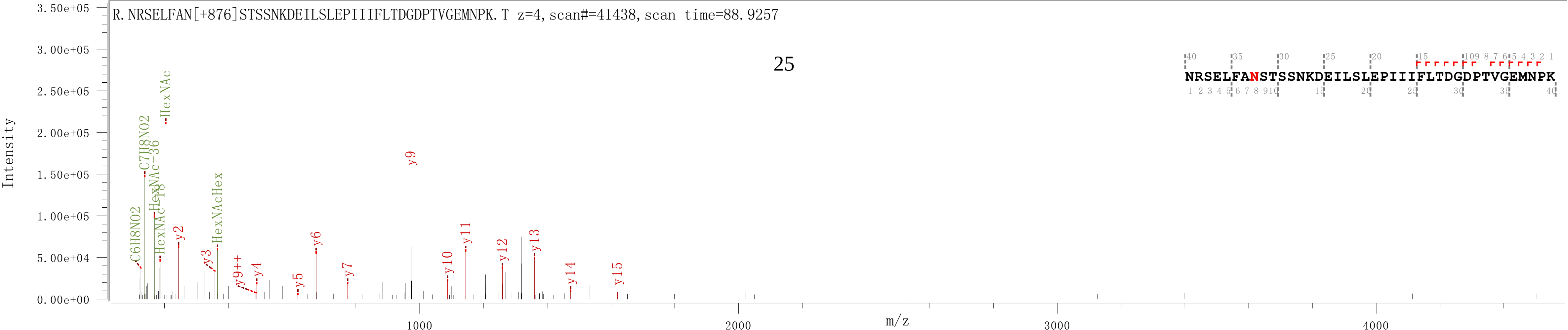

R.NRSELFAN[+876]STSSNKDEILSLEPIIIFLTDGDPTVGEMNPK.T z=4,scan#=41438,scan time=88.9257
25
NRSELFANSTSSNKDEILSLEPIIIFLTDGDPTVGEMNPK
Intensity
m/z
C6H8NO2
C7H8NO2
HexNAc-36
HexNAc-18
HexNAc
HexNAcHex
y2
y3
y9++
y4
y5
y6
y7
y9
y10
y11
y12
y13
y14
y15

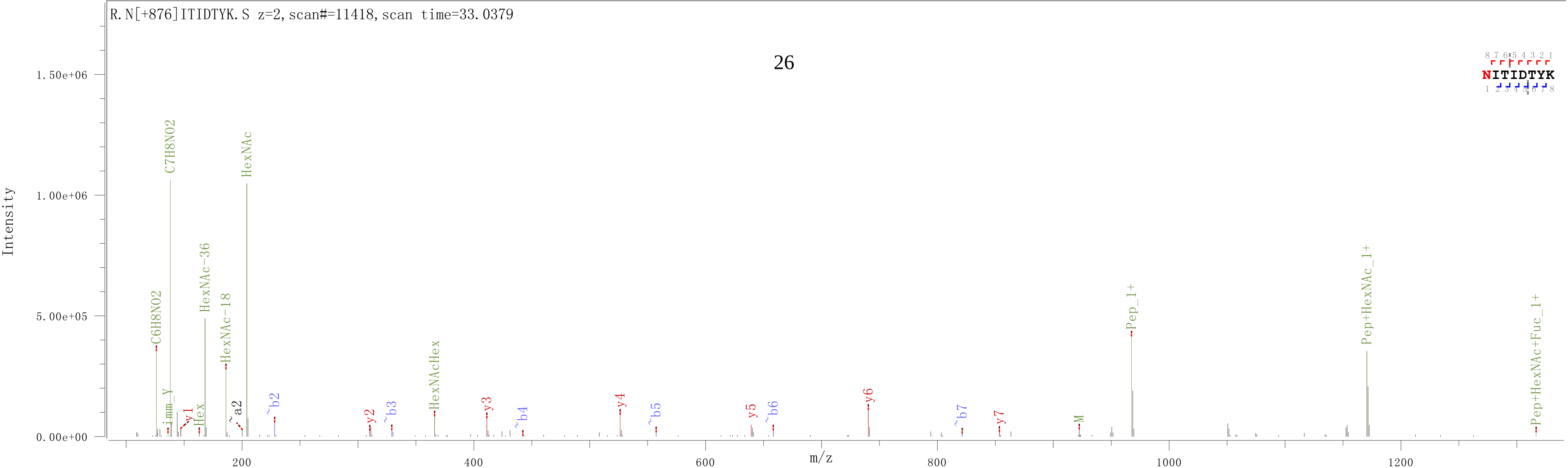

R.N[+876]ITIDTYK.S z=2,scan#=11418,scan time=33.0379
26
NITIDTYK
Intensity
m/z
C6H8NO2
C7H8NO2
HexNAc-36
HexNAc-18
HexNAc
imm_Y
y1
Hex
^a2
^b2
y2
^b3
HexNAcHex
y3
^b4
y4
^b5
y5
^b6
y6
^b7
y7
M
Pep_1+
Pep+HexNAc_1+
Pep+HexNAc+Fuc_1+

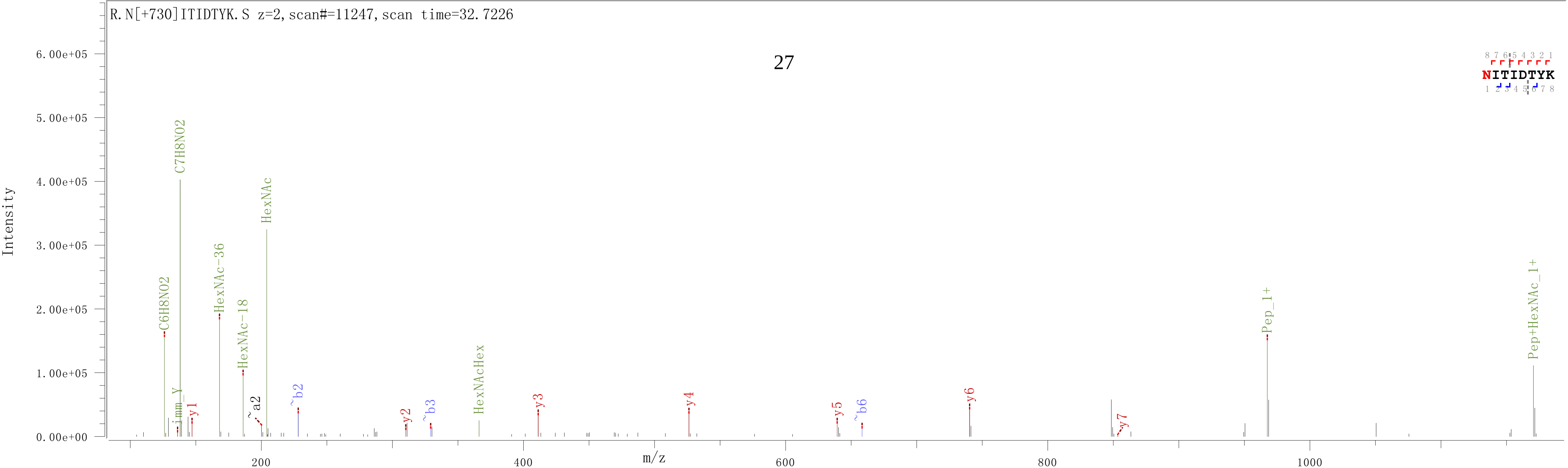
R.N[+730]ITIDTYK.S z=2,scan#=11247,scan time=32.7226
NITIDTYK
8 7 6 5 4 3 2 1
1 2 3 4 5 6 7 8
Intensity
6.00e+05
5.00e+05
4.00e+05
3.00e+05
2.00e+05
1.00e+05
0.00e+00
C6H8NO2
imm_Y
y1
C7H8NO2
HexNAc-36
HexNAc-18
~a2
HexNAc
~b2
y2
~b3
HexNAcHex
y3
y4
y5
~b6
y6
y7
Pep_1+
Pep+HexNAc_1+
200
400
600
800
1000
m/z

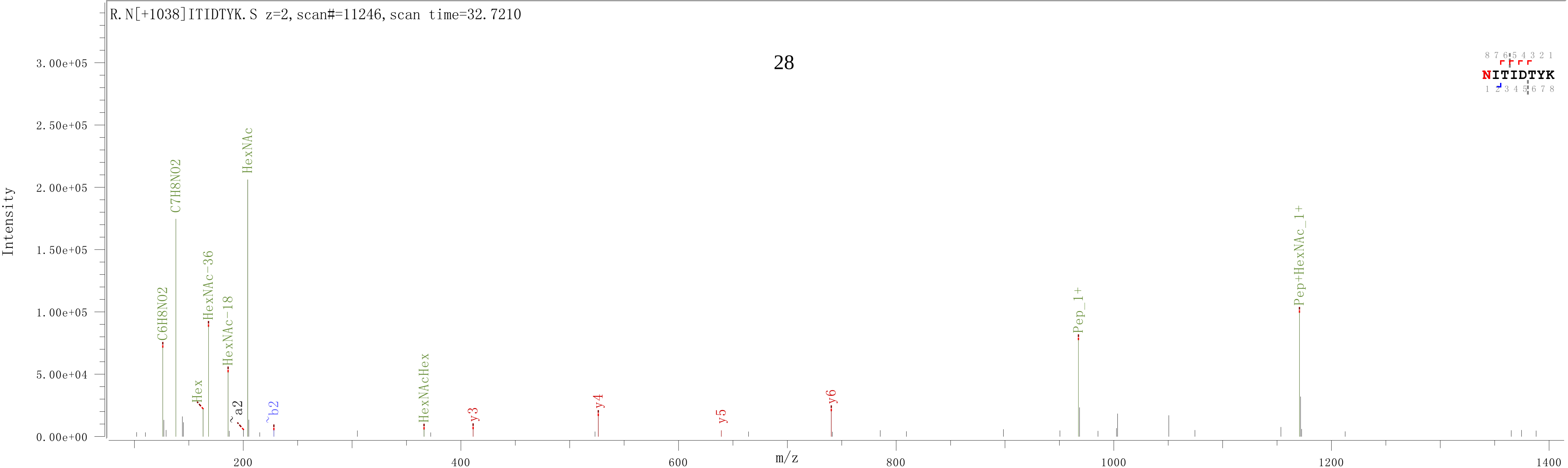

R.N[+1038]ITIDTYK.S z=2,scan#=11246,scan time=32.7210
28
NITIDTYK
Intensity
m/z
C6H8NO2
C7H8NO2
Hex
HexNAc-36
HexNAc-18
~a2
HexNAc
~b2
HexNAcHex
y3
y4
y5
y6
Pep_1+
Pep+HexNAc_1+

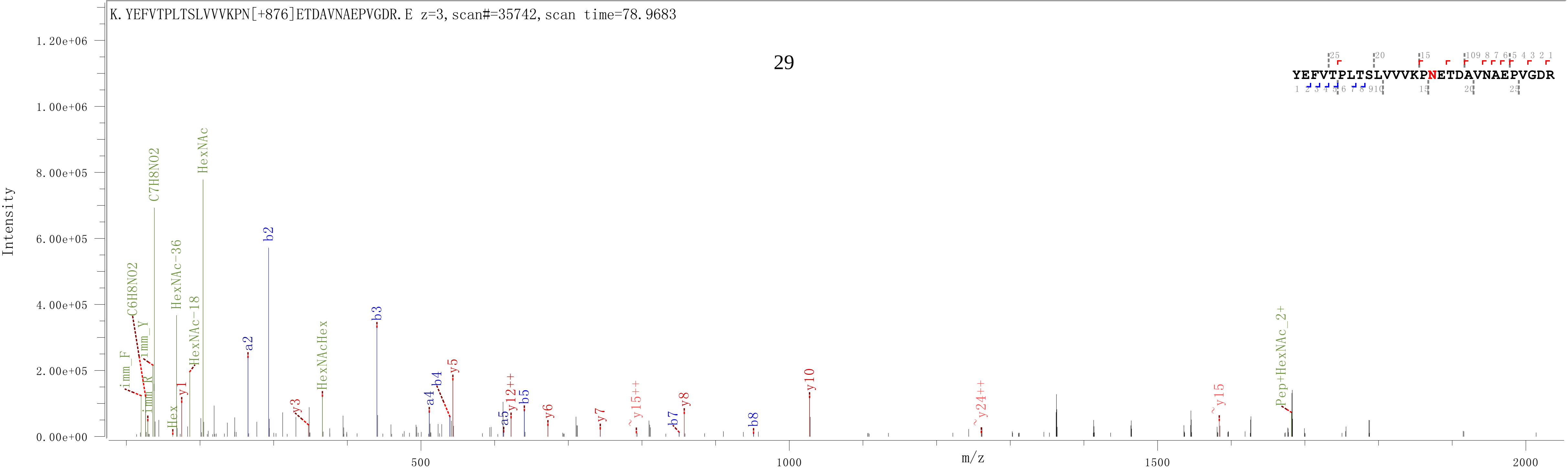

K.YEFVTPLTSLVVVKPN[+876]ETDAVNAEPVGDR.E z=3,scan#=35742,scan time=78.9683
29
YEFVTPLTSLVVVKPNETDAVNAEPVGDR
Intensity
m/z
1.20e+06
1.00e+06
8.00e+05
6.00e+05
4.00e+05
2.00e+05
0.00e+00
500
1000
1500
2000
HexNAc
C7H8NO2
C6H8NO2
imm_F
imm_Y
imm_K
Hex
HexNAc-36
HexNAc-18
y1
a2
b2
y3
HexNAcHex
b3
a4
b4
y5
a5
y12++
b5
y6
y7
~y15++
b7
y8
b8
y10
~y24++
~y15
Pep+HexNAc_2+

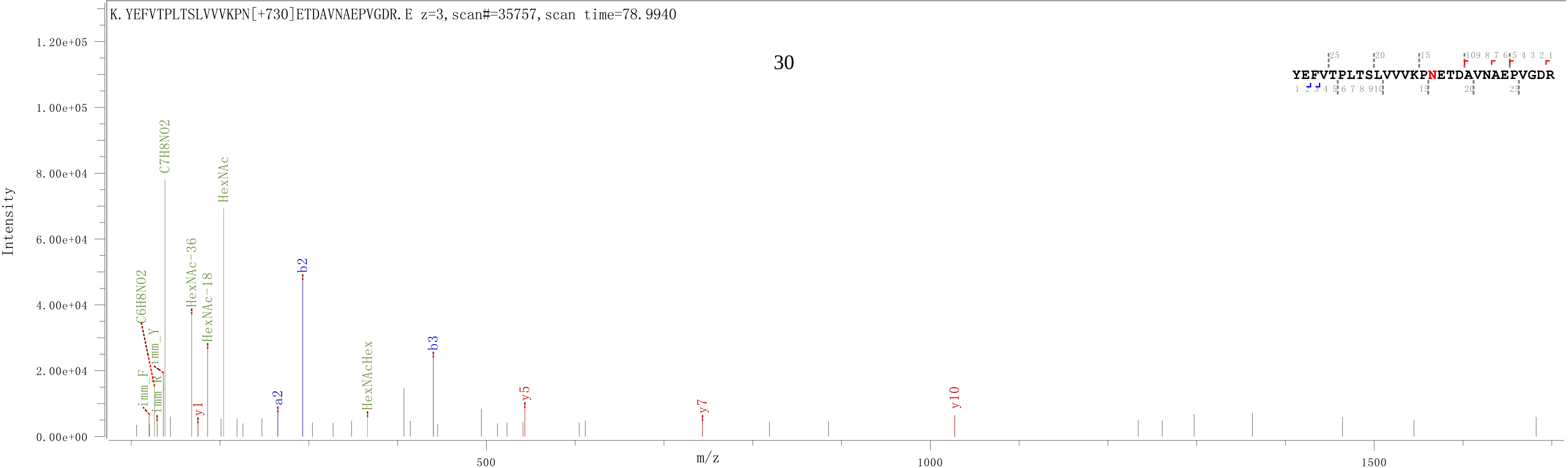
K.YEFVTPLTSLVVVKPN[+730]ETDAVNAEPVGDR.E z=3,scan#=35757,scan time=78.9940
YEFVTPLTSLVVVKPNETDAVNAEPVGDR
Intensity
m/z
C7H8NO2
HexNAc
HexNAc-36
HexNAc-18
C6H8NO2
imm_Y
imm_F
imm_R
y1
a2
b2
HexNAcHex
b3
y5
y7
y10
500
1000
1500

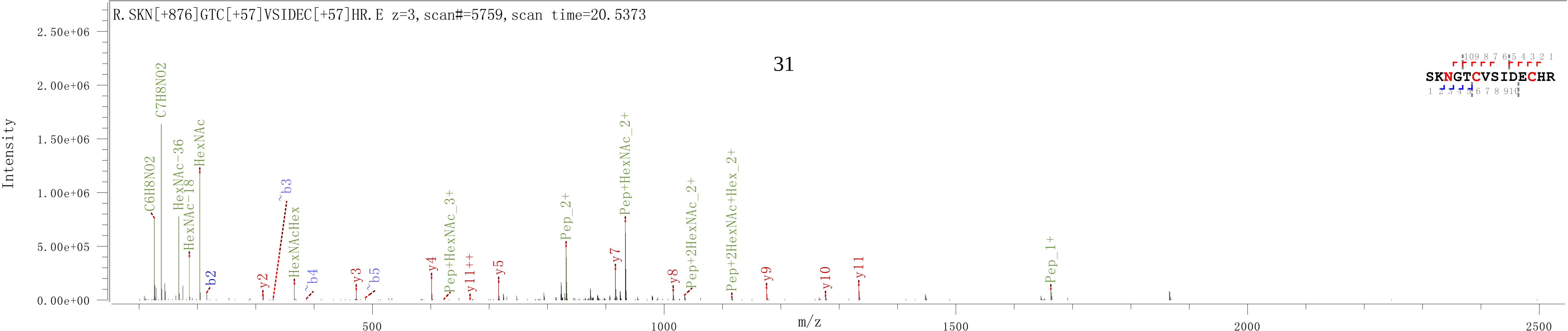
R.SKN[+876]GTC[+57]VSIDEC[+57]HR.E z=3,scan#=5759,scan time=20.5373
31
SKNGTCVSIDECHR
Intensity
m/z
C6H8NO2
C7H8NO2
HexNAc-36
HexNAc-18
HexNAc
b2
y2
~b3
HexNAcHex
~b4
y3
~b5
y4
Pep+HexNAc_3+
y11++
y5
Pep_2+
y7
Pep+HexNAc_2+
y8
Pep+2HexNAc_2+
Pep+2HexNAc+Hex_2+
y9
y10
y11
Pep_1+

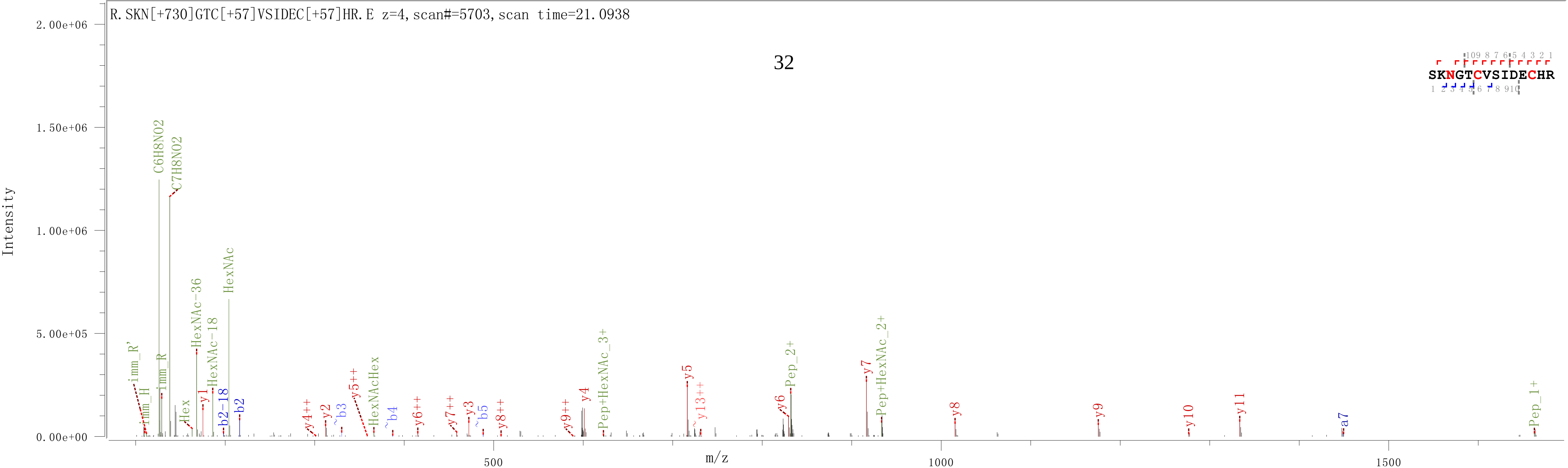
R.SKN[+730]GTC[+57]VSIDEC[+57]HR.E z=4,scan#=5703,scan time=21.0938
SKNGTCVSIDECHR
Intensity
m/z
2.00e+06
1.50e+06
1.00e+06
5.00e+05
0.00e+00
500
1000
1500
C6H8NO2
C7H8NO2
HexNAc
HexNAc-36
HexNAc-18
imm_R'
imm_H
imm_R
Hex
y1
b2-18
b2
y4++
y2
~b3
y5++
HexNAcHex
~b4
y6++
y7++
y3
~b5
y8++
y9++
y4
Pep+HexNAc_3+
y5
y13++
y6
Pep_2+
y7
Pep+HexNAc_2+
y8
y9
y10
y11
a7
Pep_1+

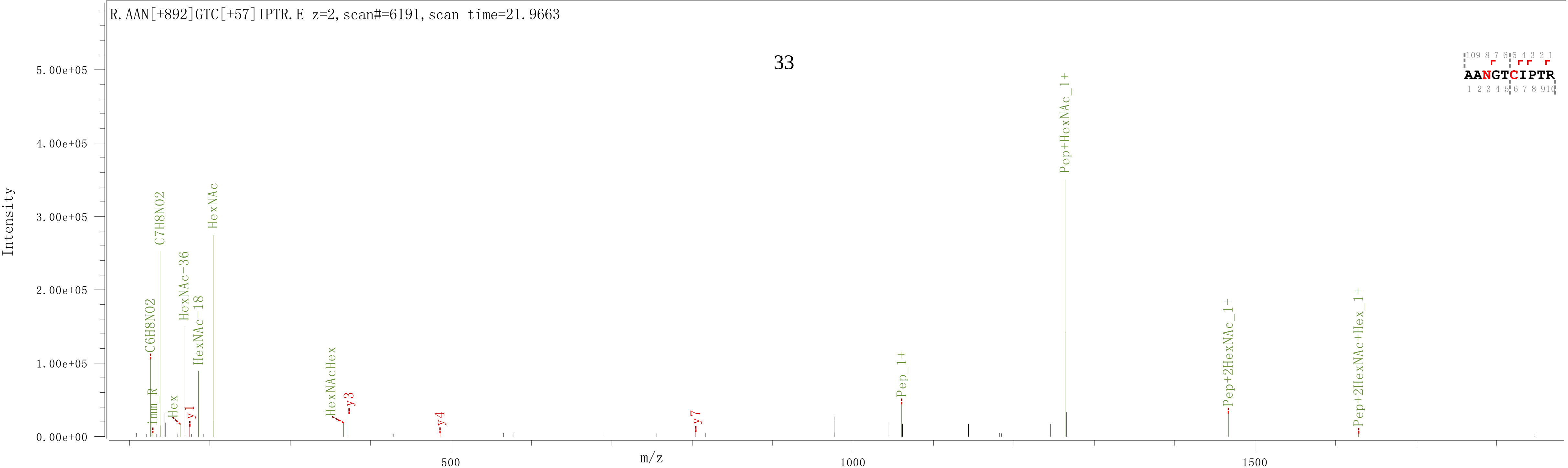
R.AAN[+892]GTC[+57]IPTR.E z=2,scan#=6191,scan time=21.9663
33
AANGTCIPTR
Intensity
m/z
5.00e+05
4.00e+05
3.00e+05
2.00e+05
1.00e+05
0.00e+00
500
1000
1500
C6H8NO2
Imm_R
C7H8NO2
Hex
HexNAc-36
y1
HexNAc-18
HexNAc
HexNAcHex
y3
y4
y7
Pep_1+
Pep+HexNAc_1+
Pep+2HexNAc_1+
Pep+2HexNAc+Hex_1+

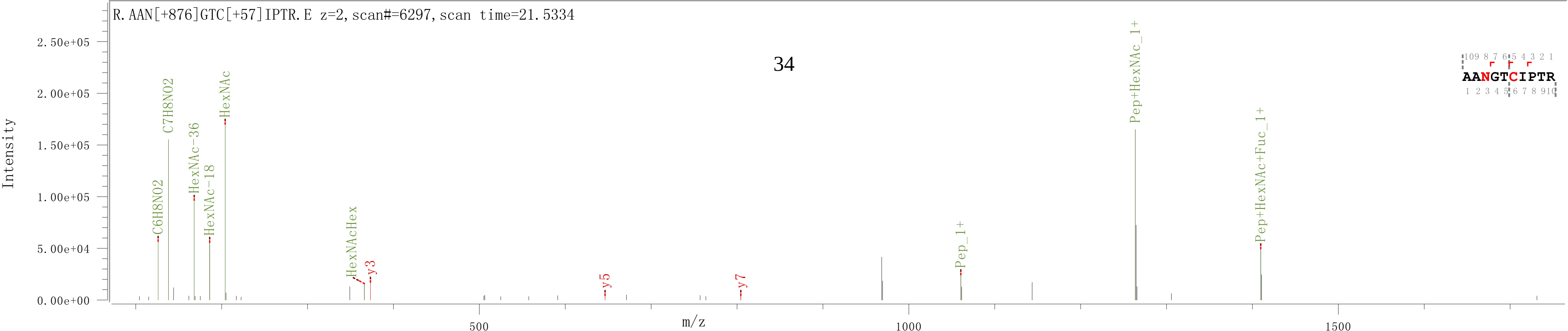
R.AAN[+876]GTC[+57]IPTR.E z=2,scan#=6297,scan time=21.5334
34
Intensity
m/z
C6H8NO2
C7H8NO2
HexNAc-36
HexNAc-18
HexNAc
HexNAcHex
y3
y5
y7
Pep_1+
Pep+HexNAc_1+
Pep+HexNAc+Fuc_1+
AANGTCIPTR

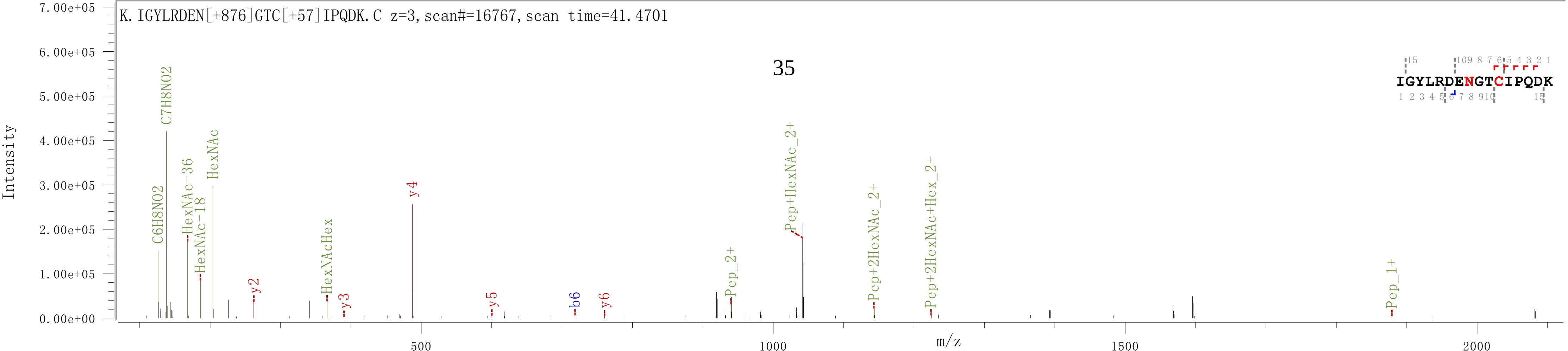
K.IGYLRDEN[+876]GTC[+57]IPQDK.C z=3,scan#=16767,scan time=41.4701
35
IGYLRDENGTCIPQDK
Intensity
m/z
C6H8NO2
C7H8NO2
HexNAc-36
HexNAc-18
HexNAc
y2
HexNAcHex
y3
y4
y5
b6
y6
Pep_2+
Pep+HexNAc_2+
Pep+2HexNAc_2+
Pep+2HexNAc+Hex_2+
Pep_1+

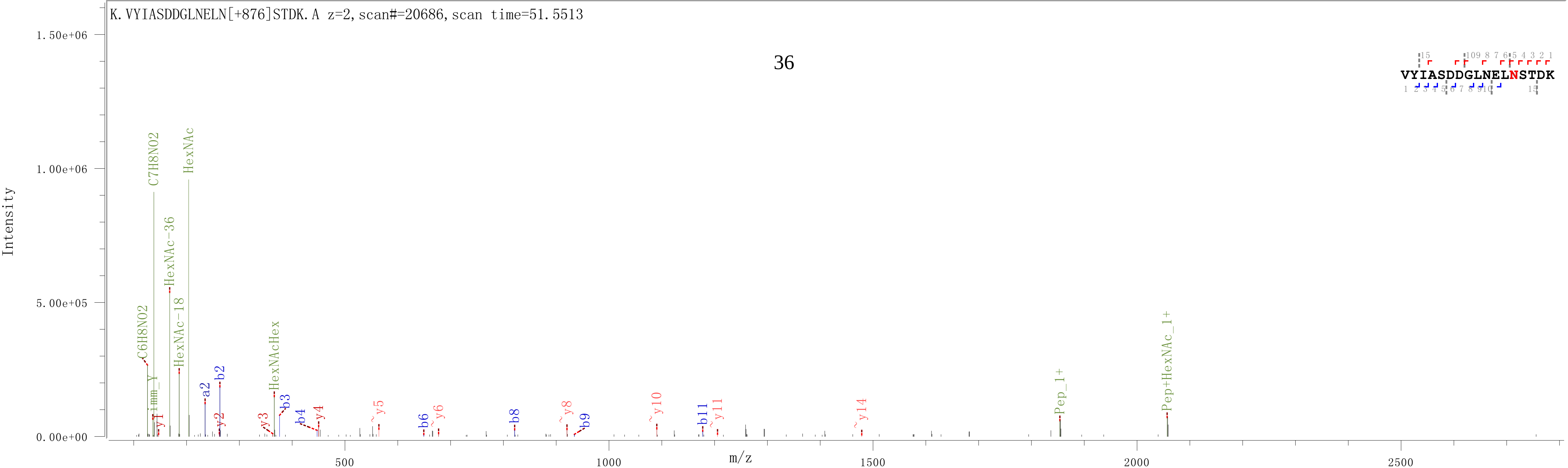
K.VYIASDDGLNELN[+876]STDK.A z=2,scan#=20686,scan time=51.5513
36
VYIASDDGLNELNSTDK
Intensity
1.50e+06
1.00e+06
5.00e+05
0.00e+00
m/z
500
1000
1500
2000
2500
C6H8NO2
C7H8NO2
Imm_Y
y1
HexNAc-36
HexNAc-18
HexNAc
a2
b2
y2
y3
HexNAcHex
b3
b4
y4
~y5
b6
~y6
b8
~y8
b9
~y10
b11
~y11
~y14
Pep_1+
Pep+HexNAc_1+

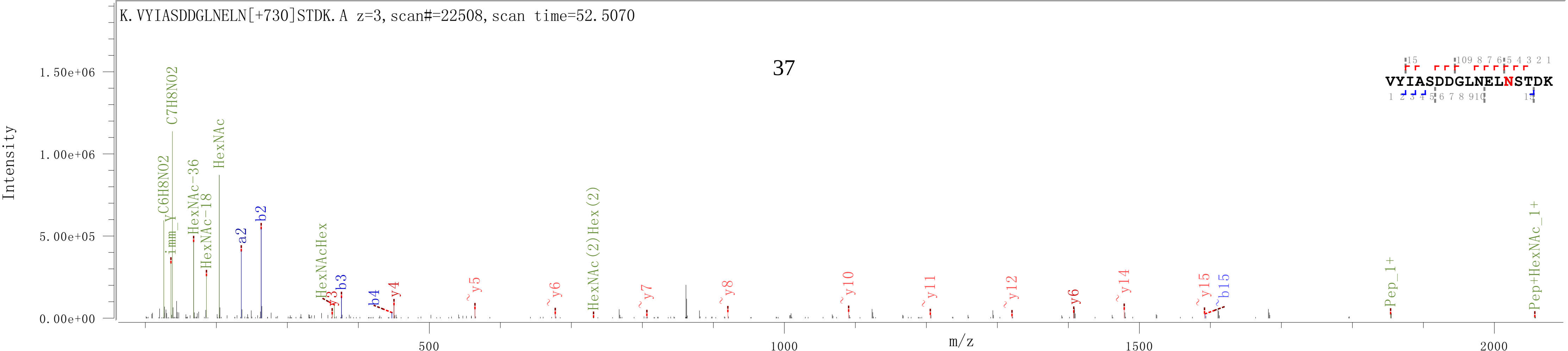
K.VYIASDDGLNELN[+730]STDK.A z=3,scan#=22508,scan time=52.5070
Intensity
m/z
VYIASDDGLNELNSTDK
1.50e+06
1.00e+06
5.00e+05
0.00e+00
500
1000
1500
2000
C7H8NO2
C6H8NO2
imm_Y
HexNAc-36
HexNAc-18
HexNAc
a2
b2
HexNAcHex
y3
b3
b4
y4
~y5
~y6
HexNAc(2)Hex(2)
~y7
~y8
~y10
~y11
~y12
y6
~y14
~y15
~b15
Pep_1+
Pep+HexNAc_1+

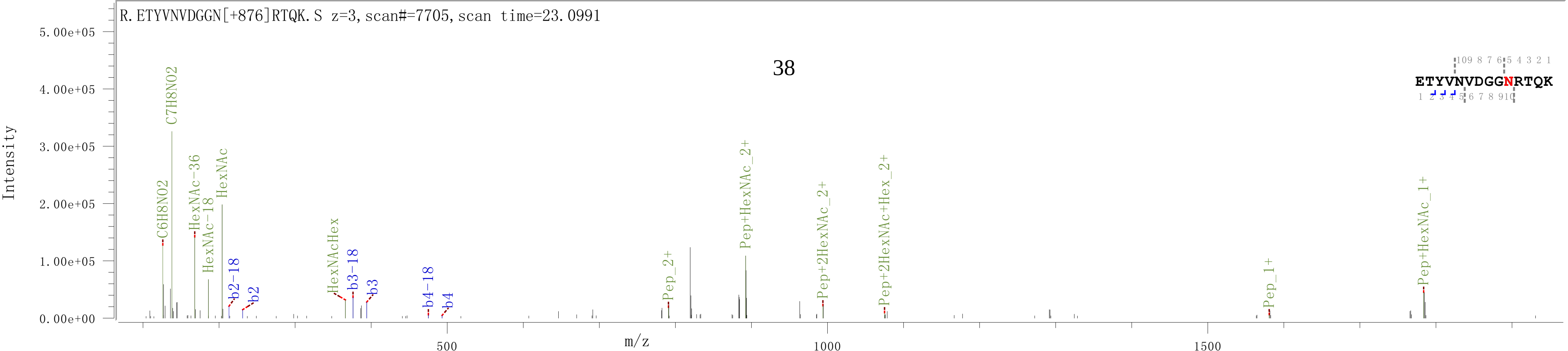
R.ETYVNVDGGN[+876]RTQK.S z=3,scan#=7705,scan time=23.0991
38
Intensity
m/z
5.00e+05
4.00e+05
3.00e+05
2.00e+05
1.00e+05
0.00e+00
500
1000
1500
C6H8NO2
C7H8NO2
HexNAc-36
HexNAc-18
HexNAc
b2-18
b2
HexNAcHex
b3-18
b3
b4-18
b4
Pep_2+
Pep+HexNAc_2+
Pep+2HexNAc_2+
Pep+2HexNAc+Hex_2+
Pep_1+
Pep+HexNAc_1+
ETYVNVDGGNRTQK

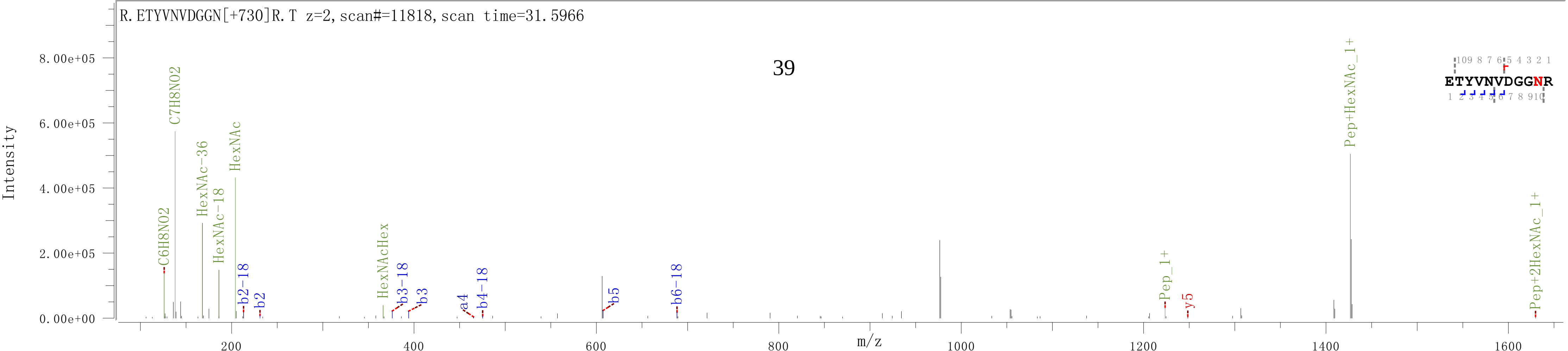
R.ETYVNVDGGN[+730]R.T z=2,scan#=11818,scan time=31.5966
39
Intensity
m/z
C6H8NO2
C7H8NO2
HexNAc-36
HexNAc-18
HexNAc
b2-18
b2
HexNAcHex
b3-18
b3
a4
b4-18
b5
b6-18
Pep_1+
y5
Pep+HexNAc_1+
Pep+2HexNAc_1+
ETYVNVDGGNR

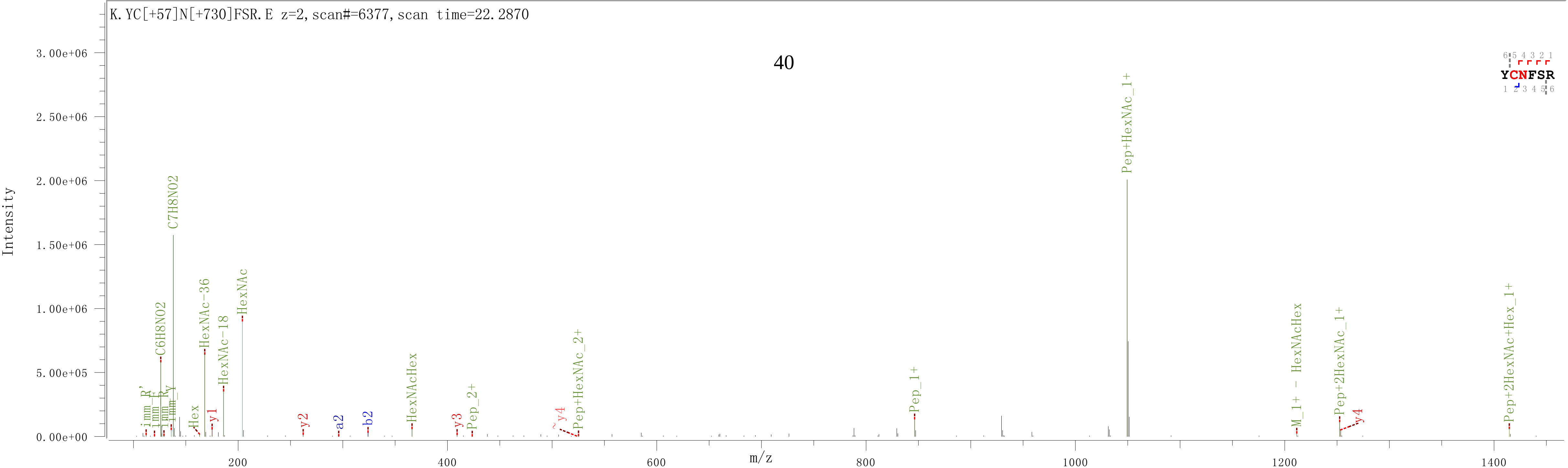

K.YC[+57]N[+730]FSR.E z=2,scan#=6377,scan time=22.2870
40
YCNFSR
Intensity
m/z
Pep+HexNAc_1+
C7H8NO2
C6H8NO2
HexNAc-36
HexNAc-18
HexNAc
HexNAcHex
Hex
y1
y2
a2
b2
y3
Pep_2+
y4
Pep+HexNAc_2+
Pep_1+
M_1+ - HexNAcHex
Pep+2HexNAc_1+
Pep+2HexNAc+Hex_1+

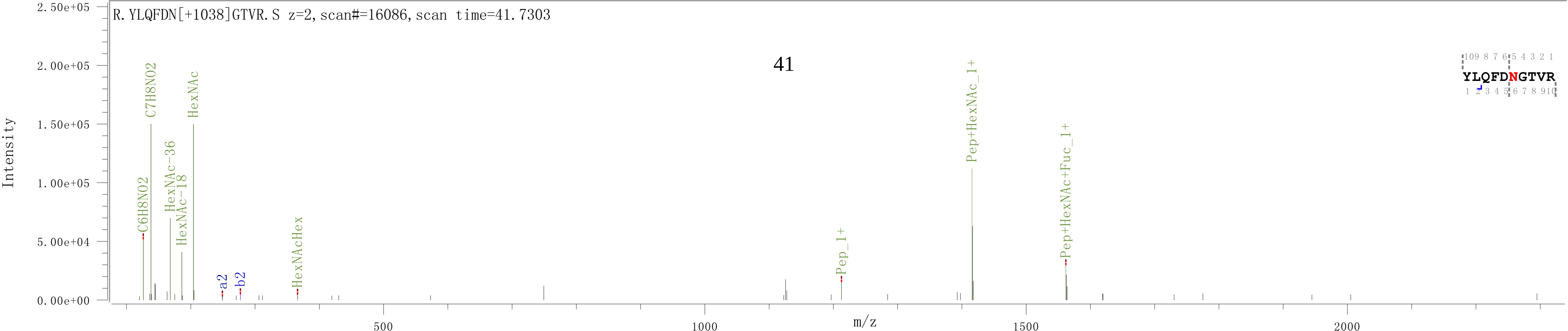
R.YLQFDN[+1038]GTVR.S z=2,scan#=16086,scan time=41.7303
Intensity
2.50e+05
2.00e+05
1.50e+05
1.00e+05
5.00e+04
0.00e+00
m/z
500
1000
1500
2000
C6H8NO2
C7H8NO2
HexNAc-36
HexNAc-18
HexNAc
a2
b2
HexNAcHex
Pep_1+
Pep+HexNAc_1+
Pep+HexNAc+Fuc_1+
YLQFDNGTVR
10 9 8 7 6 5 4 3 2 1
1 2 3 4 5 6 7 8 9 10

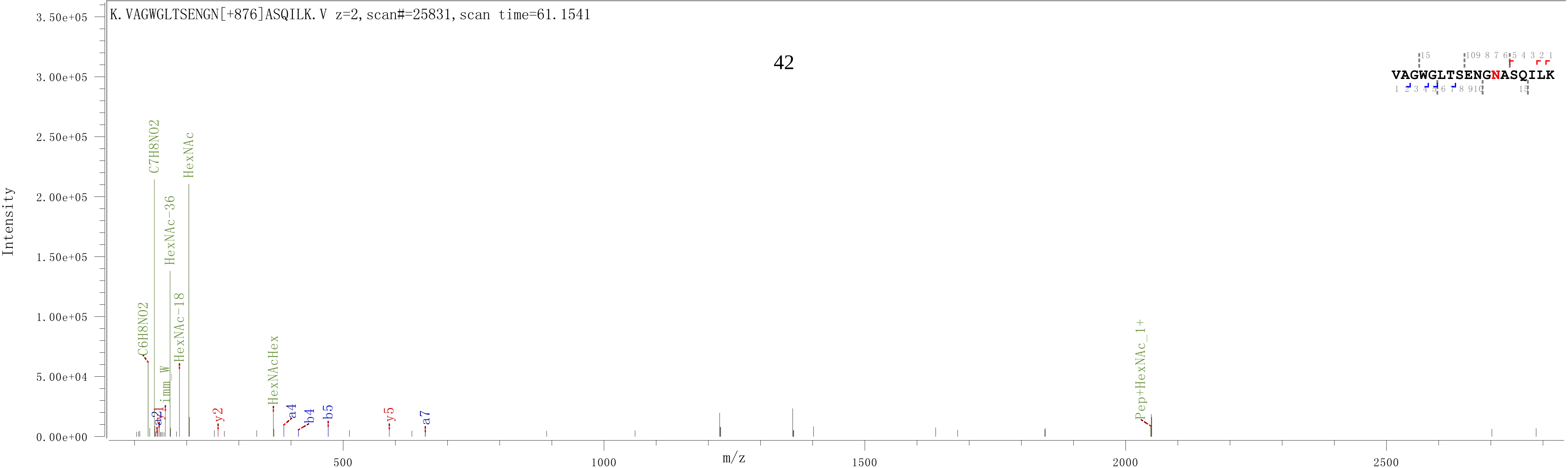

K.VAGWGLTSENGN[+876]ASQILK.V z=2,scan#=25831,scan time=61.1541
42
VAGWGLTSENGNASQILK
Intensity
m/z
C6H8NO2
C7H8NO2
HexNAc-36
HexNAc-18
HexNAc
HexNAcHex
Pep+HexNAc_1+
y2
y5
a4
b4
b5
a7

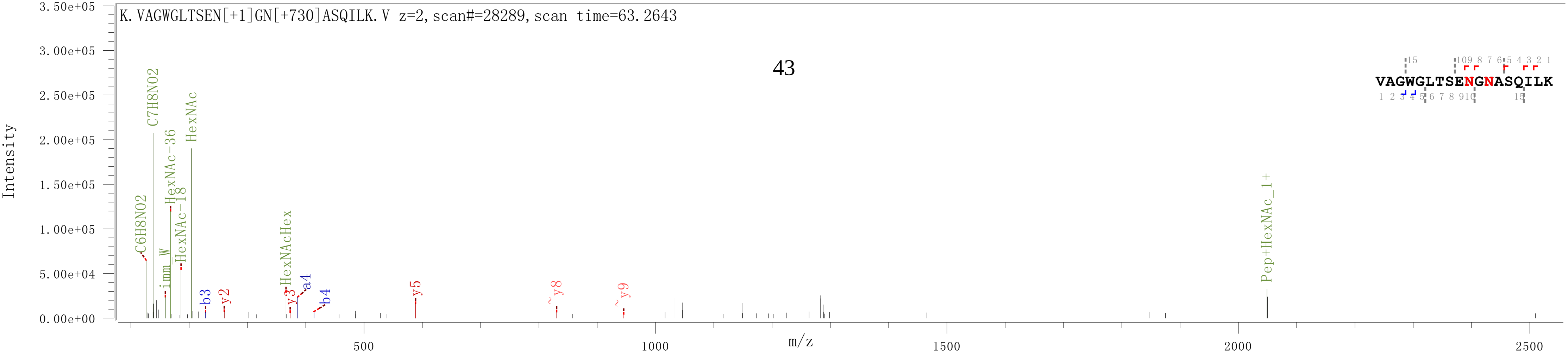
K.VAGWGLTSEN[+1]GN[+730]ASQILK.V z=2,scan#=28289,scan time=63.2643
43
VAGWGLTSENGNASQILK
Intensity
m/z
C6H8NO2
C7H8NO2
imm_W
HexNAc-36
HexNAc-18
HexNAc
b3
y2
HexNAcHex
y3
a4
b4
y5
~y8
~y9
Pep+HexNAc_1+
0.00e+00
5.00e+04
1.00e+05
1.50e+05
2.00e+05
2.50e+05
3.00e+05
3.50e+05
500
1000
1500
2000
2500

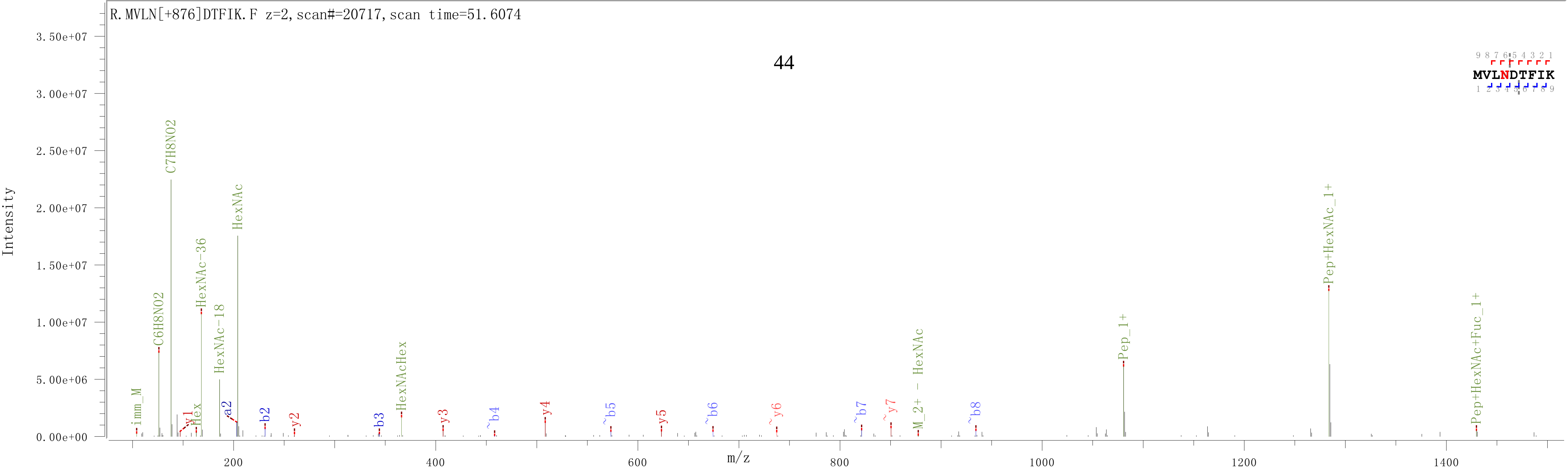
R.MVLN[+876]DTFIK.F z=2,scan#=20717,scan time=51.6074
MVLNDTFIK
Intensity
m/z
imm_M
C6H8NO2
C7H8NO2
y1
Hex
HexNAc-36
HexNAc-18
a2
HexNAc
b2
y2
b3
HexNAcHex
y3
~b4
y4
~b5
y5
~b6
~y6
~b7
~y7
M_2+ - HexNAc
~b8
Pep_1+
Pep+HexNAc_1+
Pep+HexNAc+Fuc_1+

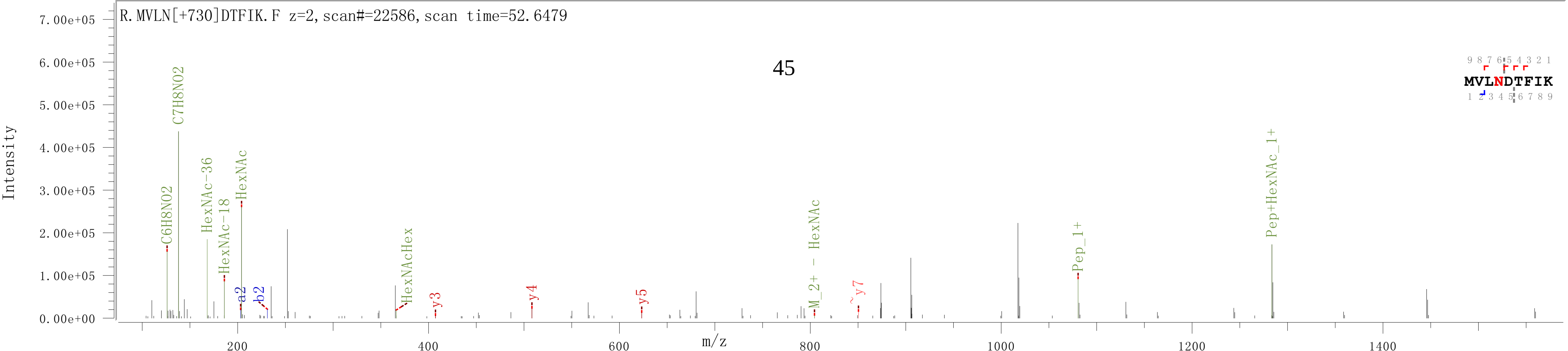
R.MVLN[+730]DTFIK.F z=2,scan#=22586,scan time=52.6479
45
MVLNDTFIK
Intensity
m/z
C6H8NO2
C7H8NO2
HexNAc-36
HexNAc-18
HexNAc
a2
b2
HexNAcHex
y3
y4
y5
M_2+ - HexNAc
~y7
Pep_1+
Pep+HexNAc_1+

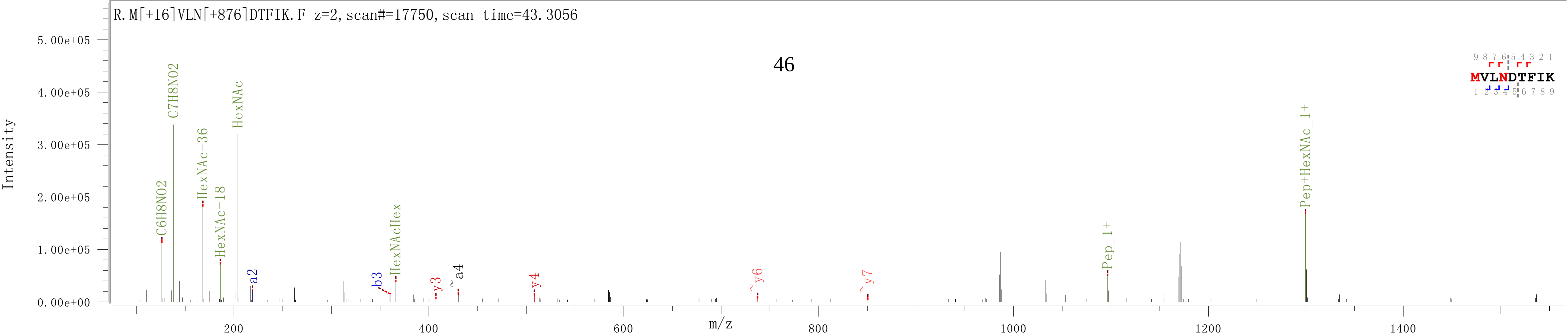

R.M[+16]VLN[+876]DTFIK.F z=2,scan#=17750,scan time=43.3056
46
Intensity
m/z
MVLNDTFIK
C6H8NO2
C7H8NO2
HexNAc-36
HexNAc-18
HexNAc
a2
b3
HexNAcHex
y3
~a4
y4
~y6
~y7
Pep_1+
Pep+HexNAc_1+

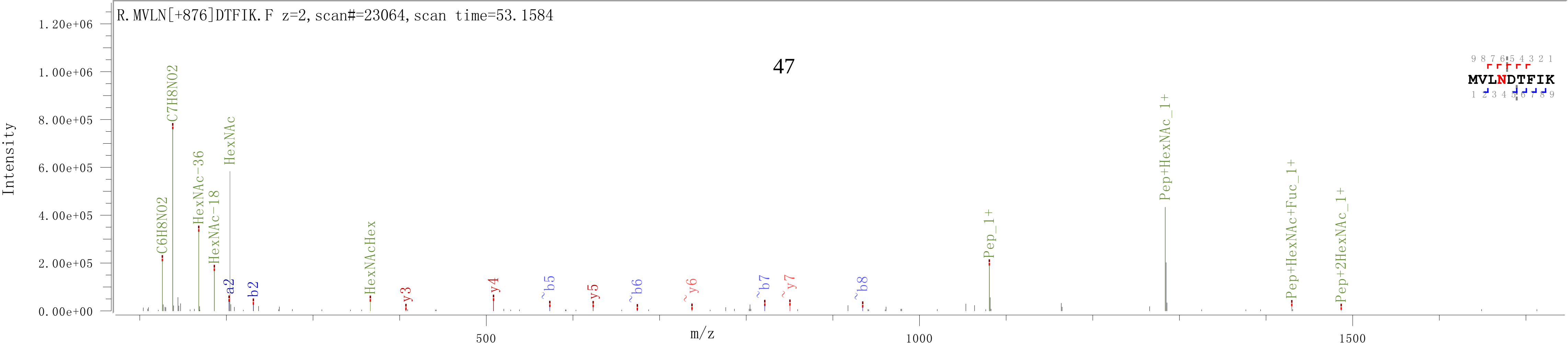
R.MVLN[+876]DTFIK.F z=2,scan#=23064,scan time=53.1584
47
Intensity
m/z
1.20e+06
1.00e+06
8.00e+05
6.00e+05
4.00e+05
2.00e+05
0.00e+00
500
1000
1500
C6H8NO2
C7H8NO2
HexNAc-36
HexNAc-18
HexNAc
a2
b2
HexNAcHex
y3
y4
~b5
y5
~b6
~y6
~b7
~y7
~b8
Pep_1+
Pep+HexNAc_1+
Pep+HexNAc+Fuc_1+
Pep+2HexNAc_1+
MVLNDTFIK

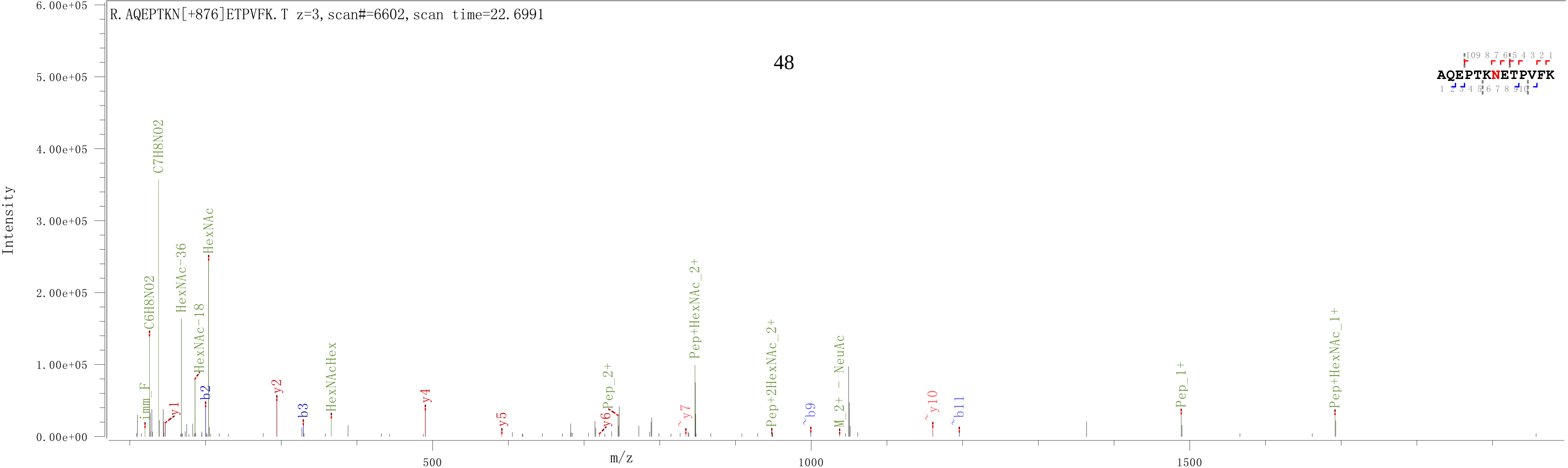
R.AQEPTKN[+876]ETPVFK.T z=3,scan#=6602,scan time=22.6991
AQEPTKNETPVFK
Intensity
m/z
6.00e+05
5.00e+05
4.00e+05
3.00e+05
2.00e+05
1.00e+05
0.00e+00
500
1000
1500
C7H8NO2
C6H8NO2
HexNAc-36
HexNAc-18
HexNAc
imm_F
y1
b2
y2
b3
HexNAcHex
y4
y5
y6
Pep_2+
~y7
Pep+HexNAc_2+
Pep+2HexNAc_2+
~b9
M_2+ - NeuAc
~y10
~b11
Pep_1+
Pep+HexNAc_1+

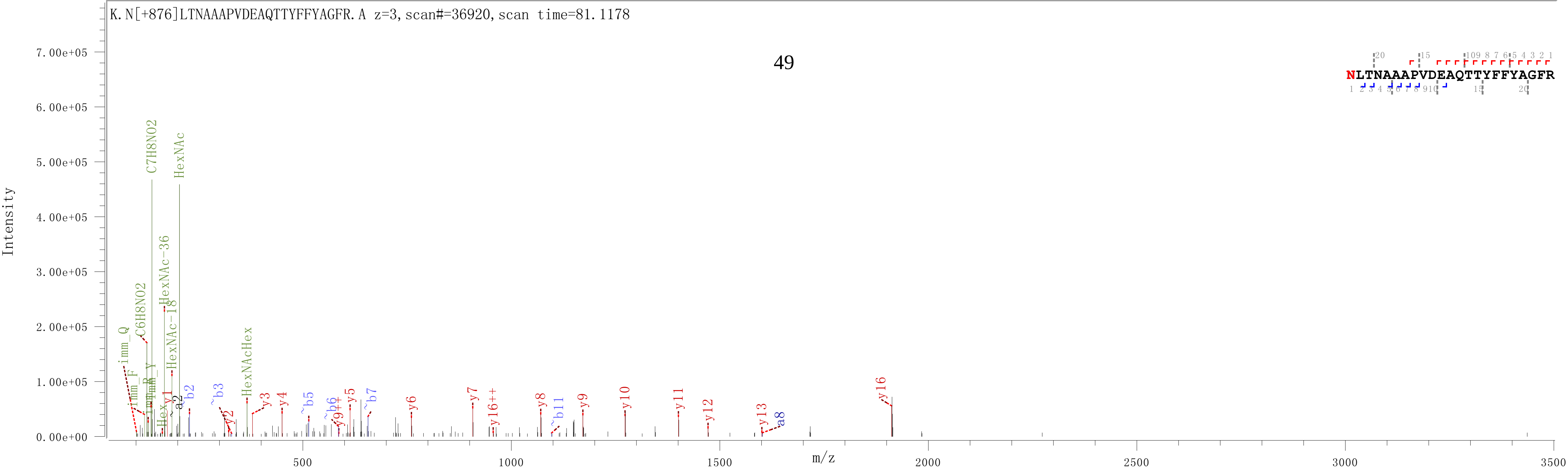
K.N[+876]LTNAAAPVDEAQTTYFFYAGFR.A z=3,scan#=36920,scan time=81.1178
NLTNAAAPVDEAQTTYFFYAGFR
Intensity
m/z
C7H8NO2
HexNAc
HexNAc-36
HexNAc-18
C6H8NO2
imm_Q
imm_F
HexNAcHex
y1
y2
y3
y4
y5
y6
y7
y8
y9
y10
y11
y12
y13
y16
y16++
y9++
~b2
~b3
~b5
~b6
~b7
~b11
a2
a8

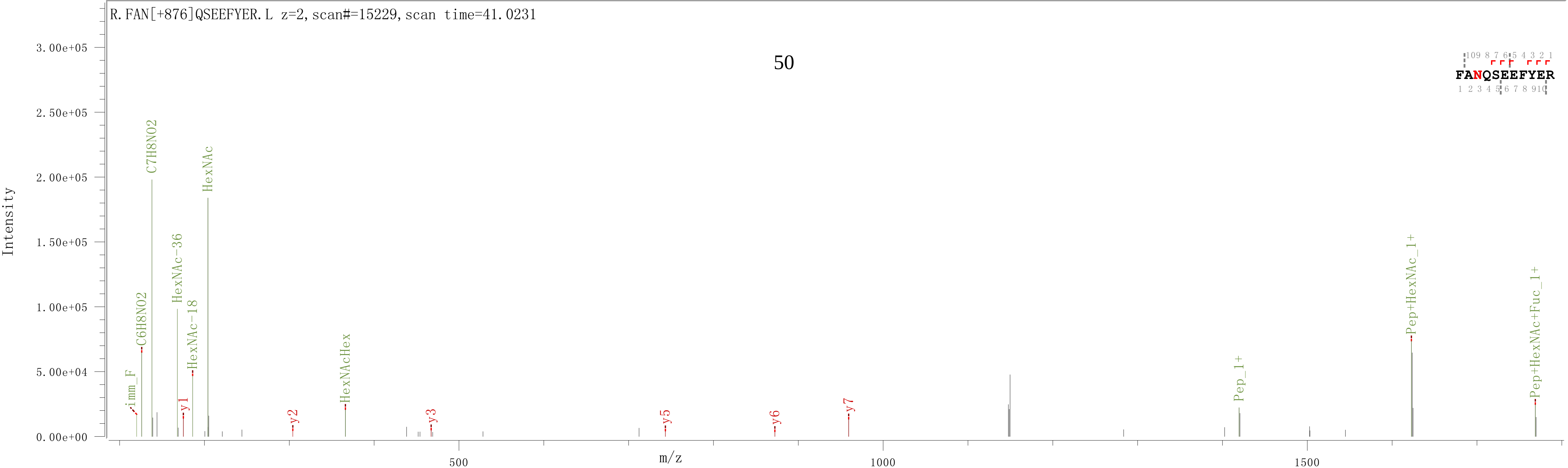
R.FAN[+876]QSEEFYER.L z=2,scan#=15229,scan time=41.0231
50
FANQSEEFYER
Intensity
m/z
imm_F
C6H8NO2
C7H8NO2
HexNAc-36
y1
HexNAc-18
HexNAc
y2
HexNAcHex
y3
y5
y6
y7
Pep_1+
Pep+HexNAc_1+
Pep+HexNAc+Fuc_1+

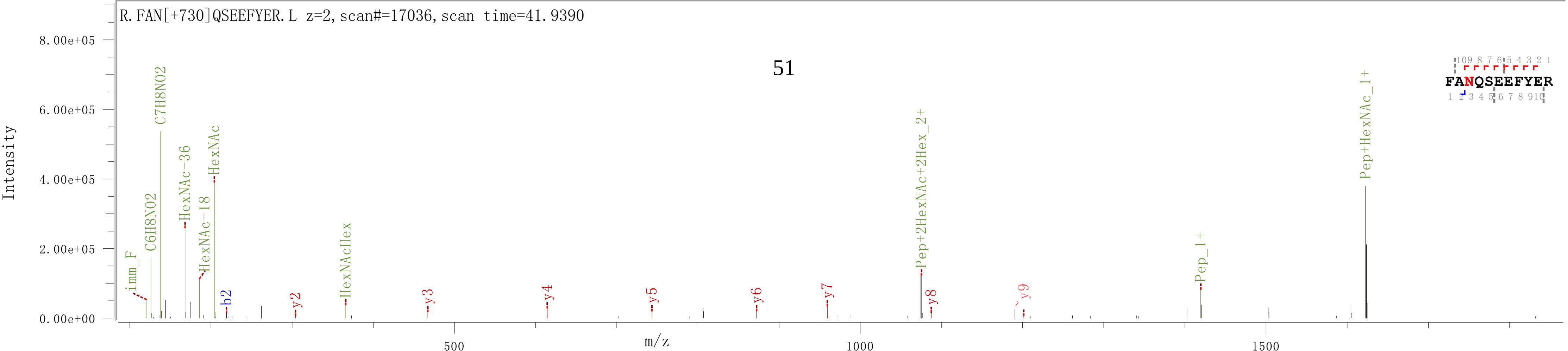

R.FAN[+730]QSEEFYER.L z=2,scan#=17036,scan time=41.9390
51
Intensity
m/z
8.00e+05
6.00e+05
4.00e+05
2.00e+05
0.00e+00
500
1000
1500
imm_F
C6H8NO2
C7H8NO2
HexNAc-36
HexNAc-18
HexNAc
b2
y2
HexNAcHex
y3
y4
y5
y6
y7
Pep+2HexNAc+2Hex_2+
y8
~y9
Pep_1+
Pep+HexNAc_1+
FANQSEEFYER

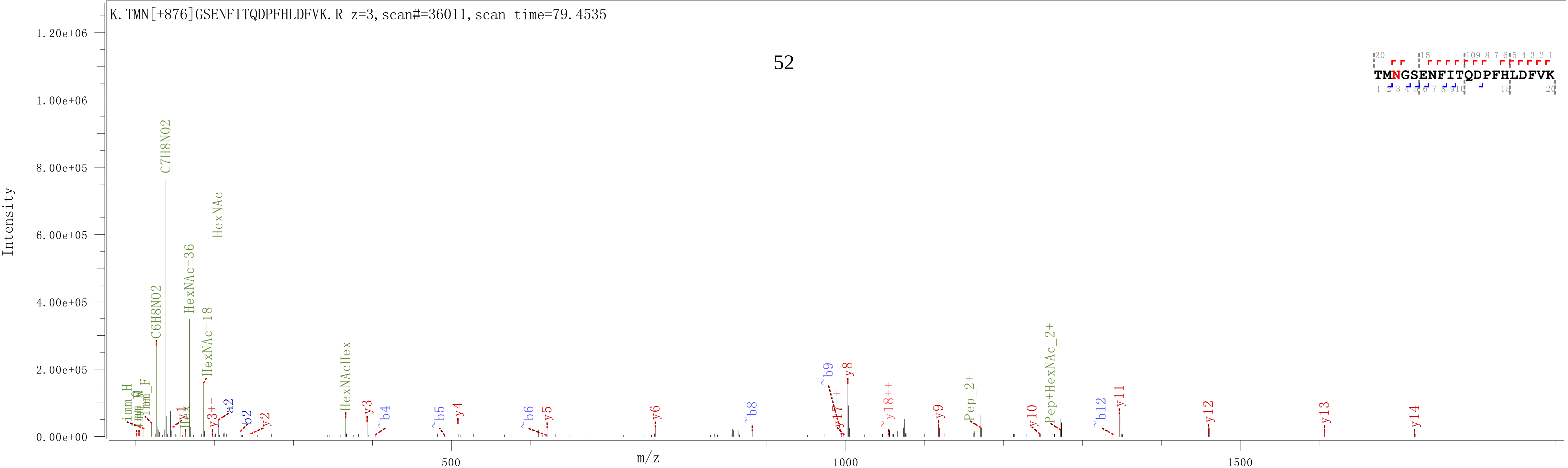
K.TMN[+876]GSENFITQDPFHLDFVK.R z=3,scan#=36011,scan time=79.4535
52
TMNGSENFITQDPFHLDFVK
Intensity
m/z
C7H8NO2
HexNAc
HexNAc-36
HexNAc-18
C6H8NO2
HexNAcHex
Pep_2+
Pep+HexNAc_2+
a2
b2
y2
y3
y3++
~b4
~b5
y4
~b6
y5
y6
~b8
~b9
y8
~y18++
y9
y10
~b12
y11
y12
y13
y14

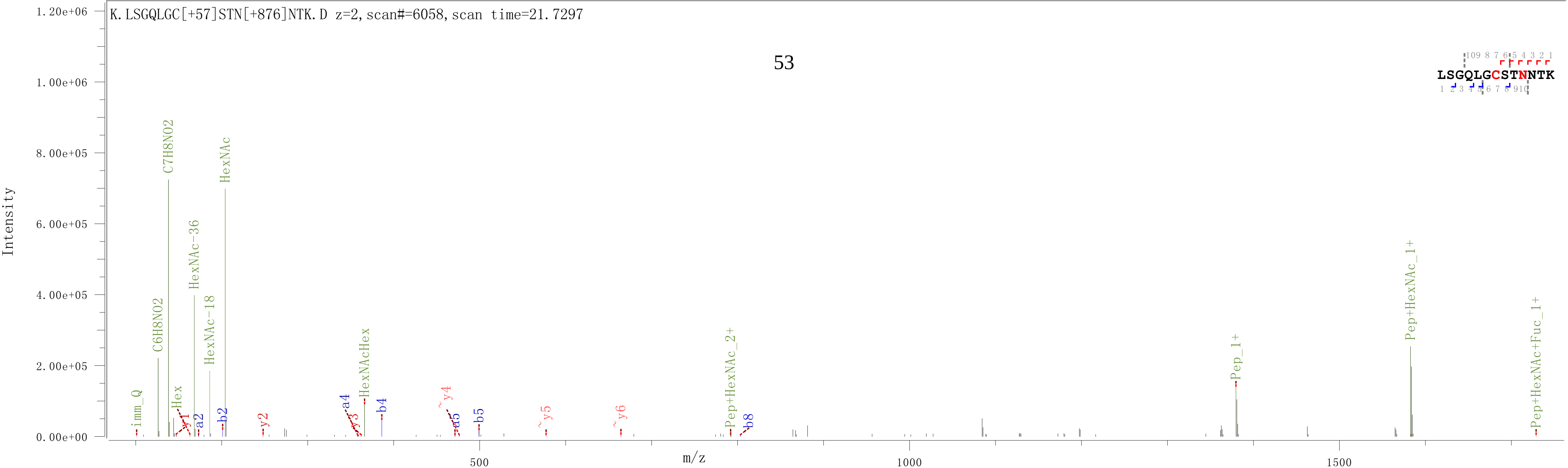

K.LSGQLGC[+57]STN[+876]NTK.D z=2,scan#=6058,scan time=21.7297
53
LSGQLGCSTNNTK
Intensity
m/z
C7H8NO2
HexNAc
HexNAc-36
HexNAc-18
C6H8NO2
Hex
imm_Q
HexNAcHex
Pep+HexNAc_2+
Pep_1+
Pep+HexNAc_1+
Pep+HexNAc+Fuc_1+

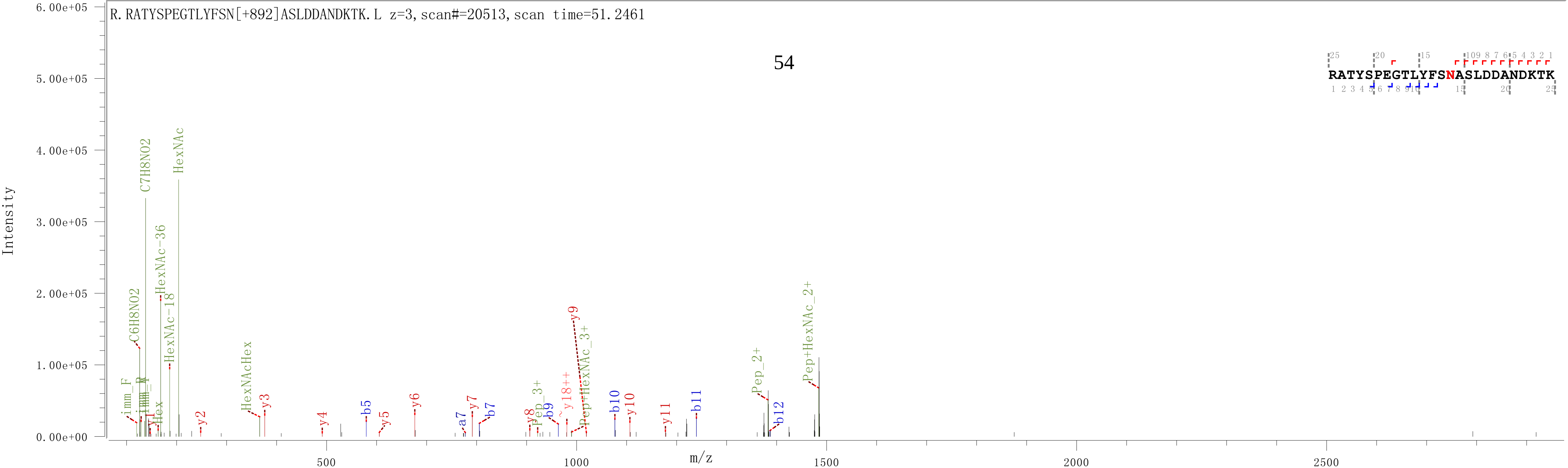
R.RATYSPEGTLYFSN[+892]ASLDDANDKTK.L z=3,scan#=20513,scan time=51.2461
54
RATYSPEGTLYFSNASLDDANDKTK
Intensity
m/z
HexNAc
C7H8NO2
HexNAc-36
HexNAc-18
C6H8NO2
imm_F
HexNAcHex
y9
Pep+HexNAc_3+
y18++
Pep_3+
Pep_2+
Pep+HexNAc_2+
y2
y3
y4
b5
y5
y6
a7
y7
b7
y8
b9
b10
y10
y11
b11
b12
6.00e+05
5.00e+05
4.00e+05
3.00e+05
2.00e+05
1.00e+05
0.00e+00
500
1000
1500
2000
2500

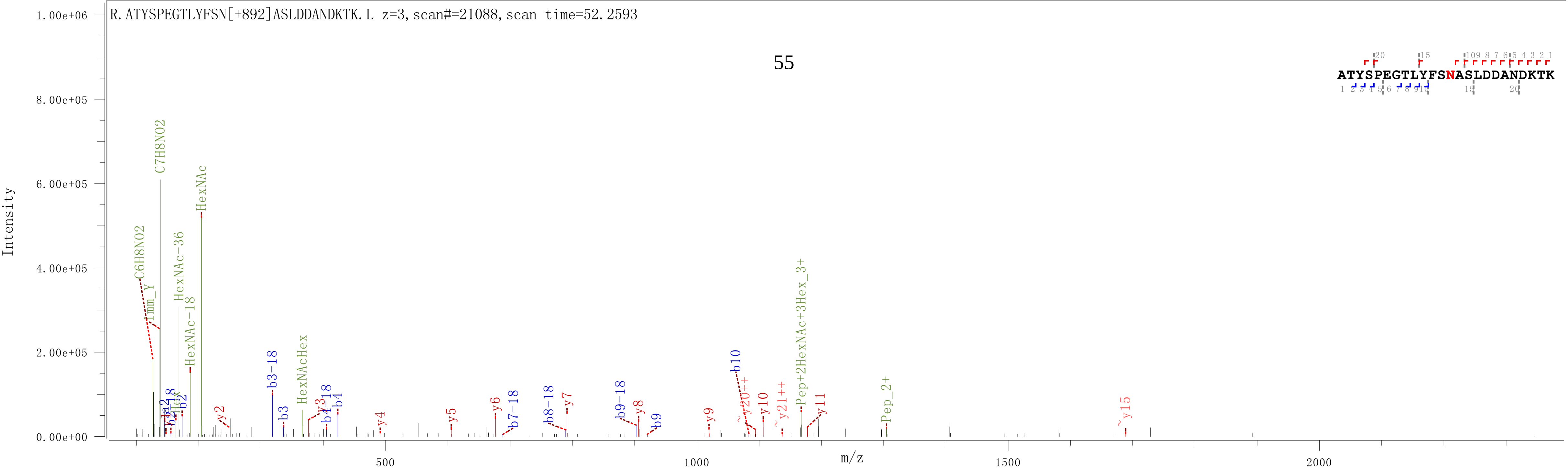

R.ATYSPEGTLYFSN[+892]ASLDDANDKTK.L z=3,scan#=21088,scan time=52.2593
55
ATYSPEGTLYFSNASLDDANDKTK
Intensity
m/z
1.00e+06
8.00e+05
6.00e+05
4.00e+05
2.00e+05
0.00e+00
500
1000
1500
2000
C6H8NO2
C7H8NO2
HexNAc-36
HexNAc-18
HexNAc
b3-18
b3
HexNAcHex
b4-18
b4
y2
y3
y4
y5
y6
b7-18
b8-18
y7
b9-18
y8
b9
y9
b10
y10
y11
Pep+2HexNAc+3Hex_3+
Pep_2+

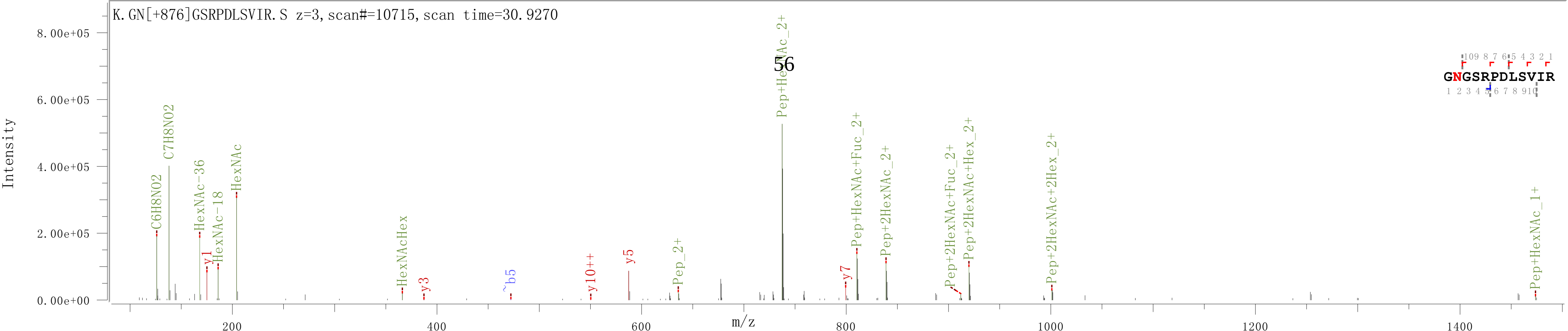

K.GN[+876]GSRPDLSVIR.S z=3,scan#=10715,scan time=30.9270
Intensity
m/z
C6H8NO2
C7H8NO2
HexNAc-36
y1
HexNAc-18
HexNAc
HexNAcHex
y3
~b5
y10++
y5
Pep_2+
Pep+HexNAc_2+
y7
Pep+HexNAc+Fuc_2+
Pep+2HexNAc_2+
Pep+2HexNAc+Fuc_2+
Pep+2HexNAc+Hex_2+
Pep+2HexNAc+2Hex_2+
Pep+HexNAc_1+
GNGSRPDLSVIR

56

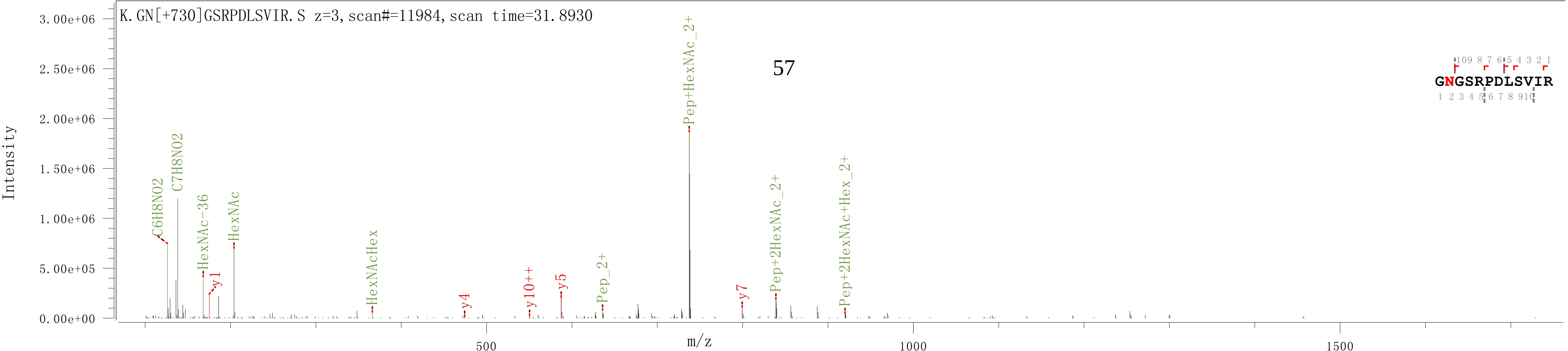

K.GN[+730]GSRPDLSVIR.S z=3,scan#=11984,scan time=31.8930
57
GNGSRPDLSVIR
Intensity
m/z
C6H8NO2
C7H8NO2
HexNAc-36
y1
HexNAc
HexNAcHex
y4
y10++
y5
Pep_2+
Pep+HexNAc_2+
y7
Pep+2HexNAc_2+
Pep+2HexNAc+Hex_2+

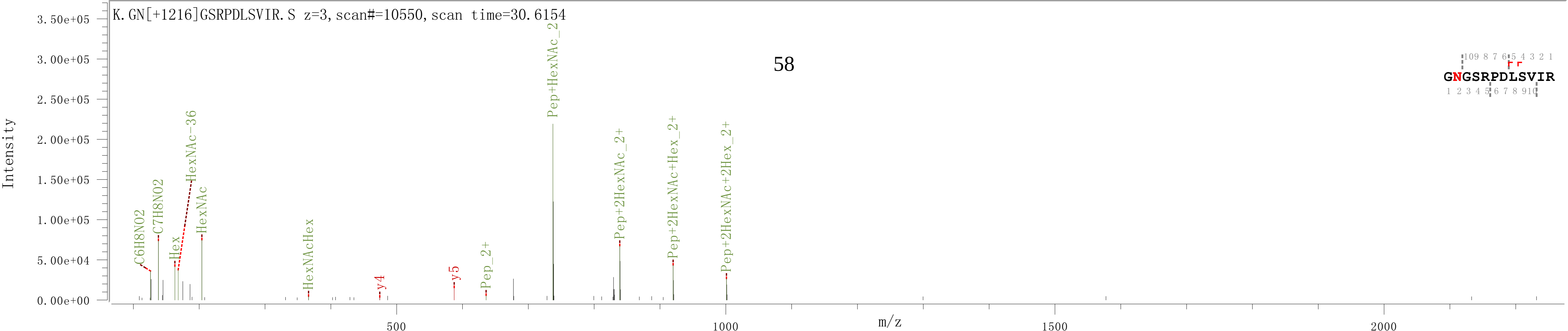

K.GN[+1216]GSRPDLSVIR.S z=3,scan#=10550,scan time=30.6154
58
GNGSRPDLSVIR
Intensity
m/z
C6H8NO2
C7H8NO2
Hex
HexNAc-36
HexNAc
HexNAcHex
y4
y5
Pep_2+
Pep+HexNAc_2
Pep+2HexNAc_2+
Pep+2HexNAc+Hex_2+
Pep+2HexNAc+2Hex_2+

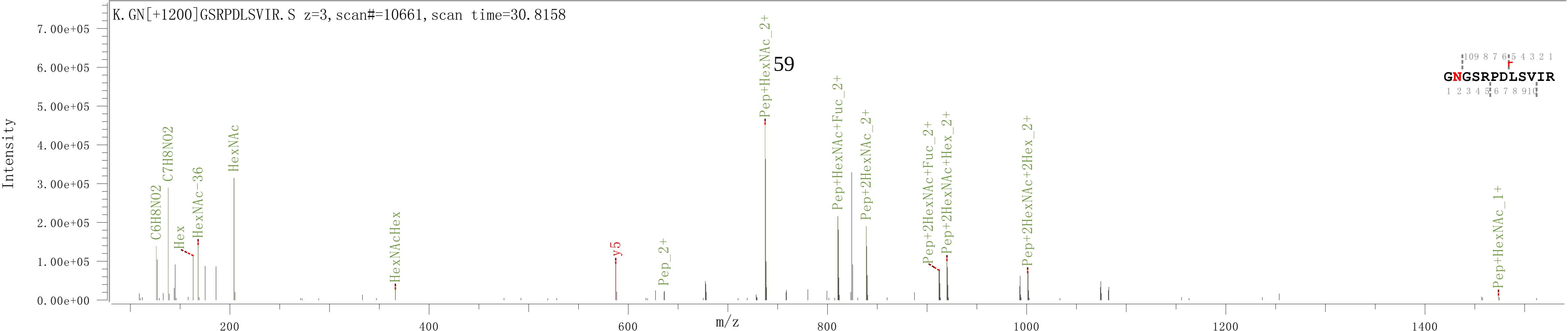

K.GN[+1200]GSRPDLSVIR.S z=3,scan#=10661,scan time=30.8158
Intensity
m/z
C6H8NO2
C7H8NO2
Hex
HexNAc-36
HexNAc
HexNAcHex
y5
Pep_2+
Pep+HexNAc_2+
Pep+HexNAc+Fuc_2+
Pep+2HexNAc_2+
Pep+2HexNAc+Fuc_2+
Pep+2HexNAc+Hex_2+
Pep+2HexNAc+2Hex_2+
Pep+HexNAc_1+
GNGSRPDLSVIR

59

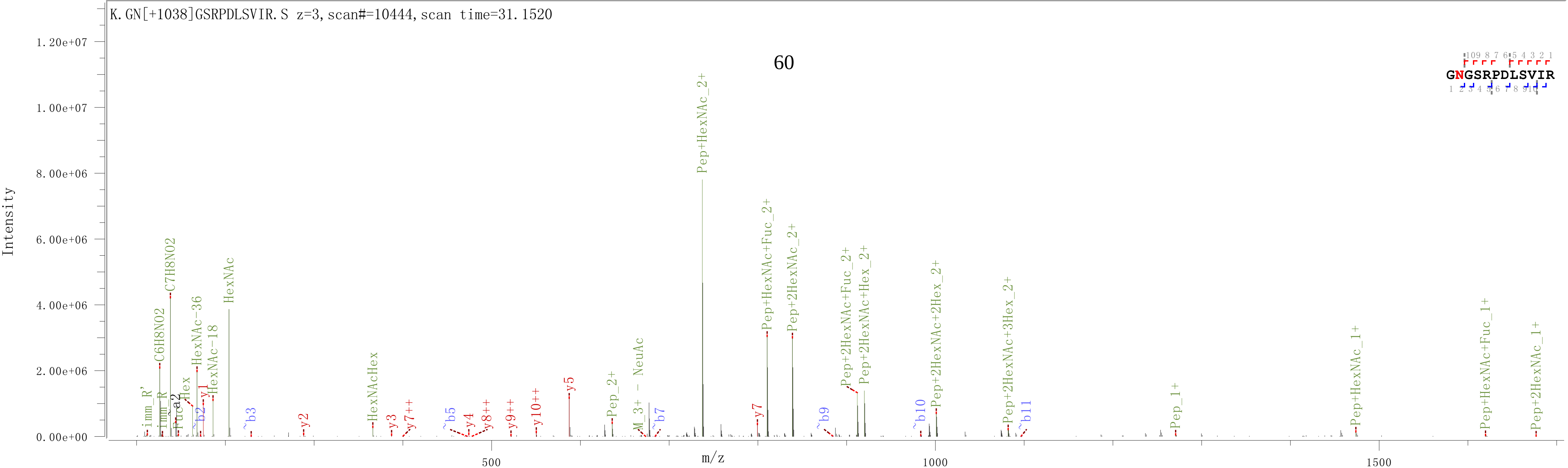

K.GN[+1038]GSRPDLSVIR.S z=3,scan#=10444,scan time=31.1520
60
GNGSRPDLSVIR
Intensity
m/z
Pep+HexNAc_2+
Pep+HexNAc+Fuc_2+
Pep+2HexNAc_2+
Pep+2HexNAc+Fuc_2+
Pep+2HexNAc+Hex_2+
Pep+2HexNAc+2Hex_2+
Pep+2HexNAc+3Hex_2+
Pep+HexNAc_1+
Pep+HexNAc+Fuc_1+
Pep+2HexNAc_1+
Pep_1+
Pep_2+
M_3+ - NeuAc
HexNAc
HexNAc-18
HexNAc-36
HexNAcHex
C7H8NO2
C6H8NO2
imm_R'
imm_R
Fuc
Hex
a2

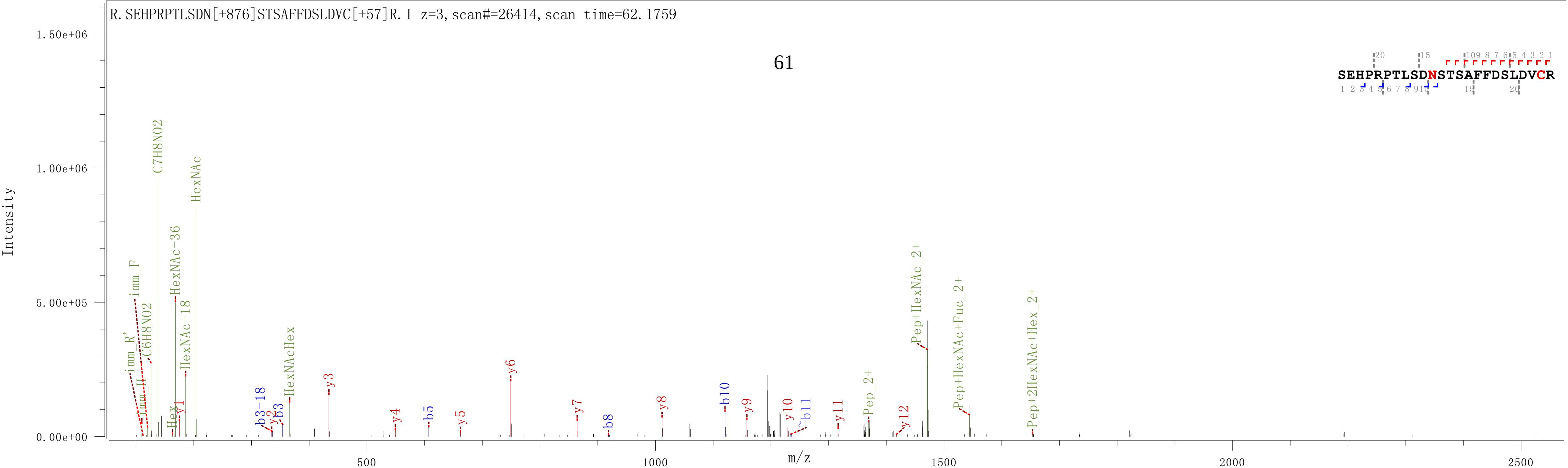
R.SEHPRPTLSDN[+876]STSAFFDSLDVC[+57]R.I z=3,scan#=26414,scan time=62.1759
61
SEHPRPTLSDNSTSAFFDSLDVCR
Intensity
m/z
1.50e+06
1.00e+06
5.00e+05
0.00e+00
500
1000
1500
2000
2500
imm_R'
imm_F
imm_H
C6H8NO2
C7H8NO2
Hex
HexNAc-36
HexNAc-18
y1
HexNAc
b3-18
y2
b3
HexNAcHex
y3
y4
b5
y5
y6
y7
b8
y8
b10
y9
y10
~b11
y11
Pep_2+
y12
Pep+HexNAc_2+
Pep+HexNAc+Fuc_2+
Pep+2HexNAc+Hex_2+

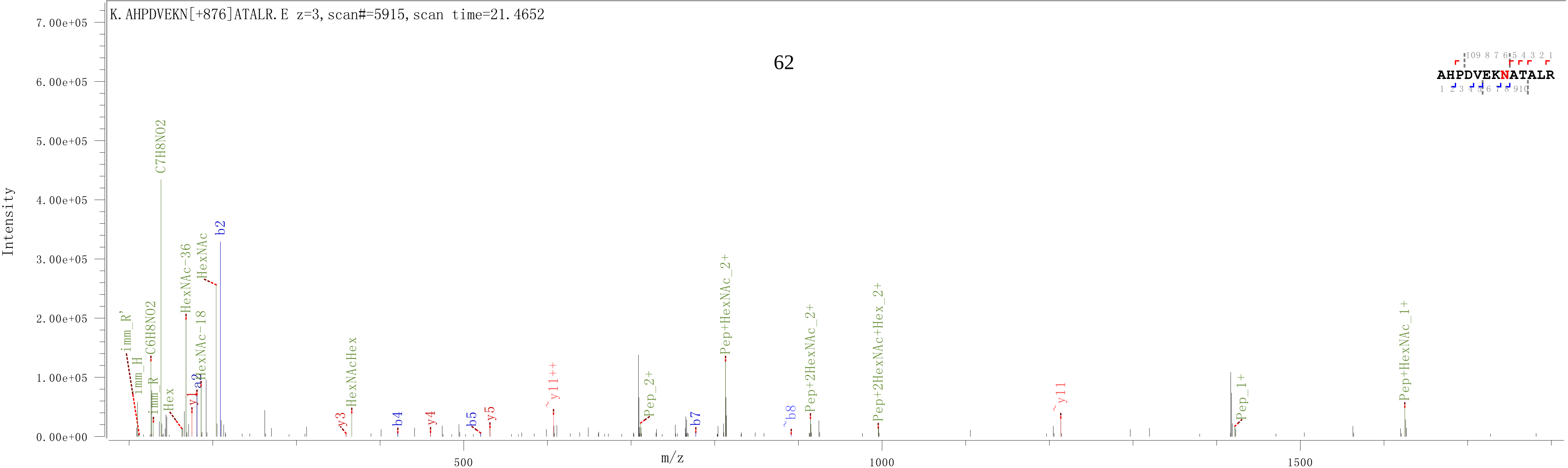
K.AHPDVEKN[+876]ATALR.E z=3,scan#=5915,scan time=21.4652
AHPDVEKNATALR
Intensity
m/z
0.00e+00
1.00e+05
2.00e+05
3.00e+05
4.00e+05
5.00e+05
6.00e+05
7.00e+05
500
1000
1500
imm_R'
imm_H
imm_R
C6H8NO2
C7H8NO2
Hex
HexNAc-36
y1
a2
HexNAc-18
HexNAc
b2
y3
HexNAcHex
b4
y4
b5
y5
~y11++
Pep_2+
b7
Pep+HexNAc_2+
~b8
Pep+2HexNAc_2+
Pep+2HexNAc+Hex_2+
~y11
Pep_1+
Pep+HexNAc_1+

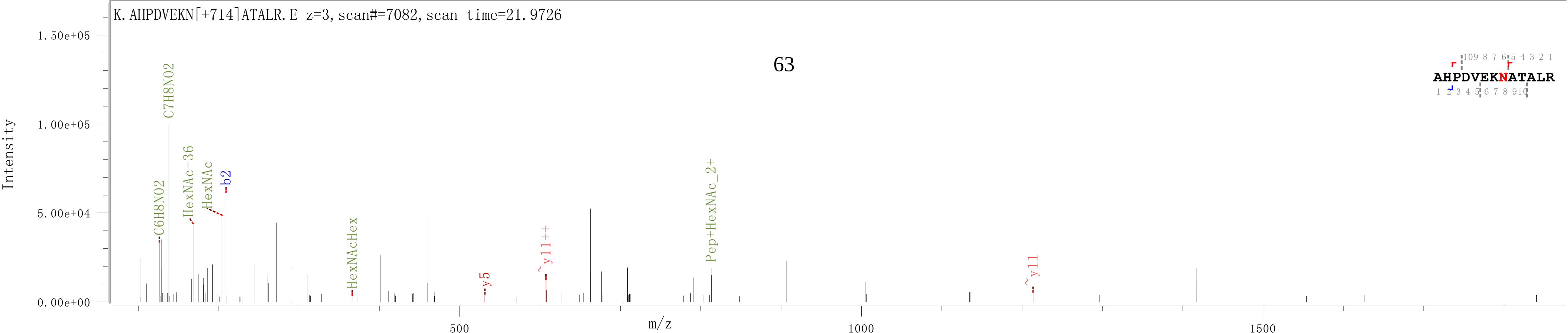
K.AHPDVEKN[+714]ATALR.E z=3,scan#=7082,scan time=21.9726
Intensity
m/z
1.50e+05
1.00e+05
5.00e+04
0.00e+00
500
1000
1500
C6H8NO2
C7H8NO2
HexNAc-36
HexNAc
b2
HexNAcHex
y5
~y11++
Pep+HexNAc_2+
~y11
AHPDVEKNATALR

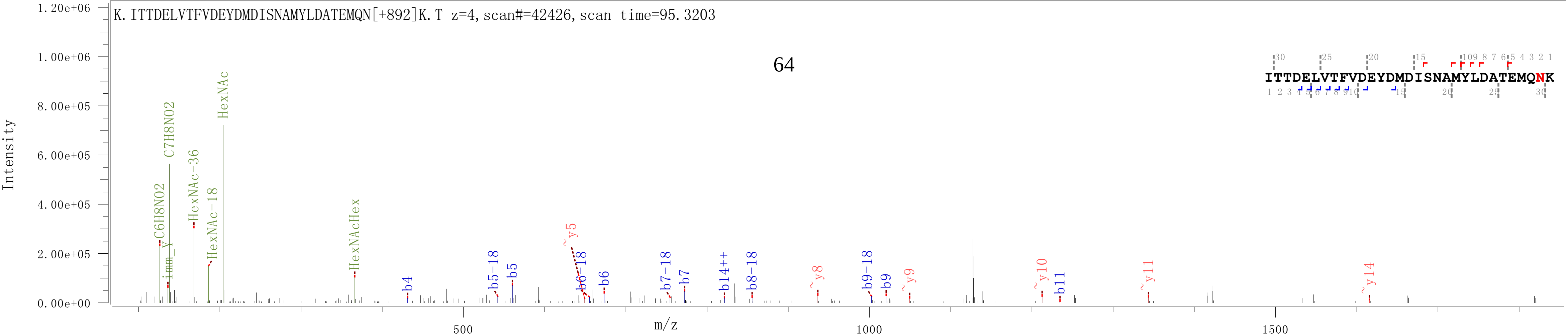
K.ITTDELVTFVDEYDMDISNAMYLDATEMQN[+892]K.T z=4,scan#=42426,scan time=95.3203
ITTDELVTFVDEYDMDISNAMYLDATEMQNK
Intensity
m/z
1.20e+06
1.00e+06
8.00e+05
6.00e+05
4.00e+05
2.00e+05
0.00e+00
500
1000
1500
HexNAc
C7H8NO2
HexNAc-36
HexNAc-18
C6H8NO2
imm_Y
HexNAcHex
b4
b5-18
b5
~y5
b6-18
b6
b7-18
b7
b14++
b8-18
~y8
b9-18
b9
~y9
~y10
b11
~y11
~y14

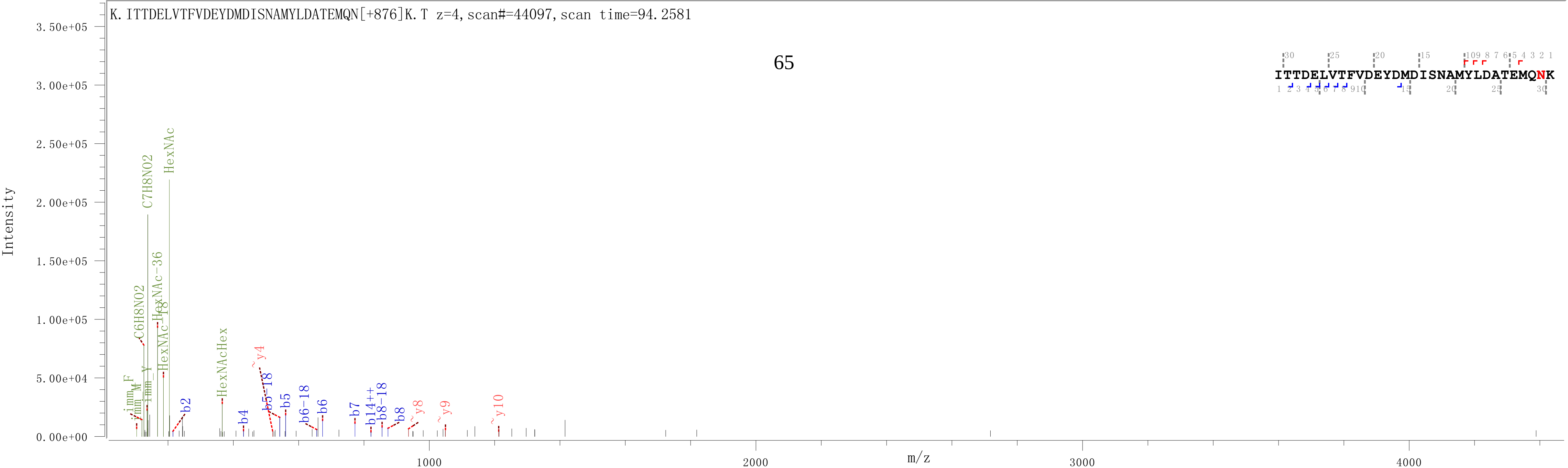
K.ITTDELVTFVDEYDMDISNAMYLDATEMQN[+876]K.T z=4,scan#=44097,scan time=94.2581
65
ITTDELVTFVDEYDMDISNAMYLDATEMQNK
Intensity
m/z
HexNAc
C7H8NO2
HexNAc-36
C6H8NO2
HexNAc-18
HexNAcHex
imm_F
imm_M
imm_Y
b2
b4
~y4
b5-18
b5
b6-18
b6
b7
b14++
b8-18
b8
~y8
~y9
~y10

66

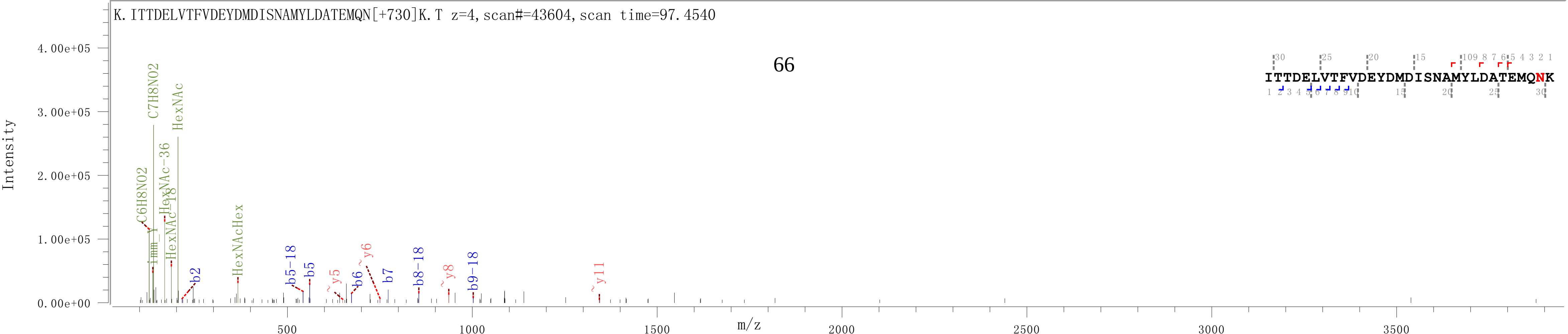
K.ITTDELVTFVDEYDMDISNAMYLDATEMQN[+730]K.T z=4,scan#=43604,scan time=97.4540
ITTDELVTFVDEYDMDISNAMYLDATEMQNK
Intensity
m/z
C6H8NO2
C7H8NO2
HexNAc
HexNAc-36
HexNAc-18
HexNAcHex
b2
b5-18
b5
~y5
b6
~y6
b7
b8-18
~y8
b9-18
~y11

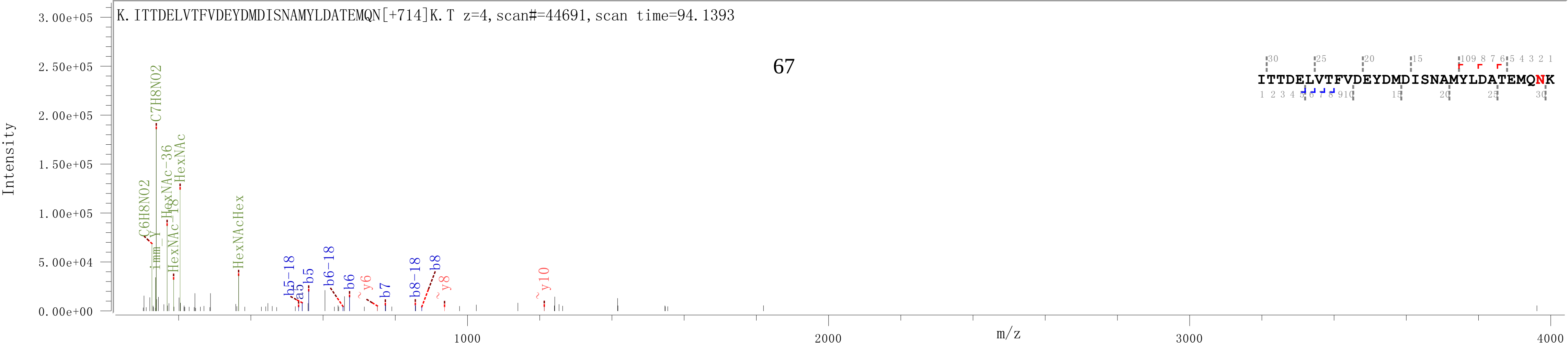
K.ITTDELVTFVDEYDMDISNAMYLDATEMQN[+714]K.T z=4,scan#=44691,scan time=94.1393
ITTDELVTFVDEYDMDISNAMYLDATEMQNK
Intensity
m/z
C7H8NO2
C6H8NO2
HexNAc
HexNAc-36
HexNAc-18
HexNAcHex
b5-18
a5
b5
b6-18
b6
~y6
b7
b8-18
b8
~y8
~y10

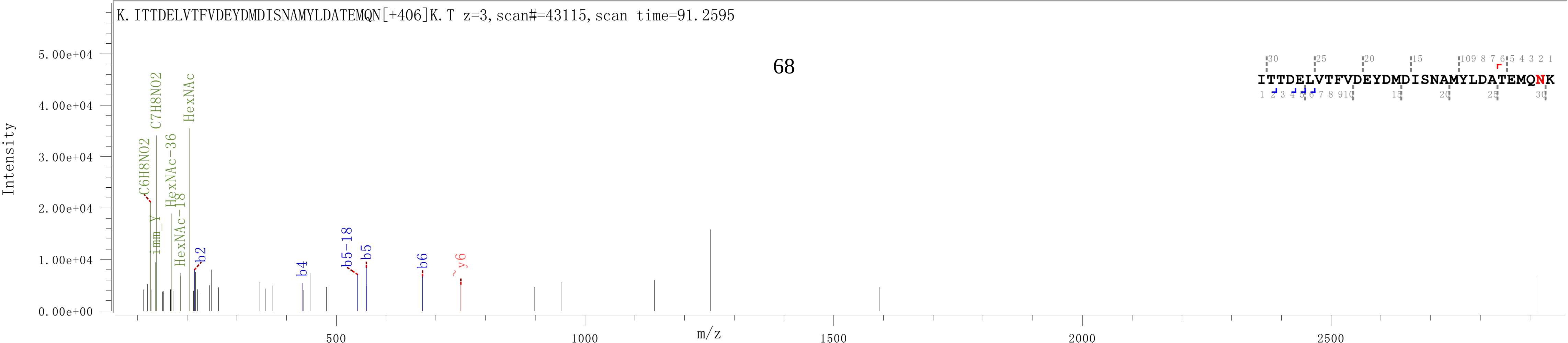
K.ITTDELVTFVDEYDMDISNAMYLDATEMQN[+406]K.T z=3,scan#=43115,scan time=91.2595
ITTDELVTFVDEYDMDISNAMYLDATEMQNK
Intensity
m/z
5.00e+04
4.00e+04
3.00e+04
2.00e+04
1.00e+04
0.00e+00
500
1000
1500
2000
2500
C6H8NO2
C7H8NO2
HexNAc
HexNAc-36
HexNAc-18
imm_Y
b2
b4
b5-18
b5
b6
~y6

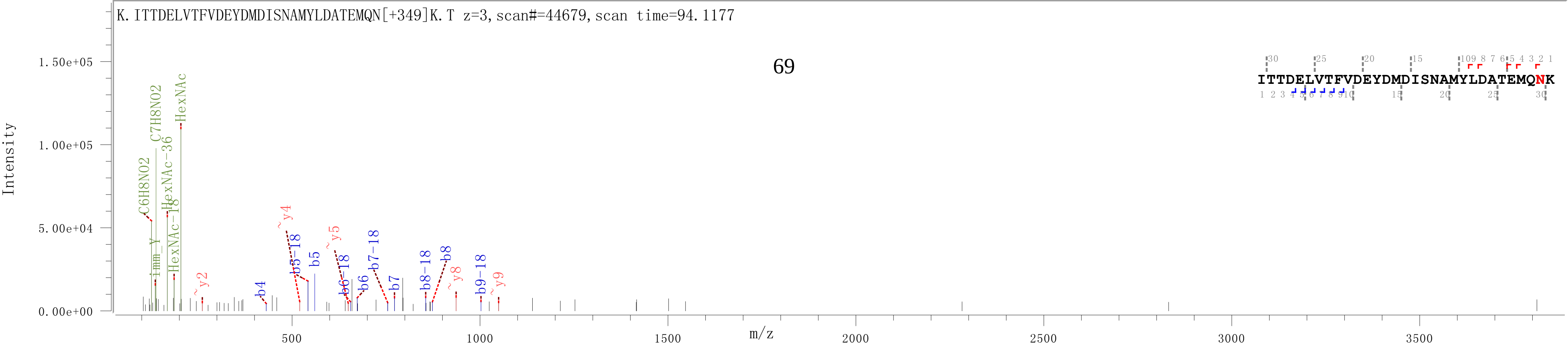
K.ITTDELVTFVDEYDMDISNAMYLDATEMQN[+349]K.T z=3,scan#=44679,scan time=94.1177
ITTDELVTFVDEYDMDISNAMYLDATEMQNK
Intensity
m/z
C6H8NO2
C7H8NO2
HexNAc
HexNAc-36
HexNAc-18
imm_Y
~y2
b4
~y4
b5-18
b5
~y5
b6-18
b6
b7-18
b7
b8-18
b8
~y8
b9-18
~y9

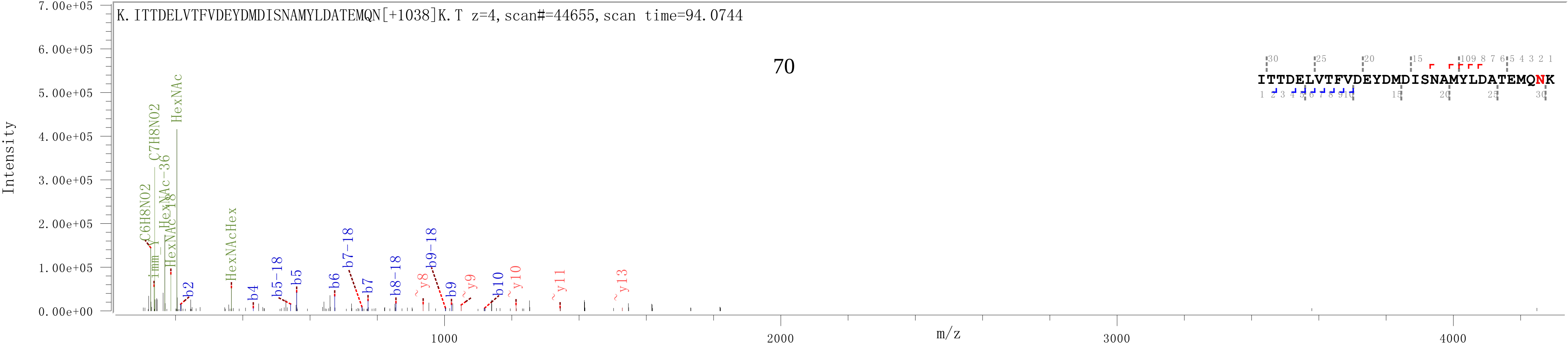

K.ITTDELVTFVDEYDMDISNAMYLDATEMQN[+1038]K.T z=4,scan#=44655,scan time=94.0744
70
ITTDELVTFVDEYDMDISNAMYLDATEMQNK
Intensity
m/z
C6H8NO2
C7H8NO2
HexNAc
HexNAc-36
HexNAc-18
HexNAcHex
b2
b4
b5-18
b5
b6
b7-18
b7
b8-18
b9-18
b9
b10
~y8
~y9
~y10
~y11
~y13

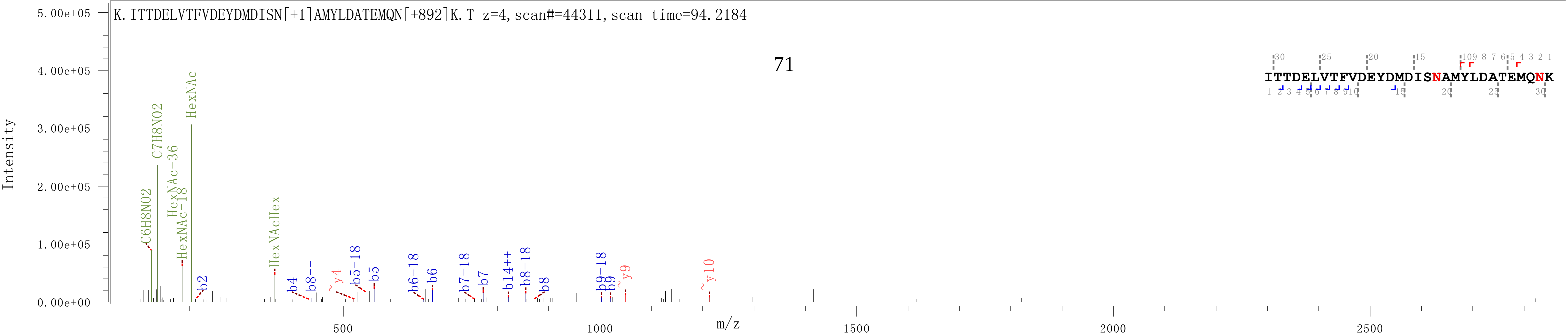

K.ITTDELVTFVDEYDMDISN[+1]AMYLDATEMQN[+892]K.T z=4,scan#=44311,scan time=94.2184
71
ITTDELVTFVDEYDMDISNAMYLDATEMQNK
Intensity
m/z

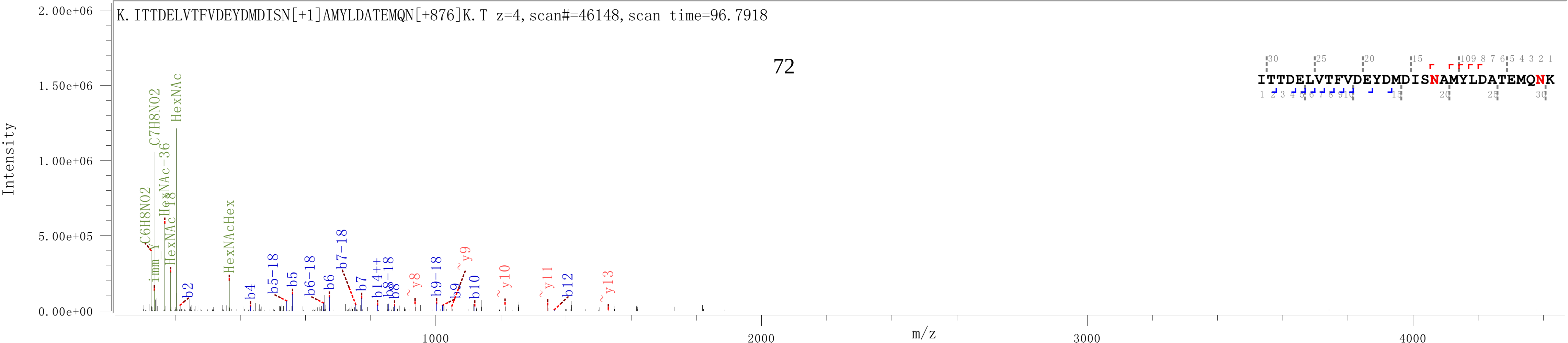
K.ITTDELVTFVDEYDMDISN[+1]AMYLDATEMQN[+876]K.T z=4,scan#=46148,scan time=96.7918
72
ITTDELVTFVDEYDMDISNAMYLDATEMQNK
Intensity
m/z
2.00e+06
1.50e+06
1.00e+06
5.00e+05
0.00e+00
1000
2000
3000
4000
HexNAc
C7H8NO2
HexNAc-36
C6H8NO2
HexNAc-18
HexNAcHex
b2
b4
b5-18
b5
b6-18
b6
b7-18
b7
b14++
b8-18
b8
~y8
b9-18
b9
~y9
b10
~y10
~y11
b12
~y13

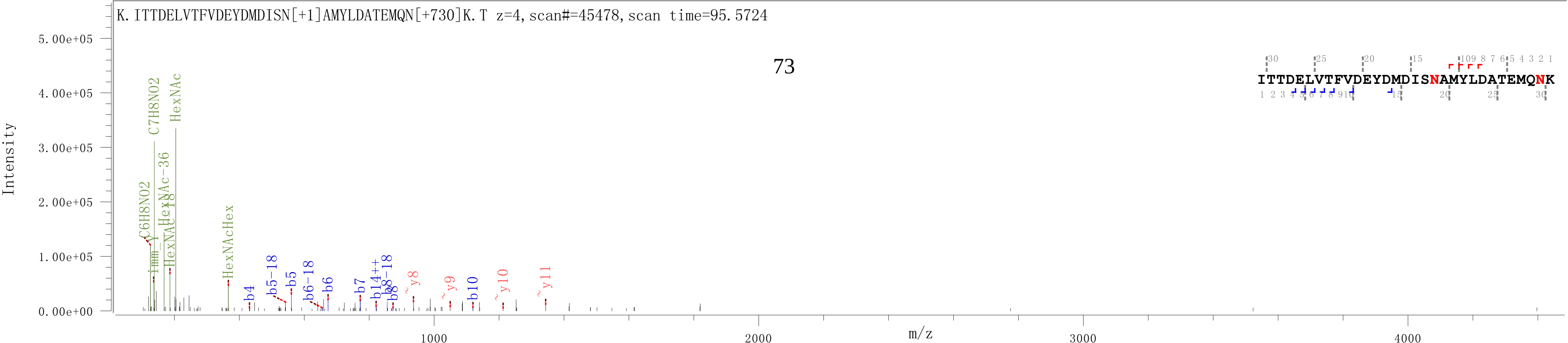
K.ITTDELVTFVDEYDMDISN[+1]AMYLDATEMQN[+730]K.T z=4,scan#=45478,scan time=95.5724
Intensity
m/z
5.00e+05
4.00e+05
3.00e+05
2.00e+05
1.00e+05
0.00e+00
1000
2000
3000
4000
C6H8NO2
C7H8NO2
HexNAc
HexNAc-36
HexNAc-18
HexNAcHex
b4
b5-18
b5
b6-18
b6
b7
b14++
b8-18
b8
y8
y9
b10
y10
y11
ITTDELVTFVDEYDMDISNAMYLDATEMQNK

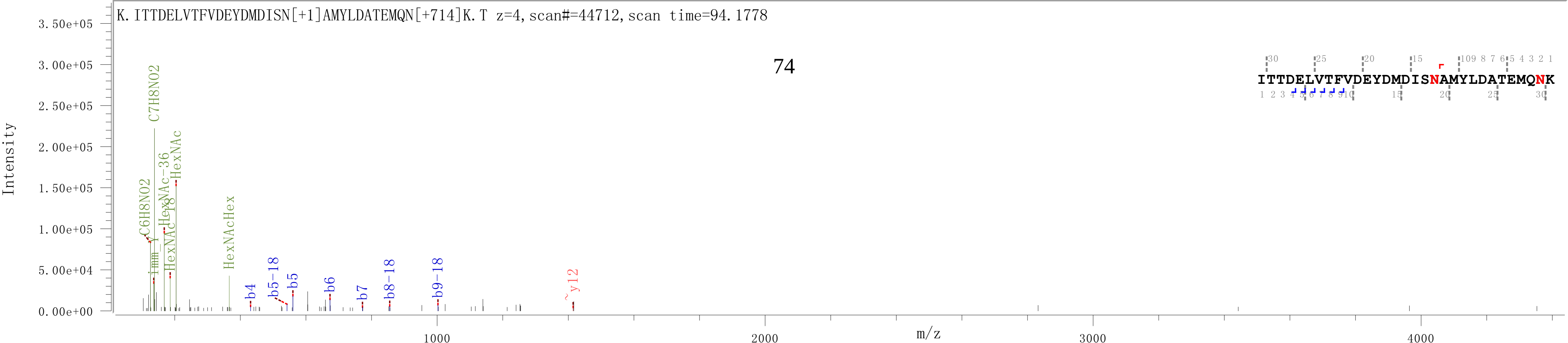
K.ITTDELVTFVDEYDMDISN[+1]AMYLDATEMQN[+714]K.T z=4,scan#=44712,scan time=94.1778
74
ITTDELVTFVDEYDMDISNAMYLDATEMQNK
Intensity
m/z
C7H8NO2
C6H8NO2
HexNAc
HexNAc-36
HexNAc-18
HexNAcHex
b4
b5-18
b5
b6
b7
b8-18
b9-18
~y12
3.50e+05
3.00e+05
2.50e+05
2.00e+05
1.50e+05
1.00e+05
5.00e+04
0.00e+00
1000
2000
3000
4000

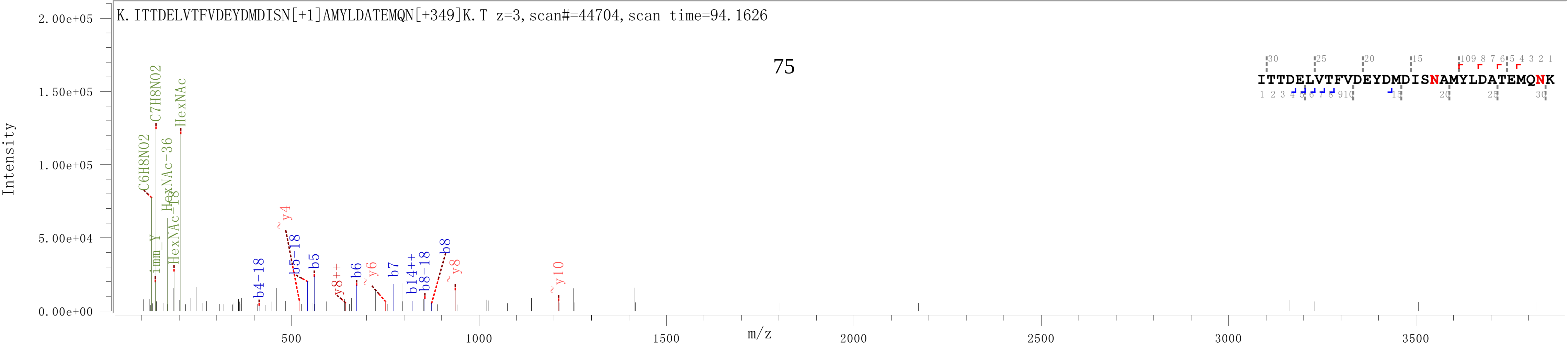

K.ITTDELVTFVDEYDMDISN[+1]AMYLDATEMQN[+349]K.T z=3,scan#=44704,scan time=94.1626
ITTDELVTFVDEYDMDISNAMYLDATEMQNK
Intensity
m/z

75

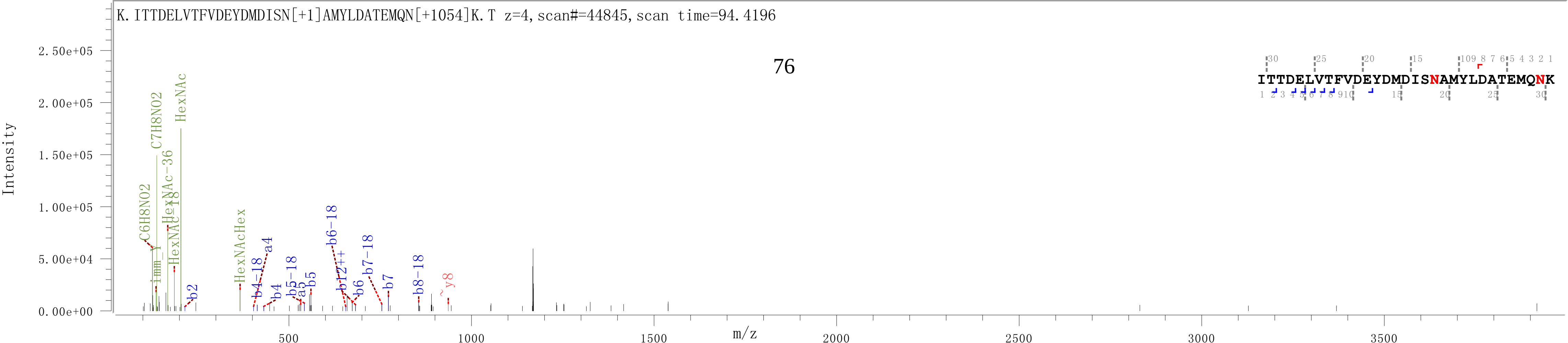
K.ITTDELVTFVDEYDMDISN[+1]AMYLDATEMQN[+1054]K.T z=4,scan#=44845,scan time=94.4196
Intensity
m/z
ITTDELVTFVDEYDMDISNAMYLDATEMQNK
HexNAc
C7H8NO2
HexNAc-36
HexNAc-18
C6H8NO2
HexNAcHex
b2
b4-18
a4
b4
b5-18
a5
b5
b6-18
b12++
b6
b7-18
b7
b8-18
y8
0.00e+00
5.00e+04
1.00e+05
1.50e+05
2.00e+05
2.50e+05
500
1000
1500
2000
2500
3000
3500

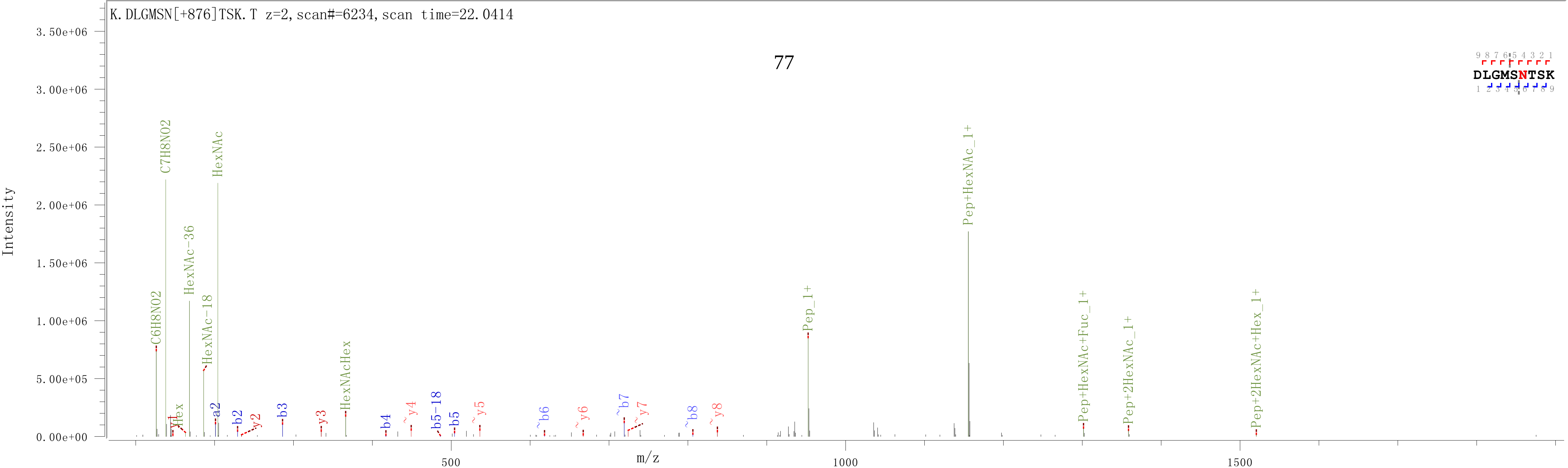
K.DLGMSN[+876]TSK.T z=2,scan#=6234,scan time=22.0414
DLGMSNTSK
Intensity
m/z
3.50e+06
3.00e+06
2.50e+06
2.00e+06
1.50e+06
1.00e+06
5.00e+05
0.00e+00
500
1000
1500
C6H8NO2
C7H8NO2
Hex
HexNAc-36
HexNAc-18
HexNAc
a2
b2
y2
b3
y3
HexNAcHex
b4
~y4
b5-18
b5
~y5
~b6
~y6
~b7
~y7
~b8
~y8
Pep_1+
Pep+HexNAc_1+
Pep+HexNAc+Fuc_1+
Pep+2HexNAc_1+
Pep+2HexNAc+Hex_1+

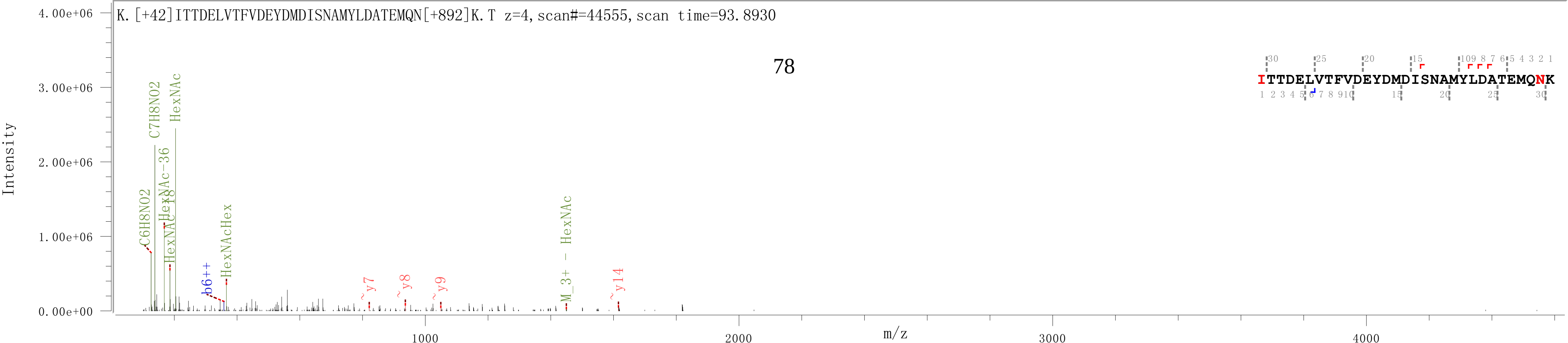
K.[+42]ITTDELVTFVDEYDMDISNAMYLDATEMQN[+892]K.T z=4,scan#=44555,scan time=93.8930
ITTDELVTFVDEYDMDISNAMYLDATEMQNK
Intensity
m/z
4.00e+06
3.00e+06
2.00e+06
1.00e+06
0.00e+00
1000
2000
3000
4000
C6H8NO2
C7H8NO2
HexNAc-36
HexNAc-18
HexNAc
b6++
HexNAcHex
~y7
~y8
~y9
M_3+ - HexNAc
~y14

R.VC[+57]SADQSYLAIINTKEEADHLVN[+876]MTR.L z=4,scan#=31641,scan time=71.5860

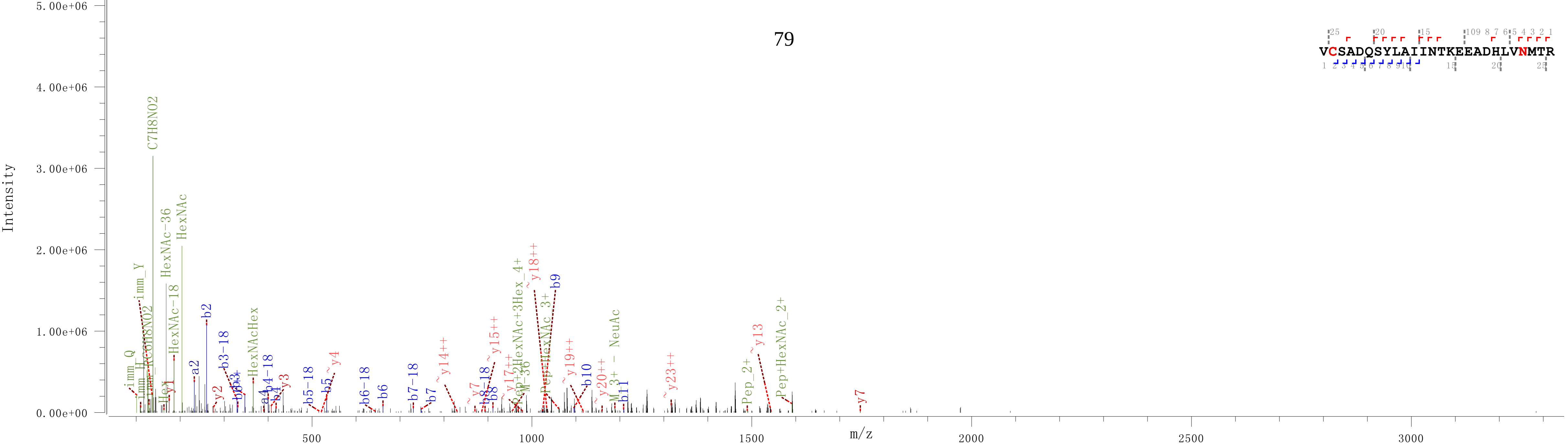

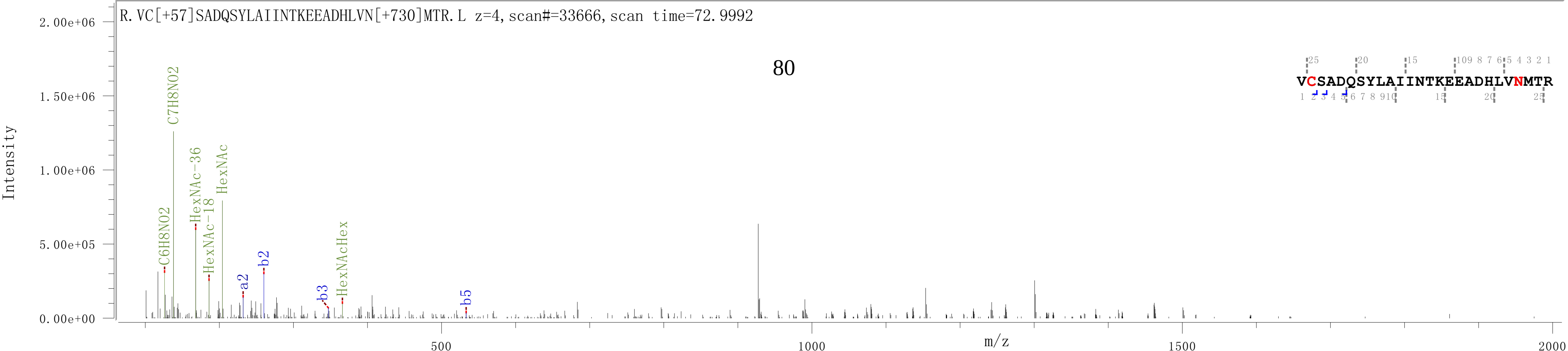

R.VC[+57]SADQSYLAIINTKEEADHLVN[+730]MTR.L z=4,scan#=33666,scan time=72.9992
80
VCSADQSYLAIINTKEEADHLVNMTR
Intensity
m/z
2.00e+06
1.50e+06
1.00e+06
5.00e+05
0.00e+00
500
1000
1500
2000
C6H8NO2
C7H8NO2
HexNAc-36
HexNAc-18
HexNAc
a2
b2
b3
HexNAcHex
b5

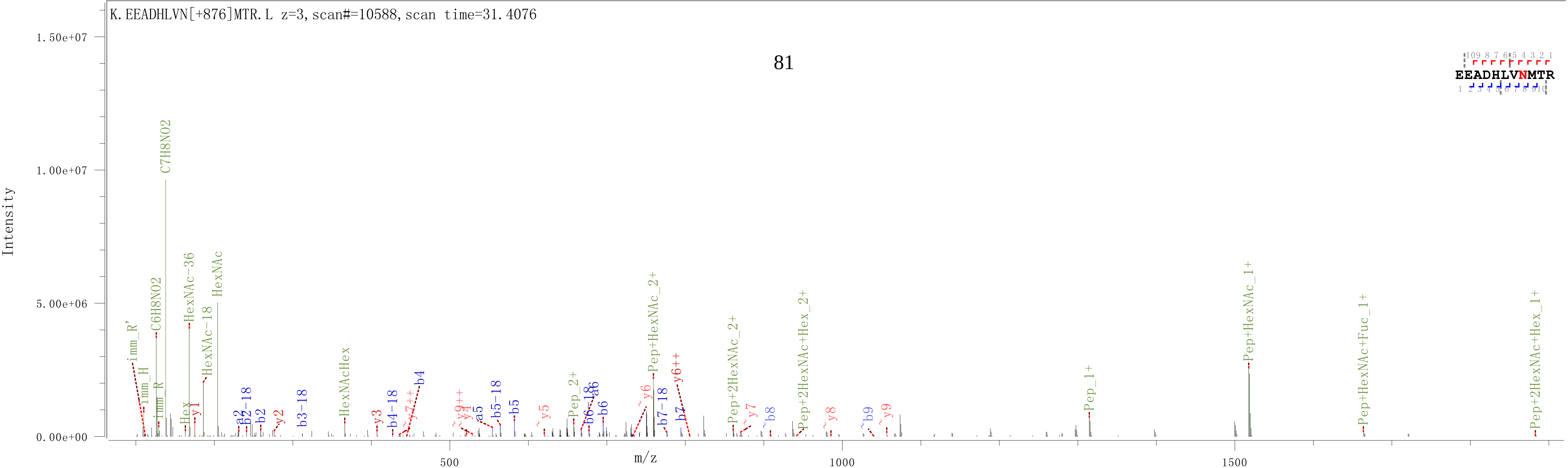

K.EEADHLVN[+876]MTR.L z=3,scan#=10588,scan time=31.4076
81
EEADHLVNMTR
Intensity
m/z
1.50e+07
1.00e+07
5.00e+06
0.00e+00
500
1000
1500
C7H8NO2
C6H8NO2
HexNAc-36
HexNAc-18
HexNAc
HexNAcHex
imm_R'
imm_H
imm_R
Hex
y1
y2
b2
b3-18
b4-18
b4
y3
b5-18
b5
~y5
Pep_2+
b6-18
a6
b6
~y6
Pep+HexNAc_2+
b7-18
b7
y6++
Pep+2HexNAc_2+
~y7
~b8
Pep+2HexNAc+Hex_2+
~y8
~b9
~y9
Pep_1+
Pep+HexNAc_1+
Pep+HexNAc+Fuc_1+
Pep+2HexNAc+Hex_1+

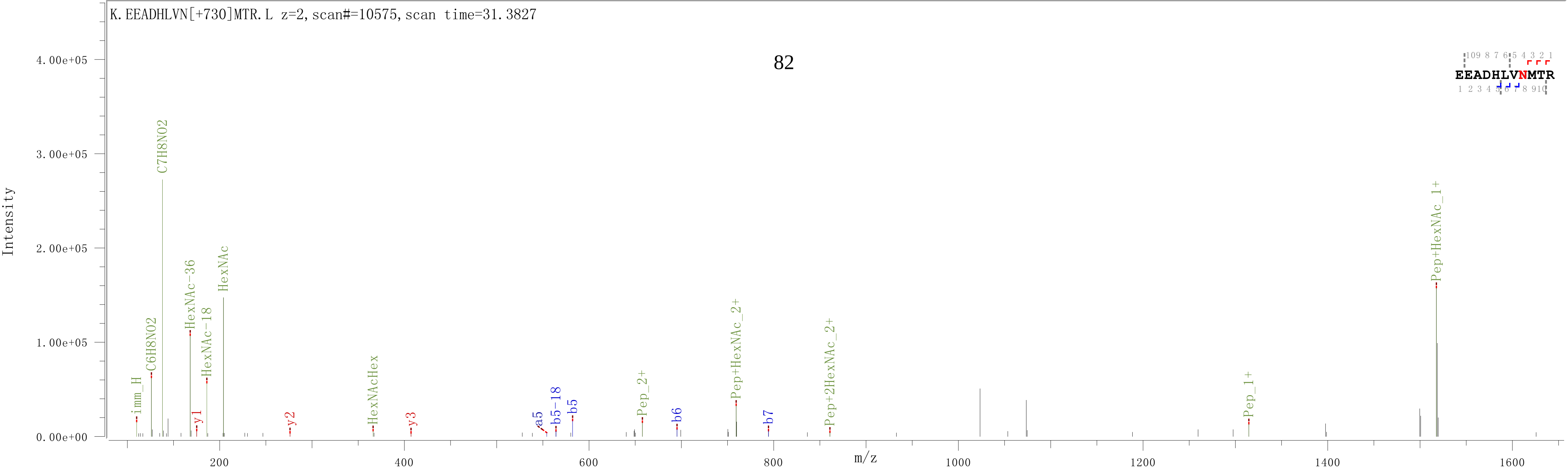
K.EEADHLVN[+730]MTR.L z=2,scan#=10575,scan time=31.3827
82
EEADHLVNMTR
Intensity
m/z
imm_H
C6H8NO2
C7H8NO2
HexNAc-36
HexNAc-18
HexNAc
y1
y2
HexNAcHex
y3
a5
b5-18
b5
Pep_2+
b6
Pep+HexNAc_2+
b7
Pep+2HexNAc_2+
Pep_1+
Pep+HexNAc_1+

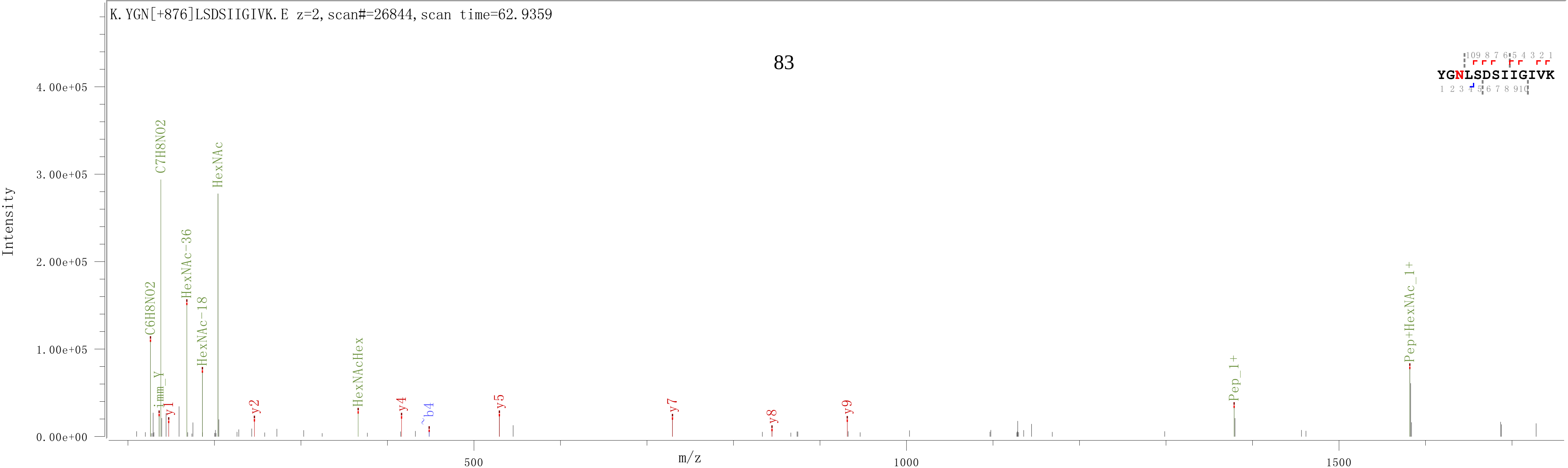

K.YGN[+876]LSDSIIGIVK.E z=2,scan#=26844,scan time=62.9359
YGNLSDSIIGIVK
Intensity
m/z
C6H8NO2
C7H8NO2
imm_Y
y1
HexNAc-36
HexNAc-18
HexNAc
y2
HexNAcHex
y4
~b4
y5
y7
y8
y9
Pep_1+
Pep+HexNAc_1+

84

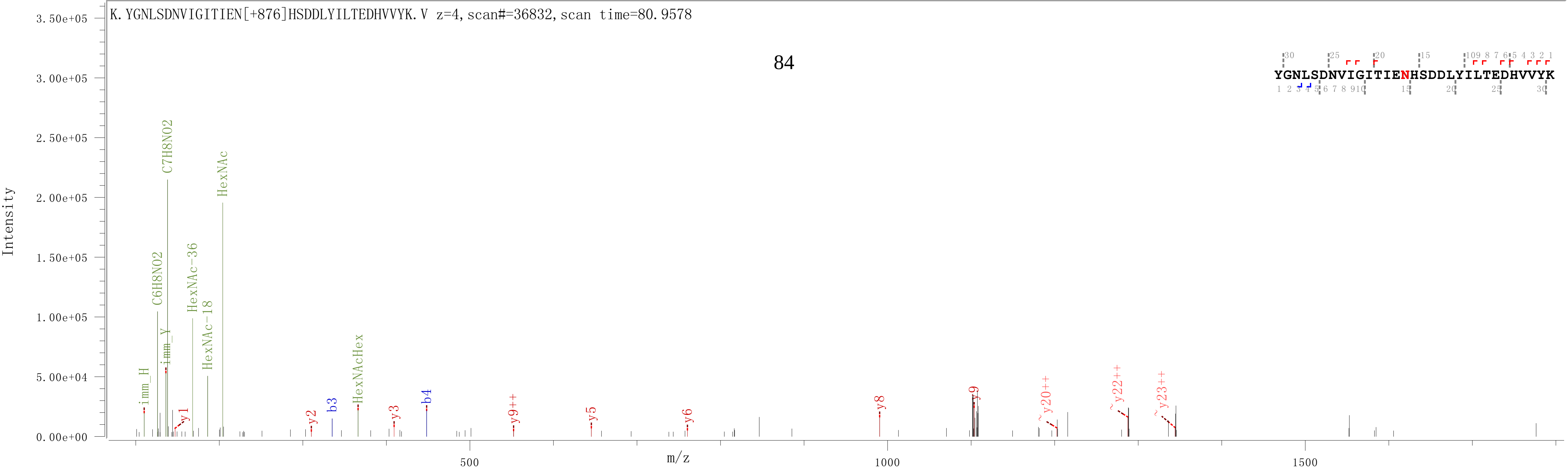

K.YGNLSDNVIGITIEN[+876]HSDDLYILTEDHVVYK.V z=4,scan#=36832,scan time=80.9578
YGNLSDNVIGITIENHSDDLYILTEDHVVYK
Intensity
m/z
C7H8NO2
HexNAc
C6H8NO2
HexNAc-36
HexNAc-18
imm_H
imm_Y
HexNAcHex
y1
y2
b3
y3
b4
y9++
y5
y6
y8
y9
~y20++
~y22++
~y23++

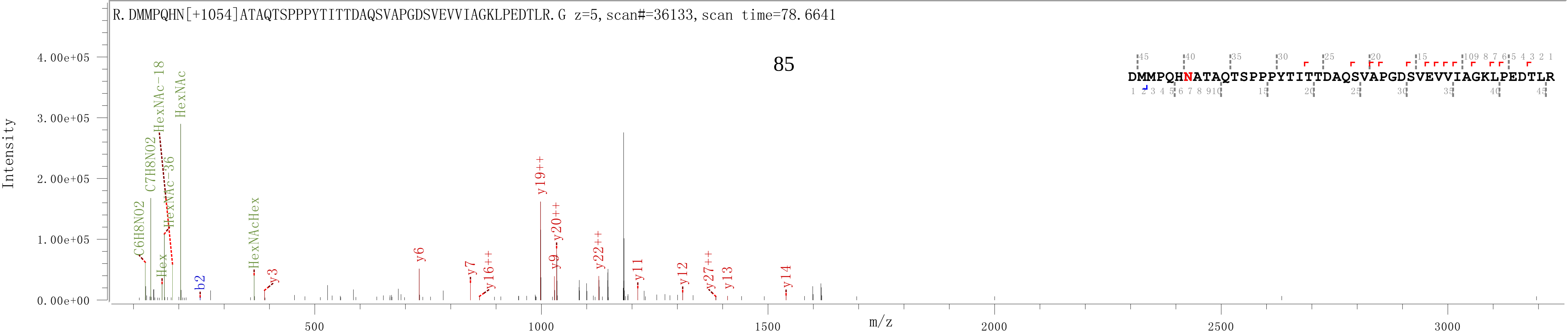
R.DMMPQHN[+1054]ATAQTSPPPYTITTDAQSVAPGDSVEVVIAGKLPEDTLR.G z=5,scan#=36133,scan time=78.6641
DMMPQHNATAQTSPPPYTITTDAQSVAPGDSVEVVIAGKLPEDTLR
Intensity
m/z
C6H8NO2
C7H8NO2
HexNAc-18
HexNAc-36
HexNAc
Hex
b2
HexNAcHex
y3
y6
y7
y16++
y19++
y9
y20++
y22++
y11
y12
y27++
y13
y14

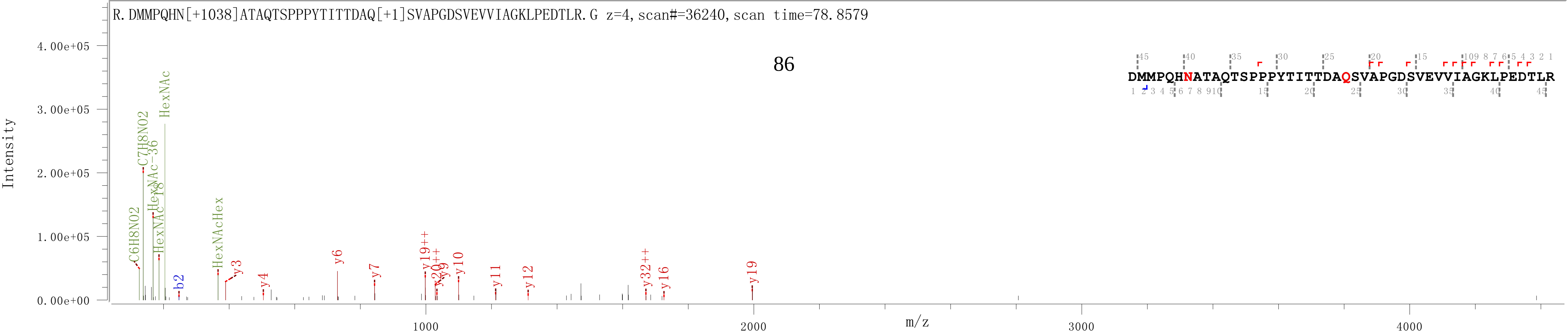
R.DMMPQHN[+1038]ATAQTSPPPYTITTDAQ[+1]SVAPGDSVEVVIAGKLPEDTLR.G z=4,scan#=36240,scan time=78.8579
86
DMMPQHNATAQTSPPPYTITTDAQSVAPGDSVEVVIAGKLPEDTLR
Intensity
m/z
C6H8NO2
C7H8NO2
HexNAc-36
HexNAc-18
HexNAc
HexNAcHex
b2
y3
y4
y6
y7
y19++
y20++
y9
y10
y11
y12
y32++
y16
y19

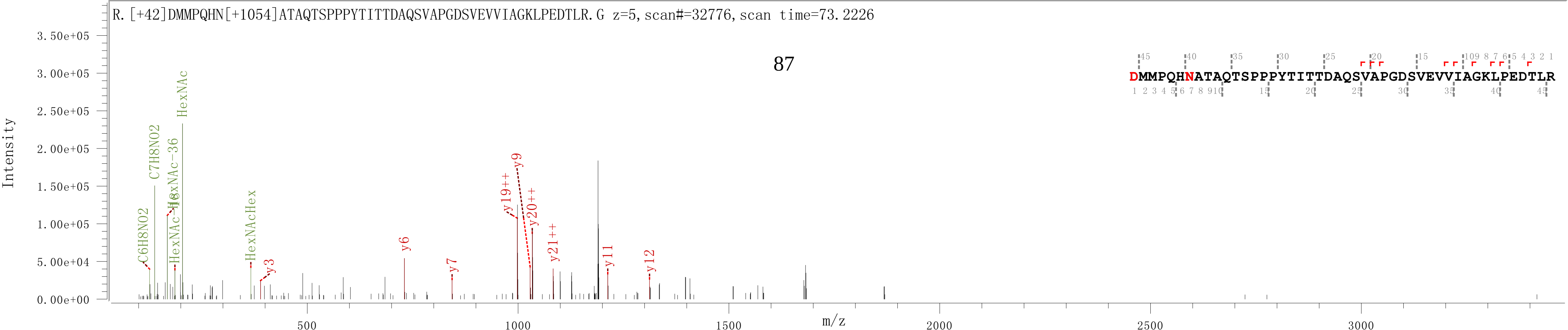
R.[+42]DMMPQHN[+1054]ATAQTSPPPYTITTDAQSVAPGDSVEVVIAGKLPEDTLR.G z=5,scan#=32776,scan time=73.2226
DMMPQHNATAQTSPPPYTITTDAQSVAPGDSVEVVIAGKLPEDTLR
Intensity
m/z
C6H8NO2
C7H8NO2
HexNAc-36
HexNAc
HexNAcHex
y3
y6
y7
y19++
y9
y20++
y21++
y11
y12

R. EKGVYEVDLNLN[+876]TTVK. I z=3, scan#=25500, scan time=60.5667

88

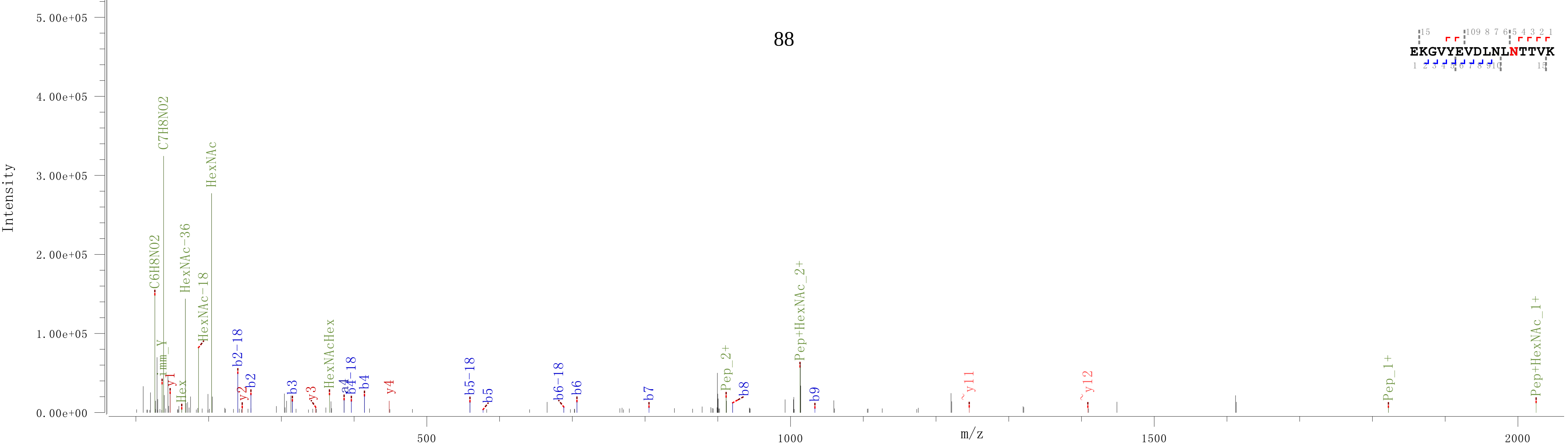

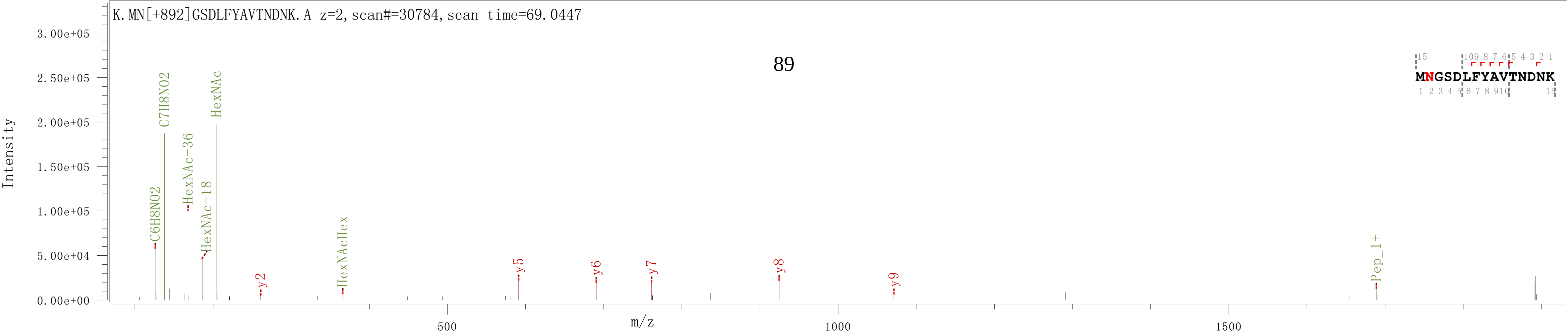
K.MN[+892]GSDLFYAVTNDNK.A z=2,scan#=30784,scan time=69.0447
89
MNGSDLFYAVTNDNK
Intensity
m/z
C6H8NO2
C7H8NO2
HexNAc-36
HexNAc-18
HexNAc
y2
HexNAcHex
y5
y6
y7
y8
y9
Pep_1+
500
1000
1500

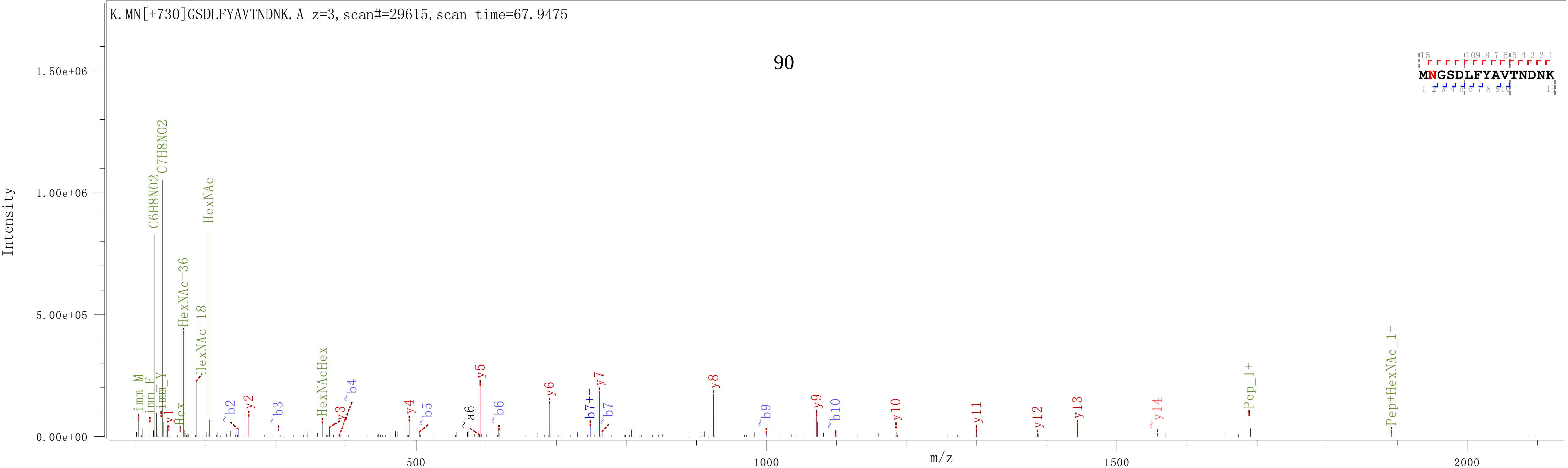
K.MN[+730]GSDLFYAVTNDNK.A z=3,scan#=29615,scan time=67.9475
MNGSDLFYAVTNDNK
Intensity
m/z
1.50e+06
1.00e+06
5.00e+05
0.00e+00
500
1000
1500
2000
C7H8NO2
C6H8NO2
HexNAc
HexNAc-36
HexNAc-18
HexNAcHex
imm_M
imm_F
imm_Y
Hex
y1
~b2
y2
~b3
~b4
y3
y4
~b5
~a6
y5
~b6
y6
b7++
~b7
y7
y8
~b9
y9
~b10
y10
y11
y12
y13
~y14
Pep_1+
Pep+HexNAc_1+

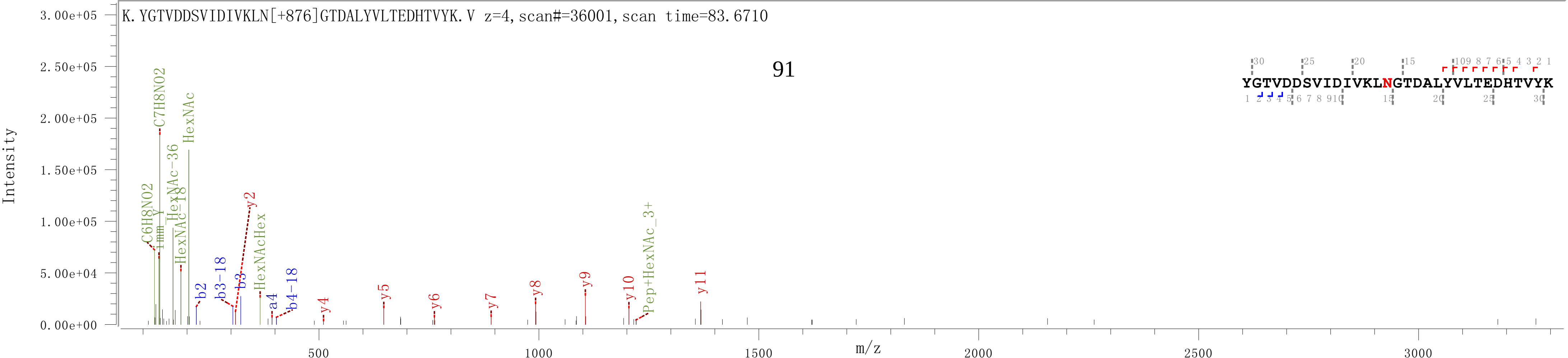
K.YGTVDDSVIDIVKLN[+876]GTDALYVLTEDHTVYK.V z=4,scan#=36001,scan time=83.6710
YGTVDDSVIDIVKLNGTDALYVLTEDHTVYK
Intensity
m/z
3.00e+05
2.50e+05
2.00e+05
1.50e+05
1.00e+05
5.00e+04
0.00e+00
500
1000
1500
2000
2500
3000
C6H8NO2
Imm_Y
C7H8NO2
HexNAc-36
HexNAc-18
HexNAc
b2
b3-18
b3
y2
HexNAcHex
a4
b4-18
y4
y5
y6
y7
y8
y9
y10
Pep+HexNAc_3+
y11

92

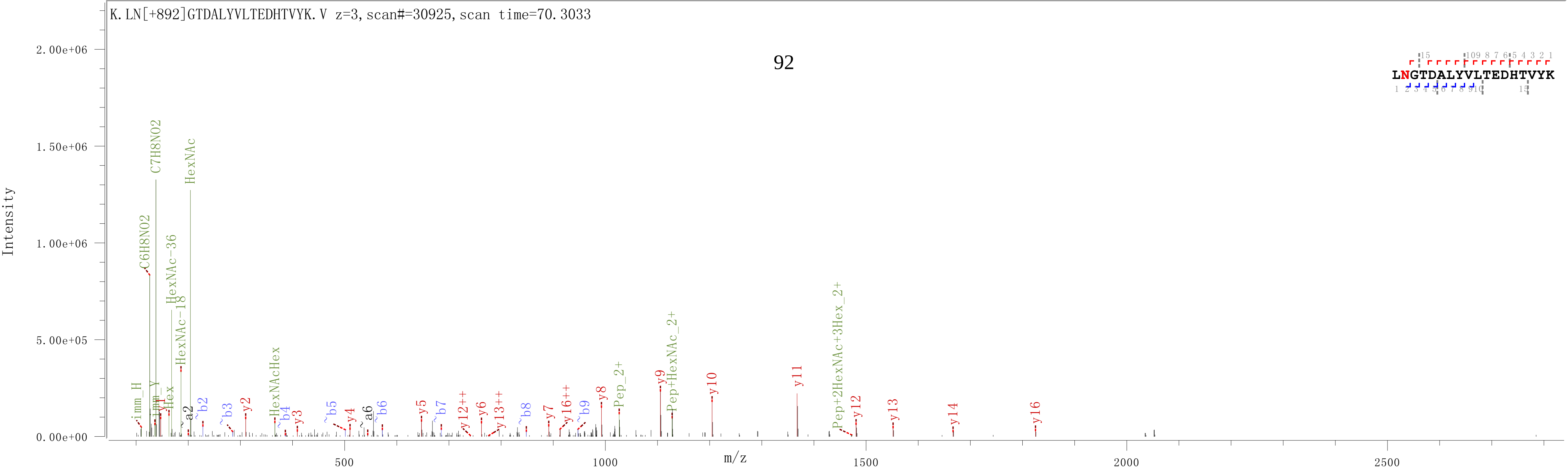

K.LN[+892]GTDALYVLTEDHTVYK.V z=3,scan#=30925,scan time=70.3033
Intensity
m/z
LNGTDALYVLTEDHTVYK
C6H8NO2
C7H8NO2
HexNAc
HexNAc-36
HexNAc-18
HexNAcHex
Pep_2+
Pep+HexNAc_2+
Pep+2HexNAc+3Hex_2+
y2
y3
y4
y5
y6
y7
y8
y9
y10
y11
y12
y13
y14
y16
y12++
y13++
y16++
b2
b3
b4
b5
b6
b7
b8
b9
a2
a6

93

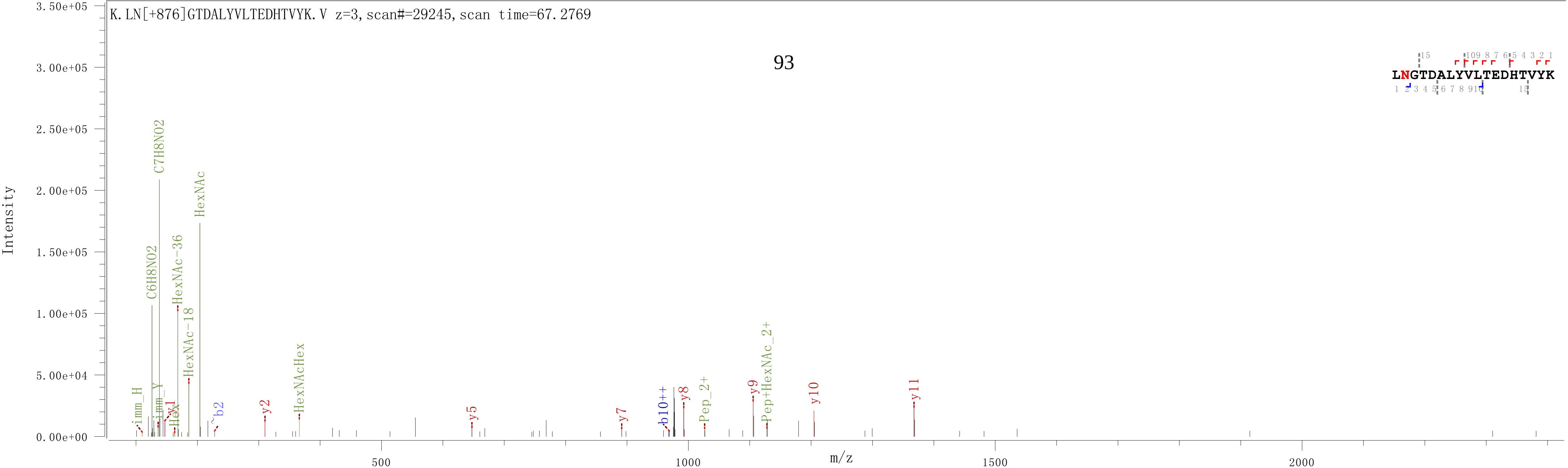

K.LN[+876]GTDALYVLTEDHTVYK.V z=3,scan#=29245,scan time=67.2769
LNGTDALYVLTEDHTVYK
Intensity
m/z
C7H8NO2
HexNAc
C6H8NO2
HexNAc-36
HexNAc-18
HexNAcHex
imm_H
imm_Y
Hex
y1
^b2
y2
y5
y7
b10++
y8
Pep_2+
y9
Pep+HexNAc_2+
y10
y11

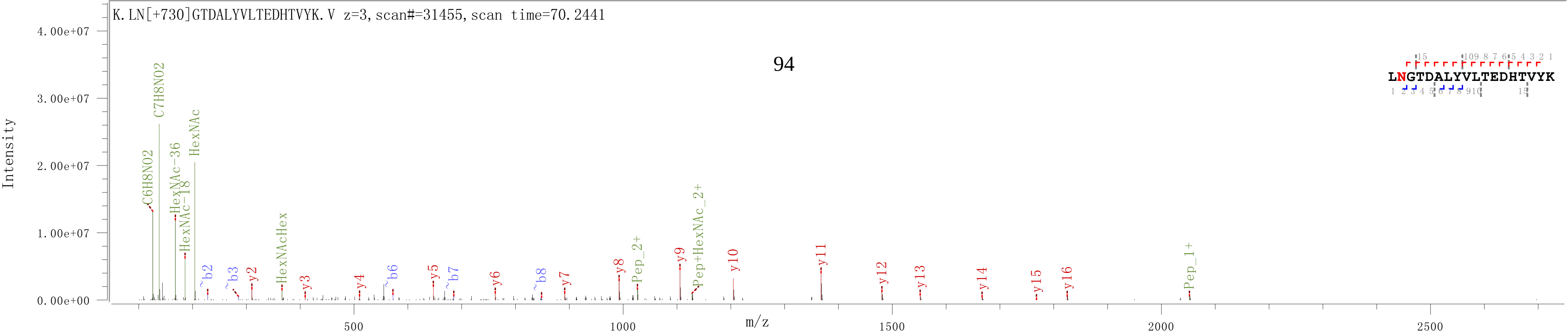
K.LN[+730]GTDALYVLTEDHTVYK.V z=3,scan#=31455,scan time=70.2441
94
LNGTDALYVLTEDHTVYK
Intensity
m/z
C6H8NO2
C7H8NO2
HexNAc-36
HexNAc-18
HexNAc
HexNAcHex
Pep_2+
Pep+HexNAc_2+
Pep_1+
y2
y3
y4
y5
y6
y7
y8
y9
y10
y11
y12
y13
y14
y15
y16
~b2
~b3
~b6
~b7
~b8
4.00e+07
3.00e+07
2.00e+07
1.00e+07
0.00e+00
500
1000
1500
2000
2500

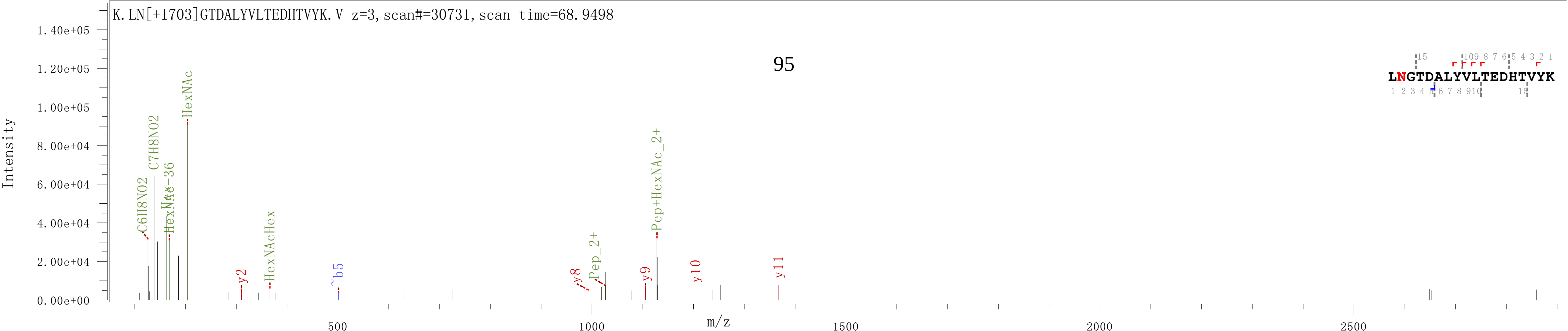

K.LN[+1703]GTDALYVLTEDHTVYK.V z=3,scan#=30731,scan time=68.9498
95
Intensity
m/z
LNGTDALYVLTEDHTVYK
C6H8NO2
C7H8NO2
HexNAc-36
Hex
HexNAc
HexNAcHex
y2
~b5
y8
Pep_2+
y9
Pep+HexNAc_2+
y10
y11

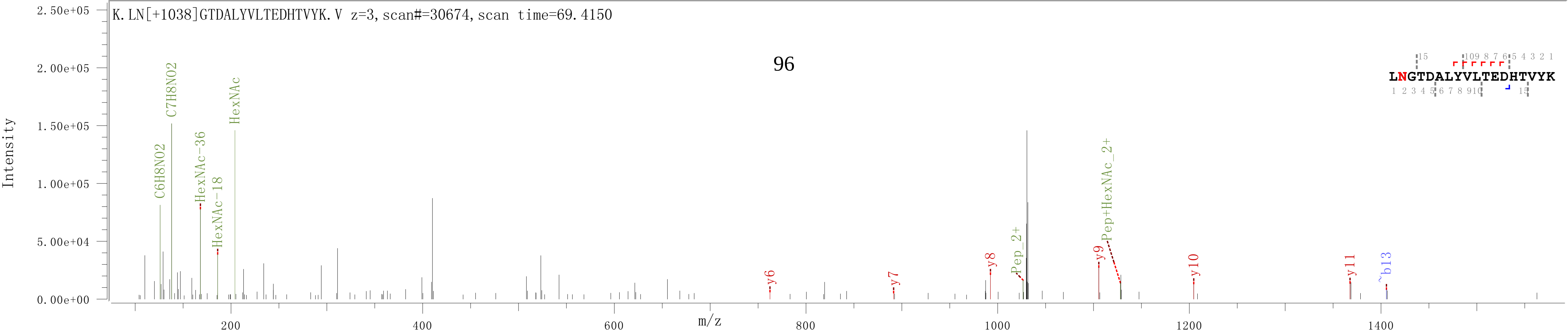

K.LN[+1038]GTDALYVLTEDHTVYK.V z=3,scan#=30674,scan time=69.4150
96
LNGTDALYVLTEDHTVYK
C6H8NO2
C7H8NO2
HexNAc-36
HexNAc-18
HexNAc
y6
y7
y8
Pep_2+
y9
Pep+HexNAc_2+
y10
y11
~b13
Intensity
m/z

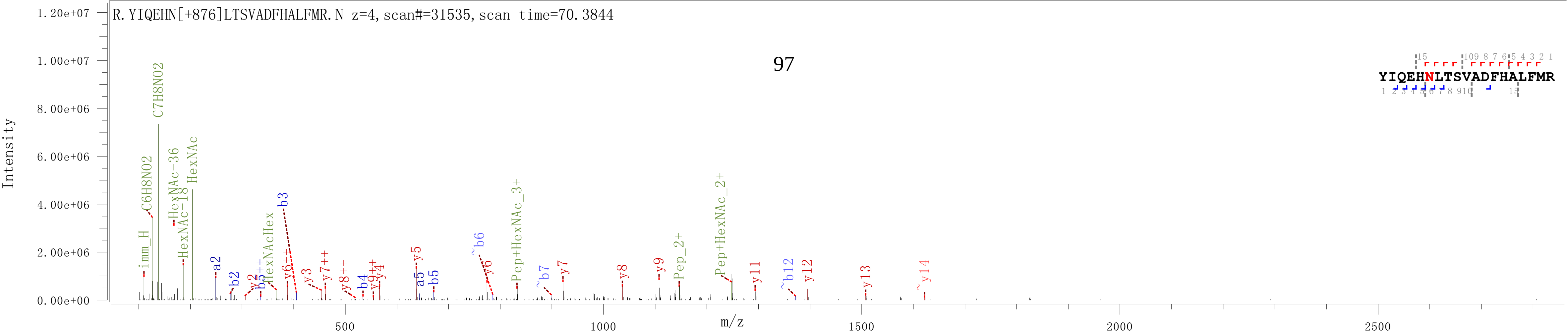
R.YIQEHN[+876]LTSVADFHALFMR.N z=4,scan#=31535,scan time=70.3844
YIQEHNLTSVADFHALFMR
Intensity
m/z
1.20e+07
1.00e+07
8.00e+06
6.00e+06
4.00e+06
2.00e+06
0.00e+00
500
1000
1500
2000
2500
imm_H
C6H8NO2
C7H8NO2
HexNAc-36
HexNAc-18
HexNAc
a2
b2
y2
b5++
HexNAcHex
y6++
b3
y3
y7++
y8++
b4
y9++
y4
a5
y5
b5
~b6
y6
Pep+HexNAc_3+
~b7
y7
y8
y9
Pep_2+
Pep+HexNAc_2+
y11
~b12
y12
y13
~y14

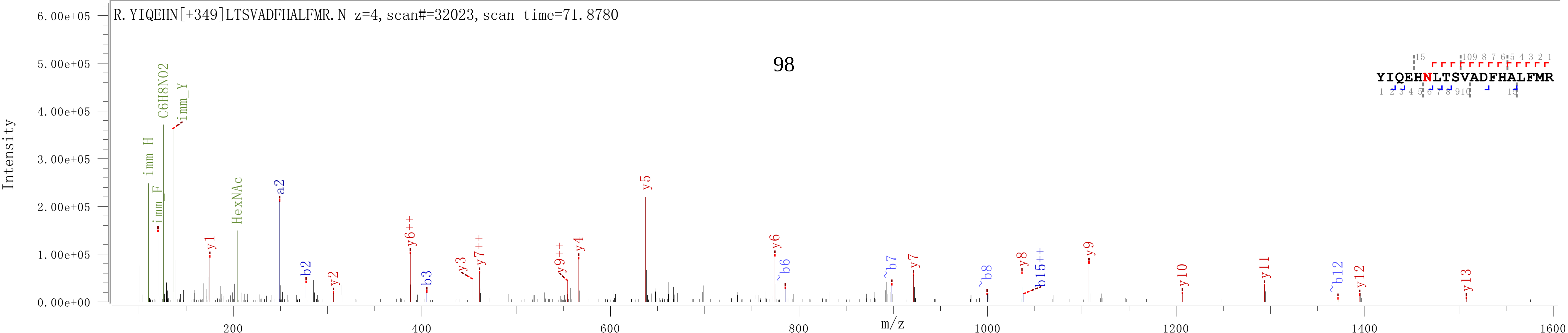
R.YIQEHN[+349]LTSVADFHALFMR.N z=4,scan#=32023,scan time=71.8780
98
YIQEHNLTSVADFHALFMR
Intensity
m/z
imm_H
imm_F
C6H8NO2
imm_Y
HexNAc
a2
b2
b3
y1
y2
y3
y4
y5
y6
y6++
y7
y7++
y8
y9
y9++
y10
y11
y12
y13
~b6
~b7
~b8
~b12
b15++

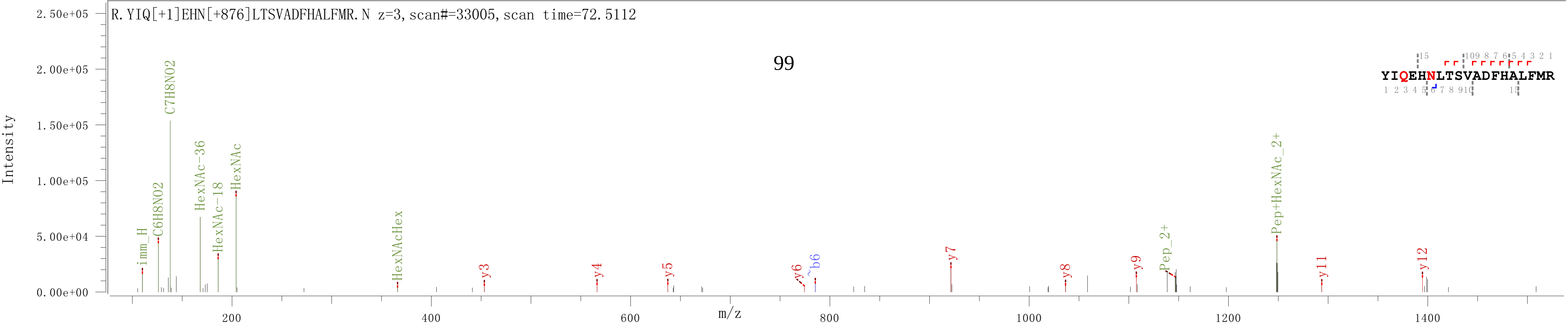
R.YIQ[+1]EHN[+876]LTSVADFHALFMR.N z=3,scan#=33005,scan time=72.5112
99
YIQEHNLTSVADFHALFMR
Intensity
m/z
2.50e+05
2.00e+05
1.50e+05
1.00e+05
5.00e+04
0.00e+00
200
400
600
800
1000
1200
1400
imm_H
C6H8NO2
C7H8NO2
HexNAc-36
HexNAc-18
HexNAc
HexNAcHex
y3
y4
y5
y6
~b6
y7
y8
y9
Pep_2+
Pep+HexNAc_2+
y11
y12

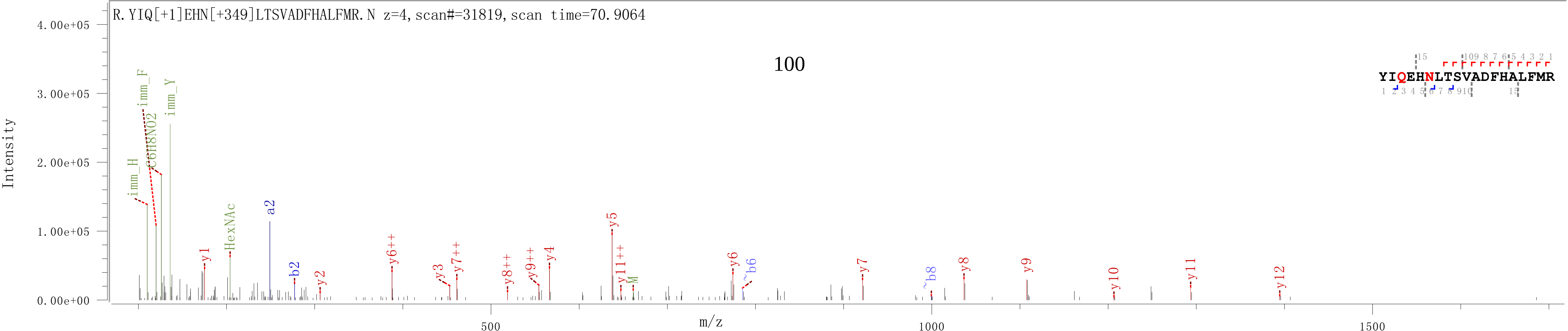
R.YIQ[+1]EHN[+349]LTSVADFHALFMR.N z=4,scan#=31819,scan time=70.9064
100
YIQEHNLTSVADFHALFMR
Intensity
m/z
imm_H
imm_F
C8H8NO2
imm_Y
y1
HexNAc
a2
b2
y2
y6++
y3
y7++
y8++
y9++
y4
y5
y11++
M
y6
~b6
y7
~b8
y8
y9
y10
y11
y12
0.00e+00
1.00e+05
2.00e+05
3.00e+05
4.00e+05
500
1000
1500

101

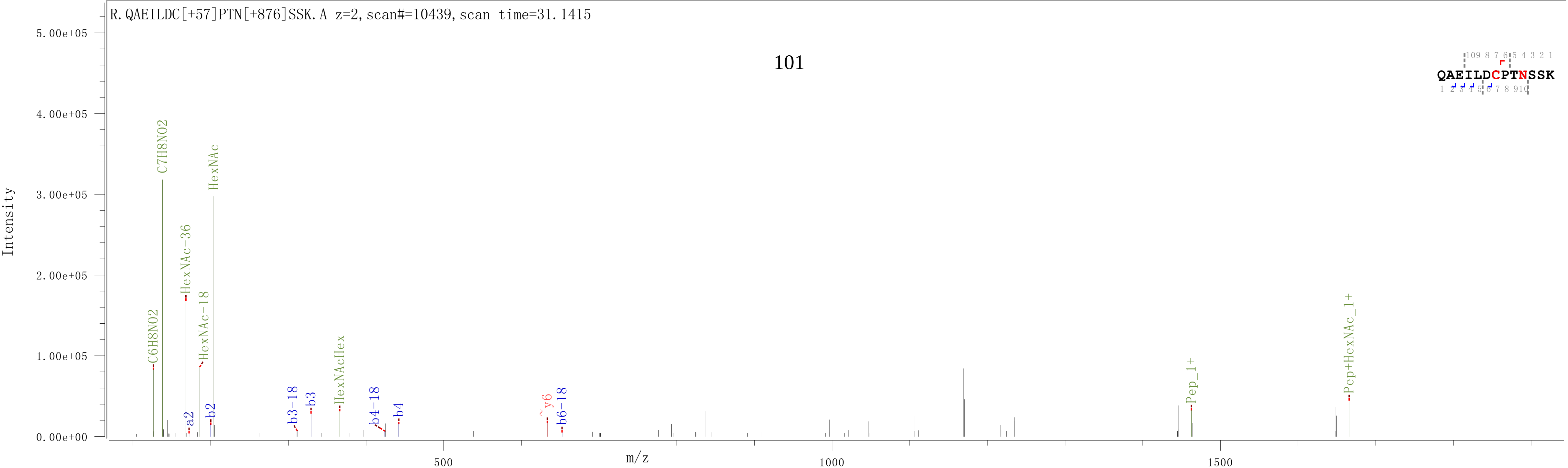
R.QAEILDC[+57]PTN[+876]SSK.A z=2,scan#=10439,scan time=31.1415
QAEILDCPTNSSK
Intensity
m/z
C6H8NO2
C7H8NO2
HexNAc-36
a2
HexNAc-18
b2
HexNAc
b3-18
b3
HexNAcHex
b4-18
b4
~y6
b6-18
Pep_1+
Pep+HexNAc_1+
5.00e+05
4.00e+05
3.00e+05
2.00e+05
1.00e+05
0.00e+00
500
1000
1500

102

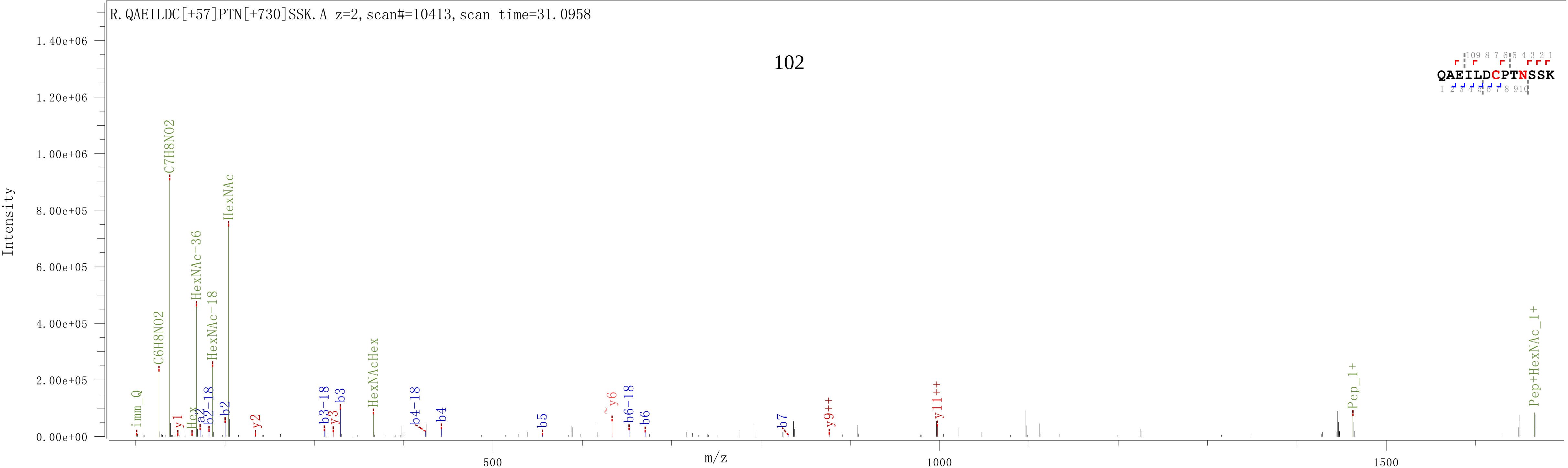

R.QAEILDC[+57]PTN[+730]SSK.A z=2,scan#=10413,scan time=31.0958
Intensity
m/z
QAEILDCPTNSSK
C7H8NO2
HexNAc
HexNAc-36
HexNAc-18
C6H8NO2
imm_Q
y1
Hex
a2
b2-18
b2
y2
b3-18
y3
b3
HexNAcHex
b4-18
b4
b5
~y6
b6-18
b6
b7
y9++
y11++
Pep_1+
Pep+HexNAc_1+

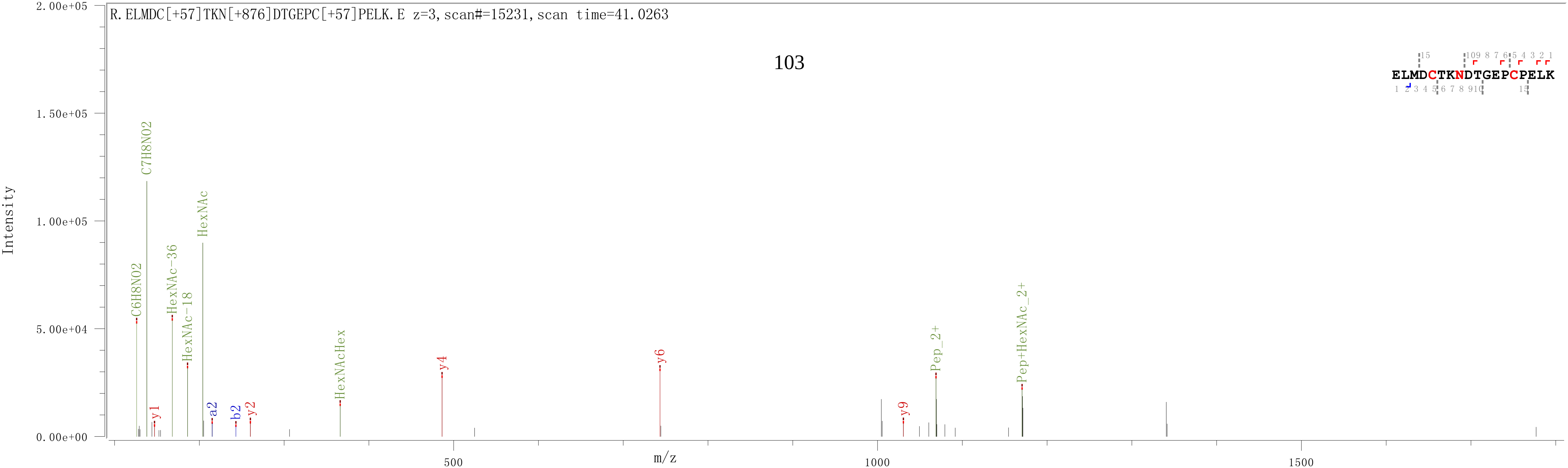

R.ELMDC[+57]TKN[+876]DTGEPC[+57]PELK.E z=3,scan#=15231,scan time=41.0263
103
ELMDCTKNDTGEPCPELK
Intensity
m/z
C6H8NO2
C7H8NO2
y1
HexNAc-36
HexNAc-18
HexNAc
a2
b2
y2
HexNAcHex
y4
y6
y9
Pep_2+
Pep+HexNAc_2+
2.00e+05
1.50e+05
1.00e+05
5.00e+04
0.00e+00
500
1000
1500

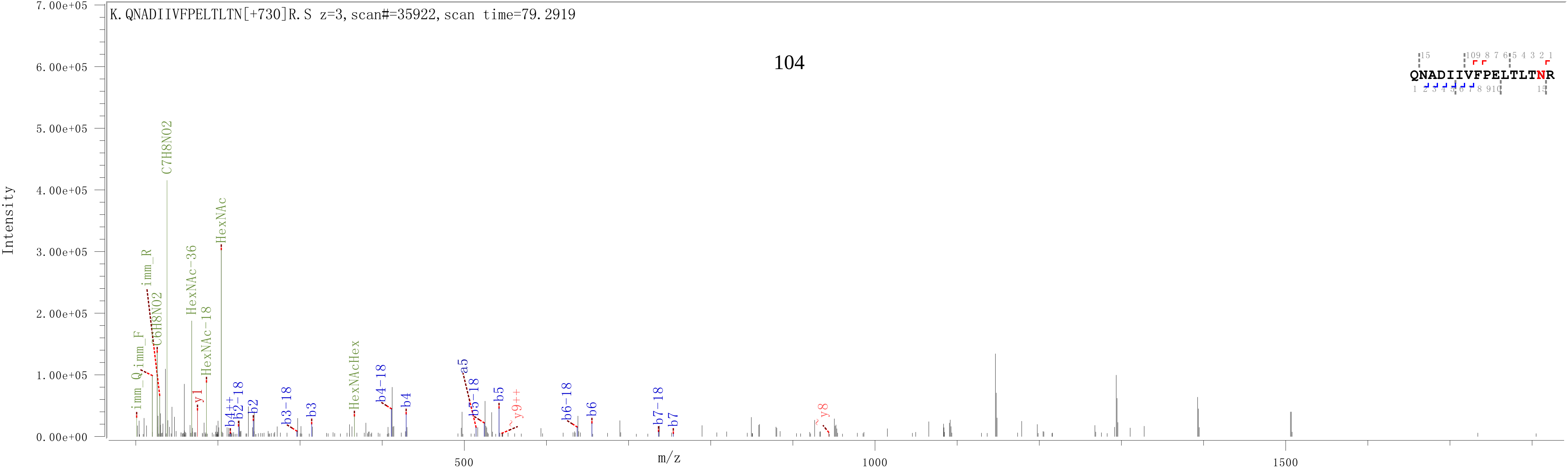

K.QNADIIVFPELTLTN[+730]R.S z=3,scan#=35922,scan time=79.2919
104
QNADIIVFPELTLTNR
Intensity
m/z
7.00e+05
6.00e+05
5.00e+05
4.00e+05
3.00e+05
2.00e+05
1.00e+05
0.00e+00
500
1000
1500
C7H8NO2
HexNAc
HexNAc-36
HexNAc-18
imm_R
C6H8NO2
imm_F
imm_Q
y1
b4++
b2-18
b2
b3-18
b3
HexNAcHex
b4-18
b4
a5
b5-18
b5
y9++
b6-18
b6
b7-18
b7
y8

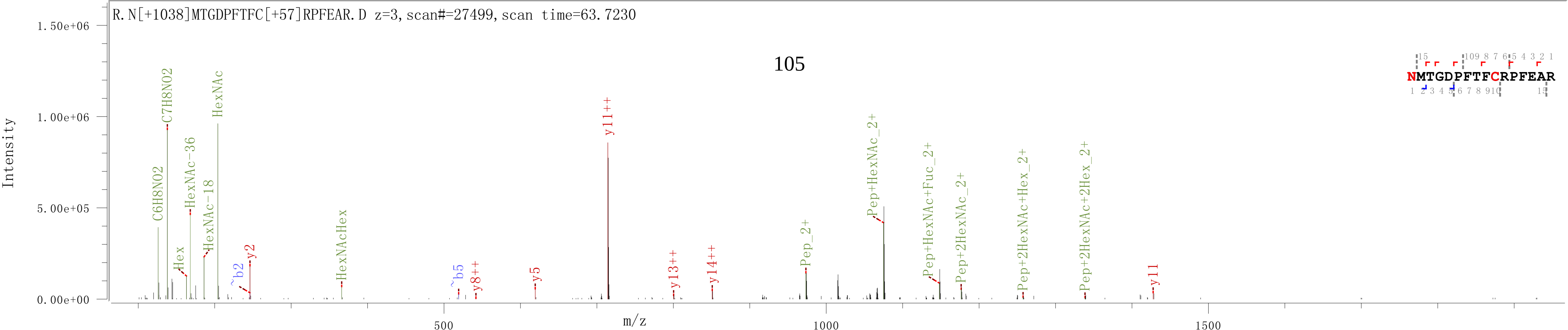

R.N[+1038]MTGDPFTFC[+57]RPFEAR.D z=3,scan#=27499,scan time=63.7230
105
Intensity
m/z
C6H8NO2
C7H8NO2
Hex
HexNAc-36
HexNAc-18
HexNAc
~b2
y2
HexNAcHex
~b5
y8++
y5
y11++
y13++
y14++
Pep_2+
Pep+HexNAc_2+
Pep+HexNAc+Fuc_2+
Pep+2HexNAc_2+
Pep+2HexNAc+Hex_2+
Pep+2HexNAc+2Hex_2+
y11
NMTGDPFTFCRPFEAR

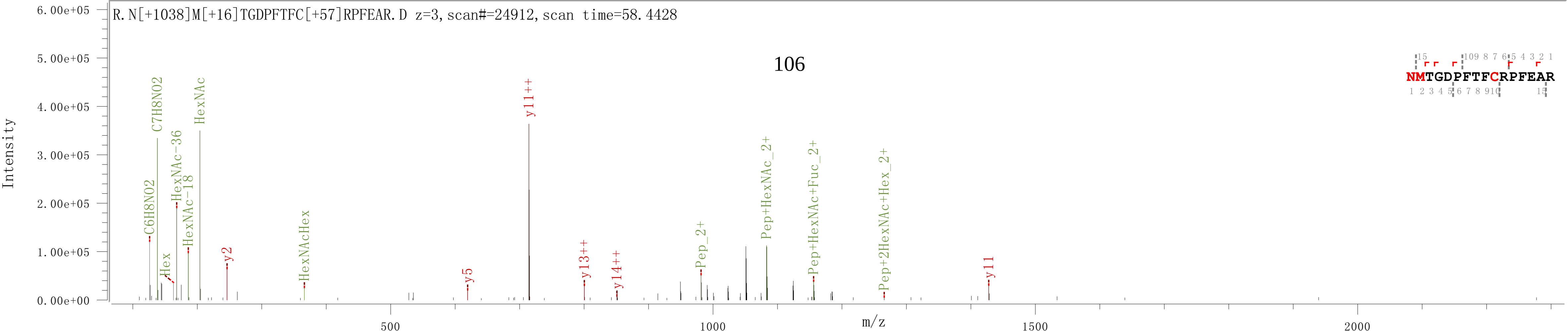
R.N[+1038]M[+16]TGDPFTFC[+57]RPFEAR.D z=3,scan#=24912,scan time=58.4428
106
Intensity
m/z
C6H8NO2
C7H8NO2
Hex
HexNAc-36
HexNAc-18
HexNAc
y2
HexNAcHex
y5
y11++
y13++
y14++
Pep_2+
Pep+HexNAc_2+
Pep+HexNAc+Fuc_2+
Pep+2HexNAc+Hex_2+
y11
NMTGDPFTFCRPFEAR

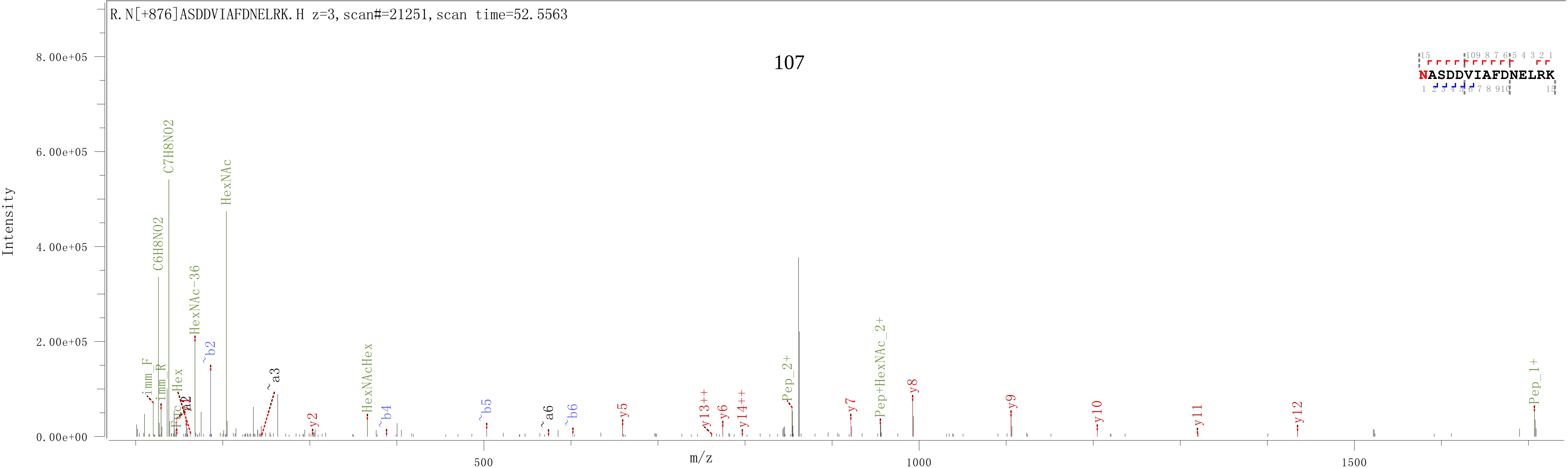
R.N[+876]ASDDVIAFDNELRK.H z=3,scan#=21251,scan time=52.5563
Intensity
m/z
NASDDVIAFDNELRK
C7H8NO2
HexNAc
C6H8NO2
HexNAc-36
~b2
imm_F
imm_R
Hex
a2
~a3
y2
HexNAcHex
~b4
~b5
~a6
~b6
y5
y13++
y6
y14++
Pep_2+
y7
Pep+HexNAc_2+
y8
y9
y10
y11
y12
Pep_1+

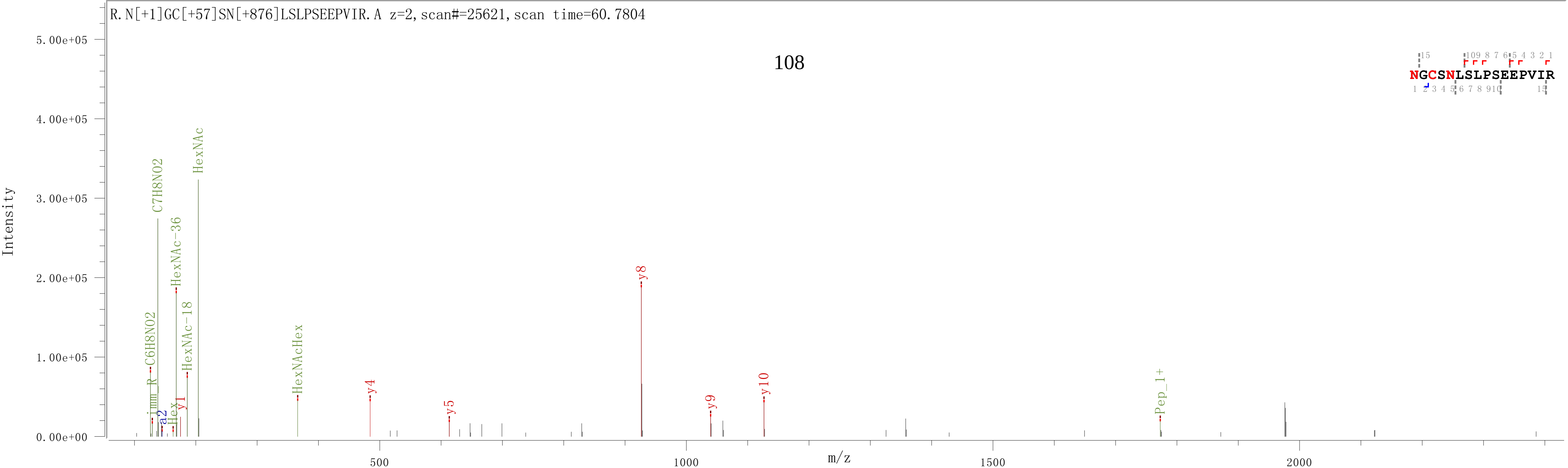

R.N[+1]GC[+57]SN[+876]LSLPSEEPVIR.A z=2,scan#=25621,scan time=60.7804
108
NGCSNLSLPSEEPVIR
Intensity
m/z
HexNAc
C7H8NO2
HexNAc-36
HexNAc-18
C6H8NO2
HexNAcHex
y8
y4
y5
y9
y10
Pep_1+
y1
a2
Hex

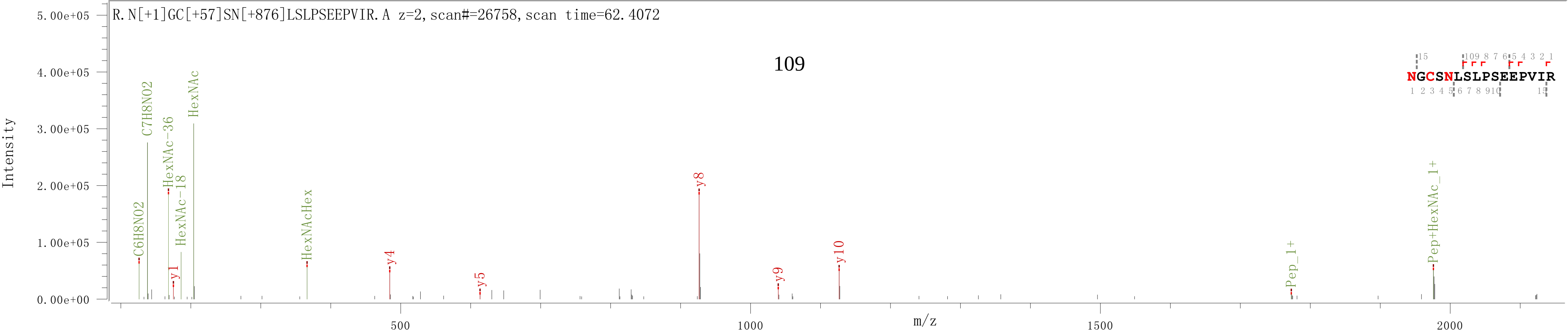
R.N[+1]GC[+57]SN[+876]LSLPSEEPVIR.A z=2,scan#=26758,scan time=62.4072
109
NGCSNLSLPSEEPVIR
Intensity
m/z
C6H8NO2
C7H8NO2
HexNAc-36
HexNAc-18
HexNAc
HexNAcHex
y1
y4
y5
y8
y9
y10
Pep_1+
Pep+HexNAc_1+

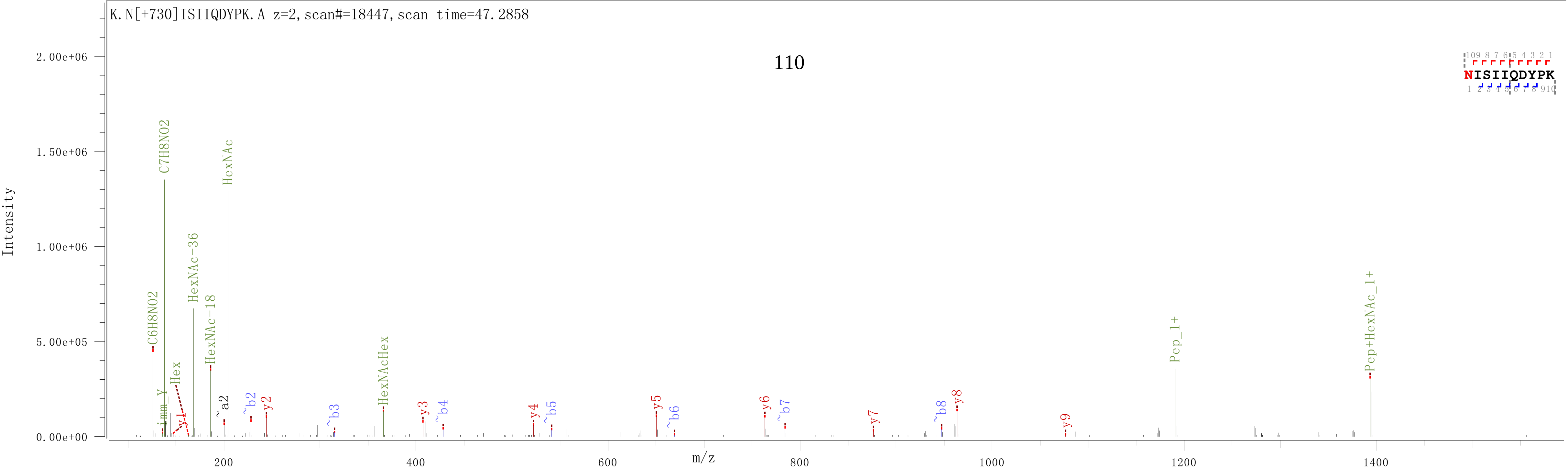

K.N[+730]ISIIQDYPK.A z=2,scan#=18447,scan time=47.2858
110
NISIIQDYPK
Intensity
m/z
C6H8NO2
C7H8NO2
imm_Y
Hex
y1
HexNAc-36
HexNAc-18
~a2
HexNAc
~b2
y2
~b3
HexNAcHex
y3
~b4
y4
~b5
y5
~b6
y6
~b7
y7
~b8
y8
y9
Pep_1+
Pep+HexNAc_1+

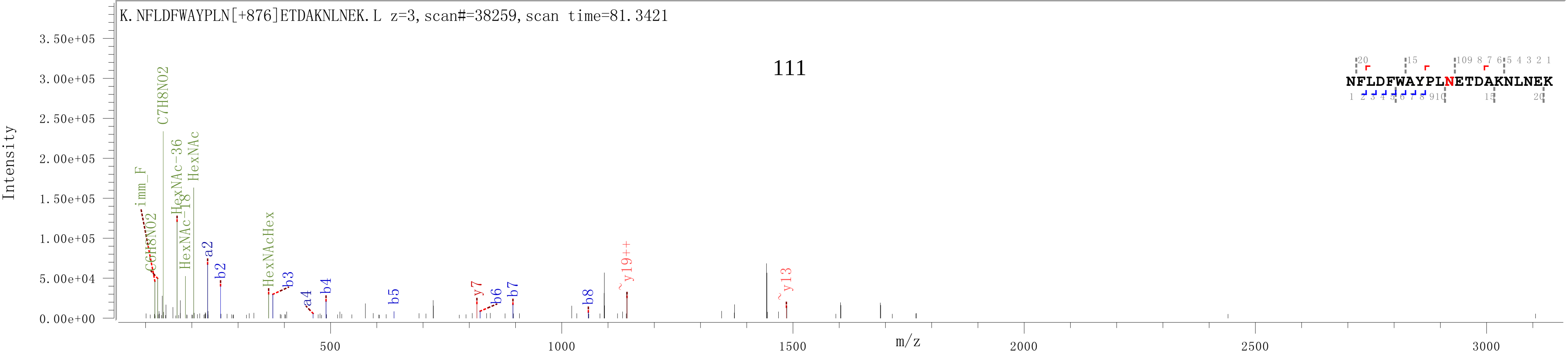
K.NFLDFWAYPLN[+876]ETDAKNLNEK.L z=3,scan#=38259,scan time=81.3421
111
NFLDFWAYPLNETDAKNLNEK
Intensity
m/z
imm_F
C7H8NO2
HexNAc-36
HexNAc
HexNAc-18
C6H8NO2
HexNAcHex
a2
b2
b3
a4
b4
b5
y7
b6
b7
b8
~y19++
~y13

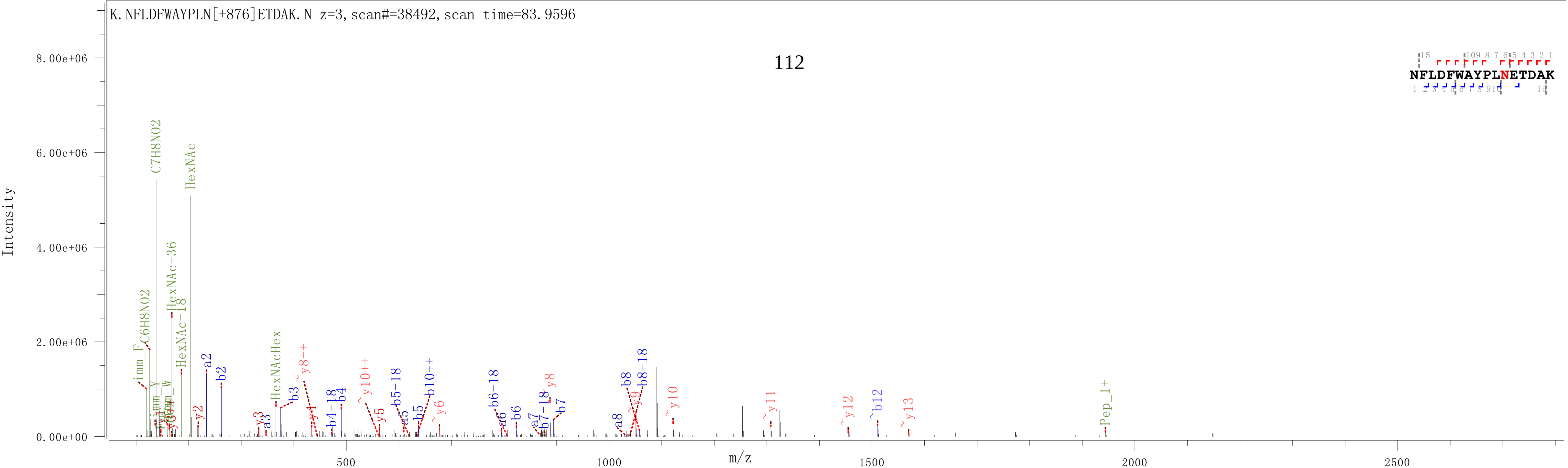

K.NFLDFWAYPLN[+876]ETDAK.N z=3,scan#=38492,scan time=83.9596
NFLDFWAYPLNETDAK
Intensity
m/z

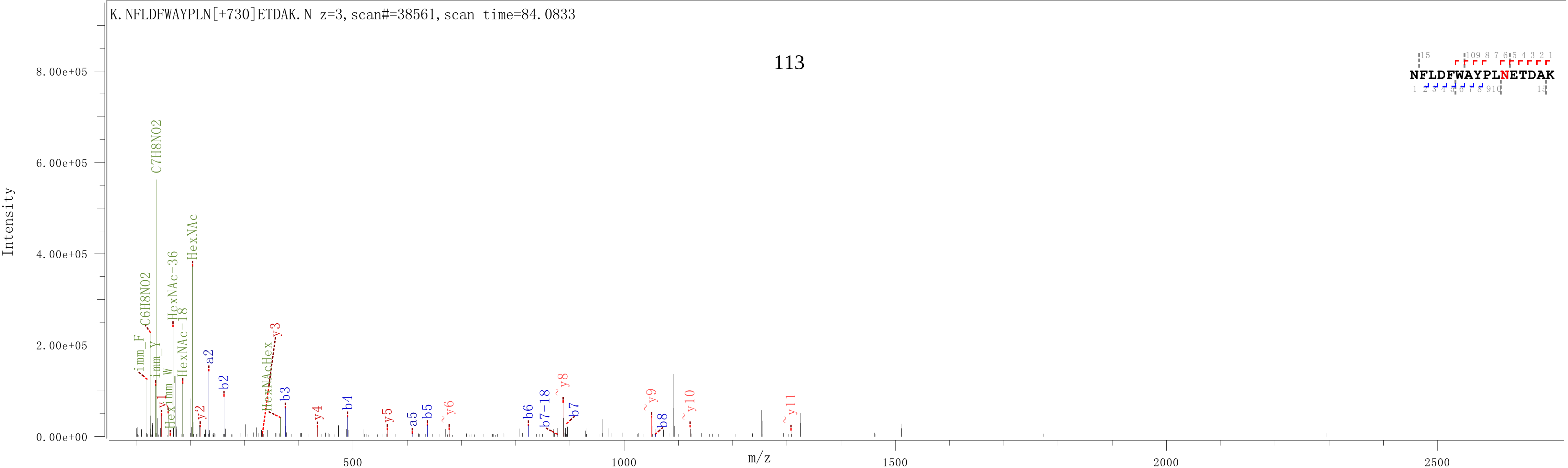
K.NFLDFWAYPLN[+730]ETDAK.N z=3,scan#=38561,scan time=84.0833
113
NFLDFWAYPLNETDAK
Intensity
m/z
8.00e+05
6.00e+05
4.00e+05
2.00e+05
0.00e+00
500
1000
1500
2000
2500
C7H8NO2
C6H8NO2
HexNAc
HexNAc-36
HexNAc-18
imm_F
imm_Y
imm_W
HexNAcHex
y3
a2
b2
b3
b4
y2
y4
y5
a5
b5
~y6
b6
b7-18
~y8
b7
~y9
b8
~y10
~y11

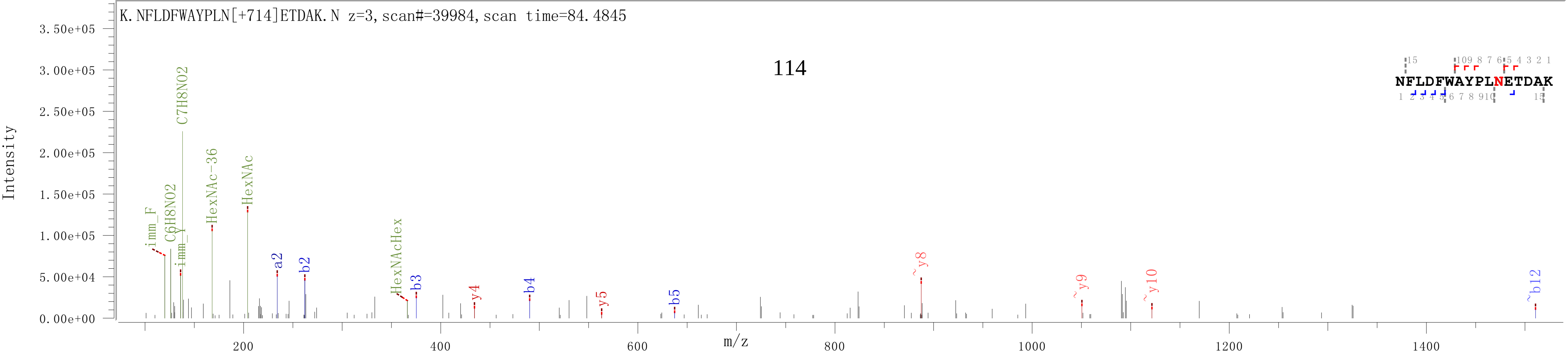
K.NFLDFWAYPLN[+714]ETDAK.N z=3,scan#=39984,scan time=84.4845
114
NFLDFWAYPLNETDAK
Intensity
m/z
imm_F
C6H8NO2
imm_Y
C7H8NO2
HexNAc-36
HexNAc
a2
b2
HexNAcHex
b3
y4
b4
y5
b5
~y8
~y9
~y10
~b12

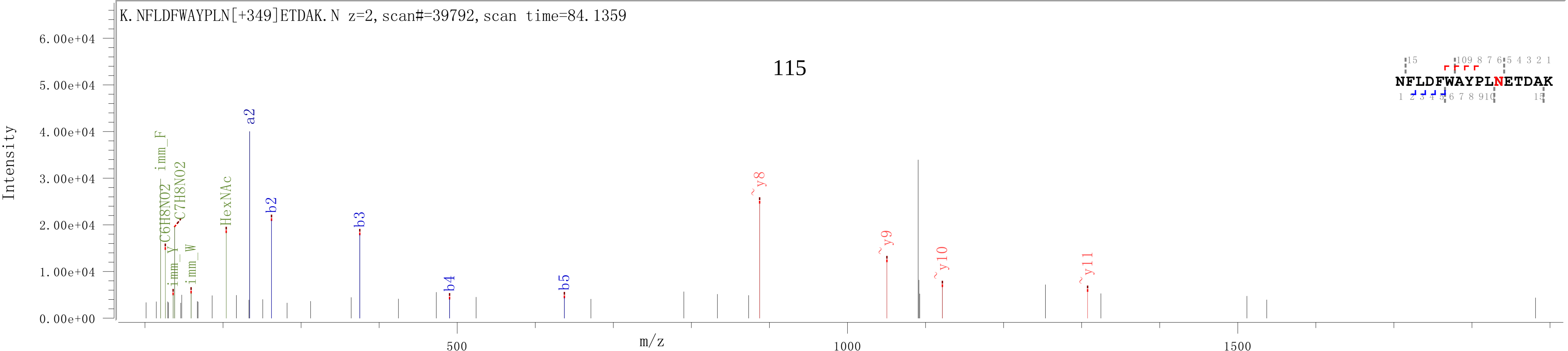

K.NFLDFWAYPLN[+349]ETDAK.N z=2,scan#=39792,scan time=84.1359
115
Intensity
m/z
6.00e+04
5.00e+04
4.00e+04
3.00e+04
2.00e+04
1.00e+04
0.00e+00
500
1000
1500
NFLDFWAYPLNETDAK
a2
b2
b3
b4
b5
~y8
~y9
~y10
~y11
HexNAc
imm_F
C6H8NO2
C7H8NO2
imm_Y
imm_W

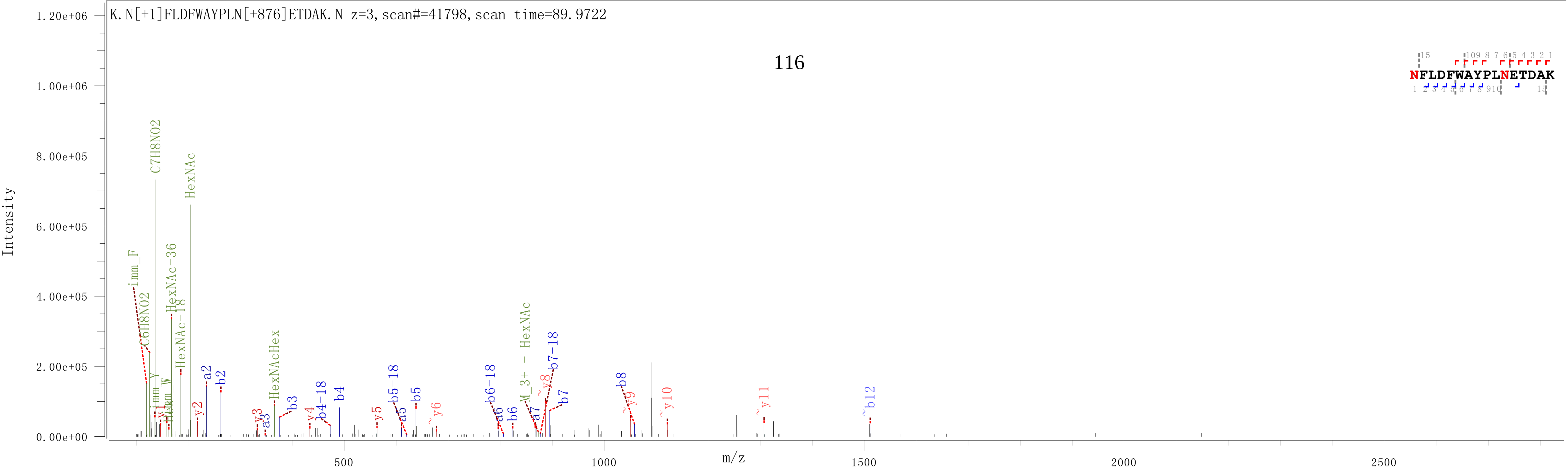

K.N[+1]FLDFWAYPLN[+876]ETDAK.N z=3,scan#=41798,scan time=89.9722
NFLDFWAYPLNETDAK
Intensity
m/z
1.20e+06
1.00e+06
8.00e+05
6.00e+05
4.00e+05
2.00e+05
0.00e+00
500
1000
1500
2000
2500
imm_F
C6H8NO2
C7H8NO2
HexNAc
HexNAc-36
HexNAc-18
HexNAcHex
a2
b2
y2
y3
a3
b3
y4
b4-18
b4
y5
b5-18
a5
b5
~y6
b6-18
a6
b6
M_3+ - HexNAc
a7
~y8
b7-18
b7
b8
~y9
~y10
~y11
~b12

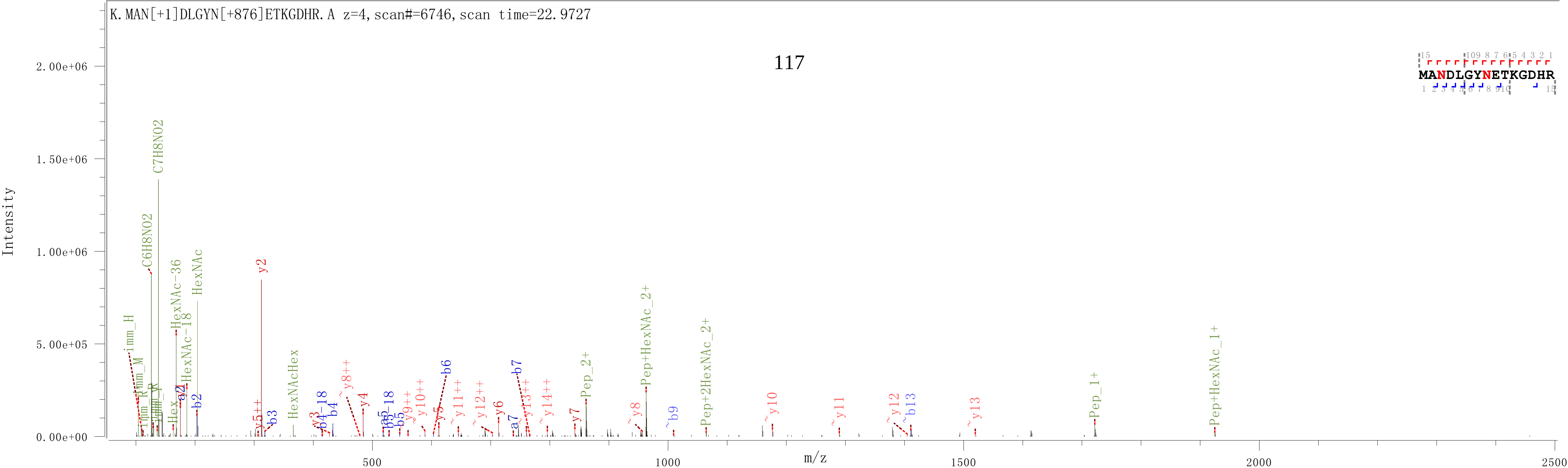

K.MAN[+1]DLGYN[+876]ETKGDHR.A z=4,scan#=6746,scan time=22.9727
MANDLGYNETKGDHR
Intensity
m/z
2.00e+06
1.50e+06
1.00e+06
5.00e+05
0.00e+00
500
1000
1500
2000
2500
C7H8NO2
C6H8NO2
imm_H
HexNAc-36
HexNAc-18
HexNAc
HexNAcHex
y2
b2
b3
b6
b7
Pep_2+
Pep+HexNAc_2+
Pep+2HexNAc_2+
~y10
~y11
~y12
~b13
~y13
Pep_1+
Pep+HexNAc_1+

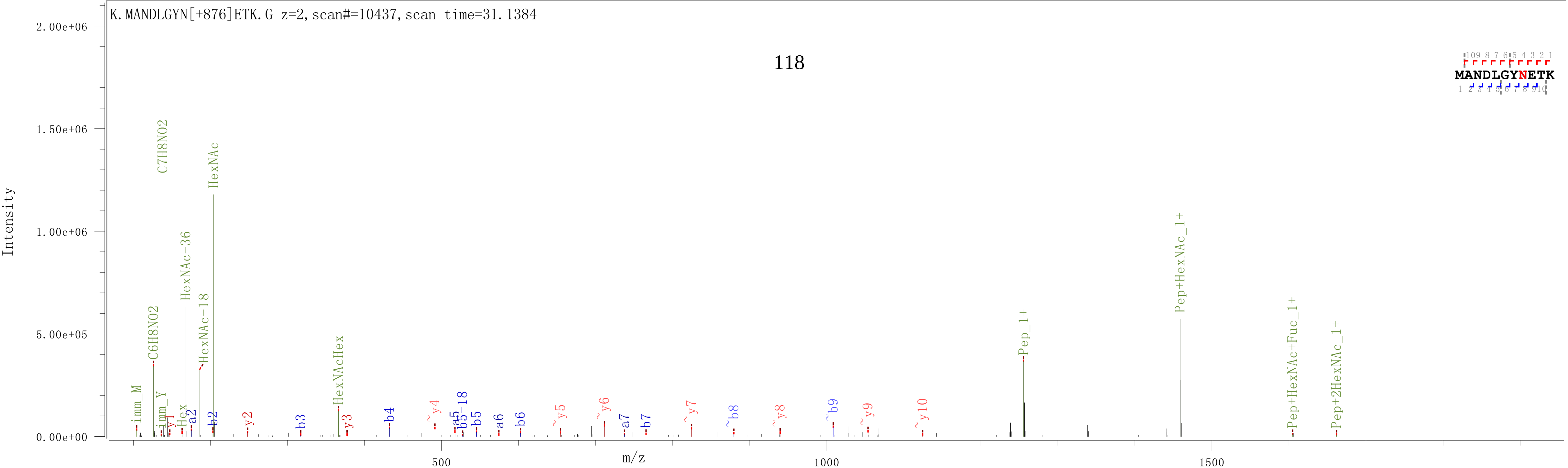

K.MANDLGYN[+876]ETK.G z=2,scan#=10437,scan time=31.1384
118
MANDLGYNETK
Intensity
m/z
2.00e+06
1.50e+06
1.00e+06
5.00e+05
0.00e+00
500
1000
1500
imm_M
C6H8NO2
imm_Y
C7H8NO2
y1
Hex
HexNAc-36
a2
HexNAc-18
b2
HexNAc
y2
b3
HexNAcHex
y3
b4
~y4
a5
b5-18
b5
a6
b6
~y5
~y6
a7
b7
~y7
~b8
~y8
~b9
~y9
~y10
Pep_1+
Pep+HexNAc_1+
Pep+HexNAc+Fuc_1+
Pep+2HexNAc_1+

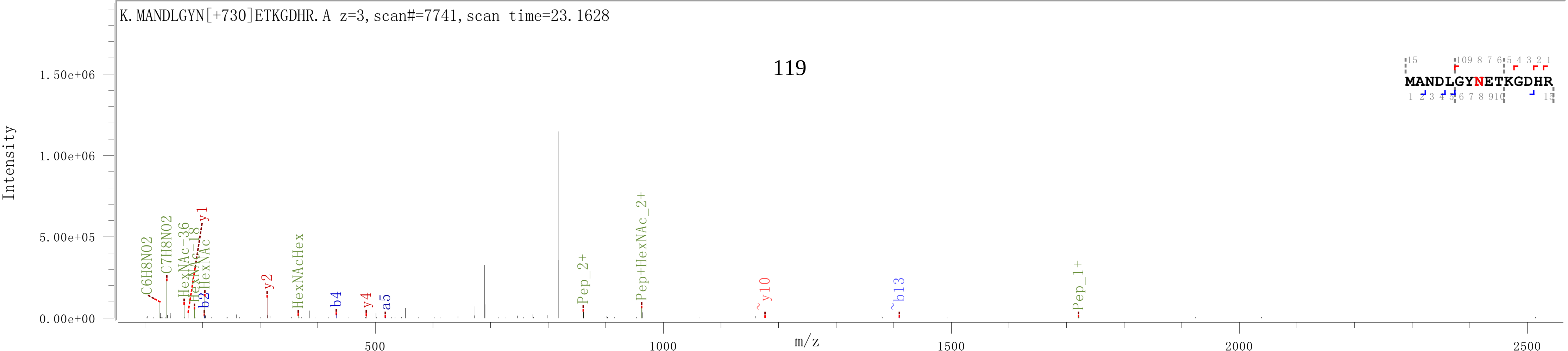
K.MANDLGYN[+730]ETKGDHR.A z=3,scan#=7741,scan time=23.1628
MANDLGYNETKGDHR
Intensity
m/z
0.00e+00
5.00e+05
1.00e+06
1.50e+06
500
1000
1500
2000
2500
C6H8NO2
C7H8NO2
HexNAc-36
HexNAc-18
HexNAc
b2
y1
y2
HexNAcHex
b4
y4
a5
Pep_2+
Pep+HexNAc_2+
~y10
~b13
Pep_1+

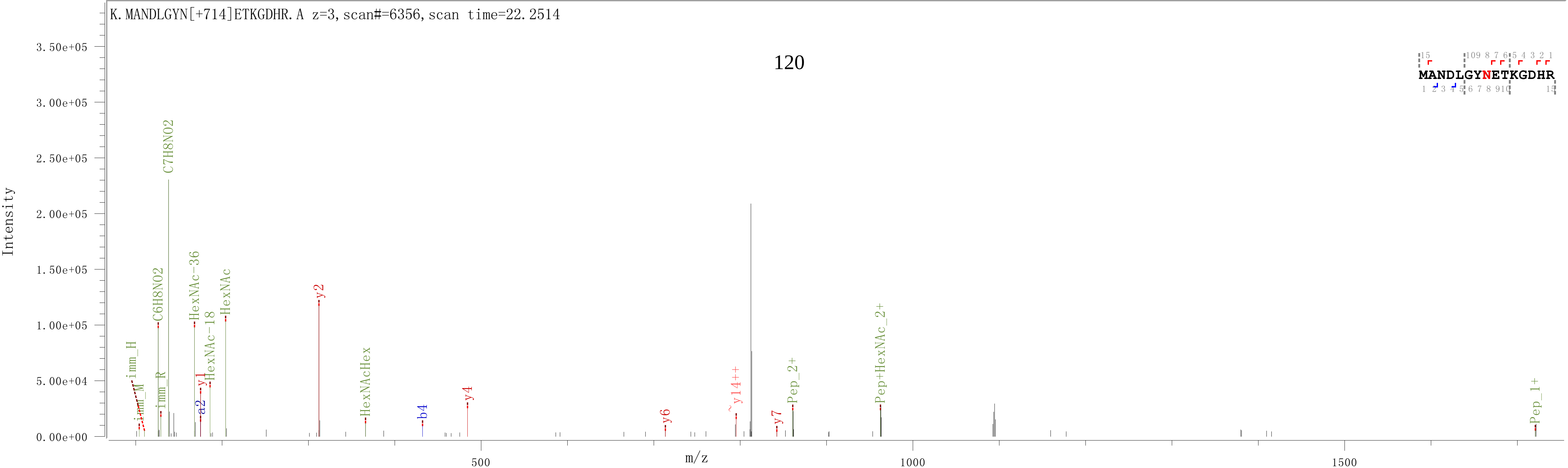
K.MANDLGYN[+714]ETKGDHR.A z=3,scan#=6356,scan time=22.2514
120
MANDLGYNETKGDHR
Intensity
m/z
C7H8NO2
C6H8NO2
HexNAc-36
HexNAc-18
HexNAc
imm_H
imm_M
imm_R
y1
a2
y2
HexNAcHex
b4
y4
y6
~y14++
y7
Pep_2+
Pep+HexNAc_2+
Pep_1+

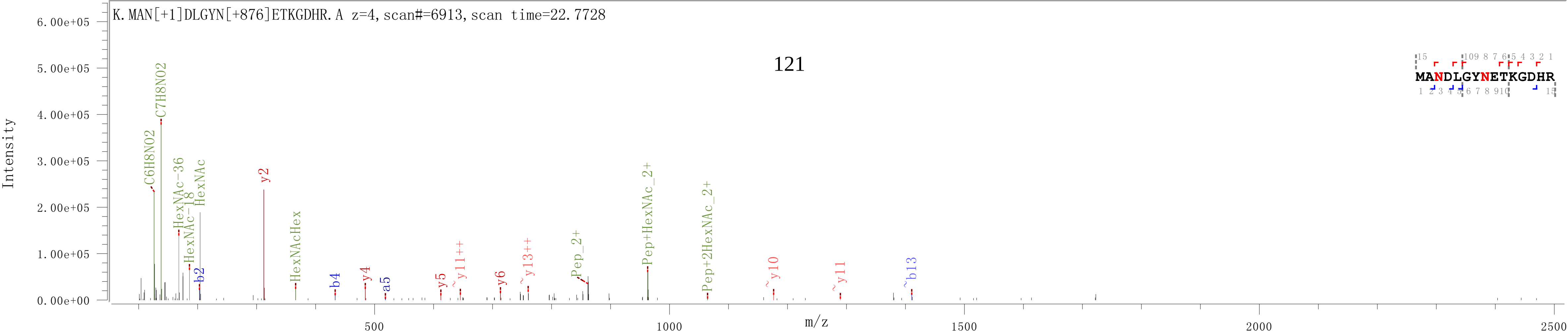

K.MAN[+1]DLGYN[+876]ETKGDHR.A z=4,scan#=6913,scan time=22.7728
121
MANDLGYNETKGDHR
Intensity
m/z
C6H8NO2
C7H8NO2
HexNAc-36
HexNAc-18
HexNAc
b2
y2
HexNAcHex
b4
y4
a5
y5
~y11++
y6
~y13++
Pep_2+
Pep+HexNAc_2+
Pep+2HexNAc_2+
~y10
~y11
~b13

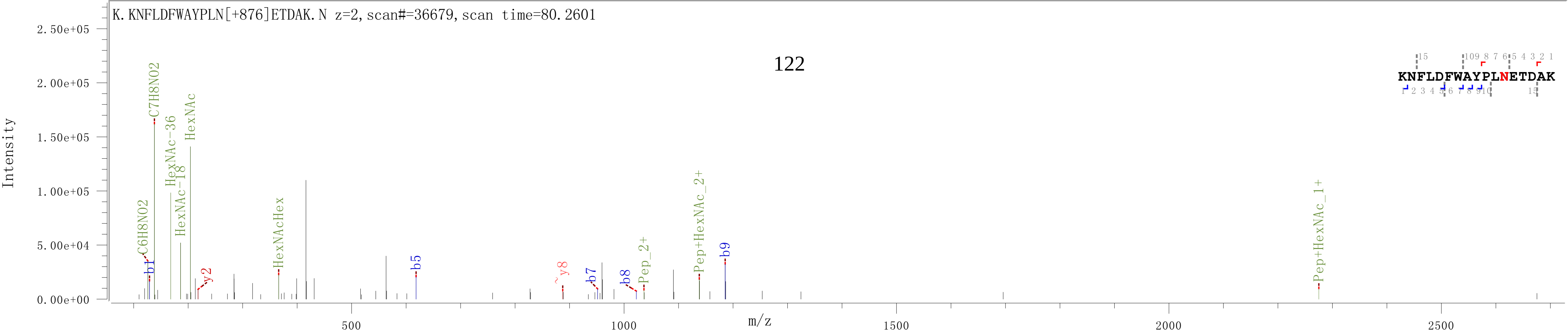
K.KNFLDFWAYPLN[+876]ETDAK.N z=2,scan#=36679,scan time=80.2601
122
KNFLDFWAYPLNETDAK
Intensity
m/z
2.50e+05
2.00e+05
1.50e+05
1.00e+05
5.00e+04
0.00e+00
500
1000
1500
2000
2500
C6H8NO2
b1
C7H8NO2
HexNAc-36
HexNAc-18
HexNAc
y2
HexNAcHex
b5
~y8
b7
b8
Pep_2+
Pep+HexNAc_2+
b9
Pep+HexNAc_1+

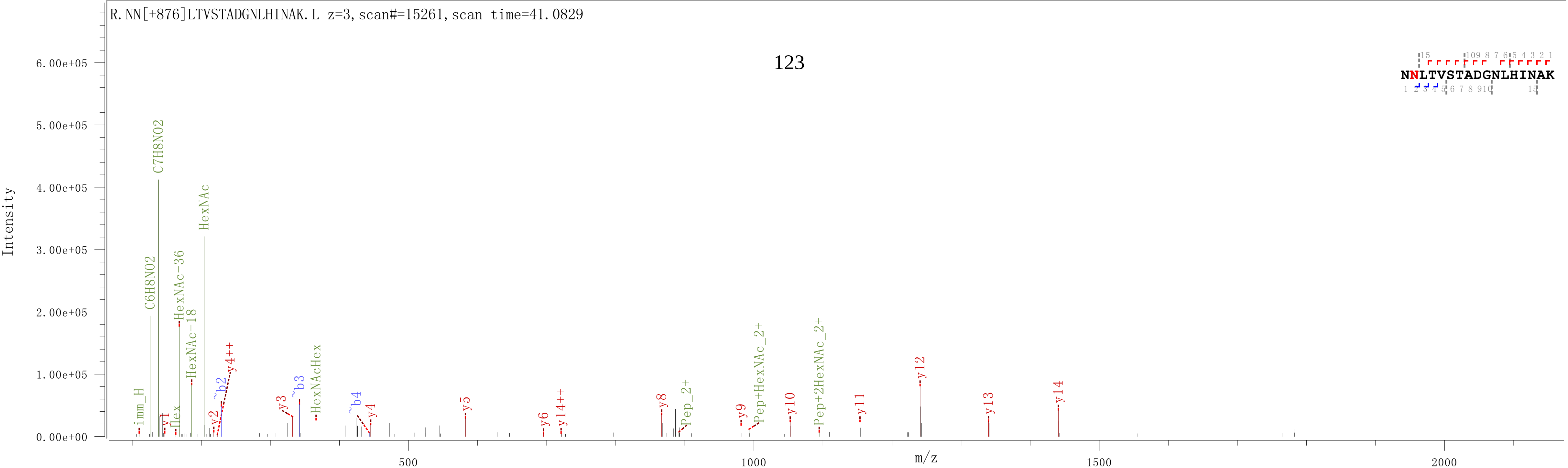
R.NN[+876]LTVSTADGNLHINAK.L z=3,scan#=15261,scan time=41.0829
NNLTVSTADGNLHINAK
Intensity
m/z
6.00e+05
5.00e+05
4.00e+05
3.00e+05
2.00e+05
1.00e+05
0.00e+00
500
1000
1500
2000
imm_H
C6H8NO2
C7H8NO2
y1
Hex
HexNAc-36
HexNAc-18
HexNAc
y2
~b2
y4++
y3
~b3
HexNAcHex
~b4
y4
y5
y6
y14++
y8
Pep_2+
y9
Pep+HexNAc_2+
y10
Pep+2HexNAc_2+
y11
y12
y13
y14

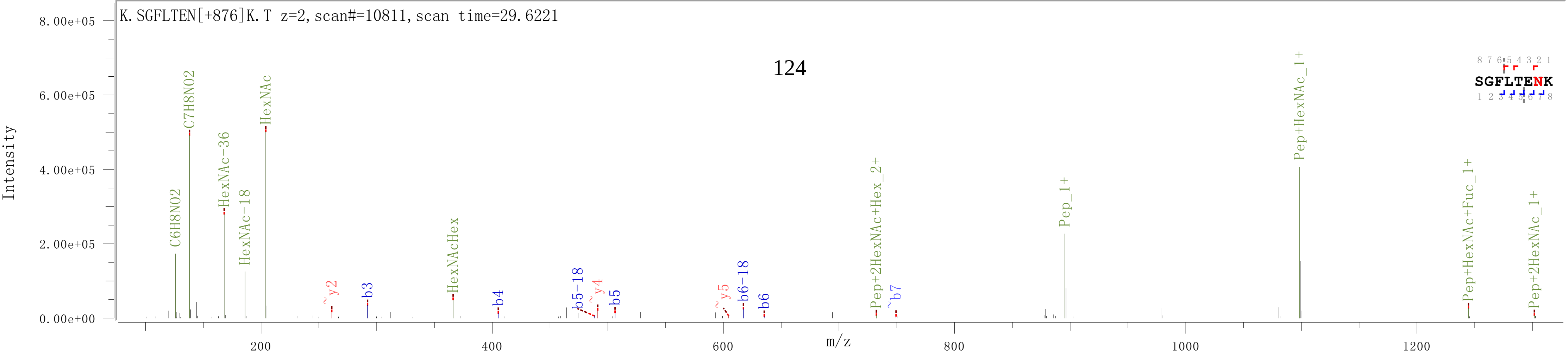

K.SGFLTEN[+876]K.T z=2,scan#=10811,scan time=29.6221
124
8.00e+05
6.00e+05
4.00e+05
2.00e+05
0.00e+00
Intensity
m/z
200
400
600
800
1000
1200
C6H8NO2
C7H8NO2
HexNAc-36
HexNAc-18
HexNAc
~y2
b3
HexNAcHex
b4
b5-18
~y4
b5
~y5
b6-18
b6
Pep+2HexNAc+Hex_2+
~b7
Pep_1+
Pep+HexNAc_1+
Pep+HexNAc+Fuc_1+
Pep+2HexNAc_1+
8 7 6 5 4 3 2 1
SGFLTENK
1 2 3 4 5 6 7 8

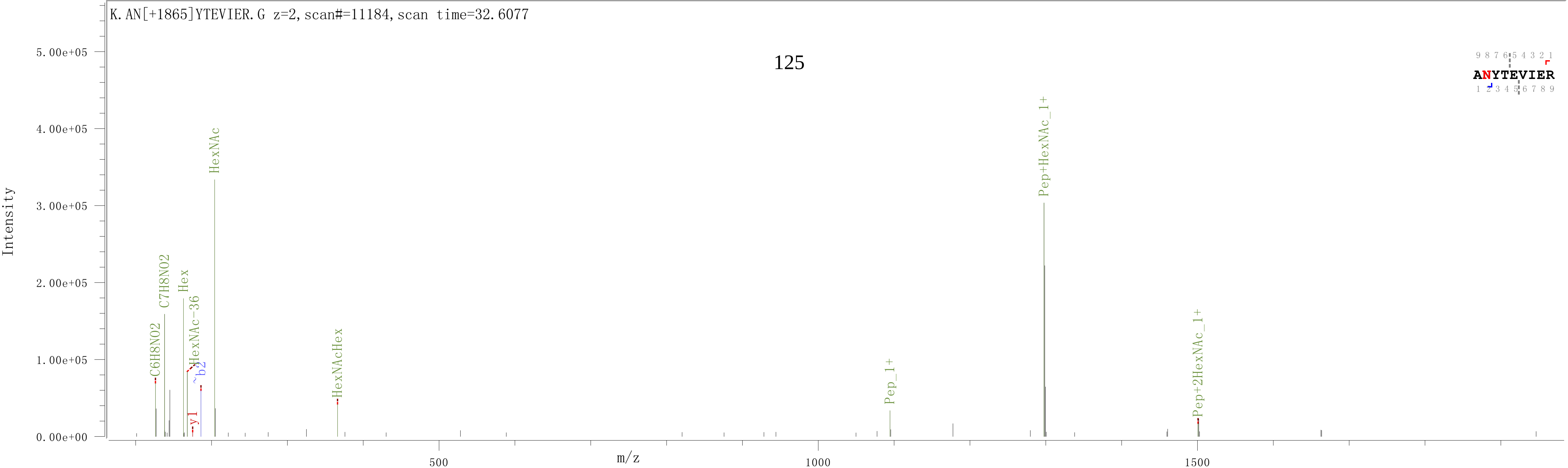
K.AN[+1865]YTEVIER.G z=2,scan#=11184,scan time=32.6077
125
ANYTEVIER
Intensity
m/z
HexNAc
Pep+HexNAc_1+
C7H8NO2
Hex
HexNAc-36
C6H8NO2
b2
y1
HexNAcHex
Pep_1+
Pep+2HexNAc_1+
500
1000
1500
0.00e+00
1.00e+05
2.00e+05
3.00e+05
4.00e+05
5.00e+05

126

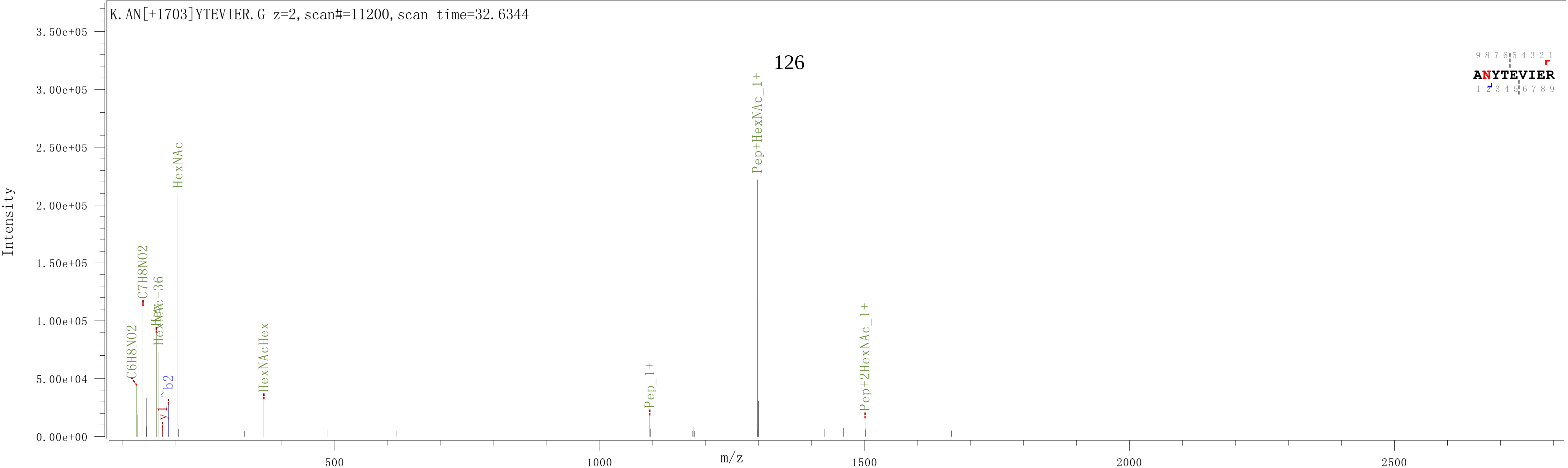

K.AN[+1703]YTEVIER.G z=2,scan#=11200,scan time=32.6344
Intensity
m/z
ANYTEVIER
C6H8NO2
C7H8NO2
HexNAc-36
Hex
HexNAc
y1
b2
HexNAcHex
Pep_1+
Pep+HexNAc_1+
Pep+2HexNAc_1+

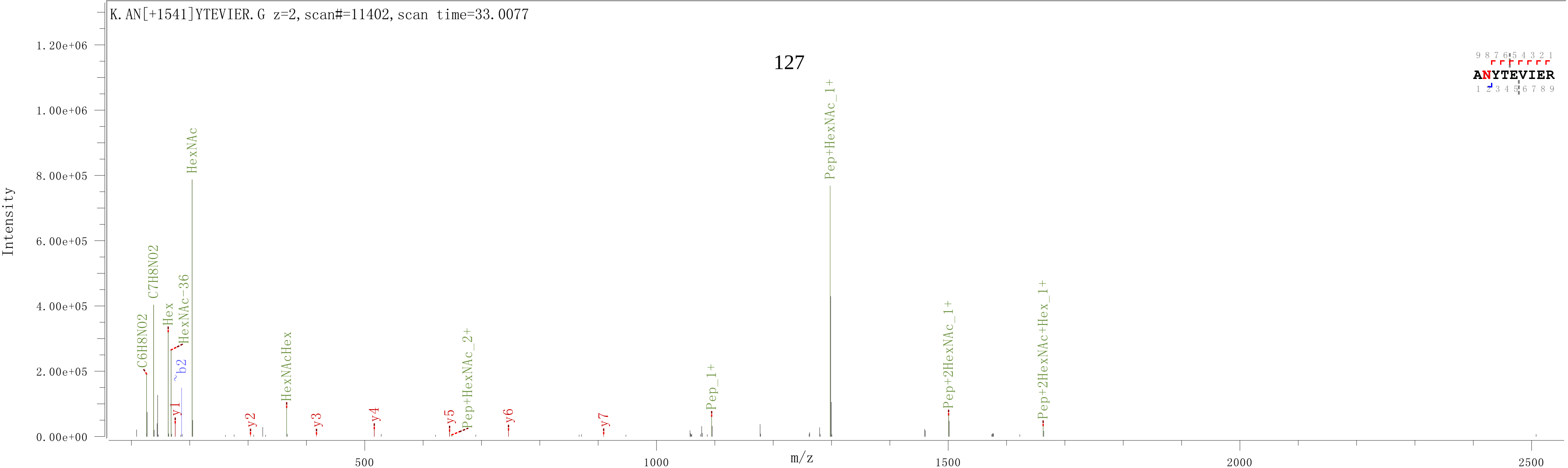
K.AN[+1541]YTEVIER.G z=2,scan#=11402,scan time=33.0077
127
ANYTEVIER
Intensity
m/z
C6H8NO2
C7H8NO2
Hex
HexNAc-36
HexNAc
~b2
y1
y2
HexNAcHex
y3
y4
y5
Pep+HexNAc_2+
y6
y7
Pep_1+
Pep+HexNAc_1+
Pep+2HexNAc_1+
Pep+2HexNAc+Hex_1+

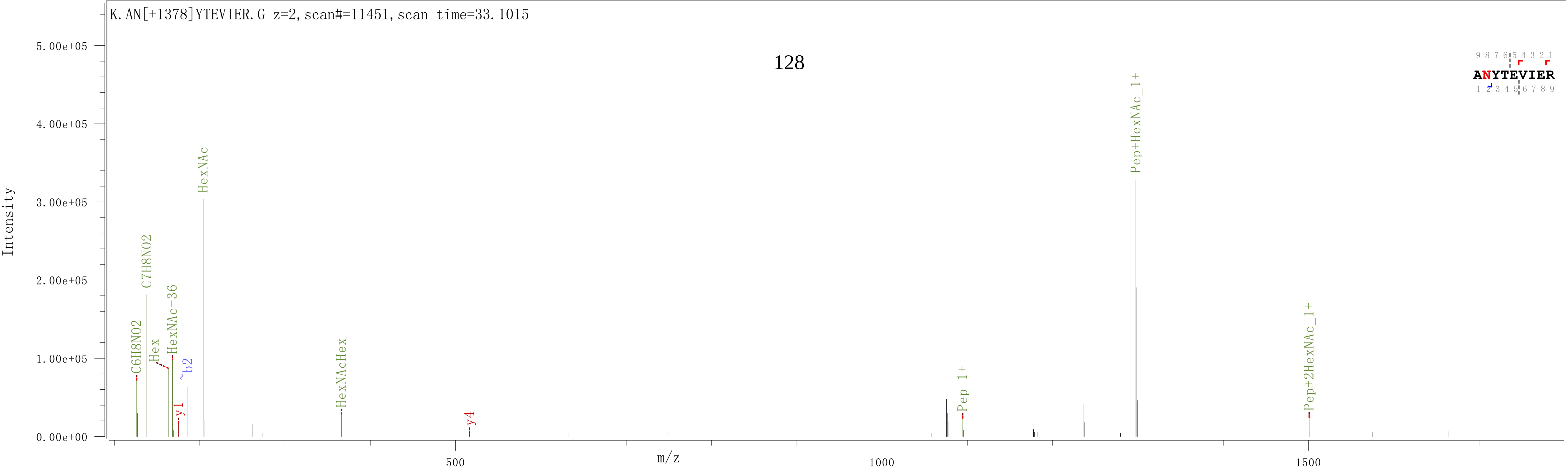
K.AN[+1378]YTEVIER.G z=2,scan#=11451,scan time=33.1015
Intensity
m/z
5.00e+05
4.00e+05
3.00e+05
2.00e+05
1.00e+05
0.00e+00
500
1000
1500
ANYTEVIER
9 8 7 6 5 4 3 2 1
1 2 3 4 5 6 7 8 9
C6H8NO2
C7H8NO2
Hex
HexNAc-36
y1
~b2
HexNAc
HexNAcHex
y4
Pep_1+
Pep+HexNAc_1+
Pep+2HexNAc_1+

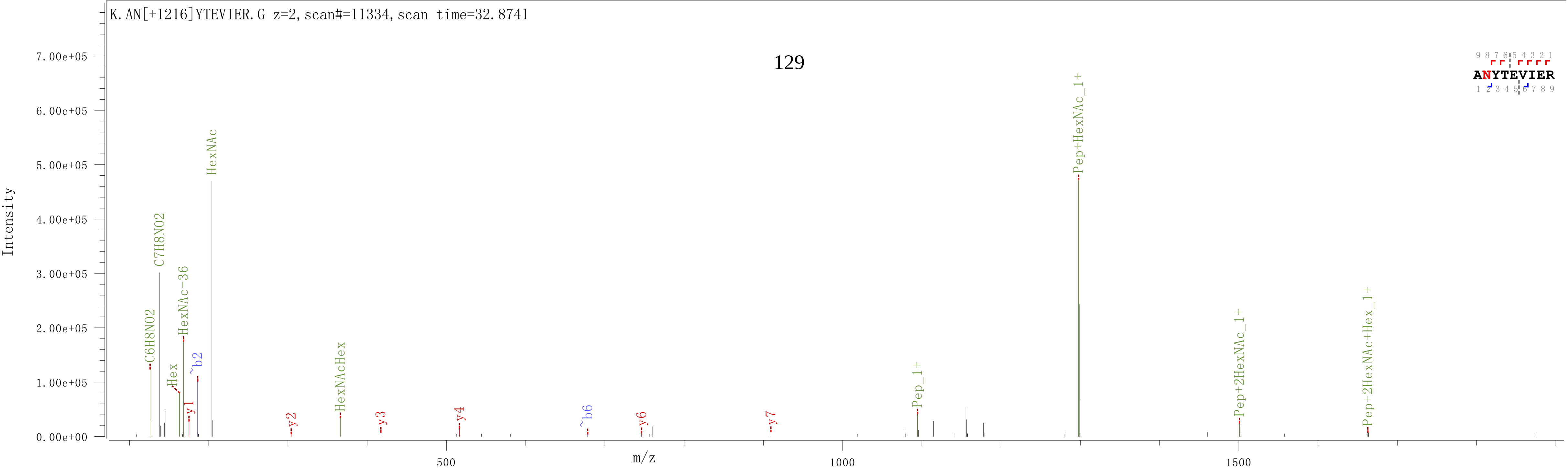
K.AN[+1216]YTEVIER.G z=2,scan#=11334,scan time=32.8741
129
Intensity
m/z
7.00e+05
6.00e+05
5.00e+05
4.00e+05
3.00e+05
2.00e+05
1.00e+05
0.00e+00
500
1000
1500
C6H8NO2
C7H8NO2
Hex
HexNAc-36
y1
~b2
HexNAc
y2
HexNAcHex
y3
y4
~b6
y6
y7
Pep_1+
Pep+HexNAc_1+
Pep+2HexNAc_1+
Pep+2HexNAc+Hex_1+
ANYTEVIER
9 8 7 6 5 4 3 2 1
1 2 3 4 5 6 7 8 9

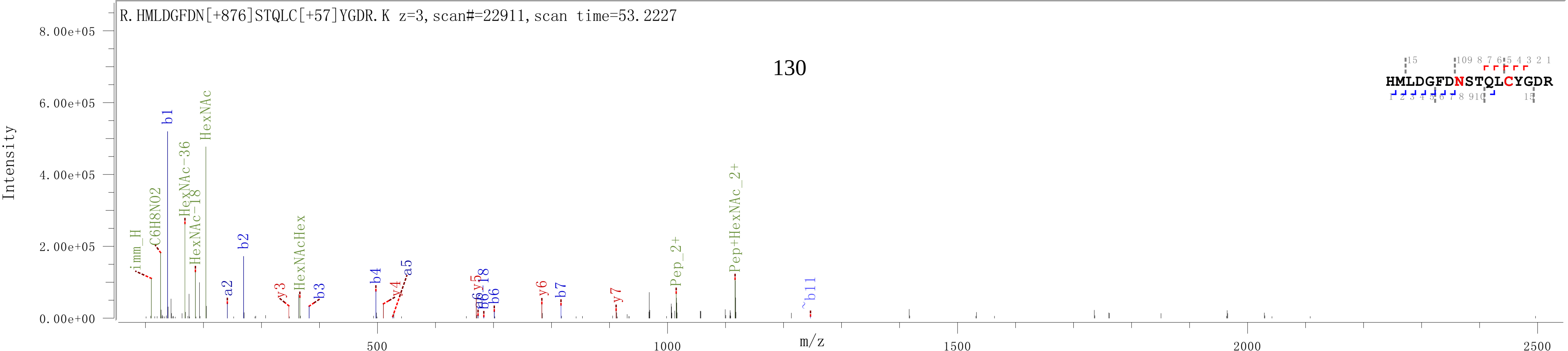
R.HMLDGFDN[+876]STQLC[+57]YGDR.K z=3,scan#=22911,scan time=53.2227
HMLDGFDNSTQLCYGDR
Intensity
m/z
imm_H
C6H8NO2
b1
HexNAc-36
HexNAc-18
HexNAc
a2
b2
y3
HexNAcHex
b3
b4
y4
a5
a6
y5
b5
y8
b6
y6
b7
y7
Pep_2+
Pep+HexNAc_2+
~b11

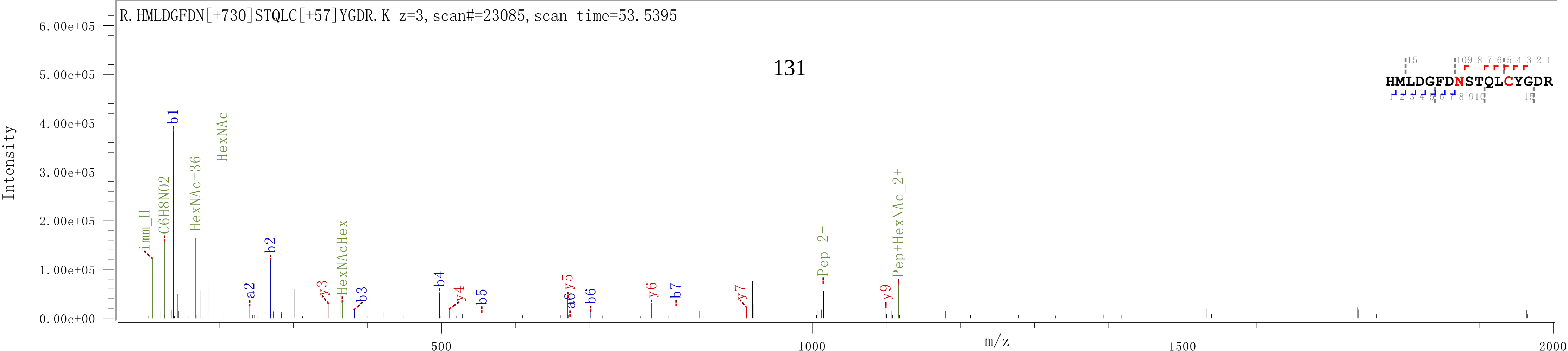
R.HMLDGFDN[+730]STQLC[+57]YGDR.K z=3,scan#=23085,scan time=53.5395
131
HMLDGFDNSTQLCYGDR
Intensity
m/z
6.00e+05
5.00e+05
4.00e+05
3.00e+05
2.00e+05
1.00e+05
0.00e+00
500
1000
1500
2000
imm_H
C6H8NO2
b1
HexNAc-36
HexNAc
a2
b2
y3
HexNAcHex
b3
b4
y4
b5
a6
y5
b6
y6
b7
y7
Pep_2+
y9
Pep+HexNAc_2+

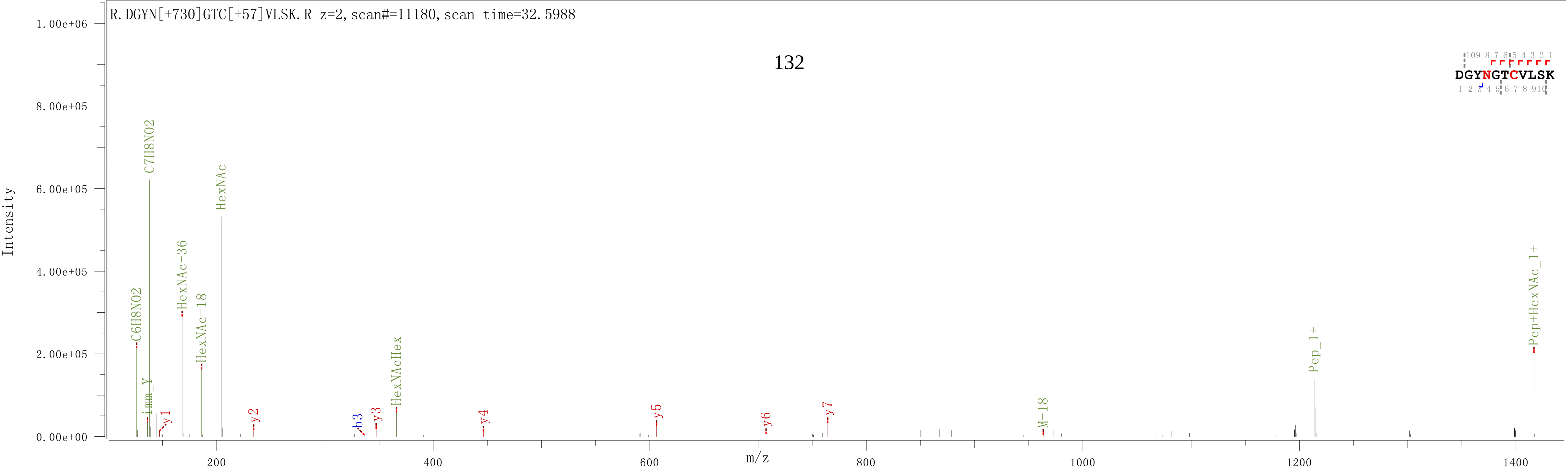

R.DGYN[+730]GTC[+57]VLSK.R z=2,scan#=11180,scan time=32.5988
132
Intensity
m/z
DGYNGTCVLSK
C7H8NO2
HexNAc
HexNAc-36
C6H8NO2
HexNAc-18
imm_Y
y1
y2
b3
y3
HexNAcHex
y4
y5
y6
y7
M-18
Pep_1+
Pep+HexNAc_1+

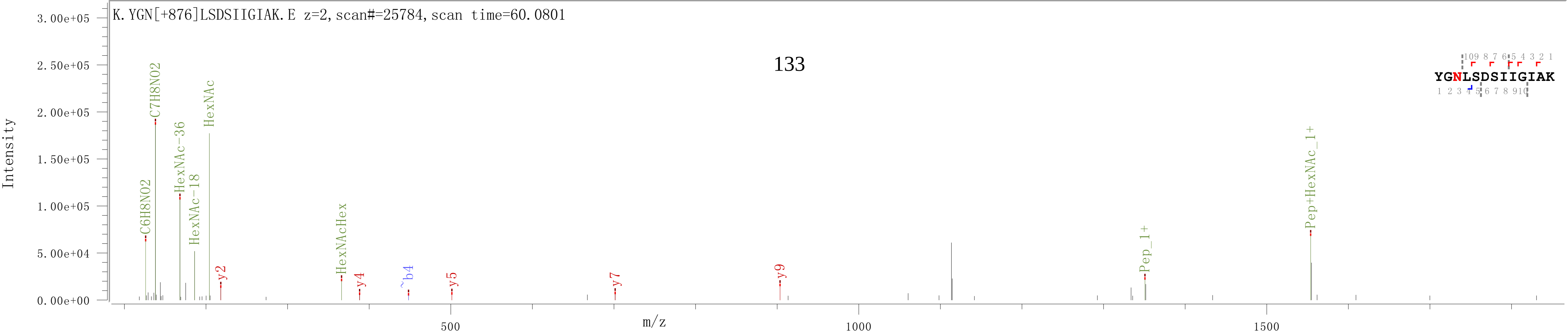

K.YGN[+876]LSDSIIGIAK.E z=2,scan#=25784,scan time=60.0801
133
YGNLSDSIIGIAK
Intensity
m/z
C6H8NO2
C7H8NO2
HexNAc-36
HexNAc-18
HexNAc
y2
HexNAcHex
y4
~b4
y5
y7
y9
Pep_1+
Pep+HexNAc_1+

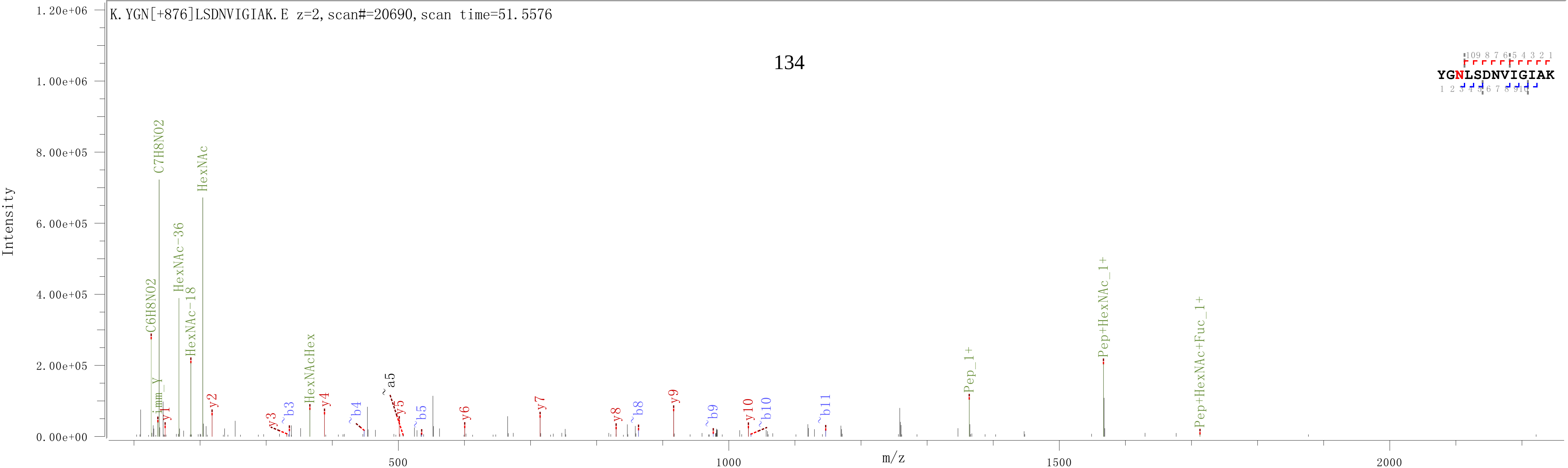

K.YGN[+876]LSDNVIGIAK.E z=2, scan#=20690, scan time=51.5576
134
YGNLSDNVIGIAK
Intensity
m/z
C7H8NO2
HexNAc
HexNAc-36
HexNAc-18
C6H8NO2
HexNAcHex
Pep+HexNAc_1+
Pep+HexNAc+Fuc_1+
Pep_1+

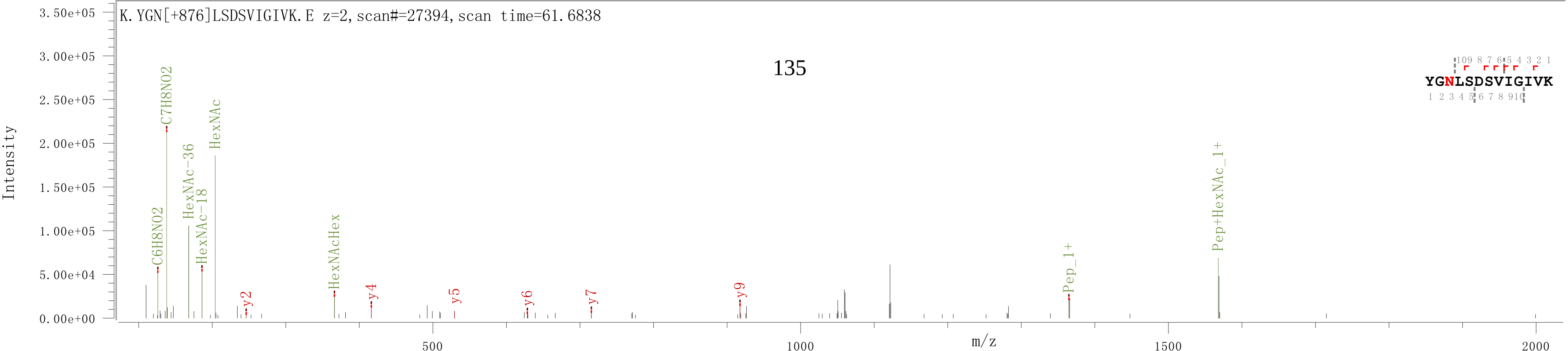

K.YGN[+876]LSDSVIGIVK.E z=2,scan#=27394,scan time=61.6838
135
YGNLSDSVIGIVK
C7H8NO2
HexNAc
HexNAc-36
HexNAc-18
C6H8NO2
HexNAcHex
y2
y4
y5
y6
y7
y9
Pep_1+
Pep+HexNAc_1+
Intensity
m/z

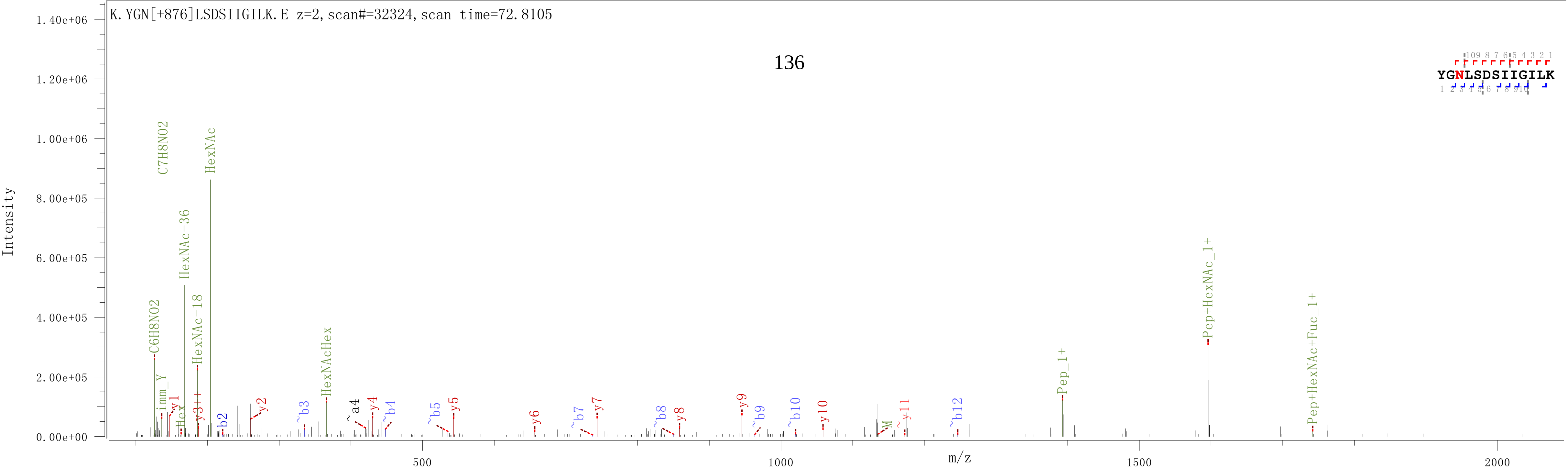
K.YGN[+876]LSDSIIGILK.E z=2,scan#=32324,scan time=72.8105
YGNLSDSIIGILK
Intensity
m/z
C7H8NO2
HexNAc
HexNAc-36
HexNAc-18
C6H8NO2
HexNAcHex
Pep+HexNAc_1+
Pep+HexNAc+Fuc_1+
Pep_1+
y1
y2
y3++
y4
y5
y6
y7
y8
y9
y10
~y11
b2
~b3
~b4
~b5
~b7
~b8
~b9
~b10
~b12
~a4
Hex
imm_Y
M
1.40e+06
1.20e+06
1.00e+06
8.00e+05
6.00e+05
4.00e+05
2.00e+05
0.00e+00
500
1000
1500
2000

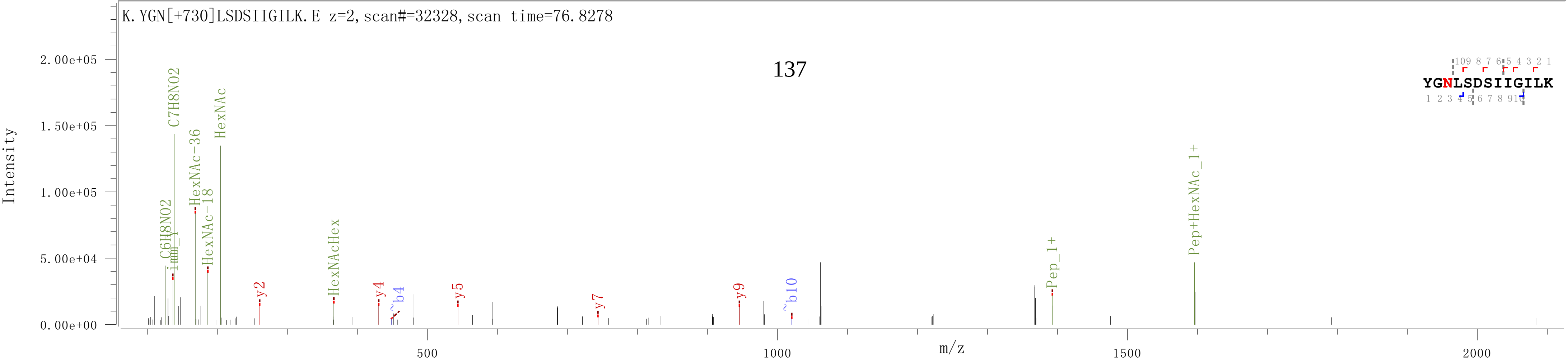

K.YGN[+730]LSDSIIGILK.E z=2,scan#=32328,scan time=76.8278
Intensity
m/z
C6H8NO2
C7H8NO2
imm_Y
HexNAc-36
HexNAc-18
HexNAc
y2
HexNAcHex
y4
~b4
y5
y7
y9
~b10
Pep_1+
Pep+HexNAc_1+
YGNLSDSIIGILK

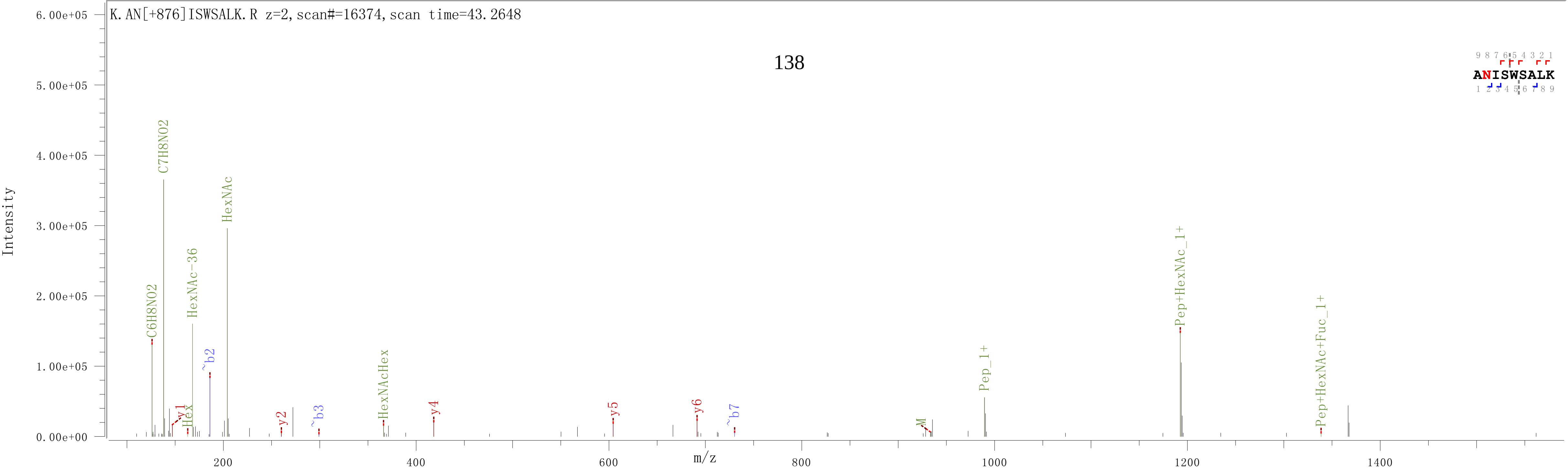

K.AN[+876]ISWSALK.R z=2,scan#=16374,scan time=43.2648
138
ANISWSALK
C6H8NO2
C7H8NO2
y1
Hex
HexNAc-36
~b2
HexNAc
y2
~b3
HexNAcHex
y4
y5
y6
~b7
M
Pep_1+
Pep+HexNAc_1+
Pep+HexNAc+Fuc_1+
Intensity
m/z

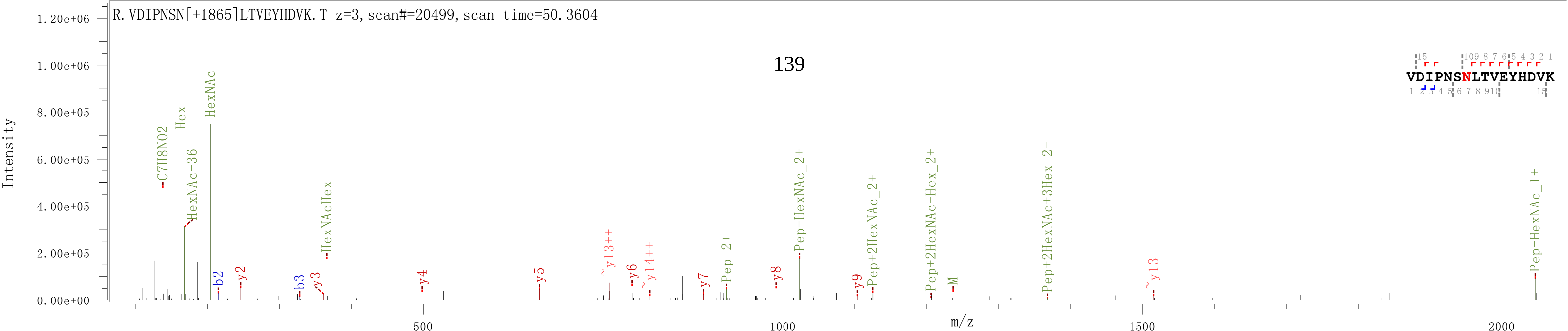

R.VDIPNSN[+1865]LTVEYHDVK.T z=3,scan#=20499,scan time=50.3604
139
VDIPNSNLTVEYHDVK
Intensity
m/z
C7H8NO2
Hex
HexNAc-36
HexNAc
b2
y2
b3
y3
HexNAcHex
y4
y5
y13++
y6
y14++
y7
Pep_2+
y8
Pep+HexNAc_2+
y9
Pep+2HexNAc_2+
Pep+2HexNAc+Hex_2+
M
Pep+2HexNAc+3Hex_2+
y13
Pep+HexNAc_1+

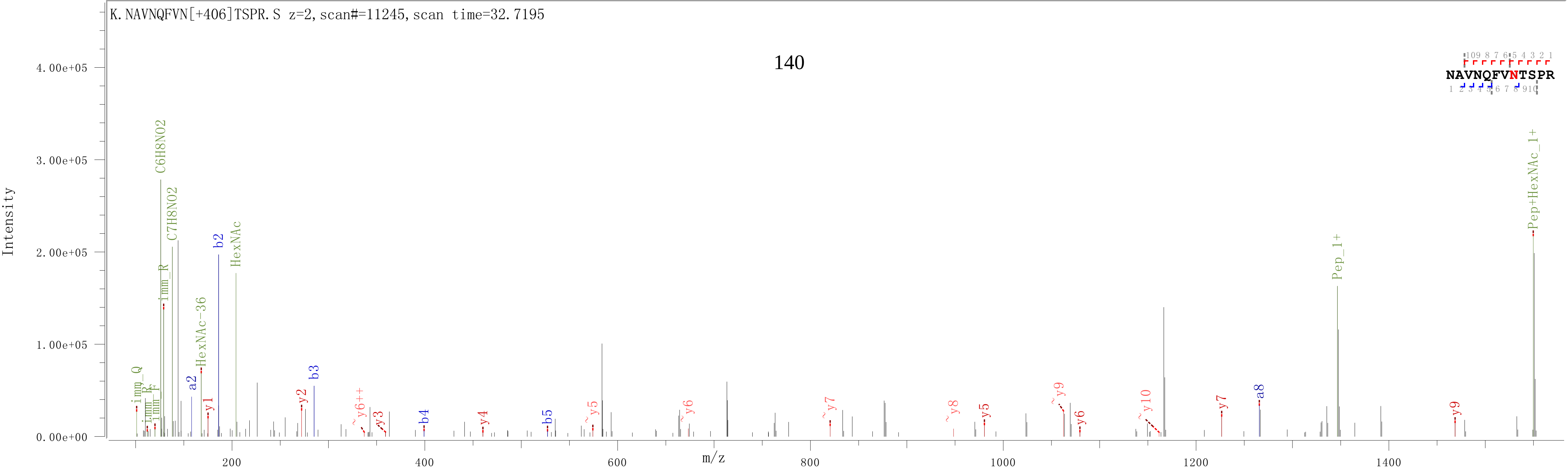

K.NAVNQFVN[+406]TSPR.S z=2,scan#=11245,scan time=32.7195
140
NAVNQFVNTSPR
Intensity
m/z
4.00e+05
3.00e+05
2.00e+05
1.00e+05
0.00e+00
200
400
600
800
1000
1200
1400
C6H8NO2
C7H8NO2
imm_R
HexNAc
HexNAc-36
b2
a2
y1
imm_Q
b3
y2
y3
b4
y4
b5
y5
y6
y7
y8
y9
y10
a8
Pep_1+
Pep+HexNAc_1+

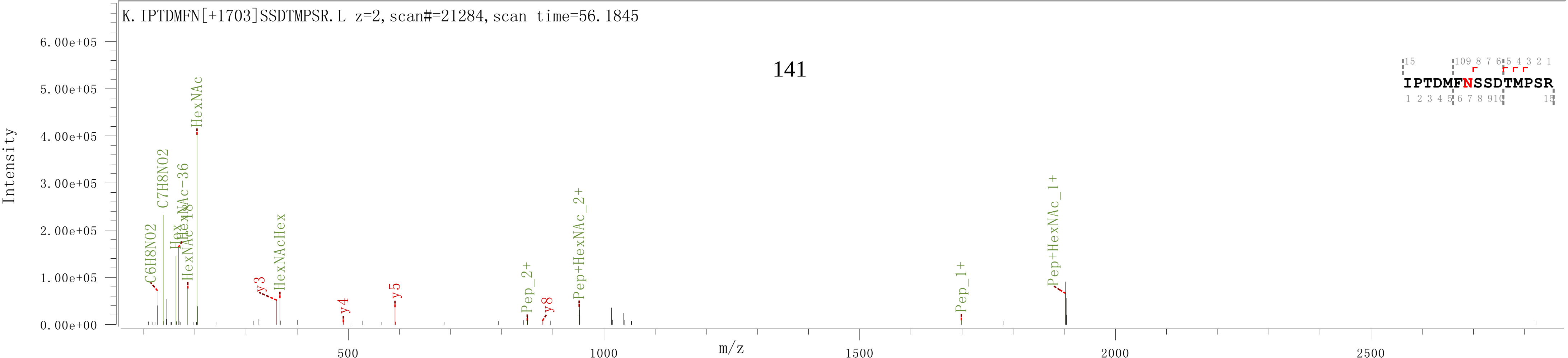

K.IPTDMFN[+1703]SSDTMPSR.L z=2,scan#=21284,scan time=56.1845
141
IPTDMFNSSDTMPSR
Intensity
m/z
C6H8NO2
C7H8NO2
Hex
HexNAc-18
HexNAc-36
HexNAc
y3
HexNAcHex
y4
y5
Pep_2+
y8
Pep+HexNAc_2+
Pep_1+
Pep+HexNAc_1+

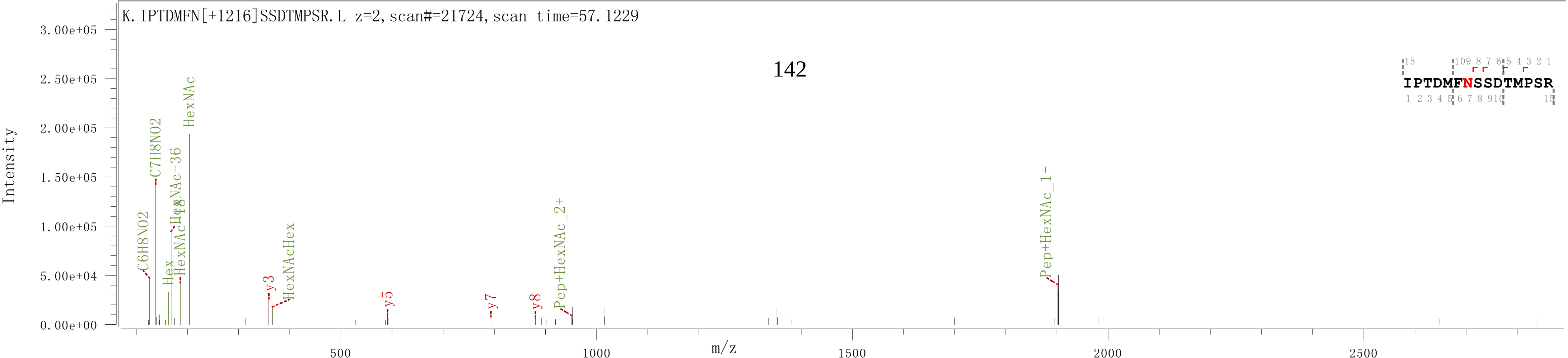

K.IPTDMFN[+1216]SSDTMPSR.L z=2,scan#=21724,scan time=57.1229
142
IPTDMFNSSDTMPSR
C6H8NO2
C7H8NO2
Hex
HexNAc-36
HexNAc-18
HexNAc
y3
HexNAcHex
y5
y7
y8
Pep+HexNAc_2+
Pep+HexNAc_1+
Intensity
m/z

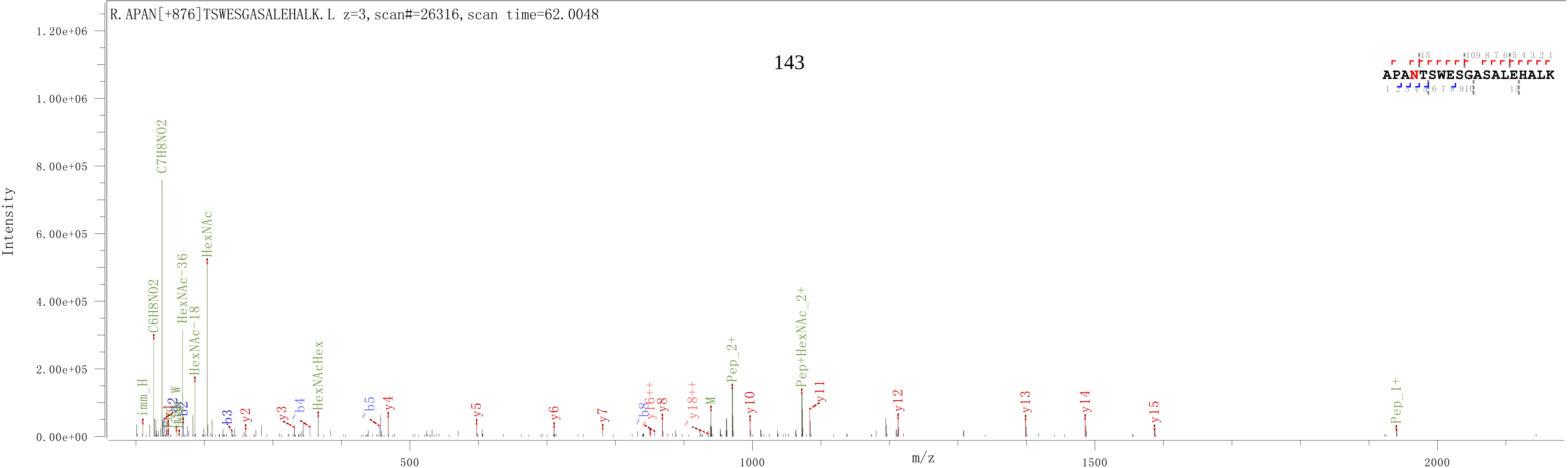
R.APAN[+876]TSWESGASALEHALK.L z=3,scan#=26316,scan time=62.0048
143
APANTSWESGASALEHALK
Intensity
m/z
C7H8NO2
HexNAc
C6H8NO2
HexNAc-36
HexNAc-18
HexNAcHex
Pep_2+
Pep+HexNAc_2+
Pep_1+
imm_H
W
M
b2
b3
y2
y3
~b4
~b5
y4
y5
y6
y7
~b8
y16++
y8
~y18++
y10
y11
y12
y13
y14
y15

R.APAN[+730]TSWESGASALEHALK.L z=3, scan#=26418, scan time=62.1848

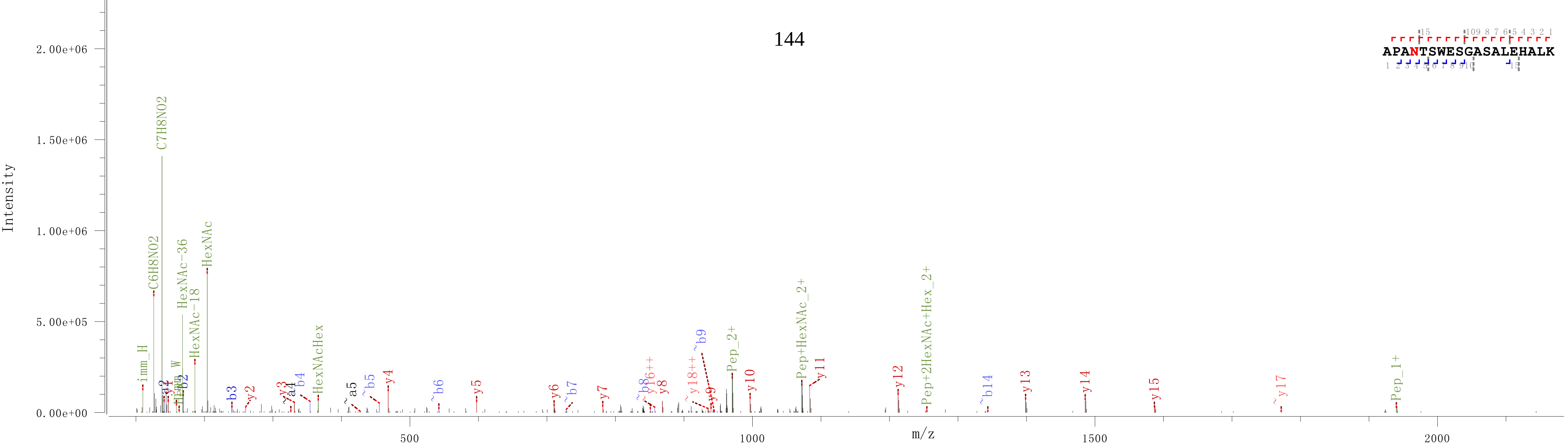

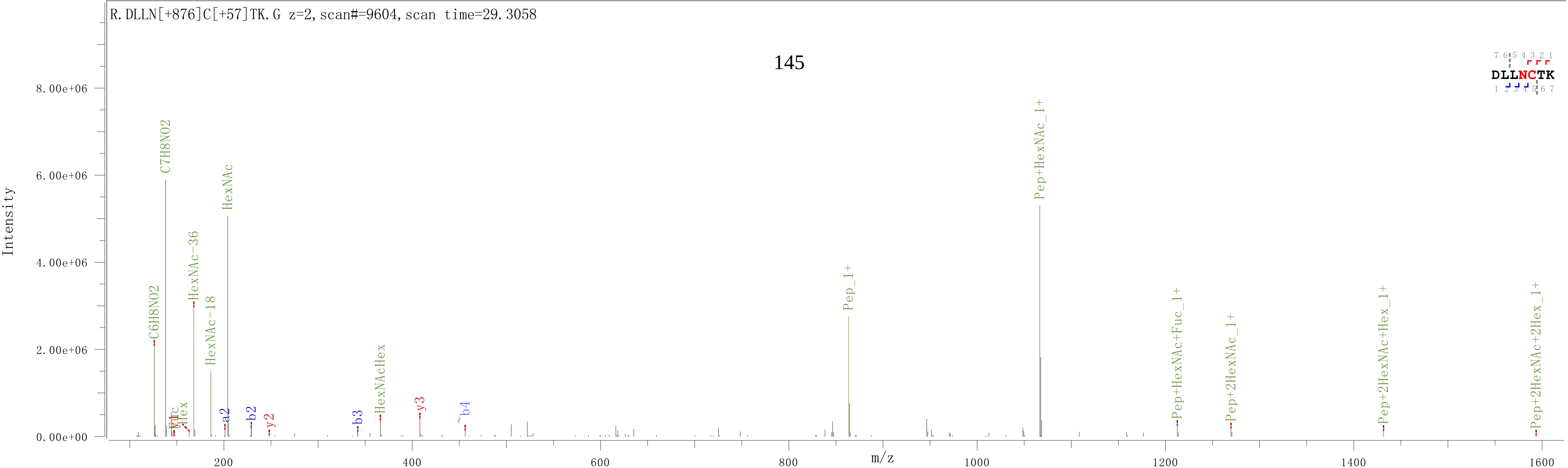
R.DLLN[+876]C[+57]TK.G z=2,scan#=9604,scan time=29.3058
145
DLLNCTK
Intensity
m/z
C6H8NO2
C7H8NO2
HexNAc-36
HexNAc-18
HexNAc
a2
b2
y2
b3
HexNAcHex
y3
~b4
Pep_1+
Pep+HexNAc_1+
Pep+HexNAc+Fuc_1+
Pep+2HexNAc_1+
Pep+2HexNAc+Hex_1+
Pep+2HexNAc+2Hex_1+

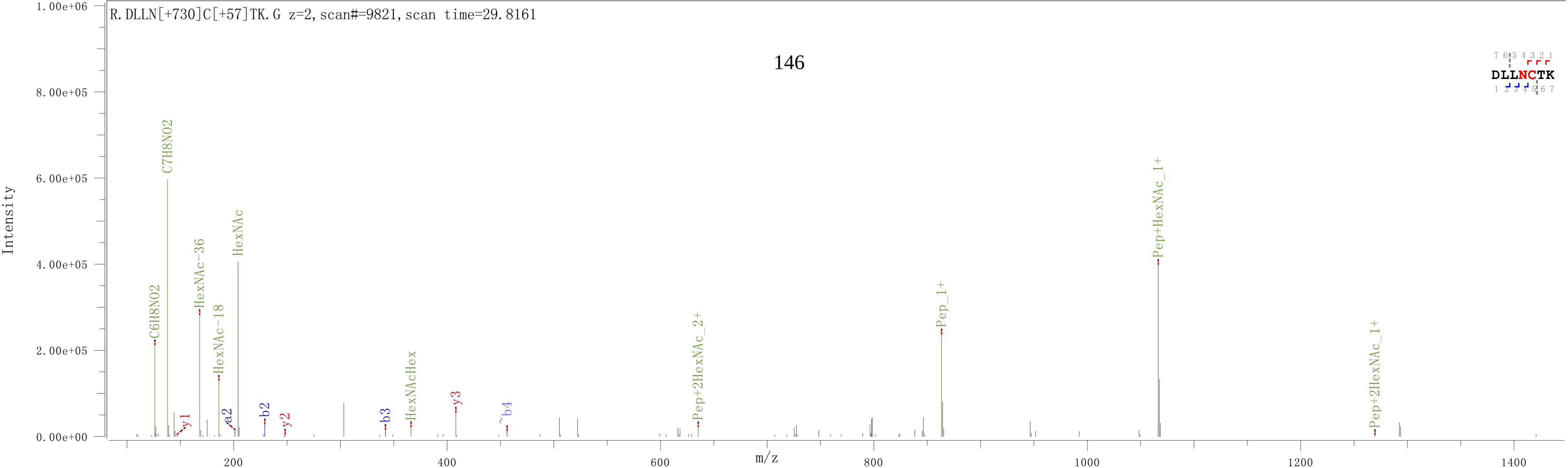

R.DLLN[+730]C[+57]TK.G z=2,scan#=9821,scan time=29.8161
146
DLLNCTK
Intensity
m/z
C6H8NO2
C7H8NO2
HexNAc-36
HexNAc-18
HexNAc
y1
a2
b2
y2
b3
HexNAcHex
y3
^b4
Pep+2HexNAc_2+
Pep_1+
Pep+HexNAc_1+
Pep+2HexNAc_1+

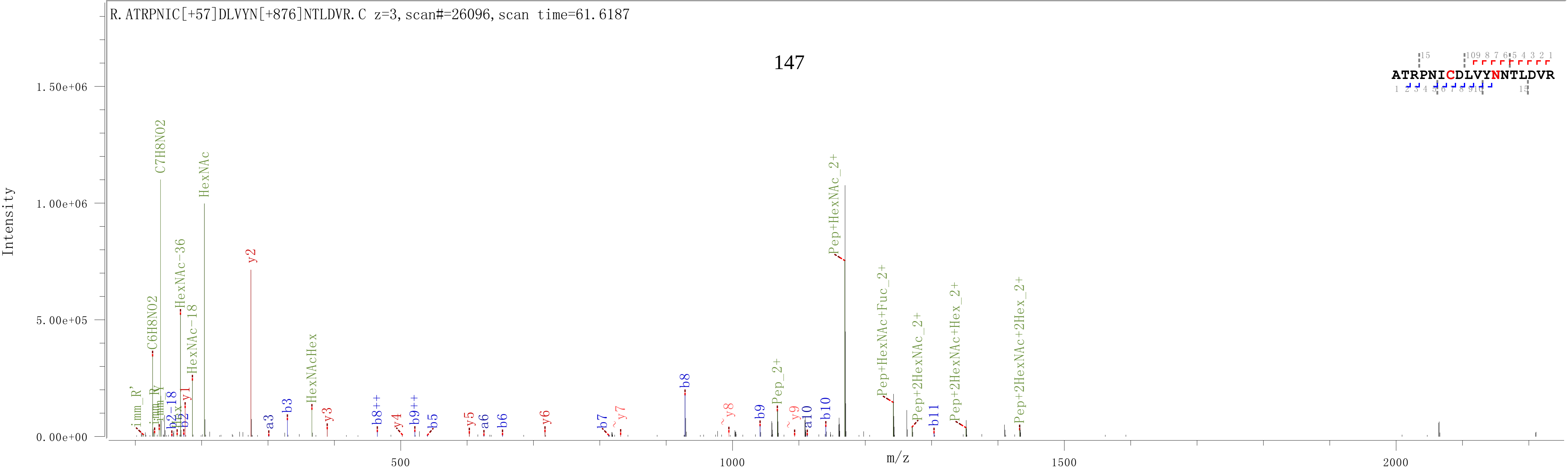
R.ATRPNIC[+57]DLVYN[+876]NTLDVR.C z=3,scan#=26096,scan time=61.6187
ATRPNICDLVYNNTLDVR
Intensity
m/z
0.00e+00
5.00e+05
1.00e+06
1.50e+06
500
1000
1500
2000
C7H8NO2
HexNAc
HexNAc-36
HexNAc-18
C6H8NO2
y2
y1
HexNAcHex
Pep+HexNAc_2+
Pep+HexNAc+Fuc_2+
Pep+2HexNAc_2+
Pep+2HexNAc+Hex_2+
Pep+2HexNAc+2Hex_2+
Pep_2+
b8
b9
b10
b11
a10
b3
a3
y3
b8++
y4
b9++
b5
y5
a6
b6
y6
b7
~y7
~y8
~y9
imm_R'

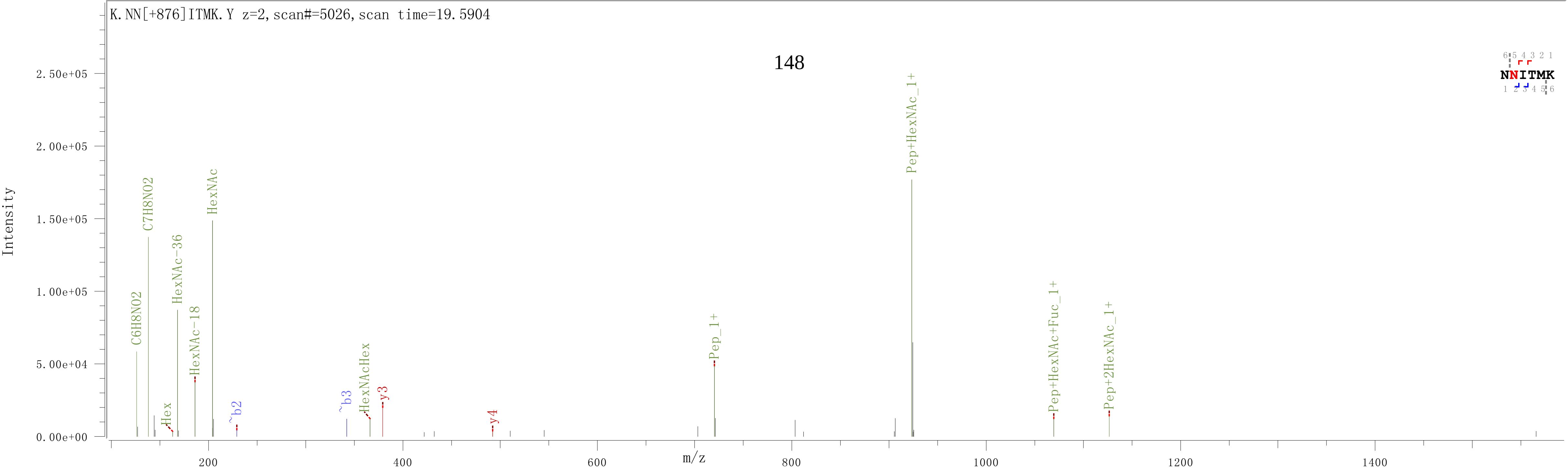

K.NN[+876]ITMK.Y z=2,scan#=5026,scan time=19.5904
148
Intensity
m/z
C6H8NO2
C7H8NO2
Hex
HexNAc-36
HexNAc-18
HexNAc
~b2
~b3
HexNAcHex
y3
y4
Pep_1+
Pep+HexNAc_1+
Pep+HexNAc+Fuc_1+
Pep+2HexNAc_1+
NNITMK

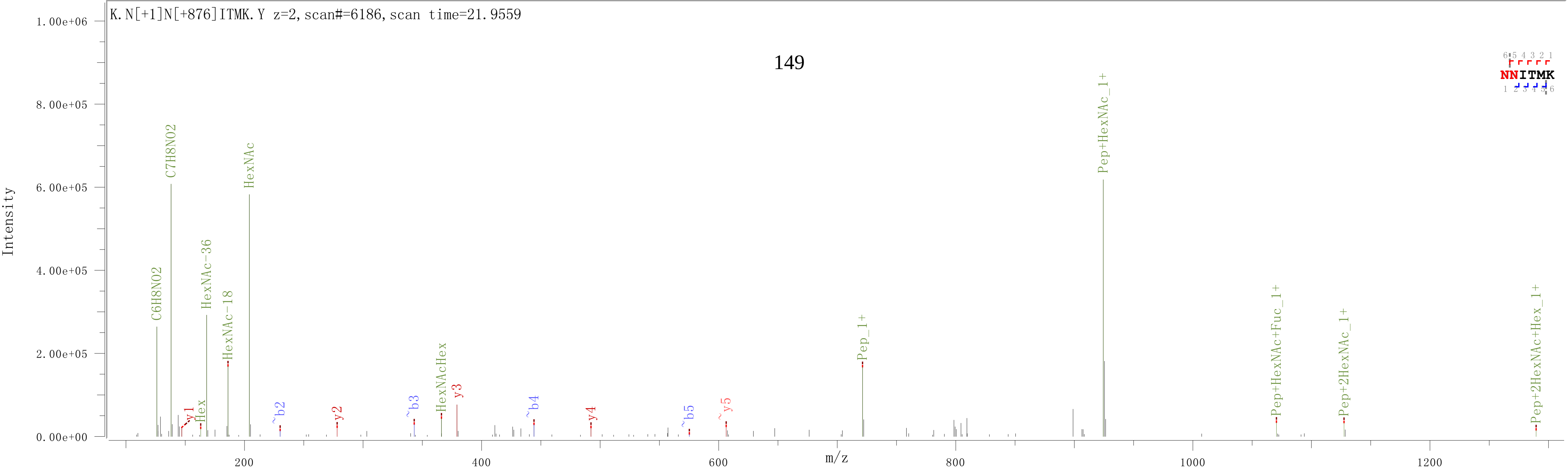

K.N[+1]N[+876]ITMK.Y z=2,scan#=6186,scan time=21.9559
149
NNITMK
Intensity
m/z
C6H8NO2
C7H8NO2
y1
Hex
HexNAc-36
HexNAc-18
HexNAc
~b2
y2
~b3
HexNAcHex
y3
~b4
y4
~b5
~y5
Pep_1+
Pep+HexNAc_1+
Pep+HexNAc+Fuc_1+
Pep+2HexNAc_1+
Pep+2HexNAc+Hex_1+

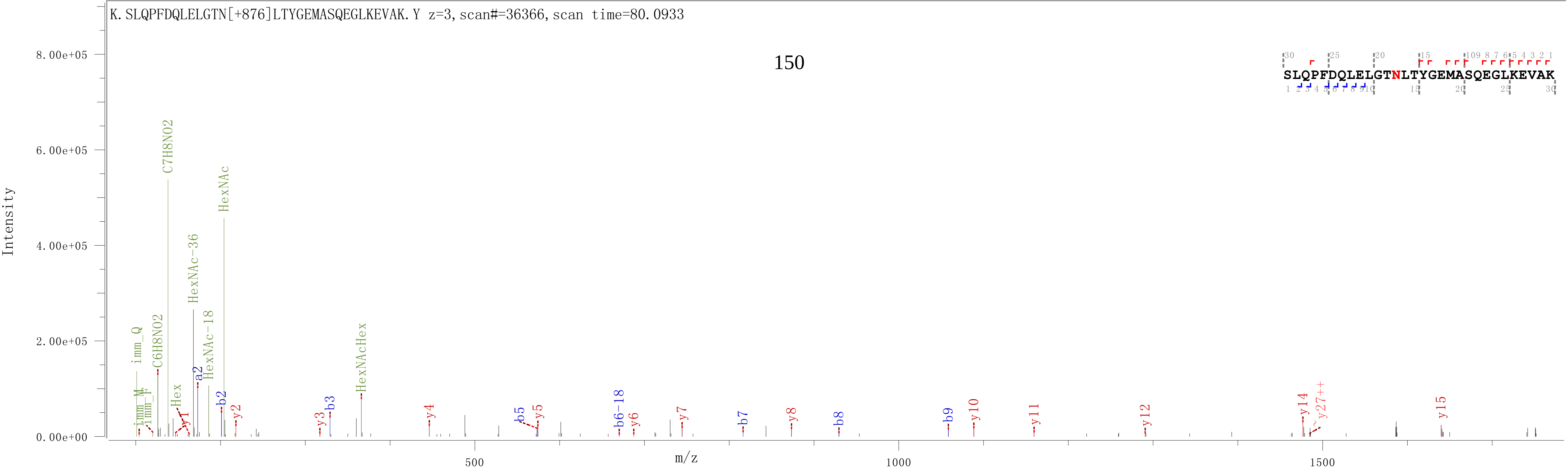
K.SLQPFDQLELGTN[+876]LTYGEMASQEGLKEVAK.Y z=3,scan#=36366,scan time=80.0933
SLQPFDQLELGTNLTYGEMASQEGLKEVAK
Intensity
m/z
8.00e+05
6.00e+05
4.00e+05
2.00e+05
0.00e+00
500
1000
1500
C7H8NO2
HexNAc
HexNAc-36
HexNAc-18
C6H8NO2
imm_Q
HexNAcHex
Hex
a2
b2
b3
b5
b6-18
b7
b8
b9
y2
y3
y4
y5
y6
y7
y8
y10
y11
y12
y14
y15
y27++

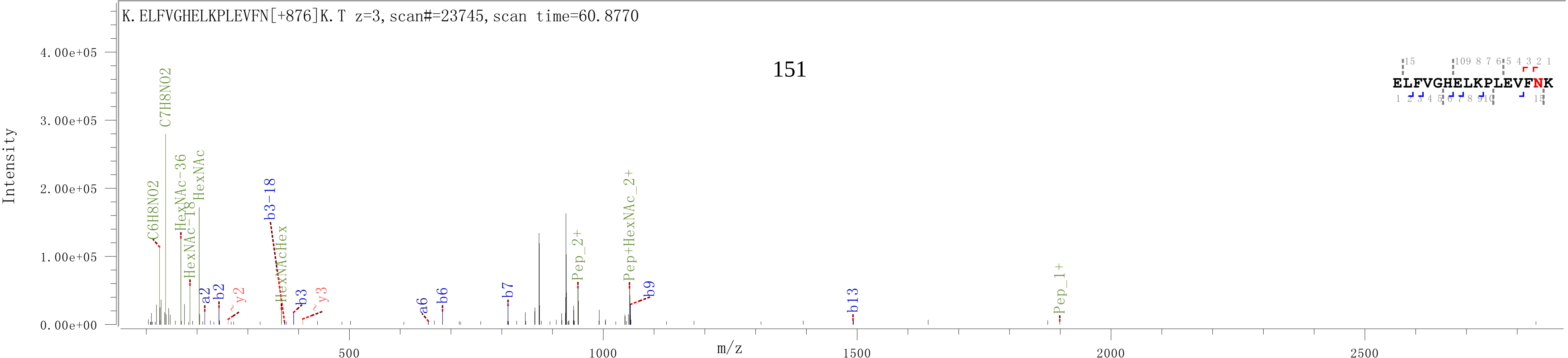
K.ELFVGHELKPLEVFN[+876]K.T z=3,scan#=23745,scan time=60.8770
151
Intensity
m/z
ELFVGHELKPLEVFNK
C7H8NO2
C6H8NO2
HexNAc-36
HexNAc-18
HexNAc
b3-18
HexNAcHex
a2
b2
y2
b3
y3
a6
b6
b7
Pep_2+
Pep+HexNAc_2+
b9
b13
Pep_1+

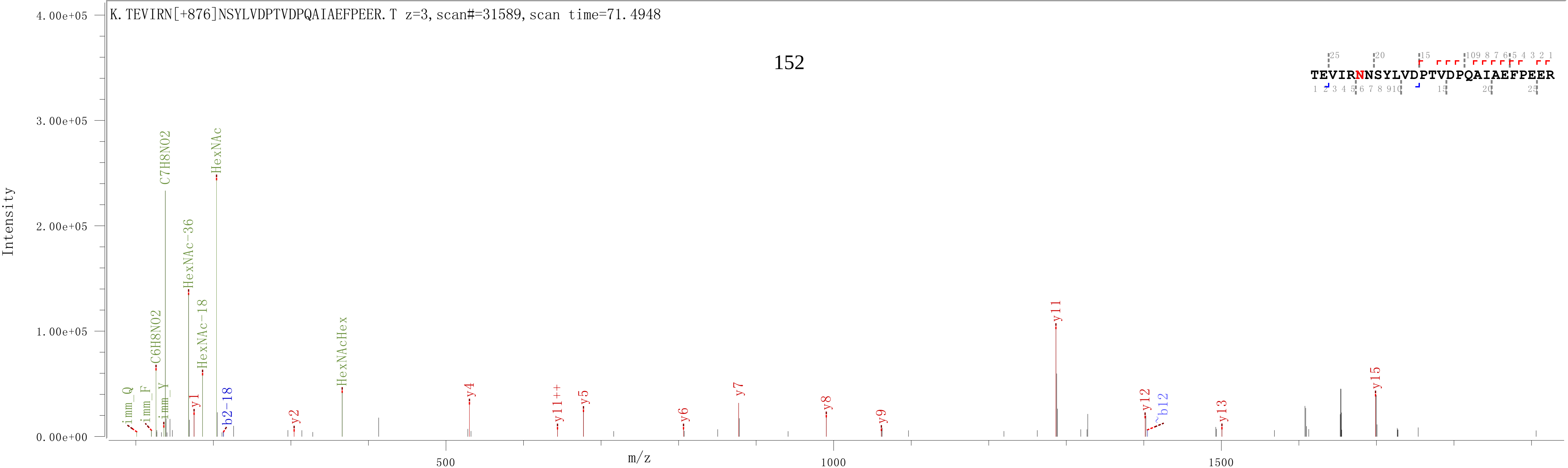
K.TEVIRN[+876]NSYLVDPTVDPQAIAEFPEER.T z=3,scan#=31589,scan time=71.4948
TEVIRNNSYLVDPTVDPQAIAEFPEER
Intensity
4.00e+05
3.00e+05
2.00e+05
1.00e+05
0.00e+00
m/z
500
1000
1500
imm_Q
imm_F
C6H8NO2
imm_Y
C7H8NO2
HexNAc-36
y1
HexNAc-18
HexNAc
b2-18
y2
HexNAcHex
y4
y11++
y5
y6
y7
y8
y9
y11
y12
^b12
y13
y15

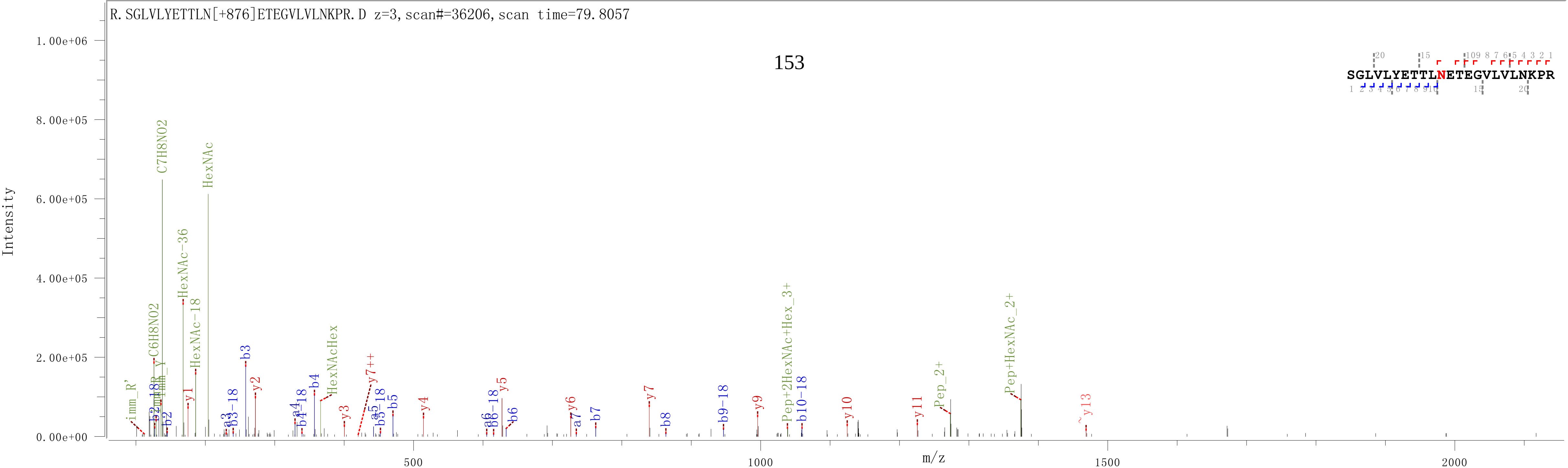
R.SGLVLYETTLN[+876]ETEGVLVLNKPR.D z=3,scan#=36206,scan time=79.8057
SGLVLYETTLNETEGVLVLNKPR
Intensity
m/z
C7H8NO2
HexNAc
HexNAc-36
HexNAc-18
C6H8NO2
HexNAcHex
Pep+2HexNAc+Hex_3+
Pep+HexNAc_2+
Pep_2+
y7++
y1
y2
y3
y4
y5
y6
y7
y9
y10
y11
y13
b2
b3
b4
b6
b7
b8
b9-18
b10-18
0.00e+00
2.00e+05
4.00e+05
6.00e+05
8.00e+05
1.00e+06
500
1000
1500
2000

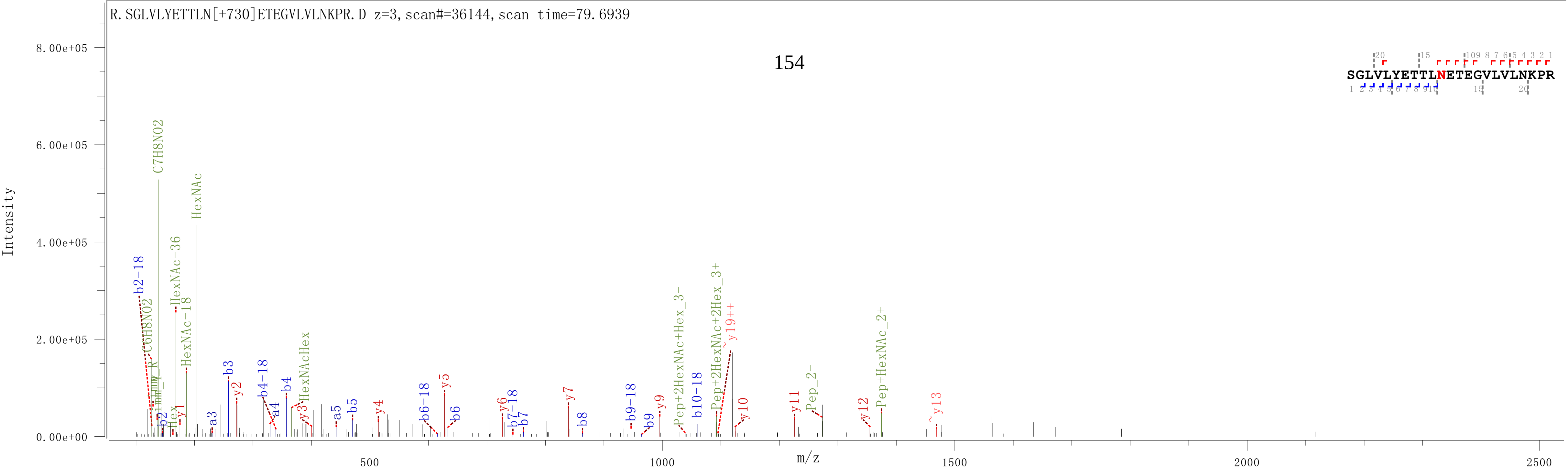
R.SGLVLYETTLN[+730]ETEGVLVLNKPR.D z=3,scan#=36144,scan time=79.6939
154
SGLVLYETTLNETEGVLVLNKPR
Intensity
m/z
8.00e+05
6.00e+05
4.00e+05
2.00e+05
0.00e+00
500
1000
1500
2000
2500
C7H8NO2
HexNAc
HexNAc-36
HexNAc-18
b2-18
C6H8NO2
HexNAcHex
Pep+2HexNAc+Hex_3+
Pep+2HexNAc+2Hex_3+
~y19++
b10-18
Pep+HexNAc_2+
Pep_2+
b3
y2
b4-18
b4
a4
y3
a5
b5
y4
b6-18
y5
b6
y6
b7-18
b7
y7
b8
b9-18
y9
b9
y10
y11
y12
~y13
y1
b2
a3
Hex

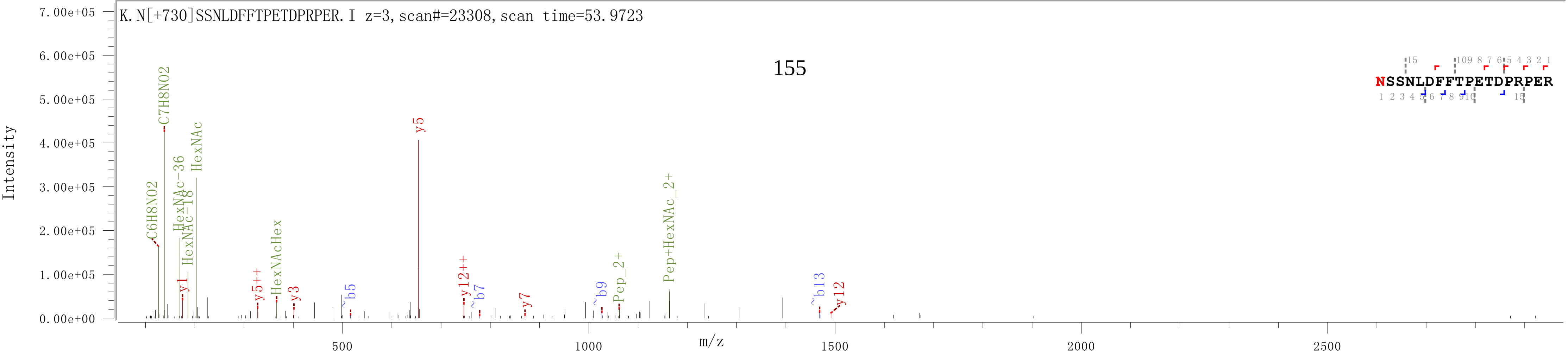

K.N[+730]SSNLDFFTPETDPRPER.I z=3,scan#=23308,scan time=53.9723
155
NSSNLDFFTPETDPRPER
Intensity
m/z
C7H8NO2
C6H8NO2
HexNAc-36
HexNAc-18
HexNAc
y1
y5++
HexNAcHex
y3
~b5
y5
y12++
~b7
y7
~b9
Pep_2+
Pep+HexNAc_2+
~b13
y12

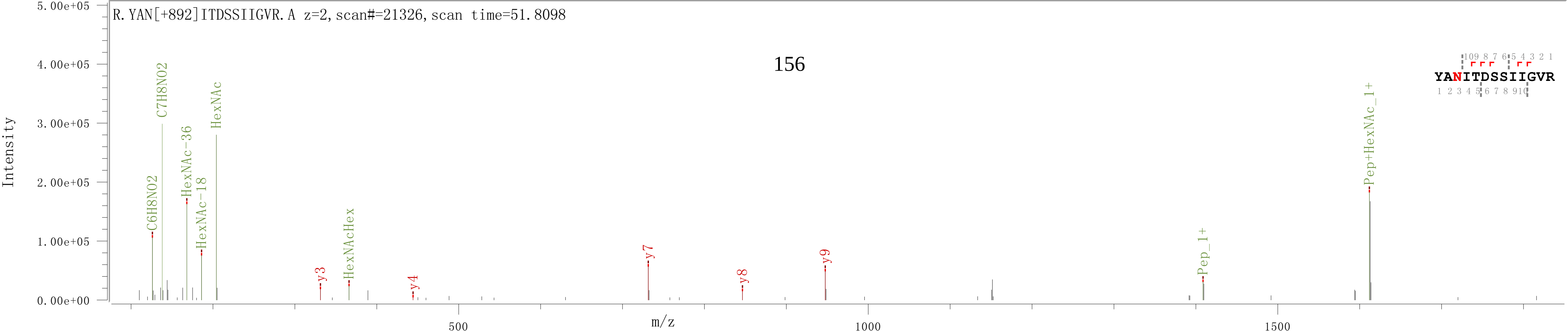
R.YAN[+892]ITDSSIIGVR.A z=2,scan#=21326,scan time=51.8098
156
Intensity
5.00e+05
4.00e+05
3.00e+05
2.00e+05
1.00e+05
0.00e+00
C6H8NO2
C7H8NO2
HexNAc-36
HexNAc-18
HexNAc
y3
HexNAcHex
y4
y7
y8
y9
Pep_1+
Pep+HexNAc_1+
YANITDSSIIGVR
m/z
500
1000
1500

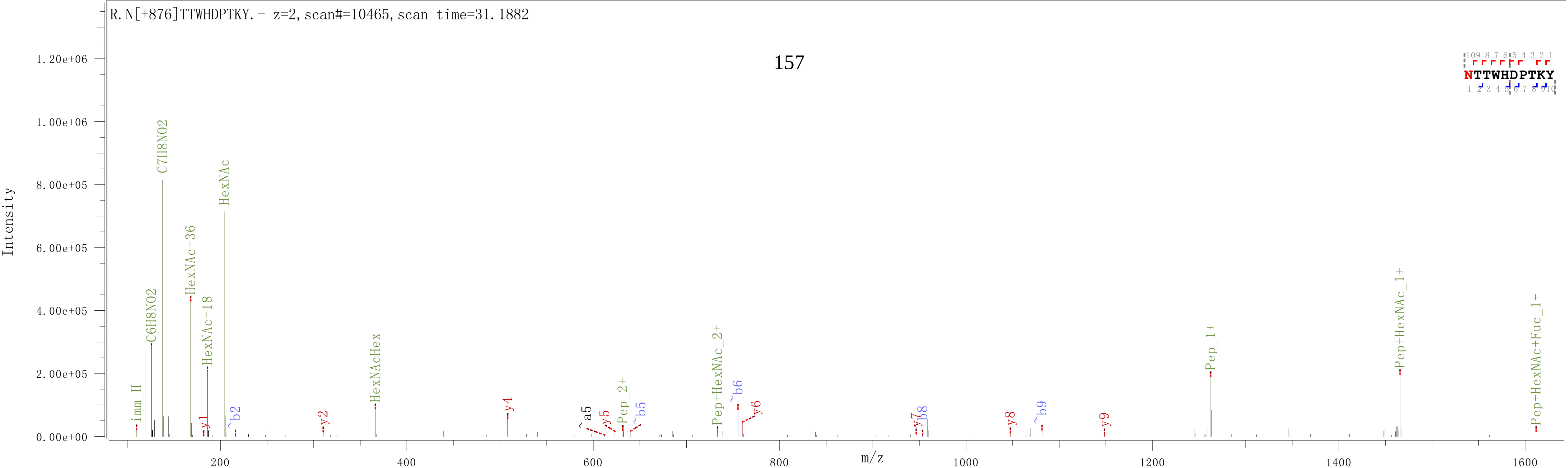
R.N[+876]TTWHDPTKY.- z=2,scan#=10465,scan time=31.1882
157
NTTWHDPTKY
Intensity
m/z
imm_H
C6H8NO2
C7H8NO2
HexNAc-36
HexNAc-18
HexNAc
y1
~b2
y2
HexNAcHex
y4
~a5
y5
Pep_2+
~b5
Pep+HexNAc_2+
~b6
y6
y7
b8
y8
~b9
y9
Pep_1+
Pep+HexNAc_1+
Pep+HexNAc+Fuc_1+

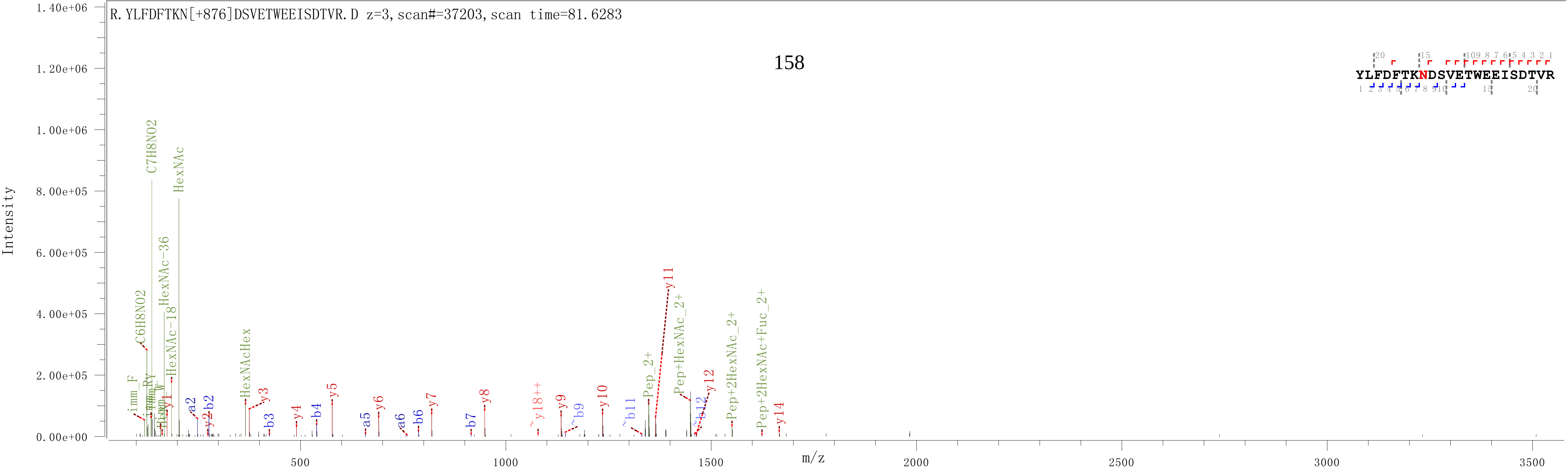
R.YLFDFTKN[+876]DSVETWEEISDTVR.D z=3,scan#=37203,scan time=81.6283
YLFDFTKNDSVETWEEISDTVR
Intensity
m/z
1.40e+06
1.20e+06
1.00e+06
8.00e+05
6.00e+05
4.00e+05
2.00e+05
0.00e+00
500
1000
1500
2000
2500
3000
3500
C7H8NO2
HexNAc
HexNAc-36
HexNAc-18
C6H8NO2
imm_F
HexNAcHex
a2
b2
y2
y1
y3
b3
y4
b4
y5
a5
y6
a6
b6
y7
b7
y8
~y18++
y9
~b9
y10
~b11
Pep_2+
y11
Pep+HexNAc_2+
~b12
y12
Pep+2HexNAc_2+
Pep+2HexNAc+Fuc_2+
y14

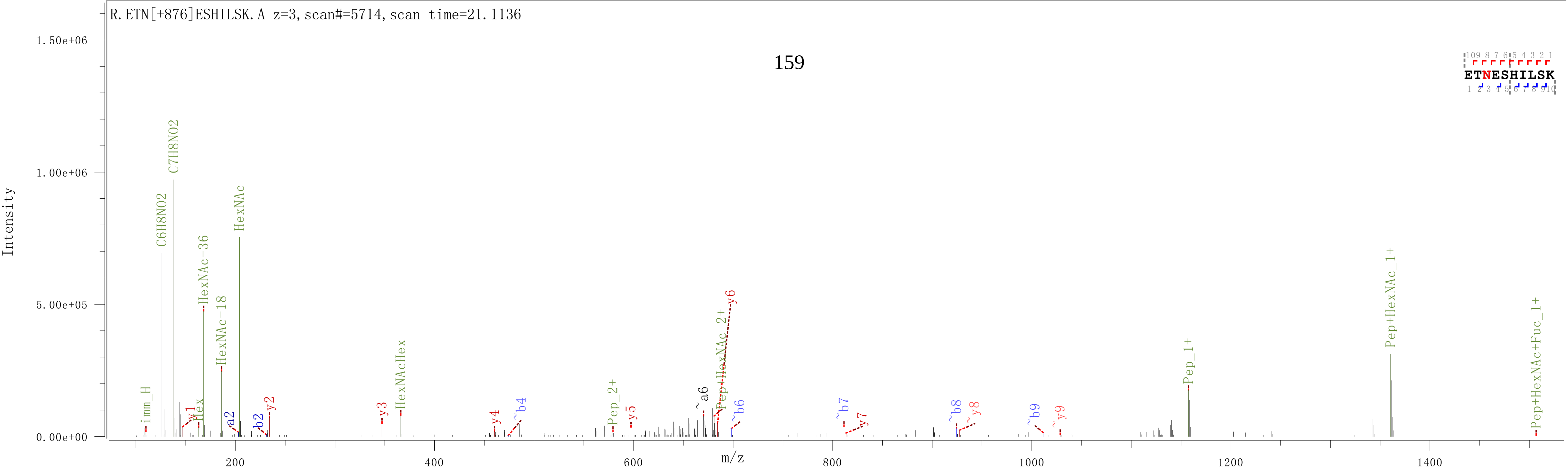
R.ETN[+876]ESHILSK.A z=3,scan#=5714,scan time=21.1136
159
ETNESHILSK
Intensity
m/z
C7H8NO2
C6H8NO2
HexNAc
HexNAc-36
HexNAc-18
HexNAcHex
imm_H
Hex
y1
a2
b2
y2
y3
y4
~b4
Pep_2+
y5
~a6
Pep+HexNAc_2+
y6
~b6
~b7
y7
~b8
~y8
~b9
~y9
Pep_1+
Pep+HexNAc_1+
Pep+HexNAc+Fuc_1+

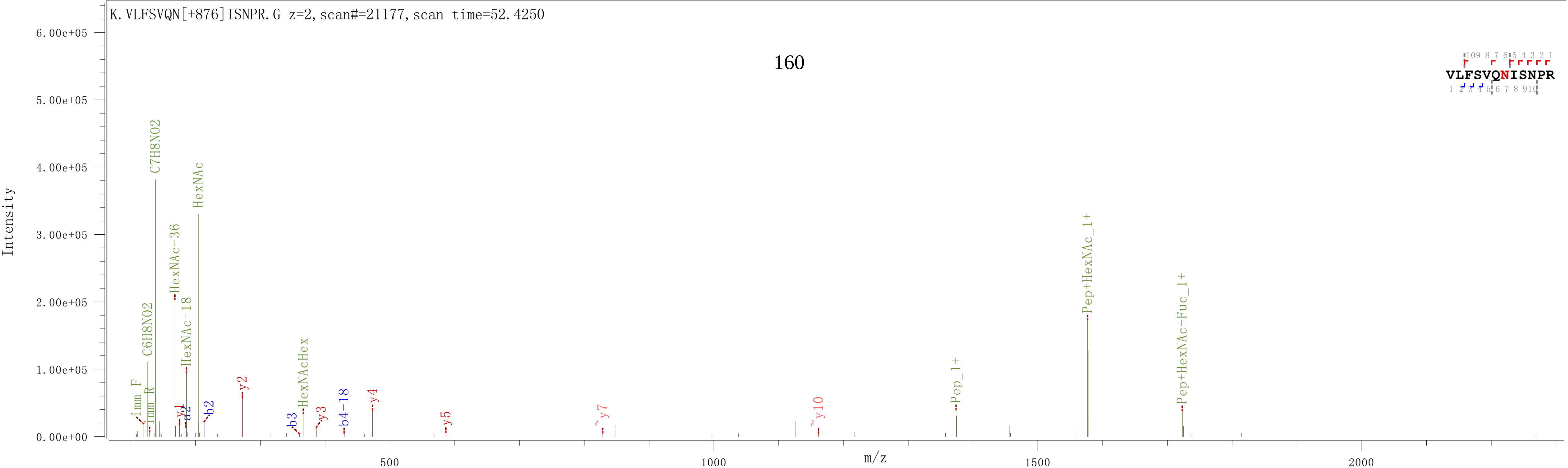

K.VLFSVQN[+876]ISNPR.G z=2,scan#=21177,scan time=52.4250
160
VLFSVQNISNPR
Intensity
m/z
C7H8NO2
HexNAc
HexNAc-36
HexNAc-18
C6H8NO2
imm_F
imm_R
y1
a2
b2
y2
b3
HexNAcHex
y3
b4-18
y4
y5
~y7
~y10
Pep_1+
Pep+HexNAc_1+
Pep+HexNAc+Fuc_1+

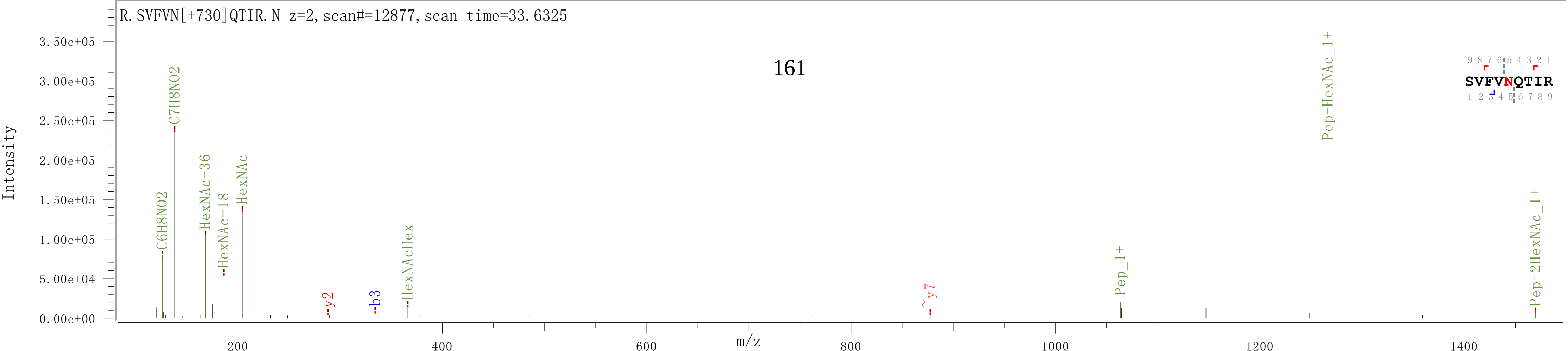
R.SVFVN[+730]QTIR.N z=2,scan#=12877,scan time=33.6325
161
Intensity
m/z
C6H8NO2
C7H8NO2
HexNAc-36
HexNAc-18
HexNAc
y2
b3
HexNAcHex
~y7
Pep_1+
Pep+HexNAc_1+
Pep+2HexNAc_1+
SVFVNQTIR

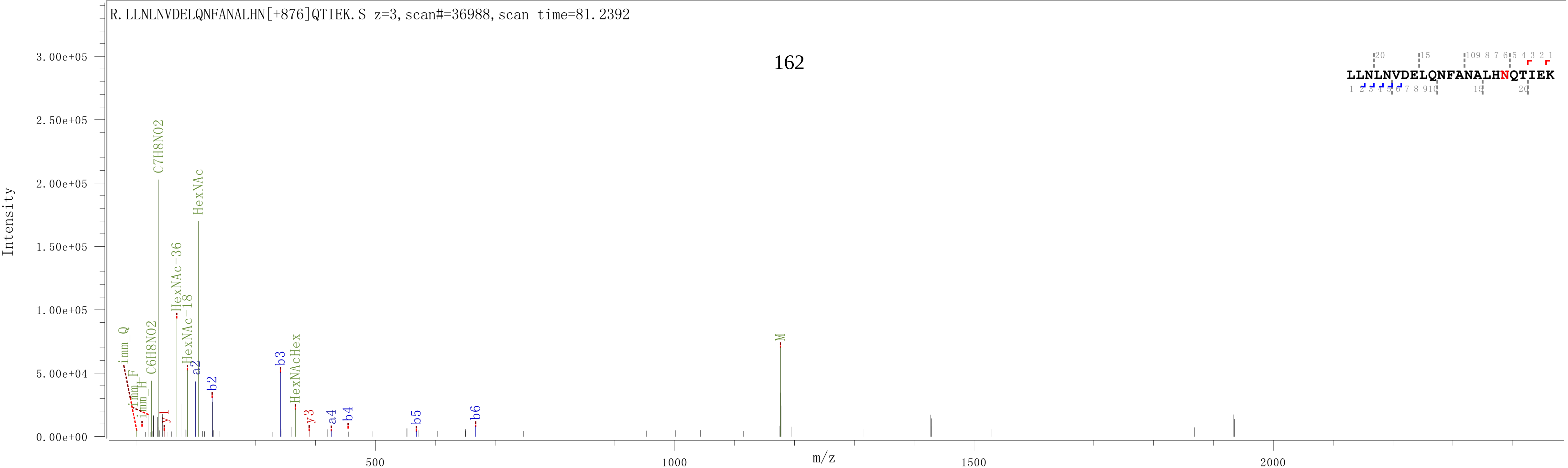

R.LLNLNVDELQNFANALHN[+876]QTIEK.S z=3,scan#=36988,scan time=81.2392
162
LLNLNVDELQNFANALHNQTIEK
Intensity
m/z
3.00e+05
2.50e+05
2.00e+05
1.50e+05
1.00e+05
5.00e+04
0.00e+00
500
1000
1500
2000
C7H8NO2
HexNAc
HexNAc-36
HexNAc-18
C6H8NO2
imm_Q
imm_F
imm_H
y1
a2
b2
b3
HexNAcHex
y3
a4
b4
b5
b6
M

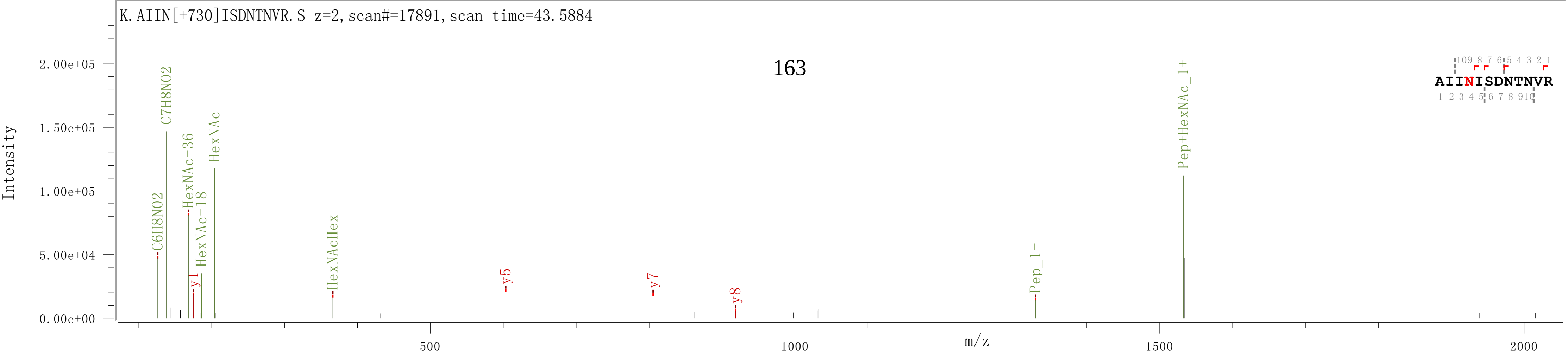

K.AIIN[+730]ISDNTNVR.S z=2,scan#=17891,scan time=43.5884
163
Intensity
m/z
C6H8NO2
C7H8NO2
HexNAc-36
HexNAc-18
y1
HexNAc
HexNAcHex
y5
y7
y8
Pep_1+
Pep+HexNAc_1+
AIINISDNTNVR

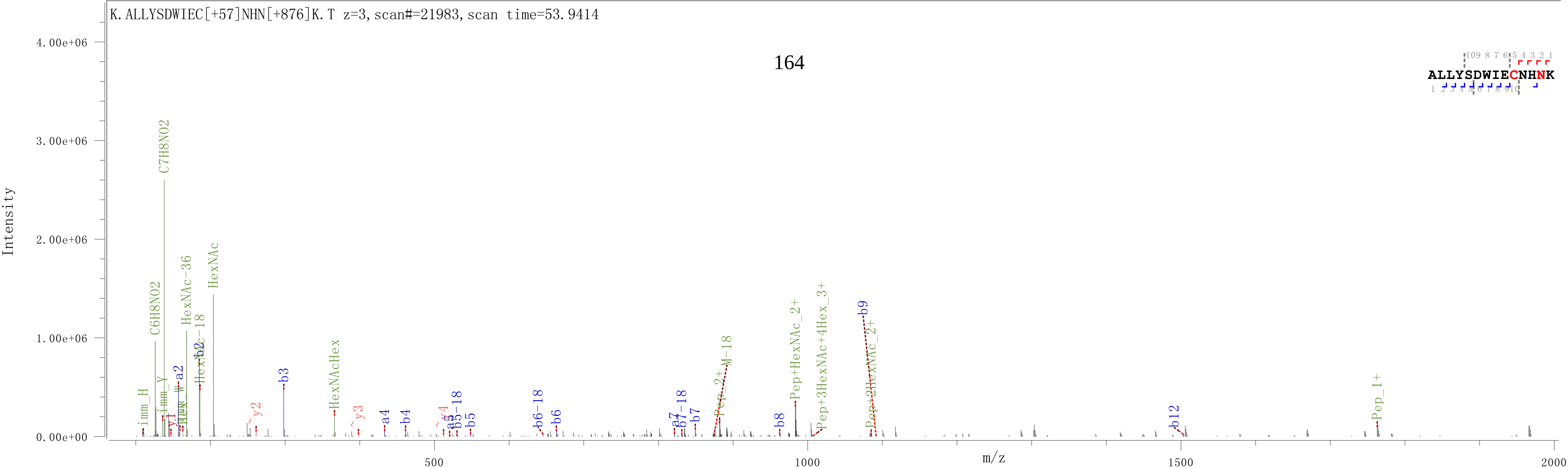

K.ALLYSDWIEC[+57]NHN[+876]K.T z=3,scan#=21983,scan time=53.9414
164
ALLYSDWIECNHNK
Intensity
m/z
4.00e+06
3.00e+06
2.00e+06
1.00e+06
0.00e+00
500
1000
1500
2000
C7H8NO2
C6H8NO2
HexNAc
HexNAc-36
HexNAc-18
HexNAcHex
b3
b2
a2
b4
a4
b5
b5-18
b6
b6-18
b7
b7-18
a7
b8
b9
b12
Pep_2+
M-18
Pep+HexNAc_2+
Pep+3HexNAc+4Hex_3+
Pep+2HexNAc_2+
Pep_1+
y2
y3
imm_H
imm_Y

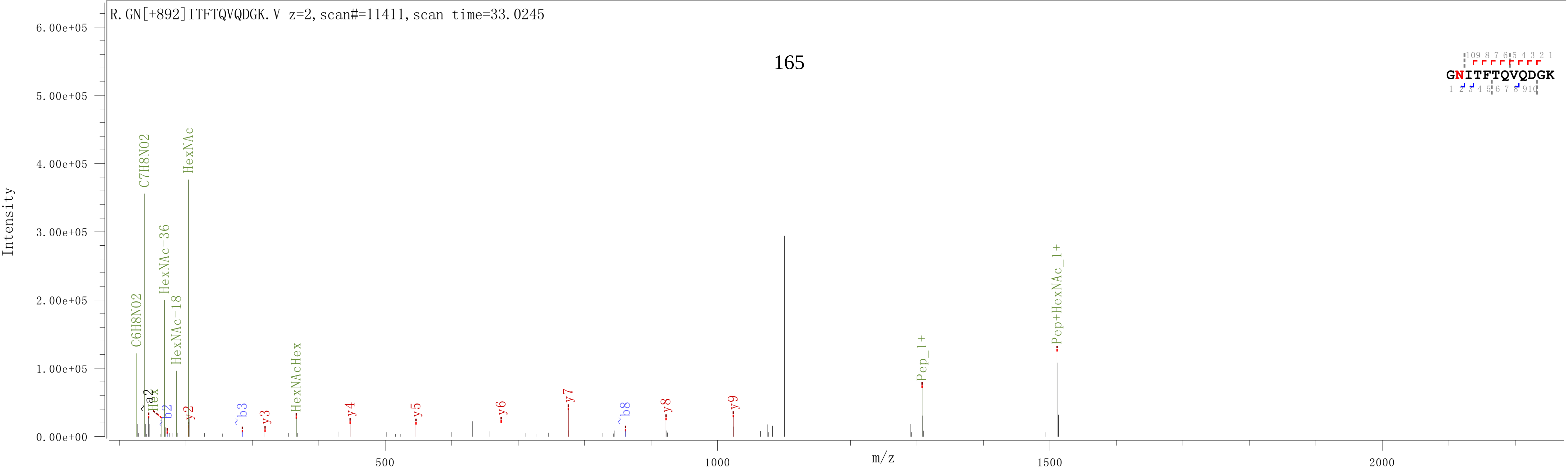

R.GN[+892]ITFTQVQDGK.V z=2,scan#=11411,scan time=33.0245
165
Intensity
m/z
GNITFTQVQDGK
C6H8NO2
C7H8NO2
HexNAc-36
HexNAc-18
HexNAc
HexNAcHex
Pep_1+
Pep+HexNAc_1+
y2
y3
y4
y5
y6
y7
y8
y9
~b2
~b3
~b8
~a2
Hex

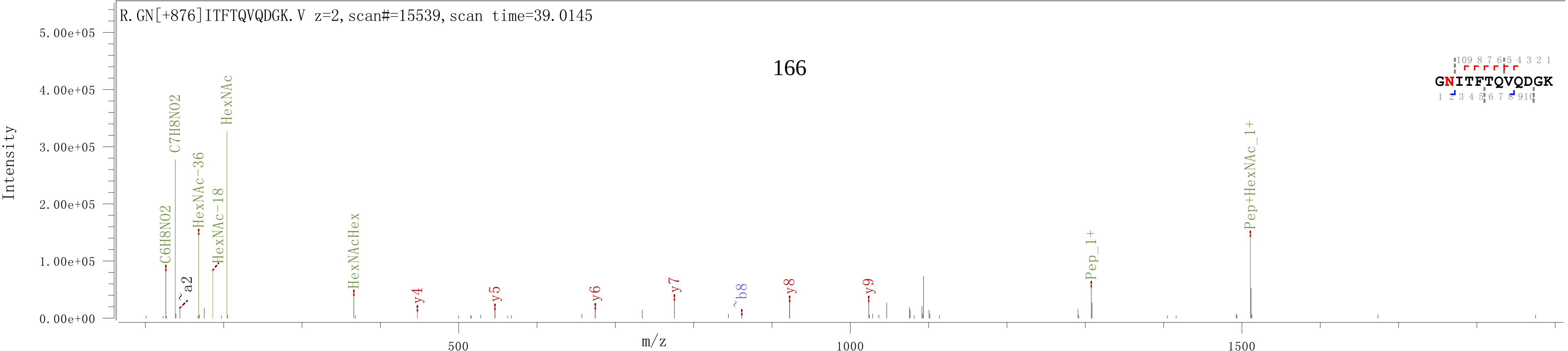

R.GN[+876]ITFTQVQDGK.V z=2,scan#=15539,scan time=39.0145
166
GNITFTQVQDGK
Intensity
m/z
C6H8NO2
C7H8NO2
~a2
HexNAc-36
HexNAc-18
HexNAc
HexNAcHex
y4
y5
y6
y7
~b8
y8
y9
Pep_1+
Pep+HexNAc_1+

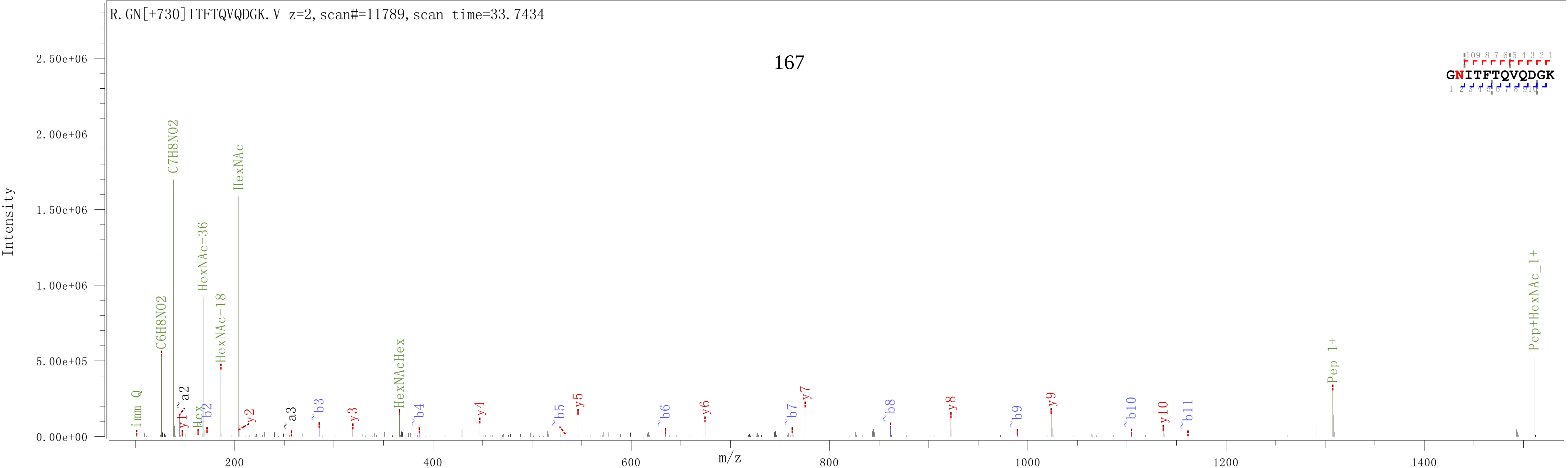

R.GN[+730]ITFTQVQDGK.V z=2,scan#=11789,scan time=33.7434
167
GNITFTQVQDGK
Intensity
m/z
C7H8NO2
HexNAc
HexNAc-36
HexNAc-18
C6H8NO2
HexNAcHex
Pep_1+
Pep+HexNAc_1+
imm_Q
Hex
y1
y2
y3
y4
y5
y6
y7
y8
y9
y10
~a2
~a3
b2
~b3
~b4
~b5
~b6
~b7
~b8
~b9
~b10
~b11

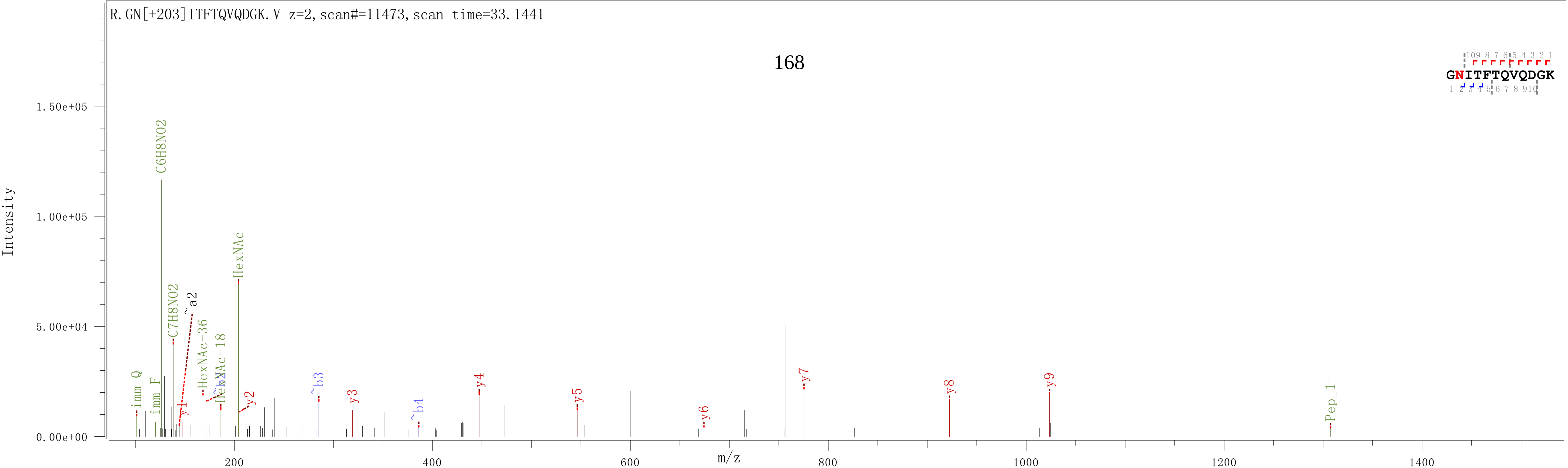
R.GN[+203]ITFTQVQDGK.V z=2,scan#=11473,scan time=33.1441
168
GNITFTQVQDGK
Intensity
m/z
C6H8NO2
C7H8NO2
HexNAc
HexNAc-36
HexNAc-18
imm_Q
imm_F
~a2
y1
y2
~b3
y3
~b4
y4
y5
y6
y7
y8
y9
Pep_1+

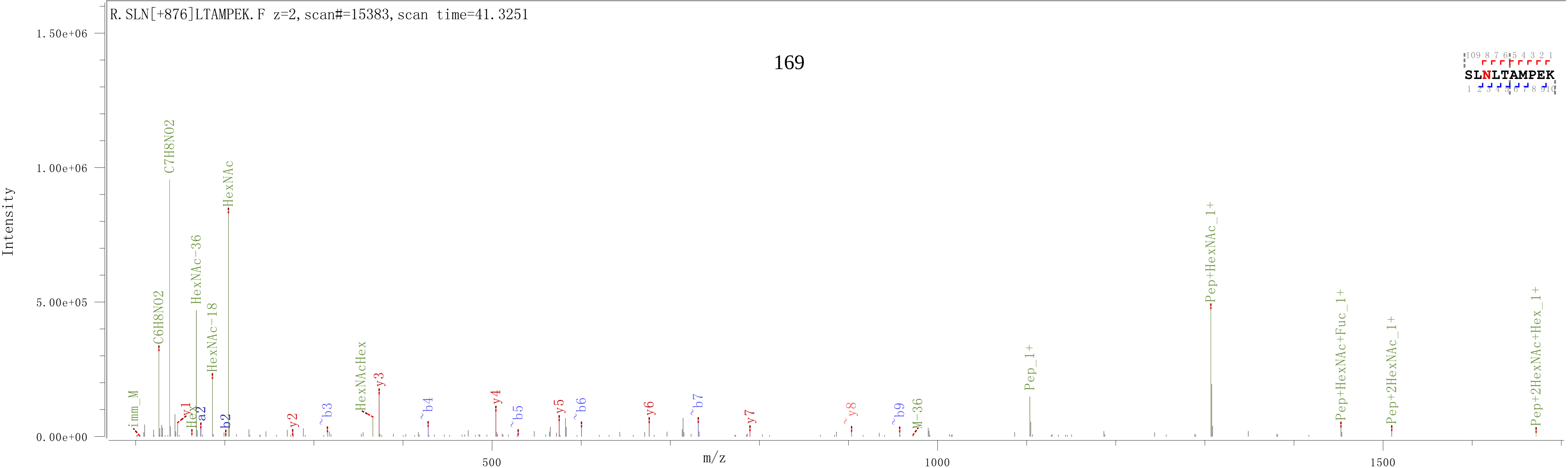
R.SLN[+876]LTAMPEK.F z=2,scan#=15383,scan time=41.3251
169
SLNLTAMPEK
Intensity
m/z
1.50e+06
1.00e+06
5.00e+05
0.00e+00
500
1000
1500
imm_M
C6H8NO2
C7H8NO2
Hex
y1
HexNAc-36
a2
HexNAc-18
b2
HexNAc
y2
~b3
HexNAcHex
y3
~b4
y4
~b5
y5
~b6
y6
~b7
y7
~y8
~b9
M-36
Pep_1+
Pep+HexNAc_1+
Pep+HexNAc+Fuc_1+
Pep+2HexNAc_1+
Pep+2HexNAc+Hex_1+

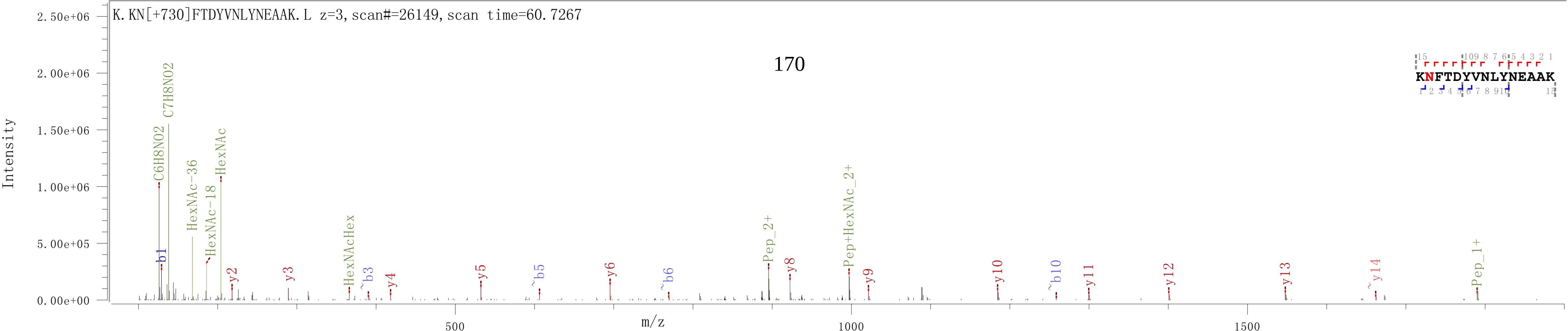
K.KN[+730]FTDYVNLYNEAAK.L z=3,scan#=26149,scan time=60.7267
Intensity
m/z
KNFTDYVNLYNEAAK
C6H8NO2
C7H8NO2
HexNAc-36
HexNAc-18
HexNAc
HexNAcHex
Pep_2+
Pep+HexNAc_2+
Pep_1+
b1
~b3
~b5
~b6
~b10
y2
y3
y4
y5
y6
y8
y9
y10
y11
y12
y13
~y14

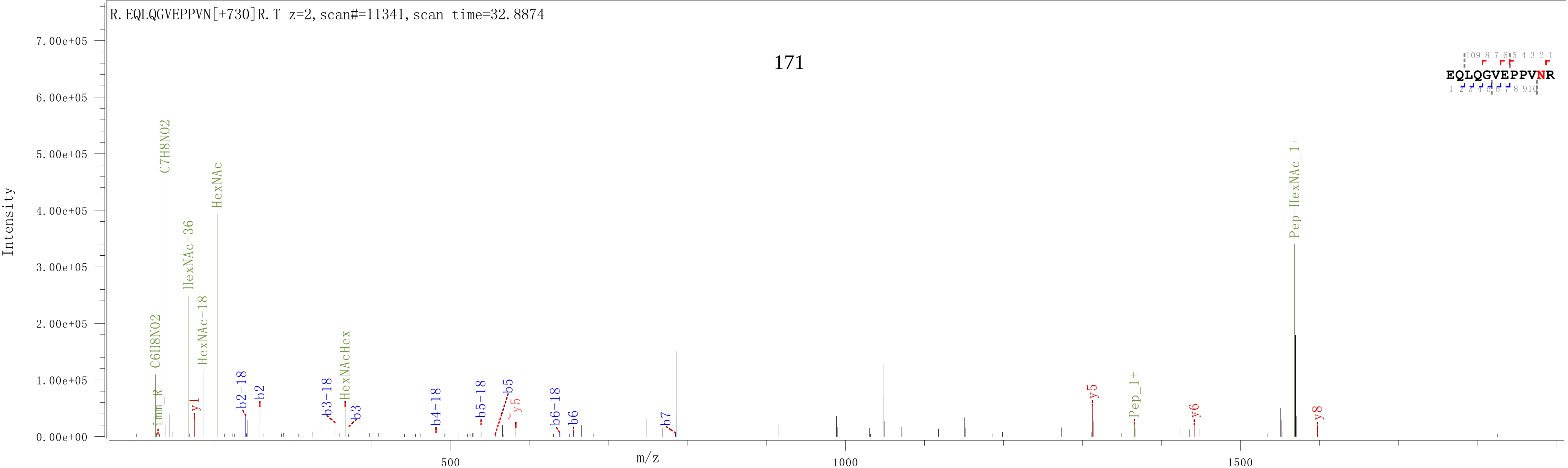
R.EQLQGVEPPVN[+730]R.T z=2,scan#=11341,scan time=32.8874
EQLQGVEPPVNR
Intensity
m/z
7.00e+05
6.00e+05
5.00e+05
4.00e+05
3.00e+05
2.00e+05
1.00e+05
0.00e+00
500
1000
1500
C6H8NO2
imm_R
C7H8NO2
HexNAc-36
y1
HexNAc-18
HexNAc
b2-18
b2
b3-18
HexNAcHex
b3
b4-18
b5-18
b5
y5
b6-18
b6
b7
y5
Pep_1+
y6
Pep+HexNAc_1+
y8

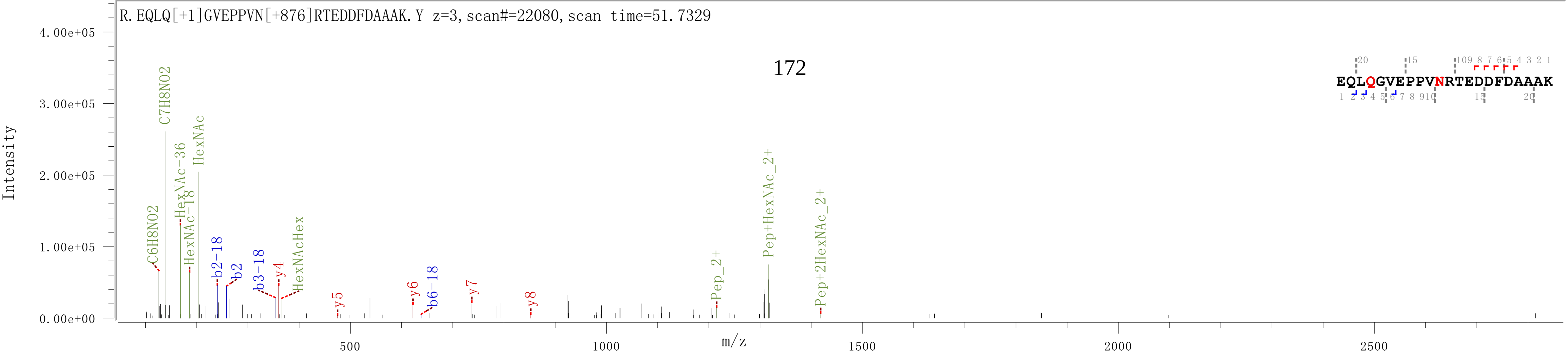
R.EQLQ[+1]GVEPPVN[+876]RTEDDFDAAAK.Y z=3,scan#=22080,scan time=51.7329
172
Intensity
m/z
EQLQGVEPPVNRTEDDFDAAAK
C7H8NO2
C6H8NO2
HexNAc-36
HexNAc-18
HexNAc
b2-18
b2
b3-18
y4
HexNAcHex
y5
y6
b6-18
y7
y8
Pep_2+
Pep+HexNAc_2+
Pep+2HexNAc_2+

R.EQLQGVEPPVN[+730]RTEDDFDAAAK.Y z=3,scan#=20544,scan time=51.3041

173

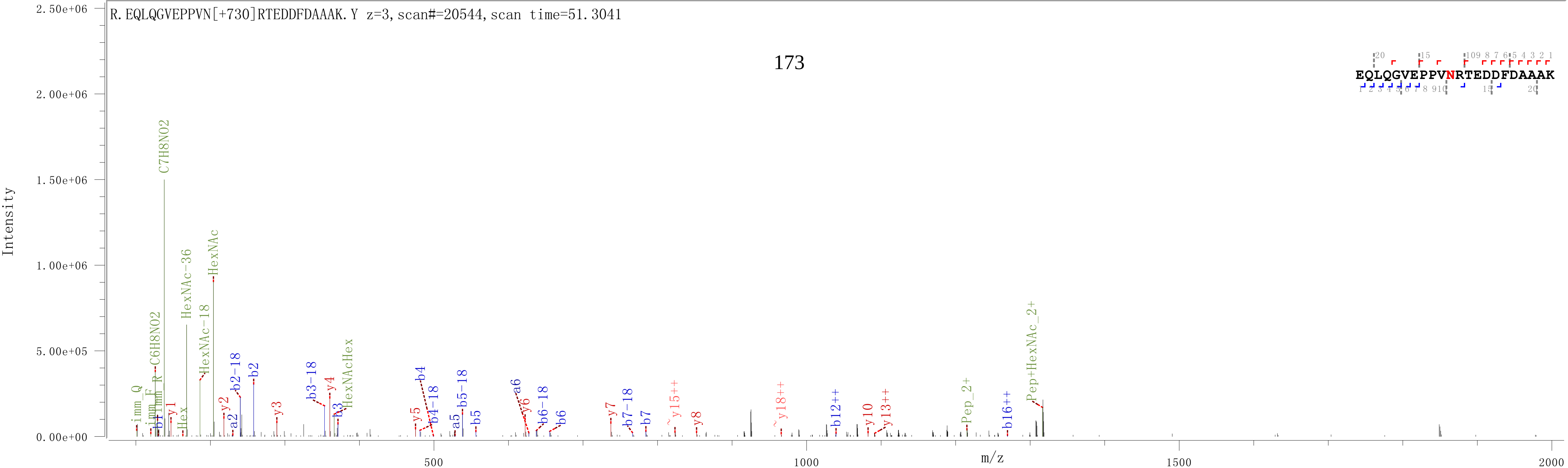

R.ASLAEWEYTSN[+730]ITKENEEK.S z=3,scan#=20745,scan time=51.6547

174

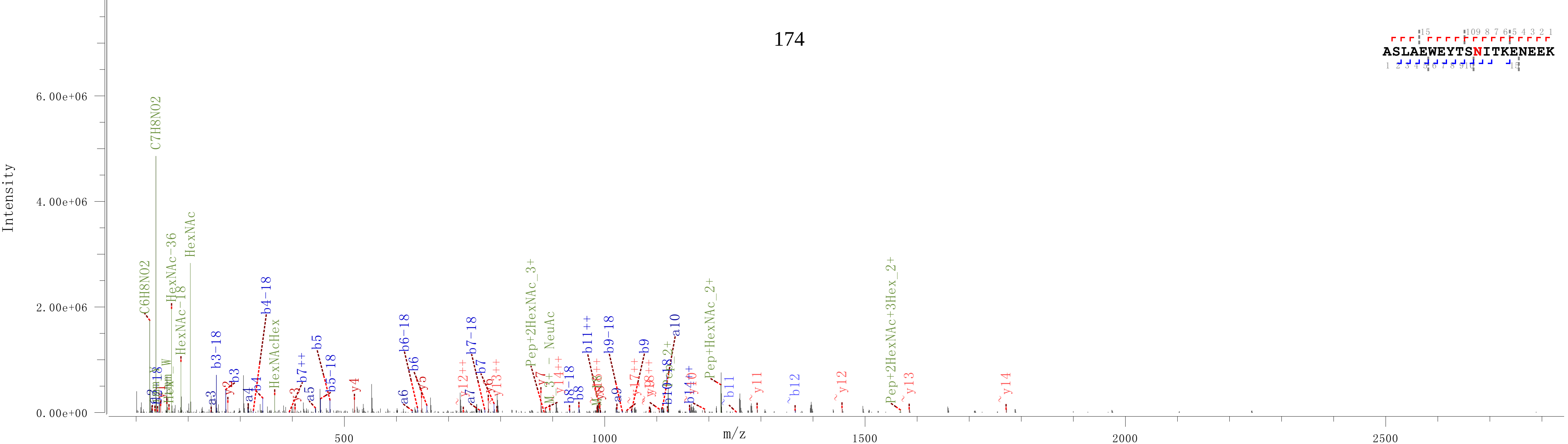

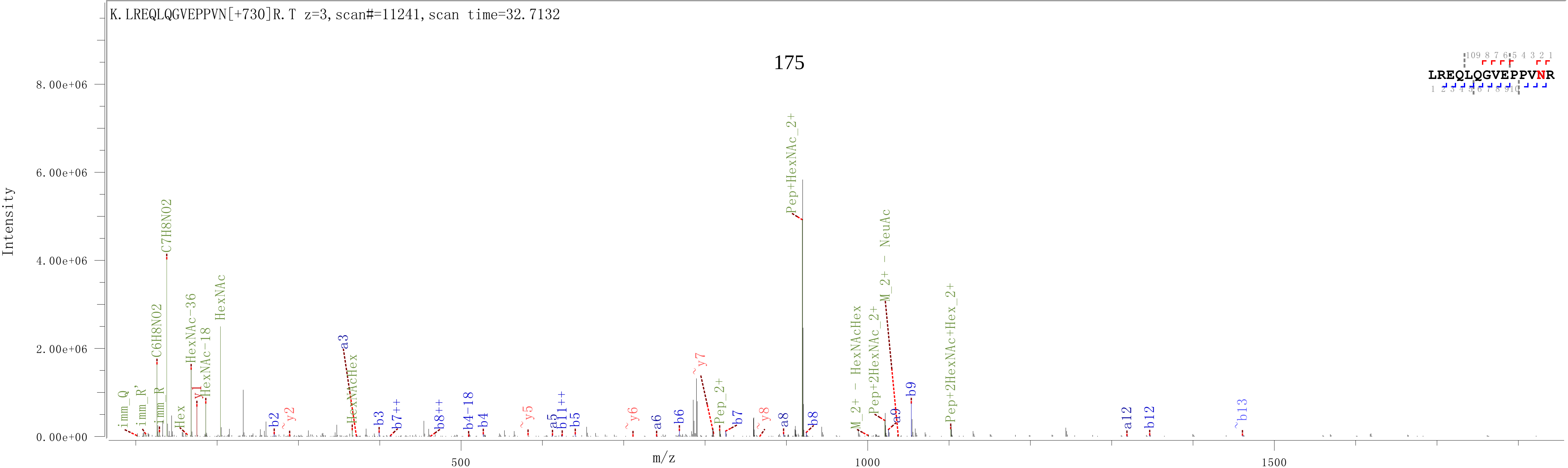
K.LREQLQGVEPPVN[+730]R.T z=3,scan#=11241,scan time=32.7132
LREQLQGVEPPVNR
Intensity
m/z
8.00e+06
6.00e+06
4.00e+06
2.00e+06
0.00e+00
500
1000
1500
imm_Q
imm_R'
imm_R
C6H8NO2
C7H8NO2
Hex
HexNAc-36
y1
HexNAc-18
HexNAc
b2
~y2
a3
HexNAcHex
b3
b7++
b8++
b4-18
b4
~y5
a5
b11++
b5
~y6
a6
b6
~y7
Pep_2+
b7
~y8
a8
Pep+HexNAc_2+
b8
M_2+ - HexNAcHex
Pep+2HexNAc_2+
M_2+ - NeuAc
a9
b9
Pep+2HexNAc+Hex_2+
a12
b12
~b13

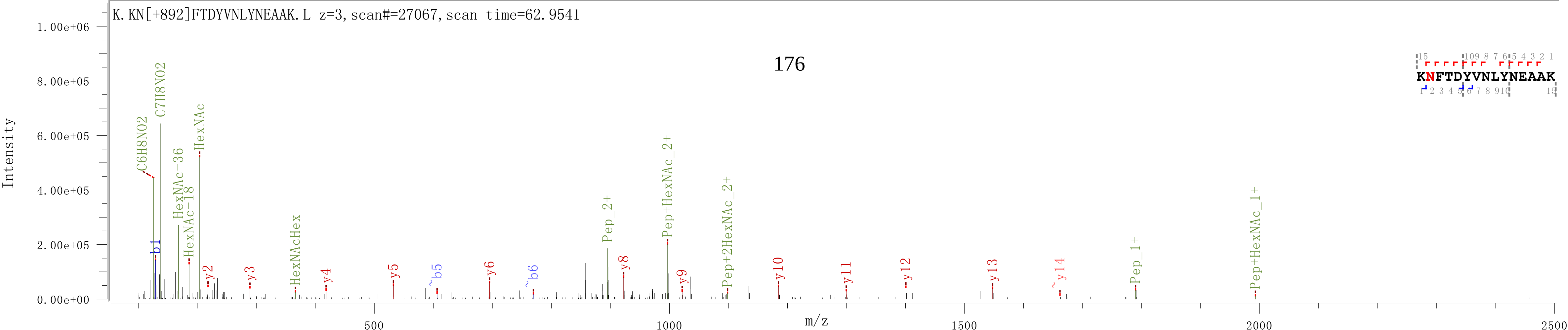
K.KN[+892]FTDYVNLYNEAAK.L z=3,scan#=27067,scan time=62.9541
176
KNFTDYVNLYNEAAK
Intensity
m/z
C6H8NO2
C7H8NO2
HexNAc-36
HexNAc-18
HexNAc
HexNAcHex
Pep_2+
Pep+HexNAc_2+
Pep+2HexNAc_2+
Pep_1+
Pep+HexNAc_1+
b1
~b5
~b6
y2
y3
y4
y5
y6
y8
y9
y10
y11
y12
y13
~y14

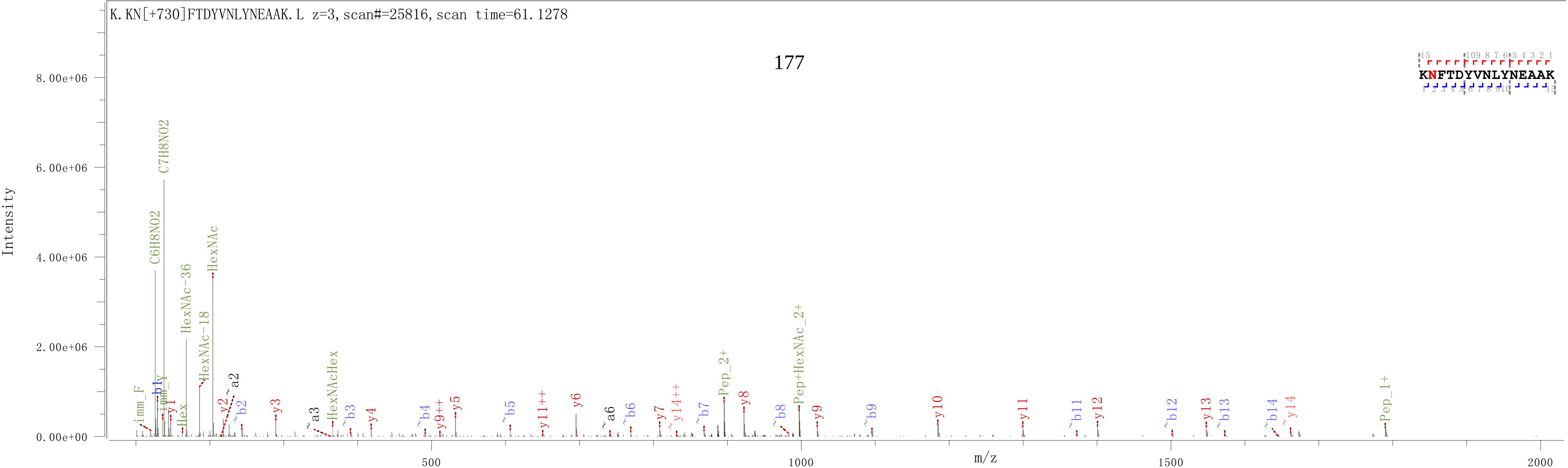
K.KN[+730]FTDYVNLYNEAAK.L z=3,scan#=25816,scan time=61.1278
KNFTDYVNLYNEAAK
Intensity
m/z
8.00e+06
6.00e+06
4.00e+06
2.00e+06
0.00e+00
500
1000
1500
2000
C7H8NO2
C6H8NO2
HexNAc
HexNAc-36
HexNAc-18
HexNAcHex
Pep_2+
Pep+HexNAc_2+
Pep_1+
imm_F
imm_Y
Hex
b1
y1
y2
~a2
y3
~a3
~b2
~b3
y4
~b4
y9++
y5
~b5
y11++
y6
~a6
~b6
y7
y14++
~b7
y8
~b8
y9
~b9
y10
y11
~b11
y12
~b12
y13
~b13
~b14
y14

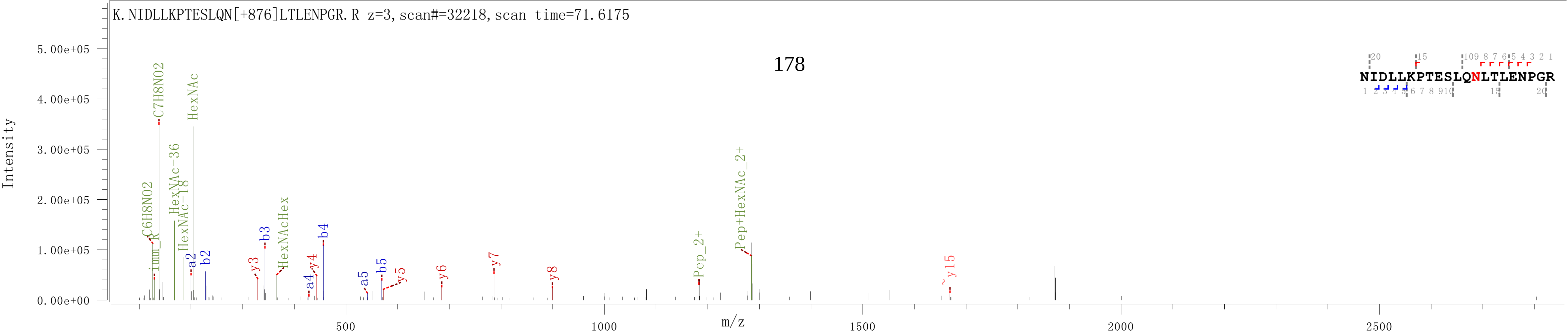

K.NIDLLKPTESLQN[+876]LTLENPGR.R z=3,scan#=32218,scan time=71.6175
178
NIDLLKPTESLQNLTLENPGR
Intensity
m/z
C7H8NO2
HexNAc
HexNAc-36
HexNAc-18
C6H8NO2
HexNAcHex
Pep_2+
Pep+HexNAc_2+
a2
b2
b3
y3
a4
y4
b4
a5
b5
y5
y6
y7
y8
y15

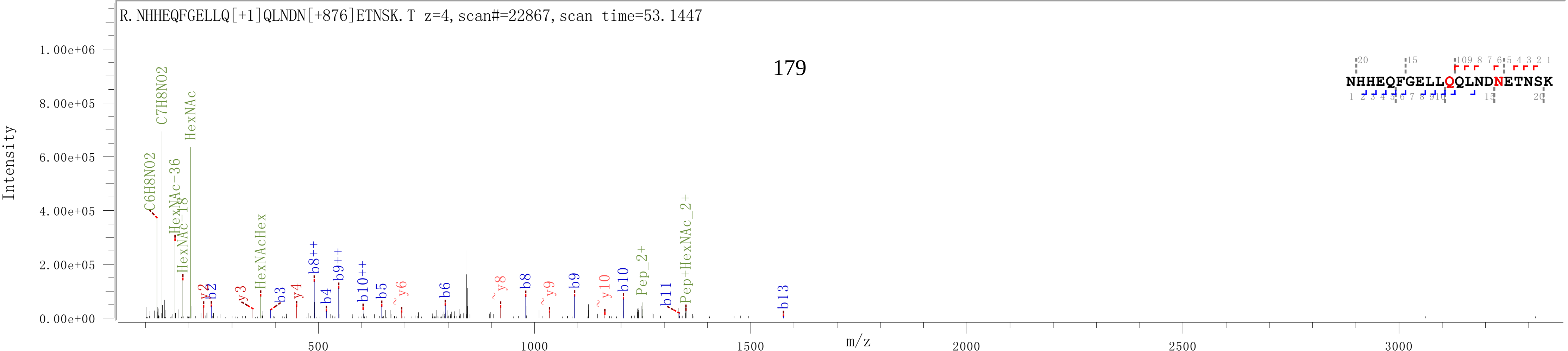

R.NHHEQFGELLQ[+1]QLNDN[+876]ETNSK.T z=4,scan#=22867,scan time=53.1447
179
Intensity
m/z
NHHEQFGELLQQLNDNETNSK
C6H8NO2
C7H8NO2
HexNAc-36
HexNAc-18
HexNAc
y2
b2
y3
HexNAcHex
b3
y4
b8++
b4
b9++
b10++
b5
~y6
b6
~y8
b8
~y9
b9
~y10
b10
Pep_2+
b11
Pep+HexNAc_2+
b13

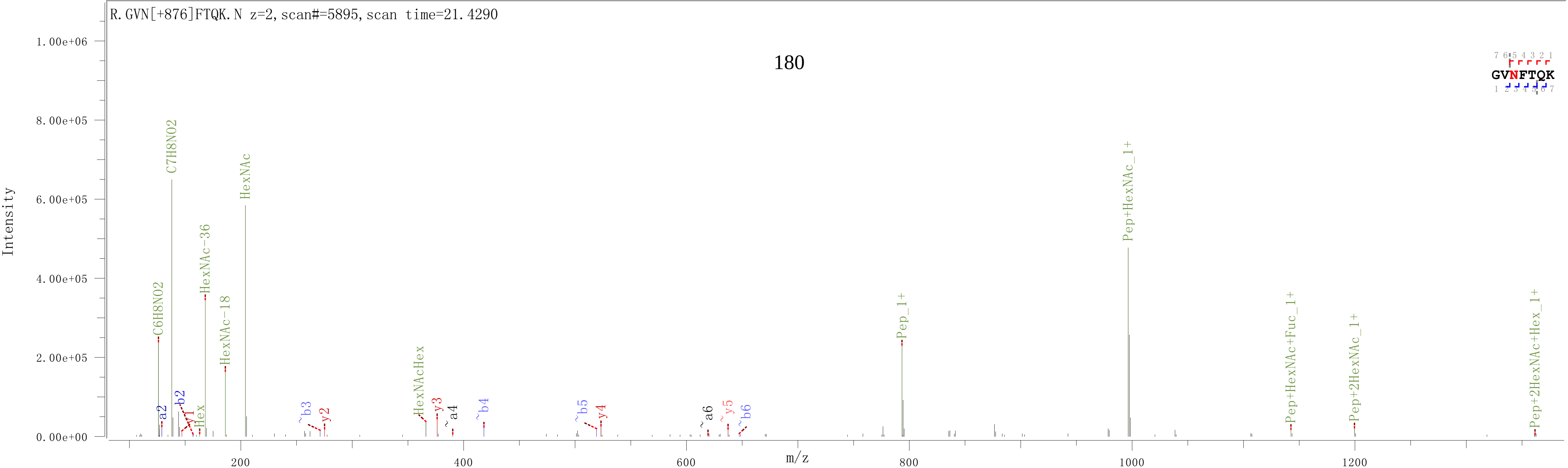
R.GVN[+876]FTQK.N z=2,scan#=5895,scan time=21.4290
180
GVNFTQK
Intensity
m/z
C6H8NO2
C7H8NO2
HexNAc-36
HexNAc-18
HexNAc
HexNAcHex
Pep_1+
Pep+HexNAc_1+
Pep+HexNAc+Fuc_1+
Pep+2HexNAc_1+
Pep+2HexNAc+Hex_1+
Hex
a2
b2
y1
~b3
y2
y3
~a4
~b4
~b5
y4
~a6
~y5
~b6
1.00e+06
8.00e+05
6.00e+05
4.00e+05
2.00e+05
0.00e+00
200
400
600
800
1000
1200

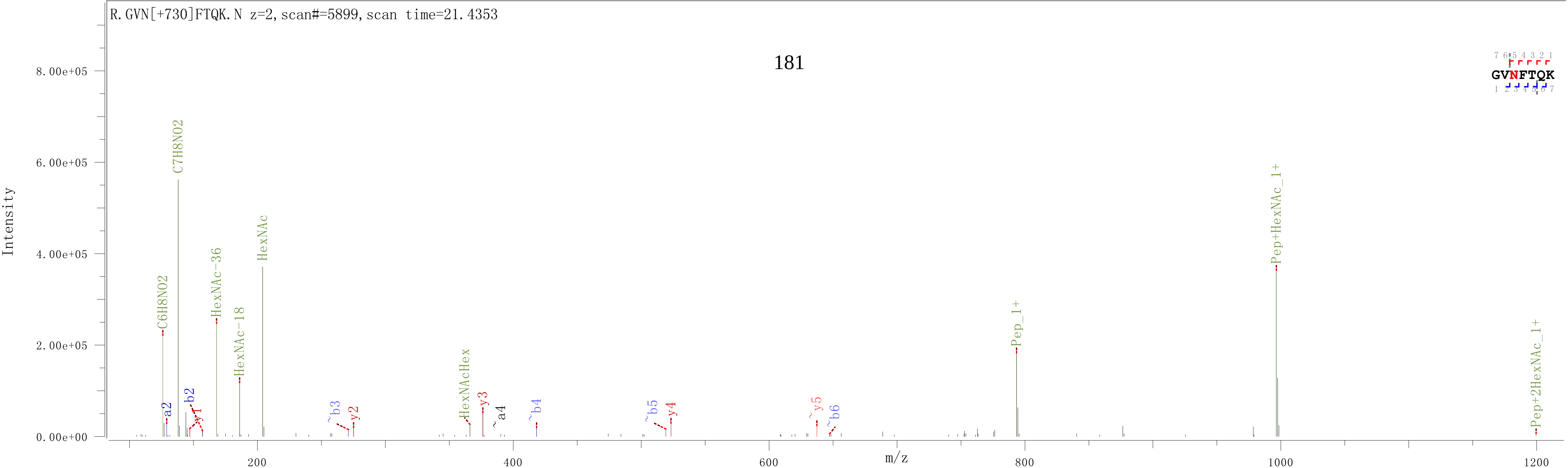

R.GVN[+730]FTQK.N z=2,scan#=5899,scan time=21.4353
181
Intensity
m/z
8.00e+05
6.00e+05
4.00e+05
2.00e+05
0.00e+00
200
400
600
800
1000
1200
C7H8NO2
C6H8NO2
HexNAc-36
HexNAc-18
HexNAc
HexNAcHex
Pep_1+
Pep+HexNAc_1+
Pep+2HexNAc_1+
a2
b2
y1
~b3
y2
y3
~a4
~b4
~b5
y4
~y5
~b6
GVNFTQK

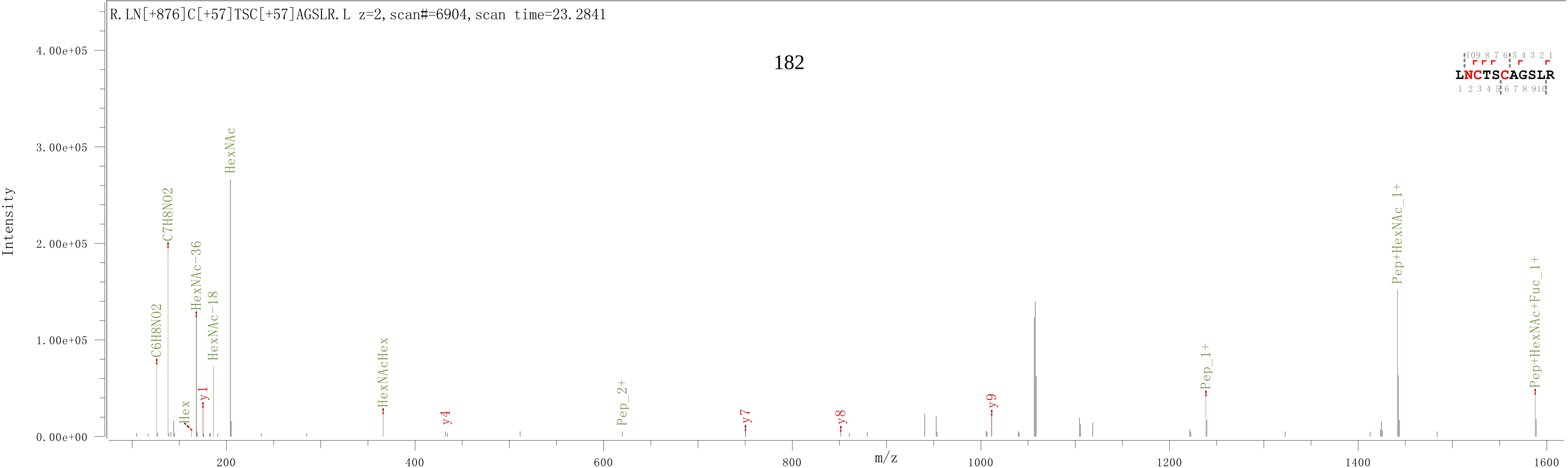
R.LN[+876]C[+57]TSC[+57]AGSLR.L z=2,scan#=6904,scan time=23.2841
182
LNCTSCAGSLR
Intensity
m/z
C6H8NO2
C7H8NO2
Hex
HexNAc-36
y1
HexNAc-18
HexNAc
HexNAcHex
y4
Pep_2+
y7
y8
y9
Pep_1+
Pep+HexNAc_1+
Pep+HexNAc+Fuc_1+

183

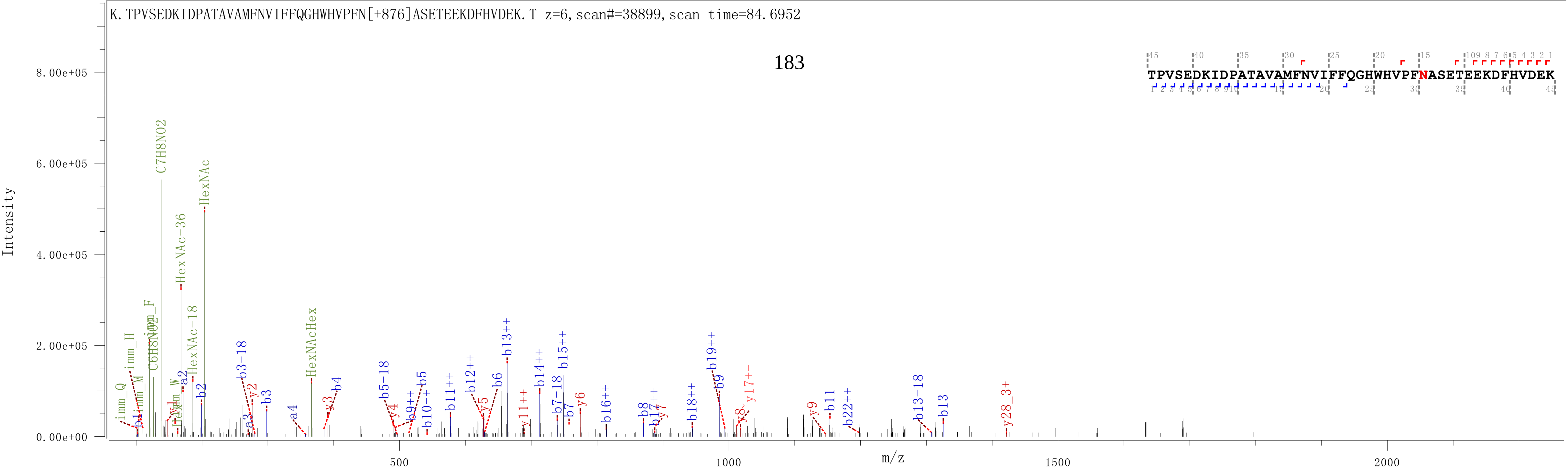
K.TPVSEDKIDPATAVAMFNVIFFQGHWHVPFN[+876]ASETEEKDFHVDEK.T z=6,scan#=38899,scan time=84.6952
TPVSEDKIDPATAVAMFNVIFFQGHWHVPFNASETEEKDFHVDEK
Intensity
m/z
8.00e+05
6.00e+05
4.00e+05
2.00e+05
0.00e+00
500
1000
1500
2000
C7H8NO2
HexNAc
HexNAc-36
HexNAc-18
HexNAcHex
C6H8NO2
imm_H
imm_Q
imm_M
imm_W
b3-18
b2
a2
b3
b4
a4
y2
y3
b5-18
b5
y4
b9++
b10++
b11++
b12++
y5
b6
b13++
y11++
b14++
b7-18
b7
b15++
y6
b16++
b8
b17++
y7
b18++
b19++
b9
y8
y17++
y9
b11
b22++
b13-18
b13
y28_3+

184

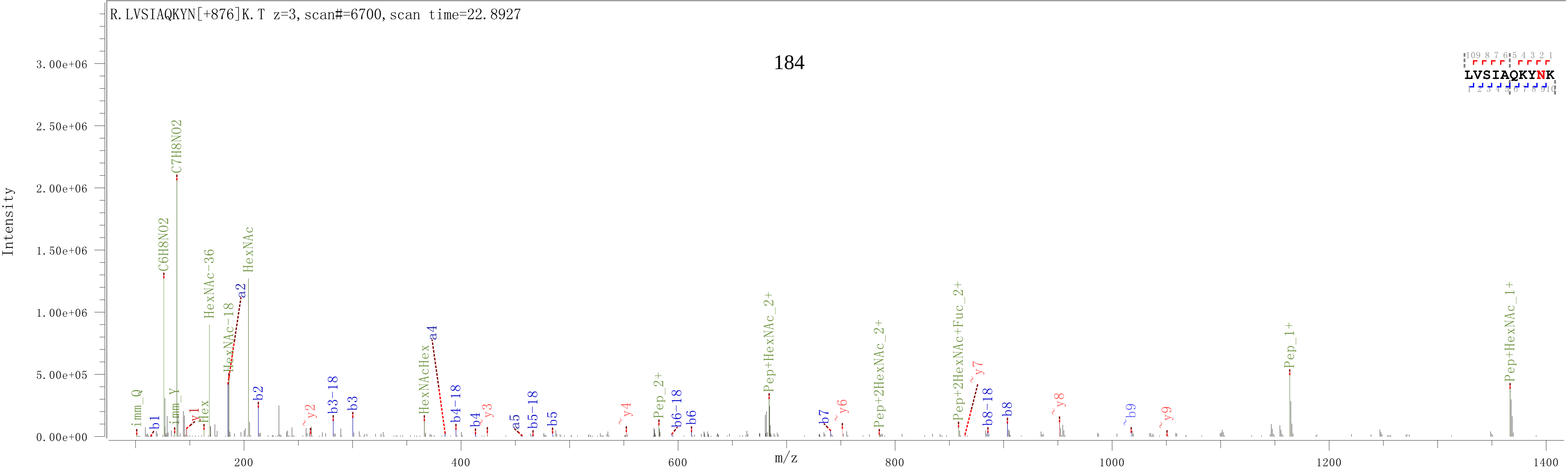
R.LVSIAQKYN[+876]K.T z=3,scan#=6700,scan time=22.8927
Intensity
m/z
LVSIAQKYNK
C7H8NO2
C6H8NO2
HexNAc
HexNAc-36
HexNAc-18
a2
b2
b3-18
b3
HexNAcHex
a4
b4-18
b4
a5
b5-18
b5
Pep_2+
b6-18
b6
Pep+HexNAc_2+
b7
Pep+2HexNAc_2+
Pep+2HexNAc+Fuc_2+
b8-18
b8
Pep_1+
Pep+HexNAc_1+

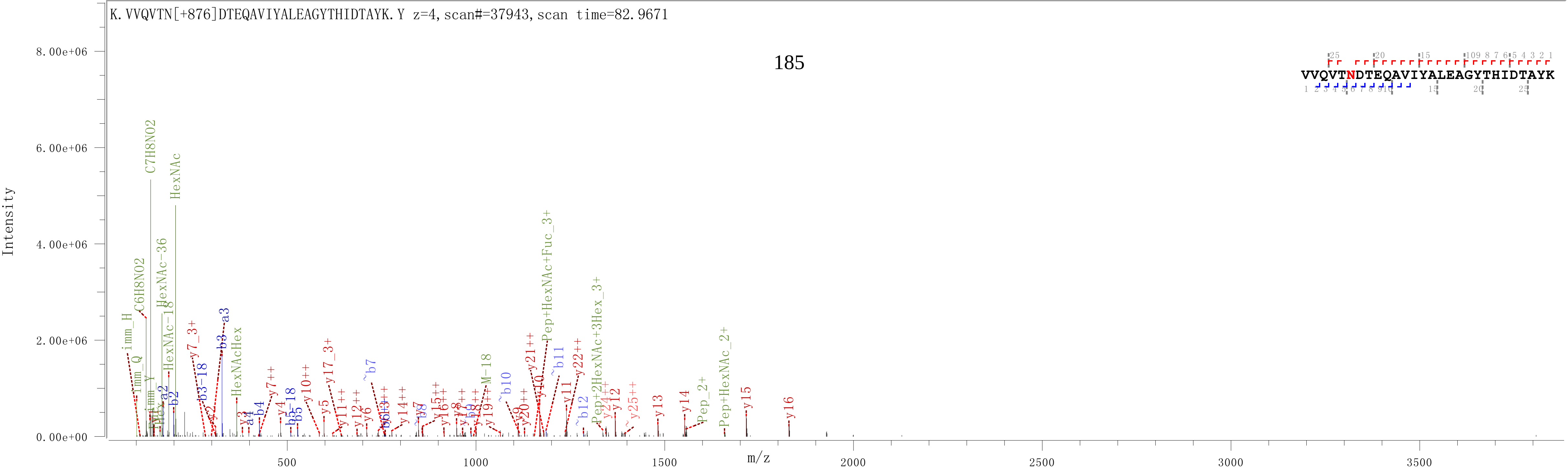
K.VVQVTN[+876]DTEQAVIYALEAGYTHIDTAYK.Y z=4,scan#=37943,scan time=82.9671
VVQVTNDTEQAVIYALEAGYTHIDTAYK
Intensity
m/z

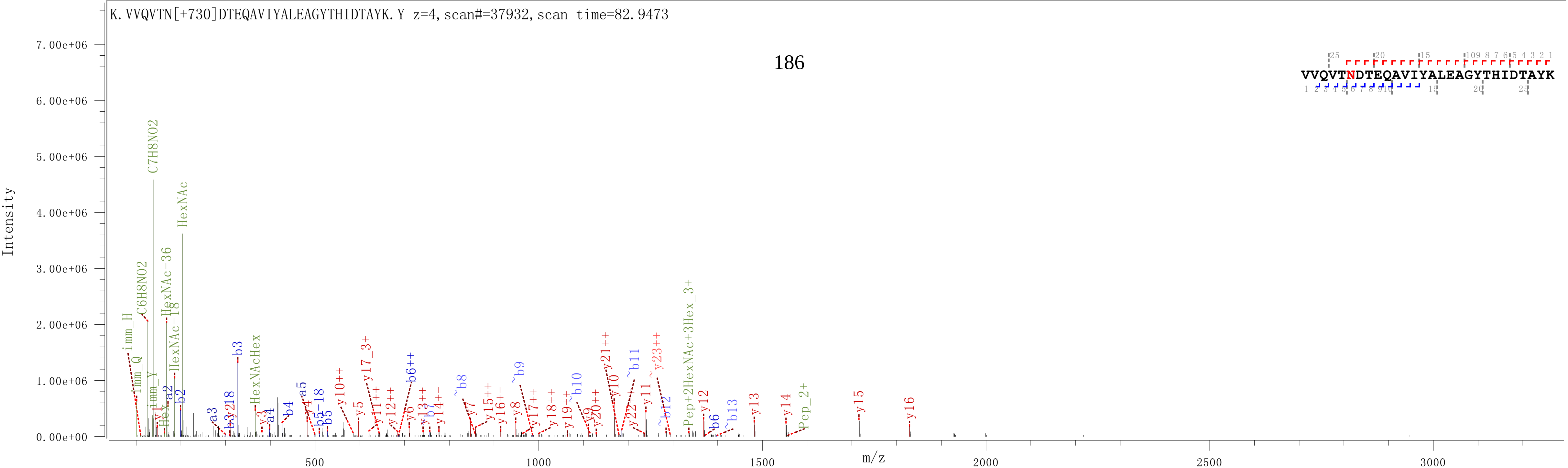
K.VVQVTN[+730]DTEQAVIYALEAGYTHIDTAYK.Y z=4,scan#=37932,scan time=82.9473
VVQVTNDTEQAVIYALEAGYTHIDTAYK
Intensity
m/z
7.00e+06
6.00e+06
5.00e+06
4.00e+06
3.00e+06
2.00e+06
1.00e+06
0.00e+00
500
1000
1500
2000
2500
3000
C7H8NO2
HexNAc
HexNAc-36
HexNAc-18
C6H8NO2
imm_H
imm_Q
imm_Y
HexNAcHex
b3
b2
a2
a3
a4
a5
b4
y3
y4
y5
y6
y7
y8
y10
y11
y12
y13
y14
y15
y16
y10++
y11++
y12++
y13++
y14++
y15++
y16++
y17++
y18++
y19++
y20++
y21++
y22++
y23++
y17_3+
b6++
~b8
~b9
~b10
~b11
~b12
~b13
b6
Pep+2HexNAc+3Hex_3+
Pep_2+

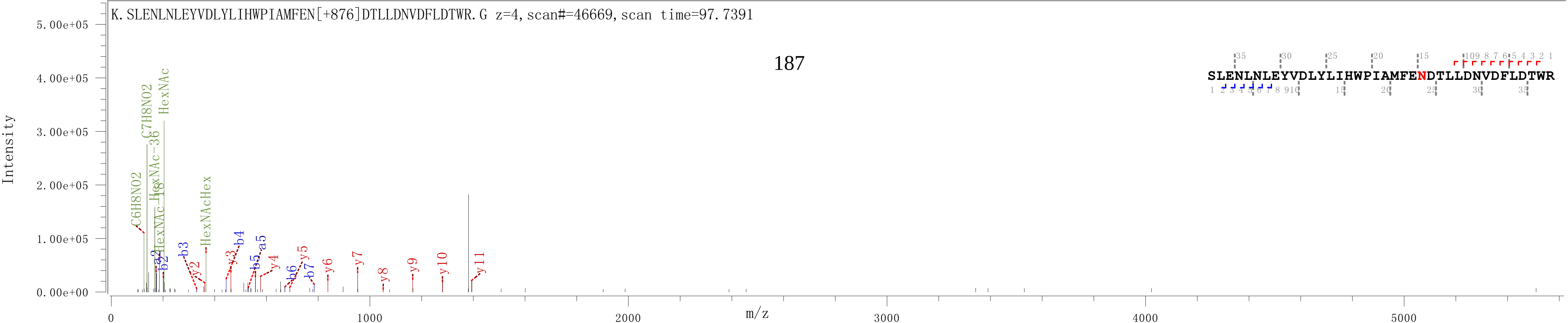
K.SLENLNLEYVDLYLIHWPIAMFEN[+876]DTLLDNVDFLDTWR.G z=4,scan#=46669,scan time=97.7391
SLENLNLEYVDLYLIHWPIAMFENDTLLDNVDFLDTWR
Intensity
m/z
5.00e+05
4.00e+05
3.00e+05
2.00e+05
1.00e+05
0.00e+00
0
1000
2000
3000
4000
5000
C6H8NO2
C7H8NO2
HexNAc-36
HexNAc-18
HexNAc
HexNAcHex
a2
b2
b3
y2
y3
b4
b5
a5
y4
b6
y5
b7
y6
y7
y8
y9
y10
y11

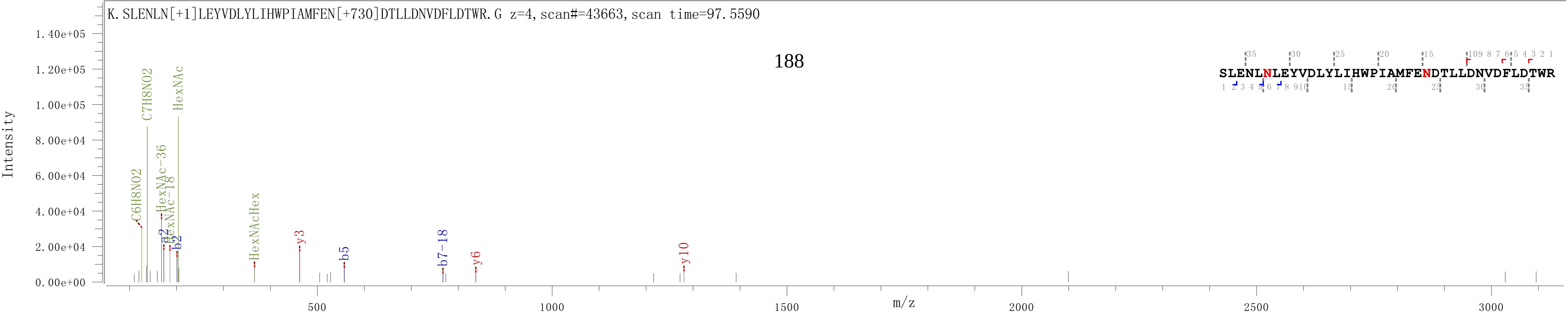

K.SLENLN[+1]LEYVDLYLIHWPIAMFEN[+730]DTLLDNVDFLDTWR.G z=4,scan#=43663,scan time=97.5590
188
SLENLNLEYVDLYLIHWPIAMFENDTLLDNVDFLDTWR
Intensity
m/z
C6H8NO2
C7H8NO2
HexNAc-36
HexNAc-18
HexNAc
a2
b2
HexNAcHex
y3
b5
b7-18
y6
y10

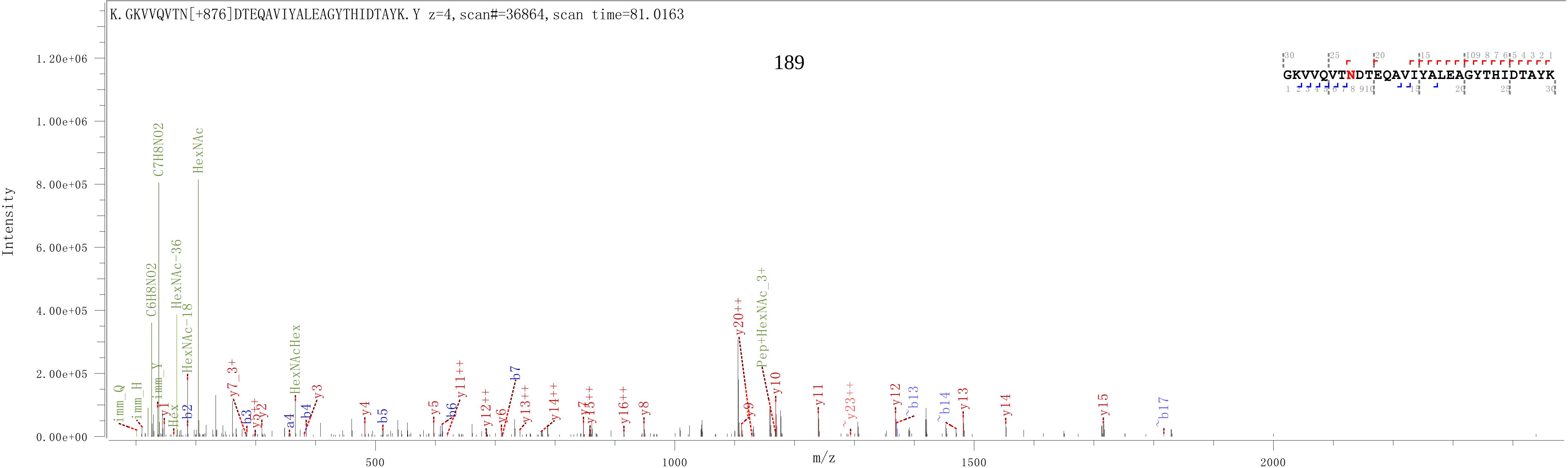
K.GKVVQVTN[+876]DTEQAVIYALEAGYTHIDTAYK.Y z=4,scan#=36864,scan time=81.0163
189
GKVVQVTNDTEQAVIYALEAGYTHIDTAYK
Intensity
m/z
C7H8NO2
HexNAc
C6H8NO2
HexNAc-36
HexNAc-18
HexNAcHex
y20++
Pep+HexNAc_3+
b7
y11++
y7_3+
y3
y10
y11
y12
y13
y14
y15
~b13
~b14
~b17
~y23++
y12++
y13++
y14++
y15++
y16++
y5
y6
y7
y8
y9
y4
b5
b6
a4
b4
b3
b2
y1
y2
y3++
imm_Q
imm_H
imm_Y
Hex
1.20e+06
1.00e+06
8.00e+05
6.00e+05
4.00e+05
2.00e+05
0.00e+00
500
1000
1500
2000

190

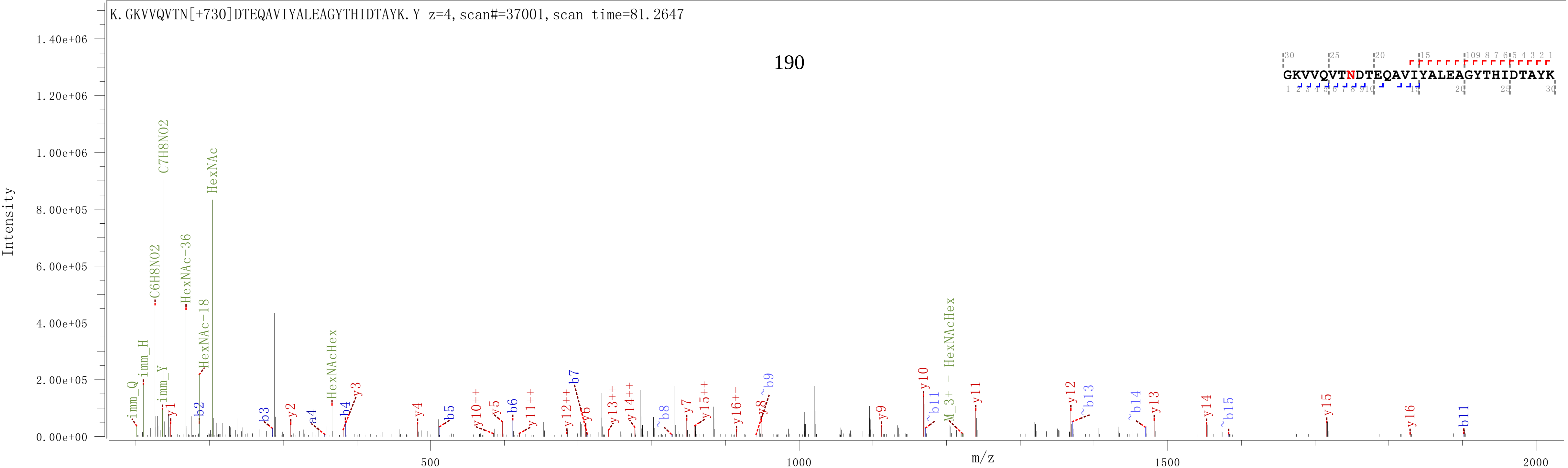

K.GKVVQVTN[+730]DTEQAVIYALEAGYTHIDTAYK.Y z=4,scan#=37001,scan time=81.2647
GKVVQVTNDTEQAVIYALEAGYTHIDTAYK
Intensity
m/z
C7H8NO2
HexNAc
C6H8NO2
HexNAc-36
HexNAc-18
HexNAcHex
M_3+ - HexNAcHex
imm_Q
imm_H
imm_Y

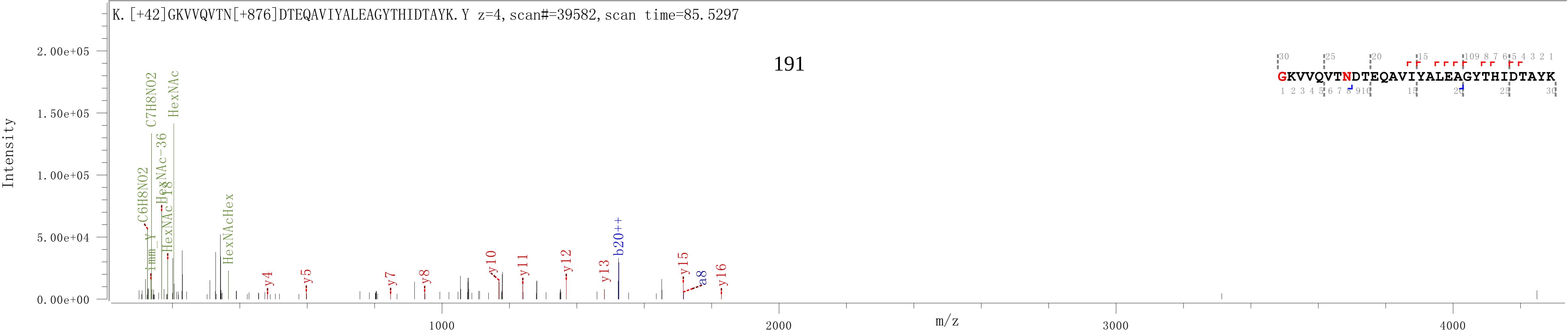

K.[+42]GKVVQVTN[+876]DTEQAVIYALEAGYTHIDTAYK.Y z=4,scan#=39582,scan time=85.5297
191
GKVVQVTNDTEQAVIYALEAGYTHIDTAYK
Intensity
m/z
0.00e+00
5.00e+04
1.00e+05
1.50e+05
2.00e+05
1000
2000
3000
4000
C6H8NO2
imm_Y
C7H8NO2
HexNAc-36
HexNAc-18
HexNAc
HexNAcHex
y4
y5
y7
y8
y10
y11
y12
y13
b20++
y15
a8
y16

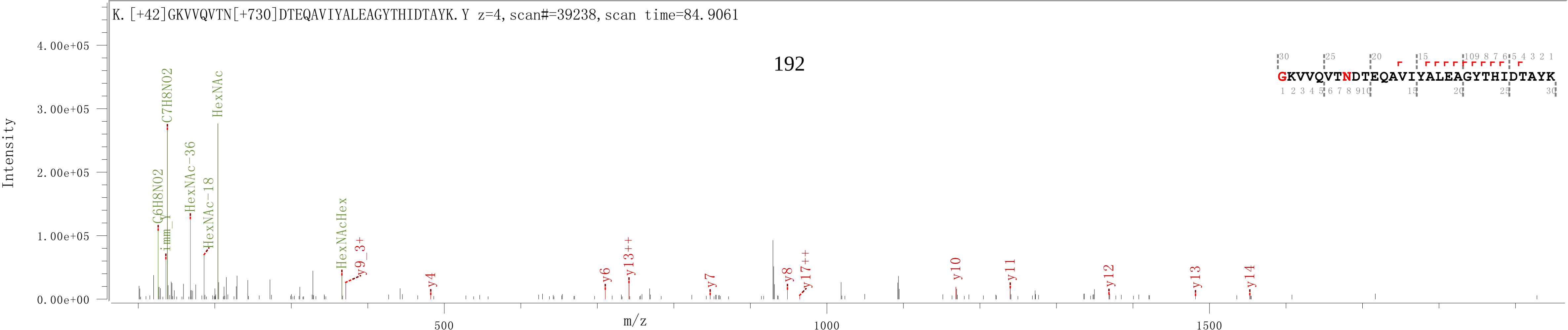

K.[+42]GKVVQVTN[+730]DTEQAVIYALEAGYTHIDTAYK.Y z=4,scan#=39238,scan time=84.9061
Intensity
4.00e+05
3.00e+05
2.00e+05
1.00e+05
0.00e+00
m/z
500
1000
1500
C6H8NO2
C7H8NO2
imm_Y
HexNAc-36
HexNAc-18
HexNAc
HexNAcHex
y9_3+
y4
y6
y13++
y7
y8
y17++
y10
y11
y12
y13
y14
GKVVQVTNDTEQAVIYALEAGYTHIDTAYK

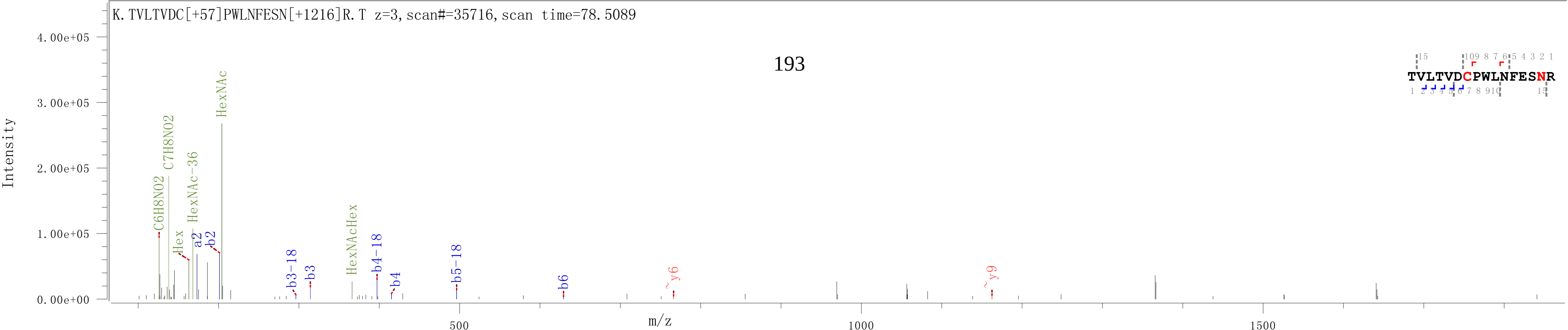
K.TVLTVDC[+57]PWLNFESN[+1216]R.T z=3,scan#=35716,scan time=78.5089
193
Intensity
m/z
4.00e+05
3.00e+05
2.00e+05
1.00e+05
0.00e+00
500
1000
1500
C6H8NO2
C7H8NO2
Hex
HexNAc-36
a2
b2
HexNAc
b3-18
b3
HexNAcHex
b4-18
b4
b5-18
b6
~y6
~y9
TVLTVDCPWLNFESNR

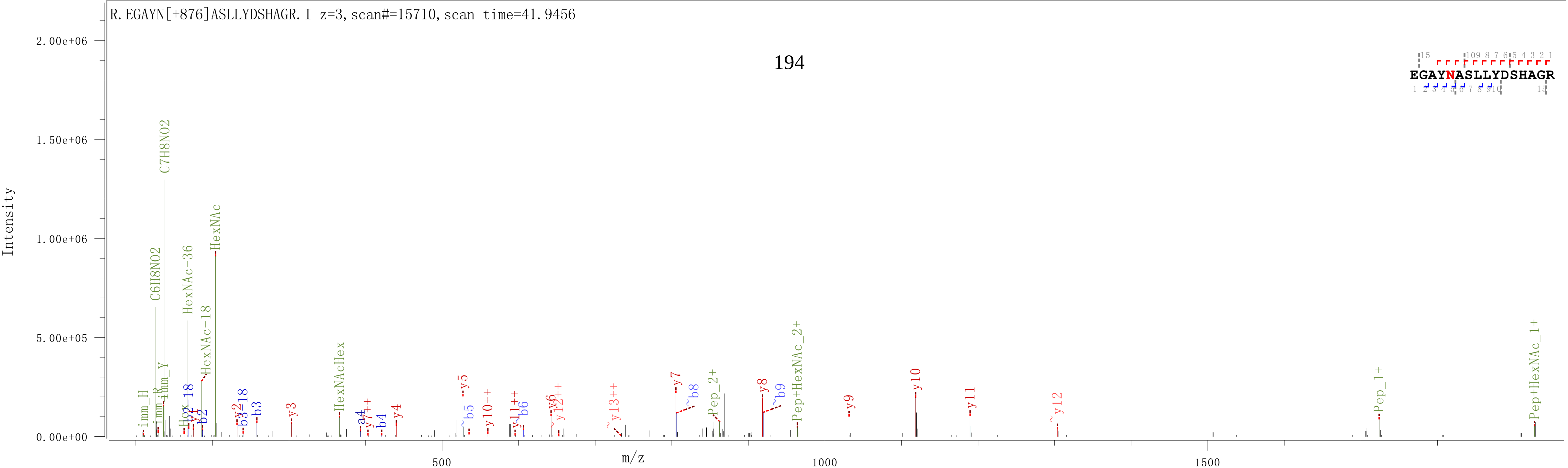
R.EGAYN[+876]ASLLYDSHAGR.I z=3,scan#=15710,scan time=41.9456
194
EGAYNASLLYDSHAGR
Intensity
2.00e+06
1.50e+06
1.00e+06
5.00e+05
0.00e+00
m/z
500
1000
1500
C7H8NO2
C6H8NO2
HexNAc
HexNAc-36
HexNAc-18
HexNAcHex
Pep+HexNAc_2+
Pep+HexNAc_1+
Pep_2+
Pep_1+
y3
y4
y5
y7
y8
y9
y10
y11
~y12
y10++
~y13++
b3
b4
~b8
~b9

195

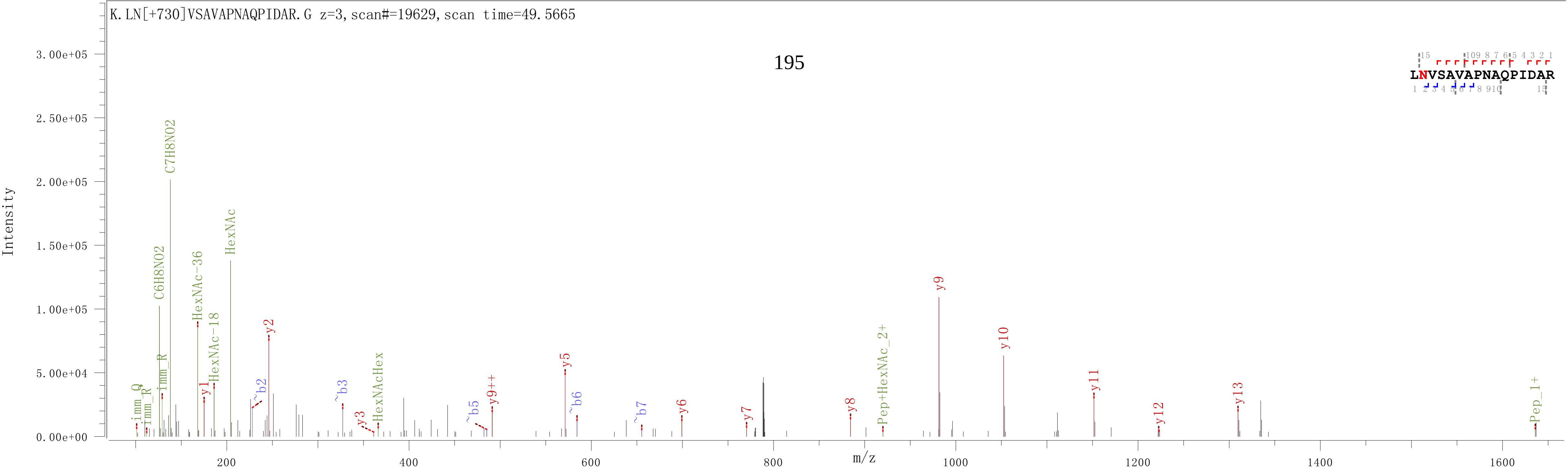
K.LN[+730]VSAVAPNAQPIDAR.G z=3,scan#=19629,scan time=49.5665
LNVSAVAPNAQPIDAR
Intensity
m/z
C7H8NO2
C6H8NO2
HexNAc-36
HexNAc-18
HexNAc
HexNAcHex
y9
y10
y11
y12
y13
y2
y5
y9++
y8
Pep+HexNAc_2+
Pep_1+

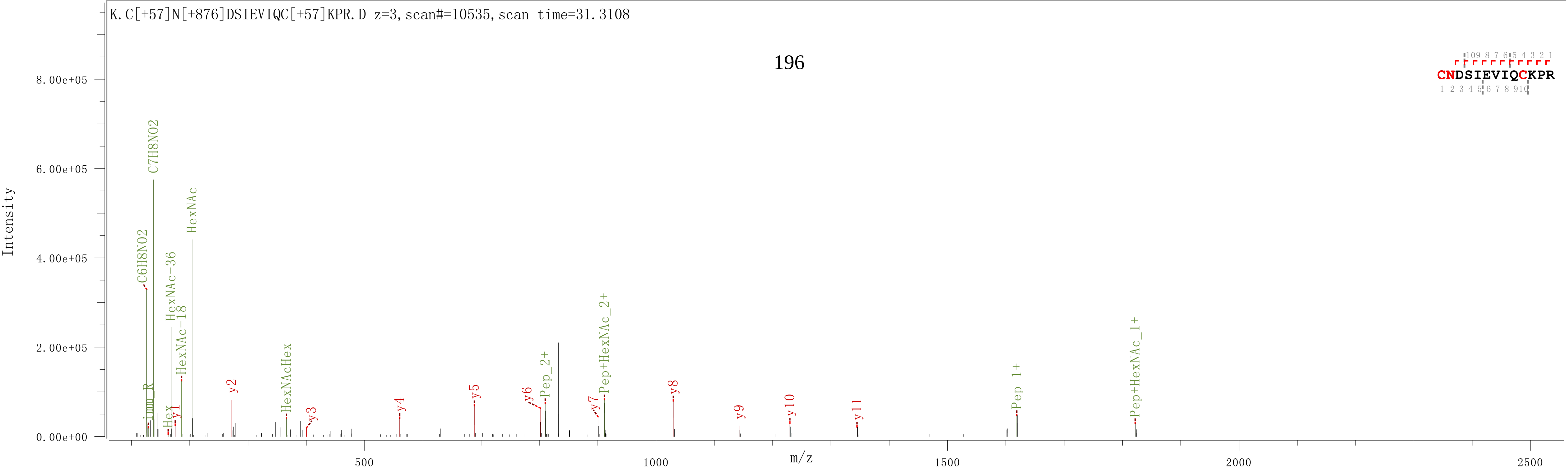

K.C[+57]N[+876]DSIEVIQC[+57]KPR.D z=3,scan#=10535,scan time=31.3108
CNDSIEVIQCKPR
Intensity
m/z
8.00e+05
6.00e+05
4.00e+05
2.00e+05
0.00e+00
500
1000
1500
2000
2500
C6H8NO2
C7H8NO2
HexNAc
HexNAc-36
HexNAc-18
Hex
y1
Imm_R
y2
HexNAcHex
y3
y4
y5
y6
Pep_2+
y7
Pep+HexNAc_2+
y8
y9
y10
y11
Pep_1+
Pep+HexNAc_1+

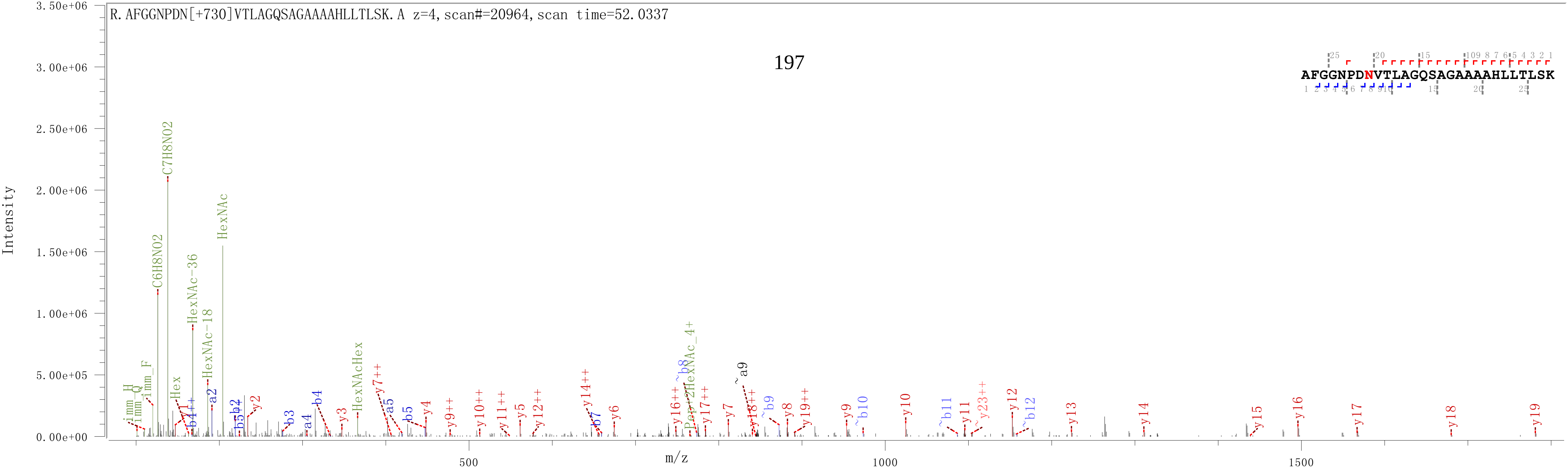
R.AFGGNPDN[+730]VTLAGQSAGAAAAHLLTLSK.A z=4,scan#=20964,scan time=52.0337
197
AFGGNPDNVTLAGQSAGAAAAHLLTLSK
Intensity
m/z
3.50e+06
3.00e+06
2.50e+06
2.00e+06
1.50e+06
1.00e+06
5.00e+05
0.00e+00
500
1000
1500
C7H8NO2
HexNAc
C6H8NO2
HexNAc-36
HexNAc-18
HexNAcHex
Hex
imm_H
imm_Q
imm_F
y1
y2
y3
y4
y5
y6
y7
y8
y9
y10
y11
y12
y13
y14
y15
y16
y17
y18
y19
b2
b3
b4
b5
b7
a2
a4
a5
~a9
~b8
~b9
~b10
~b11
~b12
y7++
y9++
y10++
y11++
y12++
y14++
y16++
y17++
y18++
y19++
~y23++
Pep+2HexNAc_4+

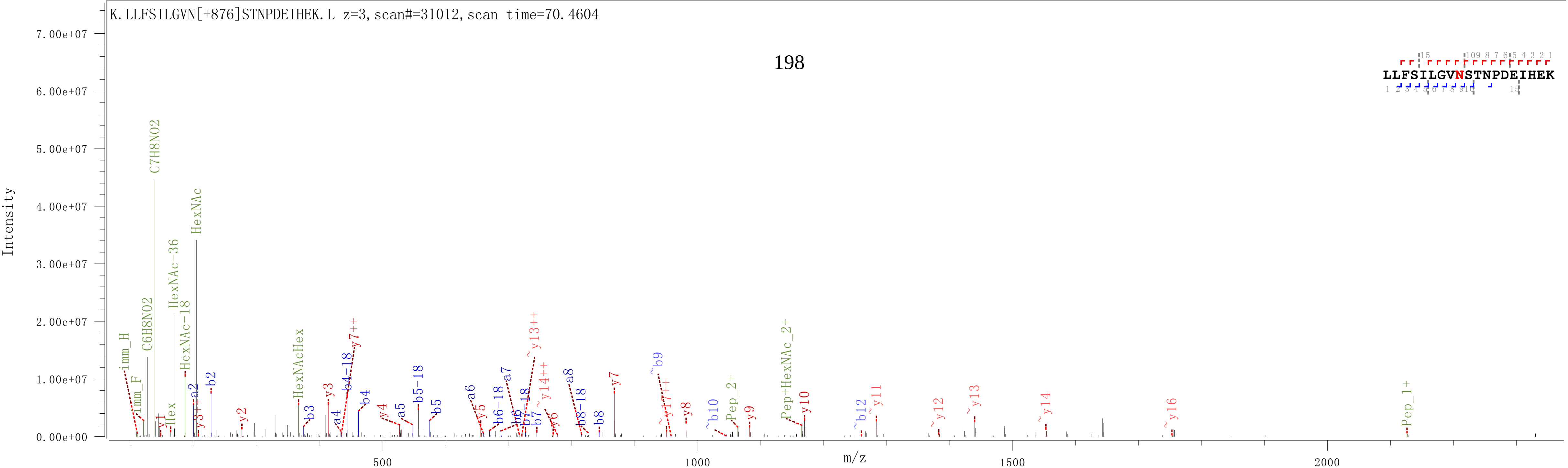
K.LLFSILGVN[+876]STNPDEIHEK.L z=3,scan#=31012,scan time=70.4604
Intensity
m/z
LLFSILGVNSTNPDEIHEK
C7H8NO2
HexNAc
HexNAc-36
HexNAc-18
C6H8NO2
Imm_H
Imm_F
HexNAcHex
Pep+HexNAc_2+
Pep_2+
Pep_1+
Hex
y7++
~y13++
~y14++
~b9
~y17++
b4-18
b2
a2
y3++
y1
y2
y3
b3
a4
b4
y4
a5
b5-18
b5
a6
y5
b6-18
a7
b6
b7-18
b7
y6
a8
b8-18
b8
y7
y8
~b10
y9
y10
~b12
~y11
~y12
~y13
~y14
~y16
0.00e+00
1.00e+07
2.00e+07
3.00e+07
4.00e+07
5.00e+07
6.00e+07
7.00e+07
500
1000
1500
2000

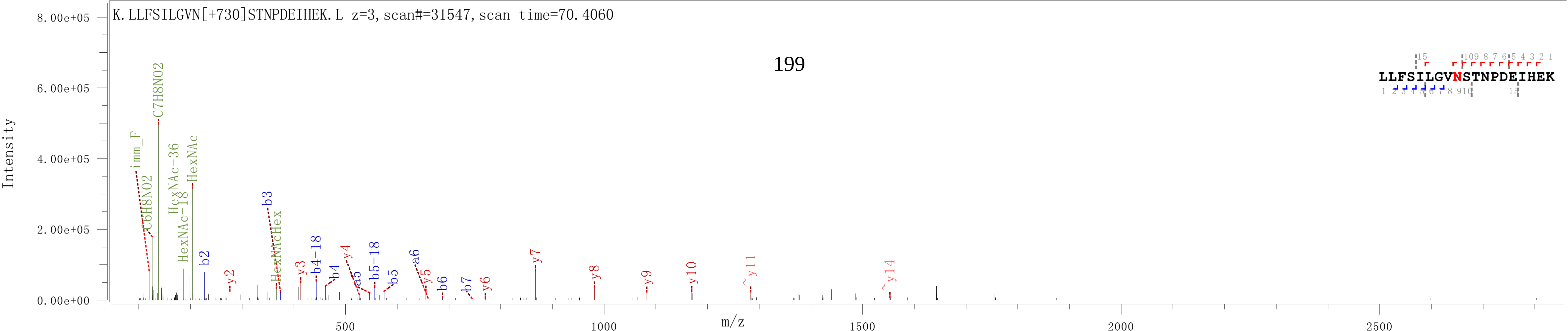
K.LLFSILGVN[+730]STNPDEIHEK.L z=3,scan#=31547,scan time=70.4060
Intensity
m/z
LLFSILGVNSTNPDEIHEK
imm_F
C6H8NO2
C7H8NO2
HexNAc-36
HexNAc-18
HexNAc
b2
y2
b3
HexNAcHex
y3
b4-18
b4
y4
a5
b5-18
b5
a6
y5
b6
b7
y6
y7
y8
y9
y10
~y11
~y14

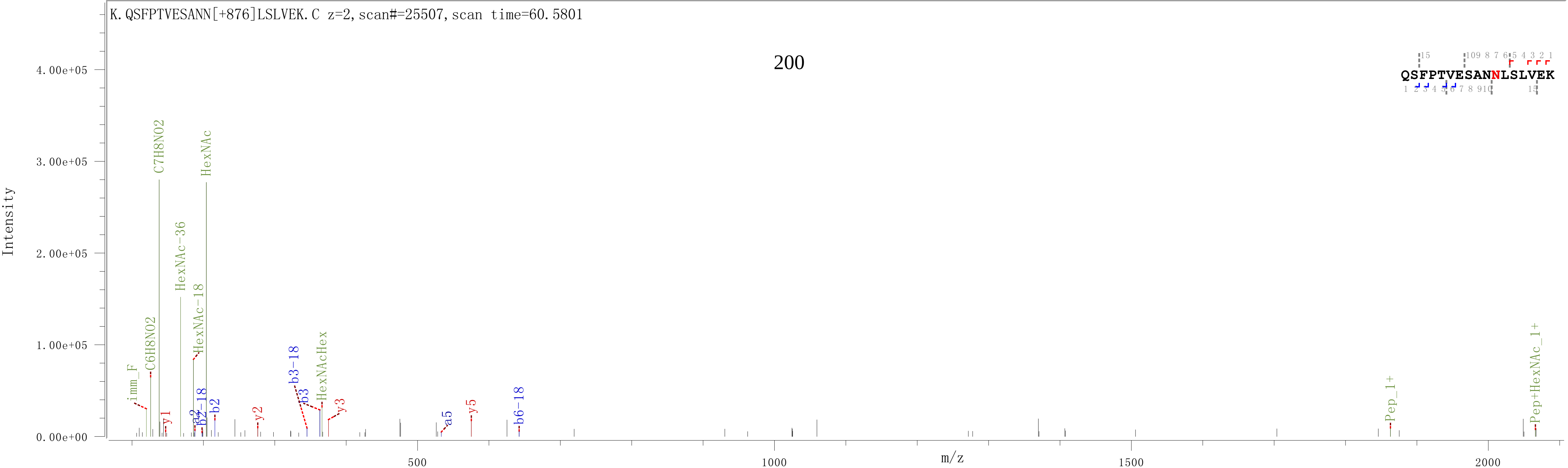
K.QSFPTVESANN[+876]LSLVEK.C z=2,scan#=25507,scan time=60.5801
QSFPTVESANNLSLVEK
Intensity
4.00e+05
3.00e+05
2.00e+05
1.00e+05
0.00e+00
m/z
500
1000
1500
2000
imm_F
C6H8NO2
C7H8NO2
HexNAc-36
HexNAc-18
HexNAc
y1
a2
b2-18
b2
y2
b3-18
b3
HexNAcHex
y3
a5
y5
b6-18
Pep_1+
Pep+HexNAc_1+
